# Physiological, Biochemical, and Root Proteome Networks Revealed New Insights Into Salt Tolerance Mechanisms in *Pongamia pinnata* (L.) Pierre

**DOI:** 10.3389/fpls.2021.771992

**Published:** 2022-01-24

**Authors:** Sureshbabu Marriboina, Kalva Madhana Sekhar, Rajagopal Subramanyam, Attipalli Ramachandra Reddy

**Affiliations:** Department of Plant Sciences, School of Life Sciences, University of Hyderabad, Hyderabad, India

**Keywords:** *A*_*sat*_/C_i_ curves, Chl *a* fluorescence, gas exchange, OJIP curves, JIP-test, proteomic analysis, nanoLC-MS/MS, root proteome

## Abstract

Cultivation of potential biofuel tree species such as *Pongamia pinnata* would rehabilitate saline marginal lands toward economic gains. We carried out a physiological, biochemical, and proteomic analysis to identify key regulatory responses which are associated with salt tolerance mechanisms at the shoot and root levels. *Pongamia* seedlings were grown at 300 and 500 mM NaCl (∼3% NaCl; sea saline equivalent) concentrations for 15 and 30 days, gas exchange measurements including leaf net photosynthetic rate (*A*_*sat*_), stomatal conductance (*g*_*s*_), and transpiration rate (*E*), and varying chlorophyll *a* fluorescence kinetics were recorded. The whole root proteome was quantified using the free-labeled nanoLC-MS/MS technique to investigate crucial proteins involved in signaling pathways associated with salt tolerance. *Pongamia* showed no visible salt-induced morphological symptoms. However, *Pongamia* showed about 50% decline in gas exchange parameters including *A*_*sat*_, *E*, and *g*_*s*_ 15 and 30 days after salt treatment (DAS). The maximum potential quantum efficiency of photosystem (PS) II (Fv/Fm) was maintained at approximately 0.8 in salt-treated plants. The thermal component of PSII (DIo) was increased by 1.6-fold in the salt-treated plants. A total of 1,062 protein species were identified with 130 commonly abundant protein species. Our results also elucidate high abundance of protein species related to flavonoid biosynthesis, seed storage protein species, and carbohydrate metabolism under salt stress. Overall, these analyses suggest that *Pongamia* exhibited sustained leaf morphology by lowering net photosynthetic rates and emitting most of its light energy as heat. Our root proteomic results indicated that these protein species were most likely recruited from secondary and anaerobic metabolism, which could provide defense for roots against Na^+^ toxicity under salt stress conditions.

## Introduction

Soil salinization is a growing problem in arid and semi-arid areas. For the past 3 decades, over 800 million ha, which is about 6% of the total landmass, have been converted to marginal saline lands (Munns and Gilliham, 2015). It is estimated that approximately 50% of land around the world will be affected by salinity by the year 2050 (Machado and Serralheiro, 2017; Qureshi et al., 2019). Global population growth and industrialization are further decreasing the accessibility of land and water for cultivation (Cosgrove and Loucks, 2015). On the other hand, improper irrigation management, inadequate water supply, and brackish water use on arable land lead to conversion of cultivable lands to saline lands. Over 10 million ha of arable lands are converted into marginal saline lands every year. It is estimated that about 124 trillion kilocalories worth of agriculture yield will be lost every year due to the salinization of arable lands (Machado and Serralheiro, 2017; Damania et al., 2019). Several attempts were made to utilize the saline arable lands for cultivation, including the rise of genetically engineered crops (Shukla et al., 2018). However, these attempts are largely unsuccessful because of salinity tolerance being governed by multigene networks and their coordination depending on soil environment (Quan et al., 2018). Recently, nitrogen-fixing tree species have been gaining immense importance not only to rehabilitate saline marginal lands but also to use these lands for economic gain (Marriboina and Attipalli, 2020a).

To survive under extreme saline conditions, plants need to adapt to various physiological and biochemical processes (Gupta and Huang, 2014). The presence of salts in soil restricts water entry across plants and thereby causes salinity-induced drought stress (Kaur and Nayyar, 2014). Plants maintain steady levels of chlorophyll, and photosynthetic accessory pigments could help in maintaining photosynthetic activity under extreme saline conditions (Marriboina et al., 2017). Salinity is known to reduce the uptake of water to limit stomatal conductance (*g*_*s*_) and transpiration rate (*E*), which could be an acclimation response under water-deprived conditions (Guha and Reddy, 2014). Excess accumulation of Na^+^ and Cl^–^ ions in plants leads to decrease in net photosynthetic rate due to reduced stomatal conductance. Salinity also affects leaf relative water content (RWC) and intercellular CO_2_ concentration (Marriboina et al., 2017). Thus, to thrive under high saline conditions, plants reduce primary activities, such as net CO_2_ assimilation/utilization, until favorable conditions appear (Eisa et al., 2012; Acosta-Motos et al., 2017). Fast chlorophyll fluorescence is known to be the best tool to assess the efficiency of photosystem (PS) II under stressful conditions (Murchie and Lawson, 2013). It is also an efficient indicator for salt stress tolerance and is used as the best tool for monitoring PSII photochemistry (Dongsansuk et al., 2013). Salinity stress reduces the maximal yield of PSII (Fv/Fm) because of the increased susceptibility of PSII, which is associated with several other non-photochemical and photochemical quenchers (Shin et al., 2020). Plants recruit various non-water electron donors such as proline and glycine betaine to compensate for electrons generated through photolysis of water at the donor side of PSII under salinity stress (Sengupta et al., 2013). Plants enhance several enzymes such as chloroplast oxidases (involved in carotenoid biosynthesis) and NAD(P)H-plastoquinone oxidoreductases to maintain plasto-quinine redox homeostasis, which enables stable electron transport rate across thylakoid membranes under extreme saline conditions (Bennoun, 2002). The root is the primary organ that senses salt stress and acts as a physical barrier to restrict Na^+^ ion distribution across plants (Marriboina and Attipalli, 2020a). Continuous accumulation of Na^+^ ions inside the root cell exerts high osmotic pressure on the cell wall (Horie et al., 2012). Plant synthesizes cell wall remodeling enzymes, including galactoside 2-alpha-L-fucosyltransferase 1 (FUT1) and probable UDP-arabinopyranose mutase 1 (RGP1), to protect cell wall turgor under salt stress (Tryfona et al., 2014; Saqib et al., 2019). In response to salt stress, plants also induce the synthesis of several protein species related to secondary metabolism, including flavonoid and anthocyanin biosynthesis, to defend against ROS damage (Chen et al., 2019). On the other hand, plants also trigger antioxidant defense systems to cope with salt-induced oxidative stress (Al-Kharusi et al., 2019). Antioxidant enzymes such as ascorbate peroxidase 1 (APX1), catalase 4 (CATA4), and monodehydroascorbate reductase (MDAR) are known to provide defense against ROS and help maintain superoxide levels under salt stress (Li et al., 2015; Sofo et al., 2015). However, an integrated analysis to demonstrate the co-existing adaptive and defensive mechanisms of roots and leaves conferring overall tolerance under salinity stress is crucial to understand the salt stress response of a specific plant.

*Pongamia pinnata* (*Millettia pinnata*) is a medium-sized leguminous tree belonging to the Fabaceae family (Marriboina et al., 2021). *Pongamia* seed oil with 50% oleic acid content can be used as potential feedstock for biofuel (Calica, 2017; Singha et al., 2019). With nitrogen-fixing ability, *Pongamia* can fix 47.4 mg of nitrogen per plant under normal conditions (Calica and Gresshoff, 2019). We have recently shown that *Pongamia* exhibits “stay green” leaf morphology under high salt stress conditions and restricts the uptake of Na^+^ ion into the leaves (Marriboina et al., 2021). However, root proteins involved in the uptake of salt have not been characterized so far. Various plants express proteins differentially to salt stress; therefore, it is important to understand the total proteome of roots from *Pongamia*. Uptake of salt from roots could directly influence the rate of photosynthesis. In the present study, we analyzed leaf gas exchange parameters, chlorophyll (Chl) *a* fluorescence and whole root proteome to unravel the deeper insights of PSII photochemistry and CO_2_ assimilation characteristics in *Pongamia*.

## Materials and Methods

### Plant Material, Growth Conditions, and NaCl Treatment

Freshly harvested seeds of *P. pinnata* (accession TOIL12) were obtained from Tree Oils India Limited (TOIL) (Zaheerabad, Telangana, India). The seeds were oven-dried at 40°C temperature overnight to remove excess moisture. Uniform-sized seeds were selected and washed thoroughly in 1% (v/v) HOCl solution for 5 min. The seeds were transferred on a moist cotton bed and incubated for 10 days in the dark for germination (Marriboina and Attipalli, 2020b). Furthermore, the germinated seeds were carefully transferred to pots filled with soil and sand mixture (3:1 w/w). The seedlings were grown for 30 days under greenhouse conditions (temperature 25 ± 1°C, relative humidity 60–70%, and natural photoperiod). After 30 days, synchronized seedlings were divided into two groups, control and salt stress seedlings. For salt stress, two salt concentrations were used, 300 and 500 mM NaCl. Salt treatment was given according to Marriboina et al. (2017), where seedlings were treated with salt stress for 30 days by following measured pot water holding capacity. Two time points were chosen for the study, 15 and 30 days. After each time point, physiological and biochemical studies were performed. Based on our previous studies (Marriboina et al., 2017; Marriboina and Attipalli, 2020a), *Pongamia* is associated with several physiological, molecular, and anatomical changes including Na^+^ vacuolar sequestration, root ultra-filtration (Marriboina and Attipalli, 2020a), and altered metabolome and phytohormonal changes 30 days after salt treatment (DAS) (Marriboina et al., 2021).

### Leaf Gas Exchange Measurements

Leaf gas exchange measurements were performed using a portable infrared gas analyzer (IRGA, LCpro-32070; ADC BioScientific Ltd., United Kingdom). Fully expanded second and third leaves were used for experimentation. All measurements were recorded between 10:00 and 11:30 h. The following conditions were maintained throughout the experimentation: saturating photosynthetically active radiation (PAR) of 1,600 μmol m^–2^ s^–1^ supplied by a LED light source (LCpro Lamp 32070–Broad; ADC BioScientific Ltd., United Kingdom) attached to a leaf chamber. Air temperature was maintained at about 25–26°C and relative humidity approximately 55–60%. Photosynthetic parameters such as light-saturated net photosynthetic rate (*A*_*sat*_), stomatal conductance (*g*_*s*_), intercellular CO_2_ concentration (C_i_) and transpiration rate (*E*) were recorded. Leaf instant water use efficiency (WUE_i_) was calculated as *A_*sat*_/E*.*A*_*sat*_/Q, and *A*_*sat*_/C_i_ curves were measured according to Sekhar et al. (2015). *A*_*sat*_/Q curves were determined with increasing PAR between ∼250 and ∼2,000 μmol m^–2^ s^–1^ supplied by the LED light source attached to the leaf chamber. Air temperature was maintained at about 25–26°C and relative humidity approximately 55–60%.

*A*_*sat*_/C_i_ curves were determined with increasing CO_2_ between ∼50 and ∼1,000 μmol mol^–1^ supplied by an external CO_2_ source attached to the leaf chamber. Air temperature was maintained at about 25–26°C and relative humidity approximately 55–60%.

### Measurement of Total Chlorophyll Content, Leaf Relative Water Content, and Photosystem II Efficiency

Measurements of PSII efficiency and Chl *a* fluorescence were performed using a portable Handy PEA (Plant Efficiency Analyzer-2126) fluorometer (Hansatech Instruments Ltd., Kings Lynn Norfolk, United Kingdom) on fully expanded second and third leaves of the plant. All the measurements were recorded between 10:00 and 11:30 h. The leaves were kept in the dark and incubated for 30 min before starting the experimentation using leaf clips, where all the reaction centers will be closed and the minimum Chl *a* fluorescence (Fo) will be close to zero (Guha et al., 2013). Fluorescence intensities were recorded by illuminating a saturating light intensity of 3,000 mol m^–2^ s^–1^ of a 650-nm peak wavelength generated by an array of three light-emitting diodes for 1 s. Data presented are average of three independent replicates. JIP parameters were analyzed using the Bioanalyzer software (Hansatech Instruments Ltd., Kings Lynn Norfolk, United Kingdom). Chlorophyll and total carotenoid contents were measured using standard protocols (Hiscox and Israelstam, 1979; Minocha et al., 2009). Leaf RWC was measured by following the methods of Barrs and Weatherley (1962).

### Estimation of Proline and Total Soluble Sugar Contents

Leaf proline content was measured according to Ábrahám et al. (2010). Fresh leaf samples (0.5 g) were powdered using liquid nitrogen followed by homogenization with 5 ml of 3% (v/v) sulfosalicylic acid. The homogenate was centrifuged at 10,000 × *g* at room temperature for 15 min. The supernatant (2 ml) was mixed with 2 ml of acid ninhydrin and 2 ml of glacial acetic acid, and incubated for 1 h in a boiling water bath. After incubation, the reaction mixture was cooled to room temperature, and 4 ml of toluene was added and mixed vigorously by vortexing for 15–20 s. The upper pink-colored toluene layer was separated carefully through a separating funnel, and absorbance was read at 520 nm with a UV-visible spectrophotometer (Eppendorf BioSpectrometer; Eppendorf, Germany) using toluene as blank. Proline concentration was determined as μg g^–1^ FW.

Total soluble sugars were estimated according to Jain et al. (2017). A total of 100 mg of finely ground dry leaf powder were placed in clean glass tubes. For sugar extraction, 5 ml of 80% ethanol was added to the leaf powder and kept in a boiling water bath at 95°C for 10 min. After extraction, the tubes were centrifuged at 2,500 rpm for 5 min, and the supernatant was collected using a fresh glass tube. The above steps were repeated three times. The supernatants were pooled for sugar analysis. To 1 ml of supernatant, 1 ml of phenol and 5 ml of sulfuric acid was added and kept in the dark for 10 min after thoroughly mixed. Furthermore, the reaction mixture was kept in a boiling water bath at 25–30°C for 20 min. The reaction mixture was allowed to cool, and absorbance was taken at a 490-nm wavelength using a UV-visible 160A spectrophotometer (Shimadzu, Tokyo, Japan). Glucose concentration was determined using the standard curve of 0–10 mg ml^–1^ concentration range).

### Root Protein Extraction and Quantification

Total root protein was extracted as described in Saravanan and Rose (2004), with some modifications (Supplementary Figure 1). Whole roots of the control and 500 mM NaCl-treated plants were collected and finely ground in liquid nitrogen with motor and pestle. Total root proteins were extracted as described in Saravanan and Rose (2004), with some modifications. Approximately 1 g of root powder was taken in a 15-ml falcon tube (Genaxy, India) and suspended in 4 ml of an extraction buffer containing 0.5 M Tris–HCl (pH 7.5), 0.7 M sucrose, 0.1 M KCl, 50 mM EDTA, 2% β-mercaptoethanol, and 1 mM PMSF. An equal volume of Tris-saturated phenol (pH 7.5) was added to the extract suspension after thorough mixing, and the whole suspension was further mixed for 30 min at 4°C in a rotor spin cyclomixer. Tris-saturated phenol was prepared by mixing an equal volume of Tris–HCl (pH 7.5) and phenol with continuous stirring for 3–4 h. The lower phenolic layer was separated, and we added an equal volume of Tris–HCl (pH 7.5) with continuous stirring for 2–3 h. The lower phenolic layer was collected and stored in an amber color glass bottle at 4°C. The sample mixture was centrifuged at 5,000 × *g* for 30 min at 4°C. The upper phenolic phase was collected carefully, and an equal volume of extraction buffer was added to it. The above steps were repeated one more time, and the phenolic phase was re-extracted. Four volumes of ice cold 0.1 M ammonium bicarbonate in methanol were added to the final collected phenolic phase, and incubated overnight at −20°C for protein precipitation. On the next day, the samples were centrifuged at 10,000 × *g* for 30 min at 4°C. The pellet was washed thrice with ice-cold methanol and twice with acetone, and air-dried for a few minutes.

The final pellet was dissolved in 200 μl of a rehydration solution containing 8 M urea, 2 M thiourea, 30 mM DTT, 4% CHAPS, and 8% IPG buffer of pH range 4–7 (GE Healthcare, United States), and protein concentration was determined using An RC-DC protein assay kit (Bio-Rad, Hercules, CA, United States) with BSA as standard (standard curve of 0–100 mg ml^–1^ concentration).

### nLC-MS/MS Analysis

A total of 100 μg of the final pellet was treated with 100 mM DTT for 1 h at 95°C followed by 250 mM iminodiacetic acid (IDA) for 45 min at room temperature in the dark. The sample suspension was incubated with trypsin at 37°C for overnight digestion. Trypsin-digested peptides were extracted in 0.1% formic acid solution at 37°C for 45 min. The solution was centrifuged at 10,000 × *g* for 15 min at room temperature, and the supernatant was collected in a fresh tube for vacuum drying. The final sample was solubilized in 20 μl of 0.1% formic acid. For separation of peptides, 10 μl of injection volume was loaded on C_18_ UPLC column, and peptides were separated on Waters Synapt G2 Q-TOF (Water, United States). For LC-MS analysis, 10 μl of the sample was injected into an ACQUITY UPLC system (Waters, United States) equipped with an ACQUITY UPLC BEH C_18_ column (Waters, United States) (150 mm × 2.1 mm × 1.7 μm), SYNAPT G2 QTOF (Waters, United States), and an electrospray ionization (ESI) source. A sample analysis was performed on the positive mode by applying 3,500-V capillary voltage and 30-L cone gas flow per hour. Source and desolvation glass flow was maintained at 1.8 and 800 L per h, and the temperatures of source and desolvation were 150 and 350°C, respectively. Protein range was used from 50 to 150 Da. Trap and transfer collision energy were maintained constantly at 6 V, and ramp collision energy was set at 20 V and increased up to 45 V. Total acquisition time was 60 min, and solution flow rate was 300 nl min^–1^. The mobile phase consisted of 0.1% formic acid in water (solvent A) and 0.1% formic acid in acetonitrile (solvent B). A linear 60 min gradient consisted of solvent A 98% and solvent B 2% for 1 min, solvent A and B 50% for 29 min, solvent A 20% and solvent B 80% for 15 min, followed by 15 min solvent A 98% and solvent B 2%. A washing solution was used at the end of each program to reduce carry-over between samples.

### Protein Identification

The raw data acquired from the above analysis were processed using PLGS software 3.0.2 (Waters, United States; identification and expression algorithm), within which data process and database search were performed. The source of the sample being Fabaceae proteins for two sample sequences in FASTA format was downloaded from Swiss-Prot and used for searching peptides present in the sample. On each run, the sample was processed using the following search parameters in the software: peptide tolerance 50 ppm, fragment tolerance 100 ppm, minimum number of fragment matches for peptides 2, and minimum number of fragment matches for proteins 5; carbamidomethylation of cysteine and oxidation of methionine were selected as fixed and variable modifications, respectively. Universal Protein Resource (UniProt) (Fabaceae, reviewed protein) was used as the database against which the search was performed.

### Gene Ontology and Bioinformatics Analysis

The identified protein species in this study were annotated based on their molecular function, biological process, and cellular component by Gene Ontology (GO) annotation using the UniProt database. Hierarchical cluster analysis was performed on correlation values using the R.3.61 statistical package. Network analysis was performed using Cytoscape bioinformatics software version 3.7.2.

### Statistical Analyses

All the statistical analyses were performed on three biological replicates (*n* = 3). The significant difference between average values of control and salt-treated plants was measured by Student’s *t*-test. Hierarchical cluster matrices and protein–protein networks were constructed based on correlation values and *P*-values less than 0.05 using the R.3.6.1 statistical package.

For physiological and biochemical analysis, a two-way ANOVA test was performed between the control and salt-treated plants for 15 and 30 days to measure the *P*-values; ns, not significant; *, *P* < 0.05; ^**^, *P* < 0.01; and ^***^, *P* < 0.001.

## Results

### Salt-Induced Changes in Morphology, Leaf Gas Exchange and Biochemical Parameters, and Chlorophyll Content

**FIGURE 1:**
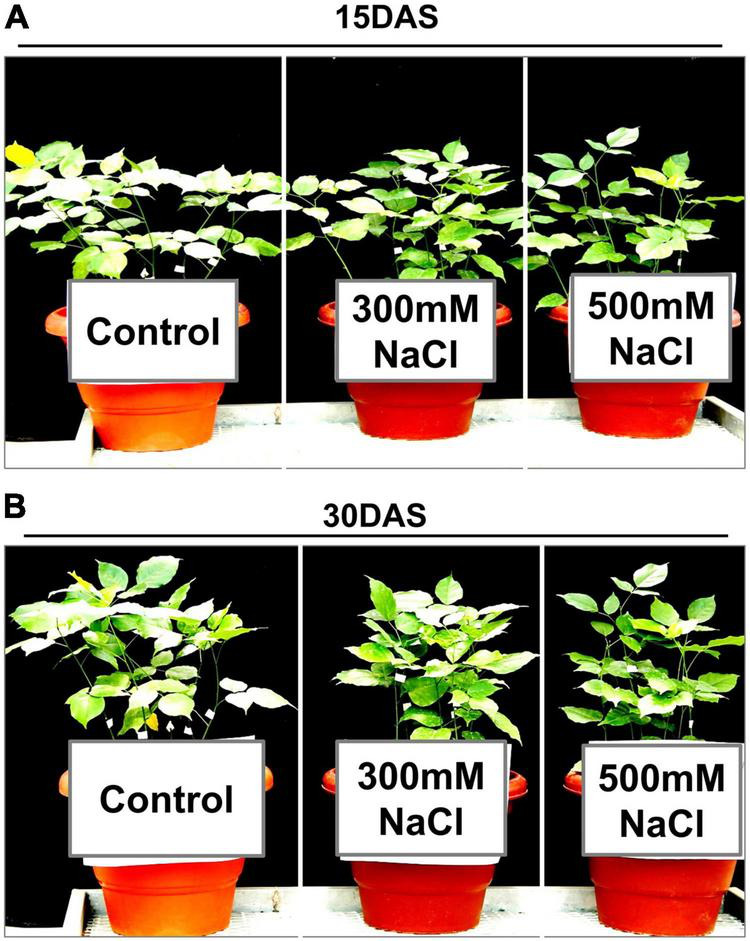
Effect of salinity stress on the plant morphology of *Pongamia pinnata*. Thirty-day-old soil-grown plants were treated with three different salt concentrations: 0 (control), 300, and 500 mM NaCl for **(A)** 15 and **(B)** 30 days. Error bars represent the mean ± SD (*n* = 4).

**FIGURE 2:**
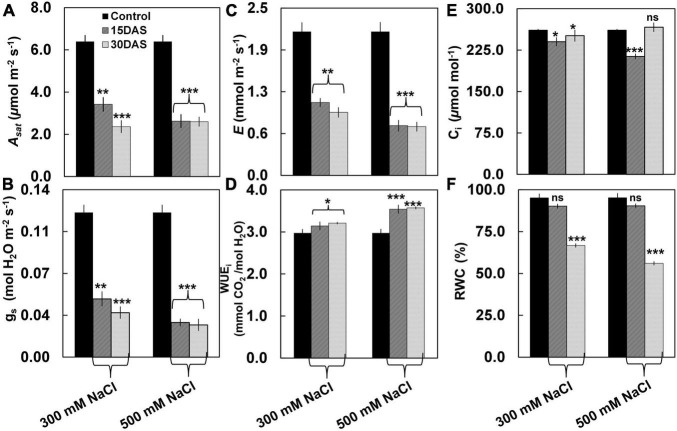
Effect of salinity stress on leaf photosynthetic performance of *P. pinnata*. Variations in **(A)** light-saturated net photosynthetic rate (*A*_*sat*_) (μmol m^–2^ s^–1^), **(B)** stomatal conductance (*g*_*s*_) (mol H_2_O m^–2^ s^–1^), **(C)** transpiration rate (*E*) (mmol m^–2^ s^–1^), **(D)** instant water use efficiency (WUE_i_) (mmol m^–2^ s^–1^), **(E)** intercellular CO_2_ (C_i_) (μmol m^–1^), and **(F)** relative water content (RWC) in *P. pinnata* grown under control and salt treatment conditions. Error bars represent the mean ± SD (*n* = 4). Two-way analysis of variance (ANOVA) test was performed to measure *P*-values ns, not significant; *, *P* < 0.05; **, *P* < 0.01; and ***, *P* < 0.001.

Despite having no visible salt-induced symptoms (Figure 1), the salt-treated plants showed significant changes in gas exchange parameters such as *A*_*sat*_, *g*_*s*_, *E*, and WUE_i_ (Figure 2). A significant decrease was observed in *A*_*sat*_ by ∼45% in the salt-treated plants 15 and 30 DAS (Figure 2A). Similarly, *g*_*s*_ values showed a significant decrease ranging from ∼0.05 to 0.03 μmol m^–2^ s^–1^ in the 300 and 500 mM NaCl-treated plants 15 and 30 DAS, respectively (*P* < 0.01; Figure 2B). We also observed a significant reduction in *E* by ∼65 and ∼60% in the 300 mM NaCl-treated plants, while ∼40% was observed in 500 mM NaCl-treated plants 15 and 30 DAS, respectively (Figure 2C). Conversely, WUE_i_ increased progressively in the salt-treated plants (Figure 2D). However, C_i_ values varied in the salt-treated plants 15 and 30 DAS (Figure 2E). The percentage of RWC did not change 15 DAS, while ∼64 and ∼55% decrease was observed 30 DAS in the 300 and 500 mM NaCl-treated plants, respectively (Figure 2F).

**FIGURE 3:**
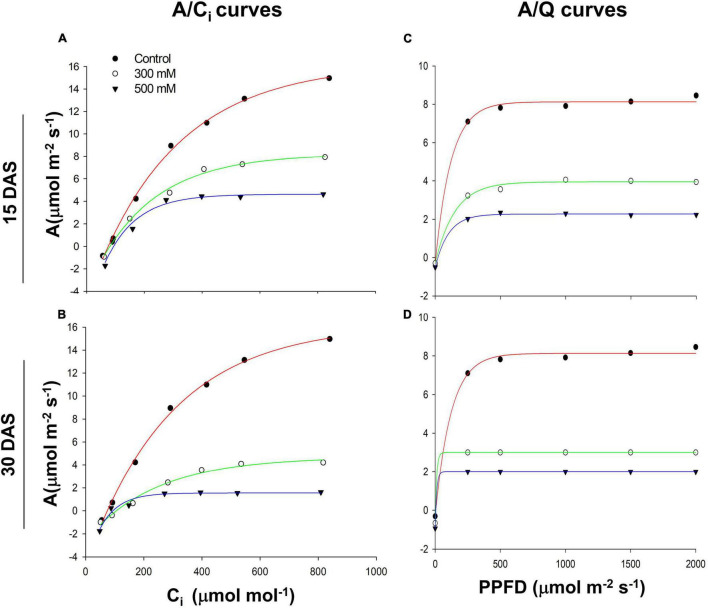
Salt-induced response of photosynthetic rates to intercellular (CO_2_) (C_i_) **(A)** 15 days after salt treatment (DAS) and **(B)** 30 DAS. Photosynthetic rates to photosynthetic photon flux density (PPFD) **(C)** 15 and **(D)** 30 DAS in *Pongamia* grown under control and salt-treated conditions.

*A*_*sat*_ followed a hyperbolic curve pattern with a decreased trend in the 300 and 500 mM NaCl-treated plants 15 and 30 DAS (Figures 3A,B). We also observed a similar trend in *A*_*sat*_ decline with various intensities of PPFD in both the 300 and 500 mM NaCl-treated plants 15 and 30 DAS (Figures 3C,D). Chlorophyll content did not change significantly in the salt-treated plants 15 and 30 DAS (Supplementary Table 1). Chl *a*, Chl *b*, total chlorophyll, total carotenoid content, and Chl *a/b* ratios showed no differences with respect to controls in the salt-treated plants 15 and 30 DAS. Leaf proline content did not change significantly 15 DAS, while its levels slightly increased 30 DAS in both the 300 and 500 mM NaCl-treated plants. Leaf total soluble sugar content did not change significantly 15 DAS, while its levels slightly decreased 30 DAS in the 300 and 500 mM NaCl-treated plants.

### Salt-Induced Changes in Chlorophyll *a* Fluorescence, JIP Parameters, and Phenomenological Fluxes

**FIGURE 4:**
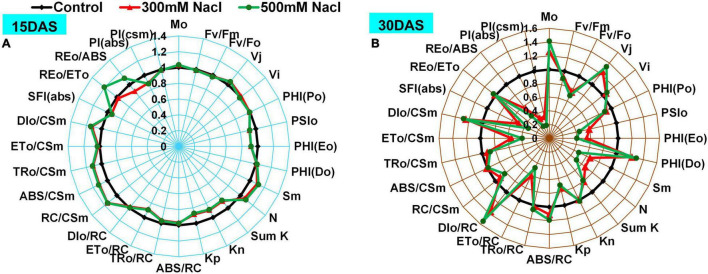
Radar plot depicting salt stress-induced changes in Chl *a* fluorescence transient parameters in *Pongamia* grown under 300 and 500 mM NaCl concentration for **(A)** 15 and **(B)** 30 day. All variables were deduced from the OJIP-test analysis. Data are mean ± SD (*n* = 4).

Changes in Chl *a* fluorescence intensities were monitored using radar plots and normalized with respect to controls (Figures 4A,B and Supplementary Table 1). The Mo and PHI (Do) showed no change 15 DAS, whereas a significant increase was observed 30 DAS in both the 300 and 500 mM NaCl-treated plants. Furthermore, Fv/Fm, Fv/Fo, SFI(abs), and PI(csm) did not change significantly 15 DAS, while a slight decrease was recorded 30 DAS in the 300 and 500 mM NaCl-treated plants. J (V_*j*_), Fo/Fm, ABS/RC, and DIo/RC showed no differences 15 DAS, whereas these increased significantly 30 DAS in the salt-treated plants. PHI (Po) value did not change significantly in the salt-treated plants 15 DAS, while a slight reduction was observed 30 DAS in the 300 and 500 mM NaCl-treated plants. A moderate decrease in PSIo, PHI(Eo), ETo/RC, PI(abs), and SumK was recorded 15 DAS, but these values significantly declined 30 DAS. Sm, Kp, area, and N were marginally high 15 DAS but showed a significant decrease 30 DAS in the 300 and 500 mM NaCl-treated plants. An increasing trend was observed in Fo 15 and 30 DAS in the salt-treated plants. Kn, Fm, and Tfm values were slightly decreased with respect to the controls 15 DAS but returned to control values 30 DAS in the salt-treated plants. RC/CSm showed a significant increase 15 DAS, while it was significantly decreased 30 DAS in the 300 and 500 mM NaCl-treated plants. REo/ETo showed a moderate increment in the 300 mM NaCl-treated plants, but showed no apparent change in the 500 mM NaCl-treated plants 15 DAS. REo/ABS was slightly increased in the 300 mM NaCl-treated plants and was moderately low in the 500 mM NaCl-treated plants 15 DAS. REo/ABS was significantly decreased 30 DAS in the 500 mM NaCl-treated plants. JIP parameters such as Sm/Tfm, PHIo/(1-PHIo), PSIo/(1-PSIo), DF, and OCE showed progressive decrease with treatment time in the salt-treated plants (Supplementary Table 2). The changes of phenomenological fluxes in the control and salt-treated plants were represented as the leaf energy pipeline model (Supplementary Figure 2). ETo/CSm was progressively decreased with increment of treatment time in the 300 and 500 mM NaCl-treated plants. In contrast, DIo/CSm showed a large difference with respect to the controls 15 and 30 DAS in the salt-treated plants, while a marginal decrease was observed in TRo/CSm in the salt-treated plants 15 and 30 DAS.

### Salt-Induced Changes in OLKJIP Transients

**FIGURE 5:**
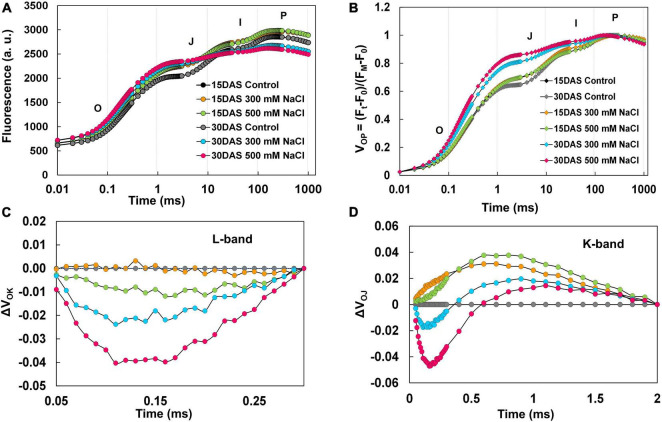
Polyphasic chlorophyll *a* transients for O-K, O-J, O-I, and I-P phases in leaves of *P. pinnata* under salt-treated conditions. **(A)** Raw Chl *a* fluorescence transient curves observed florescence intensity recorded between 0.1 and 1,000 ms time period; **(B)** double-normalized Chl *a* fluorescence transients recorded between extreme O (Fo) and P (F_M_) phases [V_OP_ = (F_t_ – Fo)/(F_M_ – Fo)]; **(C)** kinetic difference between control and treated values of ΔV_OK_ (L-band) with respective controls represented by black and gray circles; **(D)** kinetic difference between control and treated values of ΔV_OJ_ (K-band) with respective controls represented by black and gray circles; data are mean ± SD (*n* = 4).

*Pongamia* followed a typical polyphasic (OJIP) rise in Chl *a* fluorescence, and the pattern of raw fluorescence (F_M_ − F_O_ = F_V_) OJIP curves showed no difference between the control and treated plants (Figure 5A). To see further insights into variance in OJIP transients, double normalization was carried out. The double normalized V_op_ fluorescence showed a clear difference at the O-J intermediate step in the 300 and 500 mM NaCl-treated plants 15 and 30 DAS compared to respective controls (Figure 5B). Subsequently, to investigate further variance in fluorescence kinetics, normalization and subtractions were performed. Normalized relative variable fluorescence between O and K phase (50–300 μs), expressed as V_OK_ (Supplementary Figure 3A), and respective kinetic differences are depicted as ΔV_OK_ in Figure 5C. A negative bell-shaped L-band with maximum peak at around 150 μs was observed in the 500 mM NaCl-treated plants 15 and 30 DAS. The variable fluorescence between O and J phase (50 μs to 2 ms), represented as V_OJ_ (Supplementary Figure 3B), and respective kinetic differences are shown as ΔV_OJ_ in Figure 5D. A positive K-band with a maximum peak was observed between 50 μs and 1 ms in the 300 mM NaCl-treated plants 15 and 30 DAS. In the 500 mM NaCl-treated plants, negative K-band values were recorded between 0 and 50 μs. Conversely, these values returned to positive between 50 μs and 1 ms 15 and 30 DAS in the salt-treated plants. The fluorescence data between O and I [50 μs to 20 ms (<1)], expressed as V_OI_ (Supplementary Figure 3C), and respective kinetic differences are shown as ΔV_OI_ in Supplementary Figure 3D. Maximum negative peak values were reported between 100 μs and 5 ms. The normalized relative variable fluorescence between I and P phase (30–180 ms), depicted as V_IP_ (Supplementary Figure 3D), and respective kinetic differences are depicted as ΔV_IP_ in Supplementary Figure 3F. V_IP_ values followed a hyperbolic curve pattern (Supplementary Figure 3E), and it was fitted into the Michaelis–Menten equation to calculate 1/2 V_IP_, i.e., equal to Km value. Fifteen DAS, Km increased, but it was decreased 30 DAS in the 300 mM NaCl-treated plants. Additionally, in the 500 mM NaCl-treated plants, the Km values followed control patterns 15 DAS and decreased significantly 30 DAS. Similarly, ΔV_IP_ values showed positive deviation with respect to controls in 300 mM NaCl-treated plants 15 and 30 DAS, while negative ΔV_IP_ values were recorded 15 and 30 DAS in the 500 mM NaCl-treated plants (Supplementary Figure 3F).

### Identification of Proteins by nLC-MS/MS and Gene Ontology Analysis

A total of 1,062 abundant protein species (APs) were identified in both the control and salt-treated plants (Supplementary Table 3). The APs of *Pongamia* showed sequence homology with *Glycine* sp. (22%), *Pisum* sp. (21%), and *Medicago* sp. (10%), with sequence search similarity in the UniProt database (Supplementary Figure 4A). The APs were categorized into two groups, commonly abundant protein species (CAPs; proteins present in both the control and salt-treated plants) and specific to the control and salt-treated plants (Supplementary Tables 3,4). Furthermore, these CAPs were categorized based on values of log_2_ fold change (≤0.5 or ≥2) into three groups: high abundant protein species (HAPs), low abundant protein species (LAPs), and unchanged protein species (Supplementary Table 3). In *Pongamia*, most of the root protein species belonged to the unchanged protein category (82.4%), while the percentage of HAPs and LAPs was ∼7.8 and ∼9.8%, respectively (Supplementary Figure 4B). Based on biological and molecular functions (GO analysis), the APs were classified into 28 groups belonging to various metabolic pathways, namely, carbohydrates, lipids, amino acids, fatty acids, secondary metabolism, pigment metabolism, seed storage, transport, photosynthesis, defense response, cell cycle, signal transduction, cell wall synthesis, catalytic activity, DNA binding, carboxylic acid biosynthesis, mRNA processing, DNA repair, protease inhibitor, proteolysis, catalytic activity, chaperone, cytoskeleton, growth and developmental process, pathogenesis, replication, transcription, and translation regulatory proteins.

### Secondary Metabolism and Seed Storage Proteins

**FIGURE 6:**
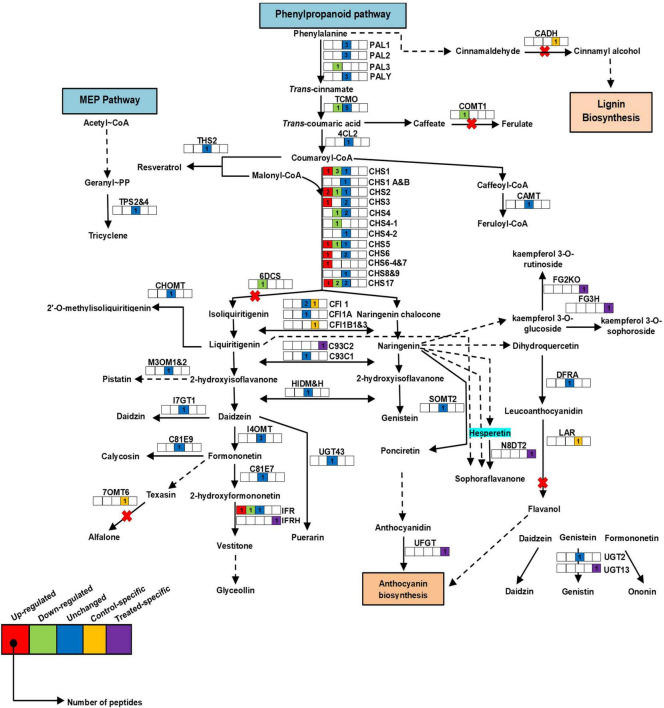
Schematic representation of secondary metabolic pathway in roots of *P. pinnata*. Relative expression of protein species related to the phenylpropanoid pathway. Color key represents fold expression, and number indicates identified peptides: red color: high abundant protein species; green color: low abundant protein species, blue color: unchanged protein species; yellow color: control specific protein species; violet color: treated specific protein species. CHS, chalcone synthase; PAL, phenylalanine ammonia lyase. For protein abbreviations, see Supplementary Table 1.

In *Pongamia*, protein species of secondary metabolism such as chalcone synthases (CHS) were highly abundant in roots of the salt-treated plants, namely CHS1_Q9SML4/P30073/Q01286/P24826/P51083, CHS2_ P51084/P17957/P30074/Q01287, CHS3_P51085/P19168/O23883, CHS4_P51086/P30075/O23882, CHS4-1_P51077, CHS5_O23884/P48406/P51078/P51087, CHS6_Q01288/P30080/P51088, CHS6-4_P51079, CHS7_P30081, CHS8_P30076, CHS17/Y_O22586/P49440/P51089/P23569, isoflavone reductase (IFR_P23569/Q00016/P52575), and isoflavone reductase homolog (IFRH_P52581) (Figure 6 and Supplementary Figure 5A; for abbreviations, see Supplementary Table 3). There were several protein species of CHS, which showed a significant decrease, namely CHS1_Q9SML4/P30073/P30073, CHS2_P51084, CHS4_ O23882, CHS4-1_P51077, CHS5_O23884, CHS17/Y_P49440/P51089, and IFR_P52576 in the roots of *Pongamia* under salt stress. However, protein species, namely, caffeic acid 3-*O*-methyltransferase (COMT1_P28002), favin (LEC_P02871), phenylalanine ammonia lyase class 3 (PAL-3_P19143), NAD(P)H-dependent-6′-deoxychalcone synthase (6DCS_P26690), and *trans*-cinnamate-4-monooxygenase (TCMO_Q42797), and their relative abundance were significantly low with respect to the controls. Additionally, protein species such as isoflavone reductase homolog (IFRH_P52581), naringenin-8-dimethylallyltransferase 2 (chloroplast origin) (chlN8DT2_B1B5P4), 2-hydroxyisoflavanone synthase (C93C2_Q9SXS3), C93C1_Q9SWR5, and flavonoid-3-*O*-glucosyltransferase (UGFGT_A6XNC6) were only abundant in roots of the salt-treated plants.

Seed storage protein species such as vicilin (VCLC_P13918/P08438), legumain (LEGU_P49046), arachin (ARA5_P04149), arachin Ahy-3 (AHY3_Q647H2), basic 7S globulin (7SB-1_P13917), legumin B2 (LEGB-2_P16078), LEGB-4_P05190, LEGB-6_P16079, LEGB-7_P16080, LEG-K_P05693, and LEG-J_P05692 were highly abundant in the salt-treated roots of *Pongamia*, while arcelin 5A (ARC5A_Q42460), ARC5B_Q41116 and albumin 2 (ALB2_D4AEP7) were low under salt stress (Supplementary Figure 5B). Notably, protein species such as LEGA-2_P15838, ALB1-D_P62929, seed-agglutinin 2 (LCS2_Q41161), ALB1_Q9FRT8, ARC1_P19329, and phaseolin-β-type (PHSB_P02853) showed high abundance only in roots of the salt-treated plants. Protein species related to saponin metabolism showed significant change under salt stress. Saponins-β-amyrin-11-oxidase (BAMO_B5BSX1) showed significant increase, while β-amyrin synthase (BAMS_Q9LRH8) and soyasaponin III rhamnosyltransferase (SGT3_D4Q9Z5) were decreased under salt stress.

### Primary Metabolism

High abundance of cell wall synthesis and energy metabolism-related protein species, namely, FUT1_Q9M5Q1, RGP1_O04300, PDC2_P51851, isocitrate lyase 1 (ACEA1_P45456), granule-bound starch synthase 2 (chlSSG2_Q43093), and malate dehydrogenase (chlMDHP_P21528/O48902) in the salt-treated roots was recorded (Supplementary Figure 6). However, a significant decrease was observed in protein species such as ribulose bisphosphate carboxylase large chain (RBL_O62964/O20304/A4GG89/O62943), malate dehydrogenase (MDHC_O48905), probable mannitol dehydrogenase (MTDH_O82515), sucrose synthase (SUS_Q01390), glucose-1-phosphate adenylyltransferase small subunit 2 (GLGS_P52417), pectinesterase 3 (PME3_Q43111), and polygalacturonase inhibitor 1 (PGIP1_P35334) in roots of the salt-treated plants (Supplementary Figure 7). Furthermore, protein species such as UGT13_A0A067YBQ3, UDP-glucose-4-epimerase GEPI48 (GALE2_O65781), chlRBS_Q42822, chlRBS1_P00865, RUBB_P08927, RuBisCO-associated protein (RUAP_P39657), fructose-1,6-bisphosphatase (F16P2_Q8RW99), isocitrate dehydrogenase (chlIDHP_Q40345), inactive UDP-glycosyltransferase79A6 (FG2KI_U3THC0), IDHC_Q06197, UDP-glycosyltransferase79A6 (FG2KO_I1LCI8), non-specific lipid-transfer protein 1 (NLTP_A0A161AT60), NLTP3_A0AT30, CASP-like protein 2D1 (CSPL4_C6T2J5), CSPL5_C6SZP8, and digalactosyl-diacylglycerol synthase 2 (chlDGDG2_Q6DW75) were abundant only in roots of the salt-treated plants.

*S*-adenosylmethionine synthase (METK_A4PU48) increased significantly, while γ-glutamyl hydrolase (GGH_P93164) and adenosylhomocysteinase (SAHH_P50246) decreased in the salt-treated roots of *Pongamia* (Supplementary Figure 8). Protein species such as ketol-acid reductoisomerase (chlILV5_O82043), pyridoxal-5′-phosphate synthase subunit PDX1 (PDX1_Q9FT25), aspartate carbamoyltransferase 1 (chlPYRB1_Q43086), arginine decarboxylase (SPE1_Q43075), isoaspartyl peptidase/L-asparaginase (ASPG_P50288), glutamate-1-semialdehyde-2,1-aminomutase (chlGSA_P45621), aspartate aminotransferase 1 (AAT1_P28011), serine carboxypeptidase-like (CBPX_Q41005), and ornithine carbamoyltransferase (chlOTC_Q43814) were observed only in roots of the salt-treated plants.

### Hormone Metabolism and Signal Transduction

High abundance of ABA-responsive protein 18 (ABR18_Q06930) and auxin-induced protein 6 (IAA6_P49680) was observed in response to salt stress (Supplementary Figures 9, 10). Additionally, significant increase was observed in serine/threonine-protein phosphatase catalytic subunit A (PP2A_Q06009), calcium-dependent protein kinase SK5 (CDPK-SK5_P28583), and phytochrome-associated serine/threonine protein phosphatase (FYPP_Q8LSN3) in the roots of *Pongamia* under salt stress conditions. The abundance of protein species associated with hormone metabolism, namely, carotenoid 9, 10 (9′, 10′) cleavage dioxygenase 1 (CCD1_Q8LP17), G2OX1_Q9SQ80, G2OX2_Q9XHM5, abscisate-β-glucosyltransferase (AOG_Q8W3P8), calmodulin 2 (CALM_P62163), guanine nucleotide-binding protein subunit-β-like protein (GBLP_Q39836), and rac-like GTP-binding protein 1 (RAC_O04369) was low in roots of the salt-treated plants. Protein species such as AUX22C_O24541, AUX22D_O24542, LAX2_Q9FEL7, LAX3_Q9FEL6, PCS3_Q2QKL5, AMO_P49252, ras-related protein rab11A (RB11A_Q40191), rab11C (Rab11C_Q40193), MMK1_Q07176, purple acid phosphatase (PPAF_Q09131), and rac-like GTP-binding protein (RHO1_Q35638) were abundant only in the salt-treated plants.

### Protein Species of Antioxidant Enzymes and Electron Transport Chain Components

CATA4_O48561 showed high abundance in the salt-treated roots of *Pongamia* (Supplementary Figure 11). Antioxidant enzymes such as MDAR (MDAR_Q40977) and L-ascorbate peroxidase (APX1_P48534) were increased only in roots of the salt-treated plants. Additionally, the abundance of protein species related to electron transport chain such as NAD(P)H-quinone oxidoreductase (NDHH) subunit H (chlNADHH_Q9BBN8), ATP synthase subunit-α (mitochondrial origin) (mtATPA_Q01915), chlATPA_A4GGB2, and ATPE_Q2PMU9 were high with respect to their controls. Protein species, namely, CYB_P05718, CYB6_Q2PMQ5, chlNU4_Q2PMN2/A4GGE6, chlNU5_Q2PMM9/P15958, and chlNDHH_A4GGF2 were abundant only in roots of the salt-treated plants.

### Other DEPs

Trypsin protease inhibitors (ITs) such as IT2_P25700 and ITRA_P01070 showed significant increase, while ITRB_P01071 was decreased in roots of the salt-treated plants (Supplementary Table 3). In addition, proteinase inhibitors, namely, bowman-birk type proteinase inhibitor A-II (IBB1_P85172), IBB3_P01057, kunitz-type trypsin inhibitor (KTI2_P25273), cysteine proteinase inhibitor (CYTI_Q06445), peptidyl-prolyl *cis*–*trans* isomerase 1 (CYP1_Q8W171), and ATP-dependent Clp protease proteolytic subunit (CLPP_Q9BBQ9) were abundant only in roots of the salt-treated plants. Protein species belonging to the monooxygenase family, such as cytochrome P450 71D10 (C71DA_O48923), C71DB_O22307, cytochrome P450 82A1 (C82A1_Q43068), and cytochrome P450 94A1 (C94A1_O81117), showed high abundance, while cytochrome P450 71D9 (C71D9_O81971) was relatively low in roots of the salt-treated plants (Supplementary Figure 12). Other protein species, namely, calnexin homolog (CALX_O82709), maturase K (MATK_Q6PSC6/Q9TKS4), proliferating cell nuclear antigen (PCNA_O82134), vacuolar-sorting receptor 1 (VSR1_P93484), low-affinity sulfate transporter 3 (SUT3_P53393), abrin-c OS (ABRC_P28590), tubulin alpha 1 chain (TBA1_P46259), cytolytic protein enterolobin (ENT_P81007), and glutamine synthetase (GS) nodule isozyme (GLNA3_Q43785) were significantly increased under salt stress (Supplementary Figure 13). Conversely, we also noticed that the fold values of protein species of MATK showed slight increase or were unchanged during salt stress. Translation protein species such as 60S ribosomal protein L24 (RL24_O65743) showed high abundance, while 50S ribosomal protein L16 (chlRK16_Q8MCA4/Q2PMP8), eukaryotic translation initiation factor 5 (IF5_P48724), RK2A_P18663, RK2B_Q2PMM3, and RK2_Q8LVH2/A4GGF8/Q9B1H9 showed low abundance in roots of the salt-treated plants. Eukaryotic translation initiation factor 1A (IF1A_P56331), 60S acidic ribosomal protein P0 (RLA0_P50345), RL27_Q05462, ribosomal protein S10 (mitochondria origin) (RT10_P51428), RK20_Q9BBR0, RK9_P11894, and RK24_P11893 were abundant only in roots of the salt-treated plants. Similarly, carbohydrate-binding protein species such as Flt3 receptor-interacting lectin (FRIL_Q9ZTA9), bark agglutinin I polypeptide B1 (LCB1_Q41159), lectin-4 (LEC4_P24146), agglutinin-1 (LEC1_Q39528), LEC2_P29257, LCB2_Q42372, and LCB3_Q41160 were high in roots of the salt-treated plants.

### Protein Network Studies

To elucidate protein–protein interaction networks, we employed Pearson’s correlation coefficient (*r*) on selected fold values ≤0.5 (LAPs) and ≥2.0 (HAPs) data points. The correlation network was generated at the *P* < 0.99 significance threshold and *P* ≤ 0.05 statistical significance. We also analyzed the network as undirected with combined paired edges.

**FIGURE 7:**
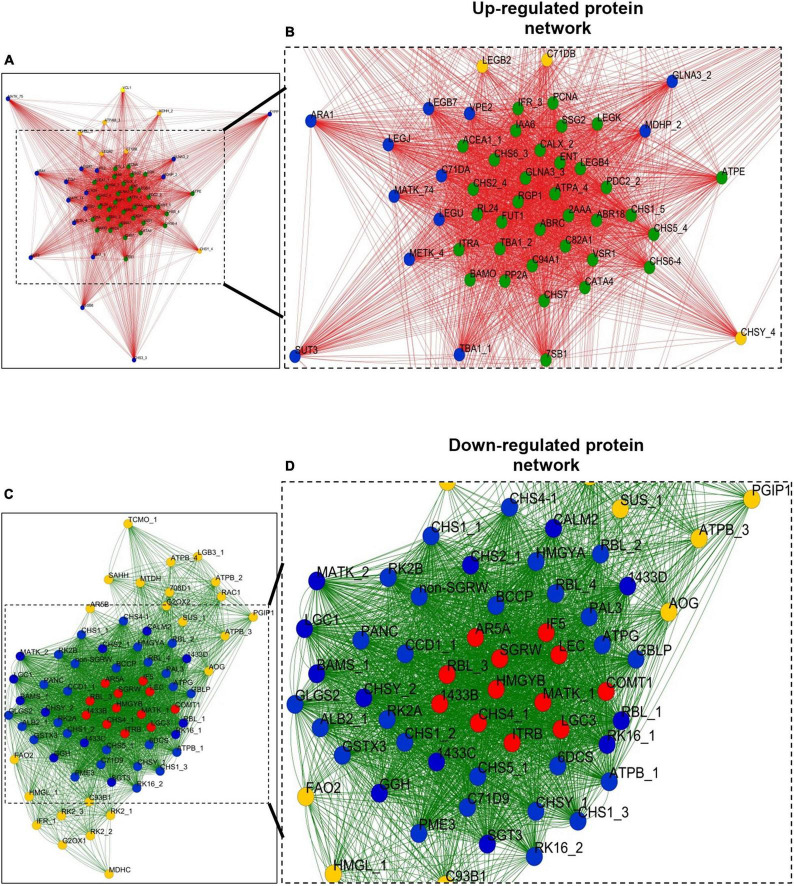
Correlation networks of abundant proteins. **(A)** Correlation network of HAPs based on Pearson correlation coefficient with probability threshold *P* < 0.0001. Each node was represented with a different color based on the degree of interaction. The order of interaction was green color node > blue color node > yellow color node. **(B)** Correlation network of 53 protein species in a cluster; box was drawn to represent zoom-in of the most highly interconnected core of proteins. **(C)** Correlation network of LAPs based on Pearson correlation coefficient with probability threshold *P* < 0.0001. Each node was represented with a different color based on the degree of interaction. The order of interaction was red color node > blue color node > yellow color node. **(D)** Correlation network of 72 protein species in a cluster; box was drawn to represent zoom-in of the most highly interconnected core of proteins. For protein abbreviations, see Supplementary Table 1.

For HAP correlation network, each data point was considered as a node, and a total of 58 nodes (each node represents single protein species) formed 1,350 edges (neighboring interactions); on average, each node shared 47 edges with neighboring nodes (clustering coefficient of 0.92) (Figures 7A,B). To simplify the correlation network, the nodes were represented in circles with three colors (yellow, blue, and green) based on the degree of interactions. The number of interactions ranges from ∼15 to ∼55, yellow circles range from ∼15 to ∼40, blue circles range from ∼45 to ∼50, and green ranges from ∼50 to ∼55. The green nodes consist of a dense network containing an average of ∼50 interaction edges (closeness centrality < 0.9) with neighboring nodes. Interestingly, the green nodes belong to protein species related to secondary metabolism (CHS1_P51083, CHS2_P30074/Q01287, CHS5_P51087, CHS6_P51088, CHS6-4_P51079, CHS7_P30081, and IFR_P52575), carbohydrate metabolism (ACEA1_P45456 and SSG2_Q43093), cell wall synthesis (FUT1_Q9M5Q1, RGP1_O04300, and PDC2_P51851), hormone metabolism (ABR18_Q06930 and IAA6_P49680), ETC protein species (ATPE_Q2PMU9 and chlATPA_A4GGB2), seed storage protein species (7SB1_P13917, LEGB4_P05190, and LEGK_P05693), CATA4_O48561, CALX_O82709, PP2A_Q06009/P36875, GLNA3_Q43785/P00965, C94A1_O81117, VSR1_P93484, C82A1_Q43068, TBA1_P46259, ITRA_P01070, BAMO_B5BSX1, RL24_O65743, ENT_P81007, PCNA_O82134, and ABRC_P28590. Similarly, 72 nodes formed a dense interaction network with 1,414 edges for a down-regulated correlation network. Each node shared its interaction network on an average of 39 neighboring nodes (clustering coefficient of 0.84) (Figures 7C,D). To simplify the correlation matrix, each node was represented with a specific color (yellow, blue, or red) based on the degree of interaction (Figures 7C,D). The number of interactions ranges from ∼10 to ∼55; yellow circles range from ∼10 to ∼40, blue circles range from ∼40 to ∼50, and red ranges from ∼50 to ∼55. Furthermore, the red and blue nodes formed a dense network containing an average of ∼50 interaction edges (closeness centrality < 0.75) with neighboring nodes, which correspond to protein species related to secondary metabolism (CHS2, CHS4, CHSY, COMT, and LEC), saponin metabolism (BAMS and SGT3), defense response proteins (1433B, C, and D), ITRB, RBL (isoform), LGC1, GGH, MATK, RK16, CALM2, SGRW, HMGYB, LGC3, IF5, and AR5A.

## Discussion

### Salinity-Induced Changes in Gas Exchange Parameters and Enhanced Photo-Protective Components of Photosystem II Improves Leaf Photosynthesis in *Pongamia* Under High Salinity Stress

Our previous study has shown that stable leaf physiological responses are crucial in salt tolerance in *Pongamia* (Marriboina and Attipalli, 2020a). *Pongamia* did not display salt-induced morphological symptoms 30 DAS, which clearly showed that there are strong adaptive mechanisms to withstand such as high salt concentrations. Decrease in *g*_*s*_ with increase in salinity treatment time in the salt-treated plants suggests that *Pongamia* might operate a water-conserving mechanism to withstand salt-induced limited water conditions (Marriboina et al., 2017). Additionally, decreased *E* and *A*_*sat*_ would help to maintain limited photosynthesis by decreasing water evaporation and net CO_2_ fixation, which might extend plant survivability until favorable conditions appear (Acosta-Motos et al., 2017). Although *E* and *A*_*sat*_ were significantly decreased in the salt-treated plants, their ratio (WUE_i_) gradually increased with treatment time, which helps to maintain the marginal photosynthetic activity under salt stress conditions (Khataar et al., 2018). Stable and constant low *A*_*sat*_/C_i_ and *A*_*sat*_/Q suggest that *Pongamia* might be operating strong adaptive mechanisms to maintain stable photosynthesis under extreme salt stress conditions. Moreover, several plant species maintain low photosynthetic rates to balance CO_2_ utilization and water usage under adverse limited water conditions (Brito et al., 2014; Sekhar et al., 2015; Miranda et al., 2016).

The excited light energy distributed between the LHCII complex and PSII units represents the connectivity or grouping between these components denoted by L-band, which was calculated through kinetic difference between O and J phases. Notably, the characteristic negative bell-shaped L-band suggests the connectivity or grouping between LHCII and PSII units to enhance the effective utilization of energy throughout PSII units under salt stress (Gururani et al., 2015). Increased Fo reflects the accumulation of inactive reaction centers in the PSII complex. Furthermore, changes in Mo, Fm/Fo, and Vj, and reduced plastoquinone pool size also reflect the accumulation of inactive reaction centers in PSII, which might cause the fractional reduction in Q_*A*_ to Q_*A*_^–^ or low energy transfer from LHCII to PSII (Guha and Reddy, 2014; Xu et al., 2020). The prominent K-band at 300 μs suggests well preserved structural and functional integrity of OEC or limitation at donor side and acceptor side of PSII, as well as the availability of non-water electron donors at the acceptor side of PSII. The appearance of a negative bell-shaped K-band might reveal the possible recruitment of non-water electron donors such as osmolytes at the acceptor side of PSII under water-limited environmental regimes (Sengupta et al., 2013). *Pongamia* also accumulated several osmolytes such as mannitol, pinitol, myo-inositol, as well as proline, in leaves under salt stress (Marriboina et al., 2021). The O-I phase denotes the state of electron transfer from Q_*A*_^–^ to Q_*B*_. The absence of a negative ΔV_OI_ band and no significant changes in Vi suggest that *Pongamia* could maintain an active PQ pool under salt-induced water-deprived conditions. However, limited electron transport from PSII to Q_*A*_ and constant maintenance of active PQ pool under high saline conditions are quite arguable. The involvement of chloroplast oxidases in higher plants and algae could maintain or recycle the PQ pool under stressful conditions (Bennoun, 2002). Step I-P showed transfer of electrons from reduced end acceptors of PSII (plastoquinol) to end acceptors of PSI [ferredoxin-NADP^+^ reductase (FNR)]. The amplitude of V_IP_ followed a hyperbolic pattern, which can be fitted into the Michaelis–Menten equation to calculate V_IP_ = 0.5 (the half-maximal time required to estimate the rate of reduction of electron acceptors of PSI) (Adamski et al., 2011). The unaltered or slightly changed amplitude of ΔV_IP_ may result in high reduction rate of PSI end acceptor (Guha and Reddy, 2014).

We determined the energy pipeline leaf model of phenomenological fluxes (per cross-section, CS) and JIP parameters to know further insights into PSII photochemical efficiency. Additionally, the unchanged TRo/RC and TRo/CSm indicate continuous supply of trapping energy, which could impose a deleterious effect on PSII under saline conditions (Guidi et al., 2016). To counteract these deleterious effects, increased ABS/RC, DIo/CSm, DIo/RC, and PHI(Do) might balance the absorbed and trapped excess energy by converting it into thermal dissipation energy (Kalaji et al., 2016). We also observed a significant decrease in electron transport components such as PHI(Eo), ETo/RC, and ETo/CSm in the salt-treated plants. The decrease in ETo leads to decrease in electron transport to the terminal electron acceptor end of PSI (REo/ABS) (Baghbani et al., 2019). Furthermore, we also noticed that the overall electron transport of intersystem electron carriers to the terminal electron acceptor end of PSI (REo/ETo) remained constant, suggesting increased dissipation of absorption fluxes and trapped energy, as well as recruitment of non-water electron donors at the donor side of PSII to maintain the reducing terminal electron acceptor end of PSI (Yan et al., 2013). The performance indices of photochemical [PHIo/(1-PHIo)] and non-light-dependent [PSIo/(1-PSIo)] reactions decreased significantly in the salt-treated plants, which was further confirmed by decreased responses of Kp, net photosynthetic rate, *A*_*sat*_/Q, and *A*_*sat*_/C_i_ curves. However, the unchanged non-photochemical rate constant (Kn), *A*_*sat*_/C_i_, and *A*_*sat*_/Q suggest that *Pongamia* operates an effective photoinhibition mechanism to survive under extreme saline conditions. Our results also elucidate that the efficiency of PSII is largely maintained by recruiting non-water electron donors and promoting enzyme-dependent redox PQ pool balance and increasing thermal dissipation of energy. Thus, gas measurements and Chl *a* fluorescence studies demonstrate that *Pongamia* has recruited thermal dissipation components and non-water electron donors to protect the photosystems to drive effective photosynthesis under salt-induced limited water conditions.

### Alternations in Root Proteome and Phenylpropanoid Pathway-Related Protein Species Conferred Salt Tolerance in *Pongamia*

A whole root of Pongamia was taken for label-free shot-gun proteome analysis. A total of 1,062 protein species were identified in the roots of *Pongamia* by label-free shot-gun proteome analysis. We also used computational platforms (R-program and Cytoscape) and performed GO-based analysis to explore more knowledge on global proteome changes in *Pongamia* as well as to further simplify the complex data into visual graphical and network forms. Most of the protein species were unchanged (∼82.5%) in response to salt stress, suggesting the dynamic nature of root cell proteome, which is essentially important to preserve cellular processes toward changing environmental cues.

The *Pongamia* roots expressed numerous protein species related to the phenylpropanoid pathway, including CHS protein species showing high abundance in root response to salt stress. CHS is known to catalyze the synthesis of naringenin chalcone, which is a branch point of flavanones, flavonols, and anthocyanins synthesis (Zhang et al., 2020). The high abundance of CHS species might help in the biosynthesis of flavonoids to scavenge the ROS generated during salt stress (Chen et al., 2019). In the network analysis, CHS1 was found to be a network node containing the highest number of interactions with other protein species. The abundance of CADH, COMT, THS2, and CAMT also indicates the importance of the phenylpropanoid pathway in the salt tolerance of *Pongamia*. Furthermore, the high-fold increase in glycosyltransferases (FG3H and FG2KO) may induce the accumulation of flavonoids such as kaempferol-3-*O*-sophoroside and kaempferol-3-*O*-rutinoside, which improve the defense mechanism of plants under stressful conditions (Martinez et al., 2016; Li et al., 2017). Thus, the high abundance of protein species related to secondary metabolism could help *Pongamia* with high antioxidant activity to defend from ROS damage and promote root growth during high salinity stress.

### Induction of Protein Species Related to Glyoxylate Cycle, Carbohydrate Metabolism, and Cell Wall Carbohydrate Synthesis Provides Resilience to Root Under Salt Stress Conditions

The high abundance of cytoplasmic GS and ASPG induce the production of glutamate and aspartate, respectively, which might increase the availability of nitrogen for newly synthesized proteins, and increase levels of SPE1 and concomitant decrease in the abundance of SPD1 and SPD2 might increase the availability of putrescine either in the free or conjugated form in the cell. Perennial plants are known to use the conjugated putrescine to stabilize membrane potential and cellular pH balance under saline conditions, albeit the free form of putrescine is toxic to the cell (Yamamoto et al., 2017). The abundance of INV indicates the conversion of sucrose to glucose and fructose, substrates for glycolysis and HMP shunt. The rise in F16P2 might signify the accumulation of fructose-6-phosphate, a substrate for numerous metabolic pathways, such as sucrose and osmolyte biosynthesis (Stein and Granot, 2019). We have previously reported increased levels of sucrose, glucose, and fructose in roots of *Pongamia* under salt stress (Marriboina et al., 2021). The steady-state levels of protein species such as SUS/SUS2, SPSA, PMM, and MTDH might help the plant in synthesizing phosphorylated sugars such as sucrose-6p, mannose-1p, and mannitol to retain cellular osmotic potential (Dong et al., 2018). The interaction between ACEA1 and PDC2 diverts the carbohydrate pool to anaerobic respiration to replenish reducing energy equivalents rather than channelizing the carbohydrate pool toward the TCA cycle (Yuenyong et al., 2019). Furthermore, the abundance of pyruvate-metabolizing protein species such as PDC2, ADHX, and AL7A1 suggest the involvement of an anaerobic process in the roots of *Pongamia* during salt stress, which might supply reduced energy equivalents, to quickly restore cellular energy needs under salt stress conditions (Luo et al., 2017). Enhanced synthesis of antioxidant enzymes such as APX1, CATA4, and MDAR may reduce the production of ROS inside the cell under salt stress (Li et al., 2015; Sofo et al., 2015). The high abundance of both PP2A and FyPP1 expressions, as well as positive interaction between PP2A and FyPP1, might lead to the formation of PP2A-FyPP1 complex to positively regulate root growth under salt stress (Dai et al., 2013). Increased PP2Ac and PP2Ar might regulate the balancing of amino acids by controlling both SAHH and GLN1 (Lillo et al., 2014; Marriboina et al., 2021). Our shotgun proteome results suggest that *Pongamia* induced different proteins associated with secondary metabolism to provide defense against ROS and maintain cell wall turgidity under salinity stress. The induction of proteins related to the glyoxylate cycle and carbohydrate metabolism could replenish the quick energy resources to maintain the vital metabolic activity of roots under salt stress. In this study, we observed that *Pongamia* induced several root proteins related to phytohormones, small GTP-binding proteins, and other proteins, which are proved to be involved active long-distance signaling from root to shoot. However, the combination of both molecular biology and physiological tools is required to understand the role of these proteins in *Pongamia* root–shoot communication under salt stress. This study investigated the changes in root proteome that are crucial to maintain sustainable leaf physiology, which can be further evidenced by advanced molecular tools such as gene silencing and gene heterologous expression studies.

## Conclusion

This study explicitly focused on global root proteome changes and determined the alternations in *P. pinnata* leaf photosynthesis under salt stress conditions. *Pongamia* relies on protein species related to several critical regulatory pathways to maintain its growth under salt stress. *Pongamia* also triggered protein species belonging to various metabolic pathways including secondary metabolism, carbohydrate metabolism, anaerobic respiration, and antioxidant metabolism, which may help in adjusting the root cells toward balancing the cell wall turgidity and ROS cellular energy redox homeostasis under saline environment conditions. The stable marginal leaf photosynthesis in response to salt-induced limited water conditions suggests a strong communication between root and shoot in *Pongamia*. This leaf physiology and whole root proteome study highlights the key mechanisms of high salt tolerance in Pongamia, which can be highly crucial for further research to develop Pongamia in marginal land cultivation.

## Data Availability Statement

The original contributions presented in the study are publicly available. This data can be found here: The mass spectrometry proteomic data have been deposited in ProteomeXchange with the accession code: PXD021558.

## Author Contributions

SM and KS designed the research and analyzed the data. SM performed the research. SM, RS, and AR wrote the manuscript. RS and AR supervised the research. All authors contributed to the article and approved the submitted version.

## Conflict of Interest

The authors declare that the research was conducted in the absence of any commercial or financial relationships that could be construed as a potential conflict of interest.

## Publisher’s Note

All claims expressed in this article are solely those of the authors and do not necessarily represent those of their affiliated organizations, or those of the publisher, the editors and the reviewers. Any product that may be evaluated in this article, or claim that may be made by its manufacturer, is not guaranteed or endorsed by the publisher.
