# Supplementary material for: Physiological, Biochemical, and Root Proteome Networks Revealed New Insights Into Salt Tolerance Mechanisms in Pongamia pinnata (L.) Pierre
Source: Front Plant Sci. 2022 Jan 24;12:771992. doi: 10.3389/fpls.2021.771992 (PMC8818674; doi:10.3389/fpls.2021.771992)
Supplement: Supplementary file 1 [file Data_Sheet_1.docx]

**Physiological, biochemical and root proteome networks revealed new insights into salt tolerance mechanisms in *Pongamia pinnata* (L.) pierre**

**Authors:** Sureshbabu Marriboina, Kalva Madhana Sekhar, Rajagopal Subramanyam^*^, Attipalli Ramachandra Reddy^*^

Department of Plant Sciences, School of Life Sciences, University of Hyderabad, Hyderabad-500046, India

***For Correspondence:**

**E-mail:** [srgsl@uohyd.ac.in](about:blank); [arrsl@uohyd.ernet.in](mailto:arrsl@uohyd.ernet.in)

**SUPPLEMENTARY FIGURES AND TABLES**

Figure S1. Experimental design carried out to analyse whole proteome analysis of 500 mM NaCl treated *P.pinnata* roots.

Figure S2. Energy pipeline leaf model of phenomenological fluxes in *Pongamia* grown under 300 and 500 mM NaCl concentration for 15 and 30 DAS.

Figure S3. The polyphasic chlorophyll *a* transients for O-K, O-J, O-I and I-P phases in leaves of *P. pinnata* under salt-treated conditions. (A) double normalized variable chl *a* between phase O (50 *μ*s) and K (300 *μ*s) [V_OK_ = (F_t_-Fo)/(F_K_-Fo)]; (B) double normalized variable chl *a* between phase O (50 *μ*s) and J (2 ms) [V_OJ_ = (F_t_-Fo)/(F_J_-Fo)]; (C) double normalized variable chl *a* between phase O (50 *μ*s) and I (30 ms) (V_OI_ < 1) [V_OI_ = (F_t_-Fo)/(F_I_-Fo)]; (D) kinetic difference between control and treated values of ΔV_OI_ with respective controls represented black and grey circles; (E) double normalized variable chl *a* between phase I (30 *μ*s) and P (180 ms) shift in the Km values (time required to attain V_IP_ = 0.5) [V_IP_ = (F_t_-F_I_)/(F_M_-F_I_)]; (F) kinetic difference between control and treated values of ΔV_IP_ with respective controls represented black and grey circles. Data are mean ± SD (*n* = 4).

Figure S4. (A) Percentage homology of *Pongamia* protein species with other related legume species and (B) percentage of abundant proteins.


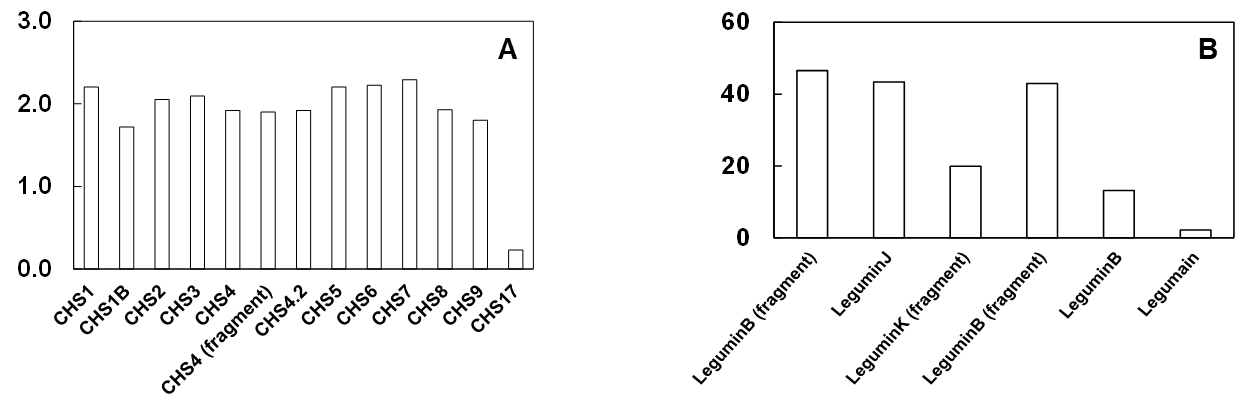


Figure S5. (A) Relative fold expression of chalcone synthase proteins and (B) isoforms of seed storage proteins. For protein abbreviations see in Table S3 and S4.

Figure S6. Relative fold expression of DEPs belongs to various metabolic pathways. Colour key represents fold expression and number indicates number of identified peptides; red colour: up-regulated proteins, green colour: down-regulated proteins, blue colour: unchanged proteins, yellow colour: control specific proteins, and violet colour: treated specific proteins. For protein abbreviations see in Table S3 and S4.

Figure S7. Schematic representation of carbohydrate metabolic pathway. The relative expression of protein species related to carbohydrate metabolism. Colour key represents fold expression and number indicates number of identified peptides; red colour: high abundant protein species, green colour: low abundant protein species, blue colour: unchanged protein species, yellow colour: control specific protein species, and violet colour: treated specific protein species. G6P: glucose-6-phosphatase, MTDH: mannitol dehydrogenase. For protein abbreviations see in Table S3 and S4.

Figure S8. Schematic representation of amino acid metabolic pathway. The relative expression of proteins related to amino acid metabolism. Colour key represents fold expression and number indicates number of identified peptides; red colour: up-regulated proteins, green colour: down-regulated proteins, blue colour: unchanged proteins, yellow colour: control specific proteins, and violet colour: treated specific proteins. For protein abbreviations see in Table S3 and S4.

Figure S9. Relative fold expression of DEPs belongs to various metabolic pathways. Colour key represents fold expression and number indicates number of identified peptides; red colour: up-regulated proteins, green colour: down-regulated proteins, blue colour: unchanged proteins, yellow colour: control specific proteins, and violet colour: treated specific proteins. For protein abbreviations see in Table S3 and S4.


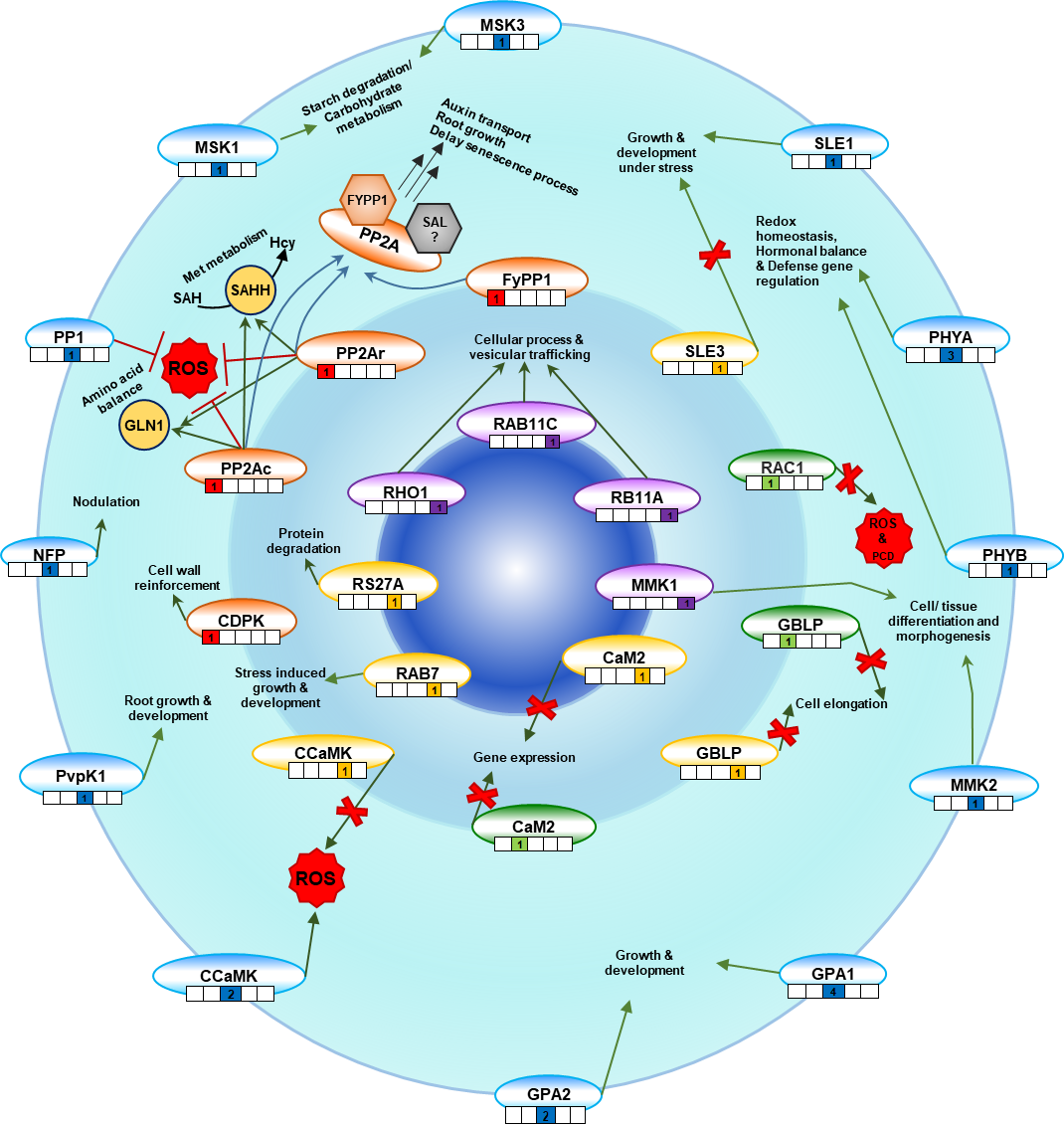


Figure S10. Proposed model for signalling transcription factors. Colour key represents fold expression and number indicates number of identified peptides; red colour: up-regulated proteins, green colour: down-regulated proteins, blue colour: unchanged proteins, yellow colour: control specific proteins, and violet colour: treated specific proteins. For protein abbreviations see in Table S3 and S4.


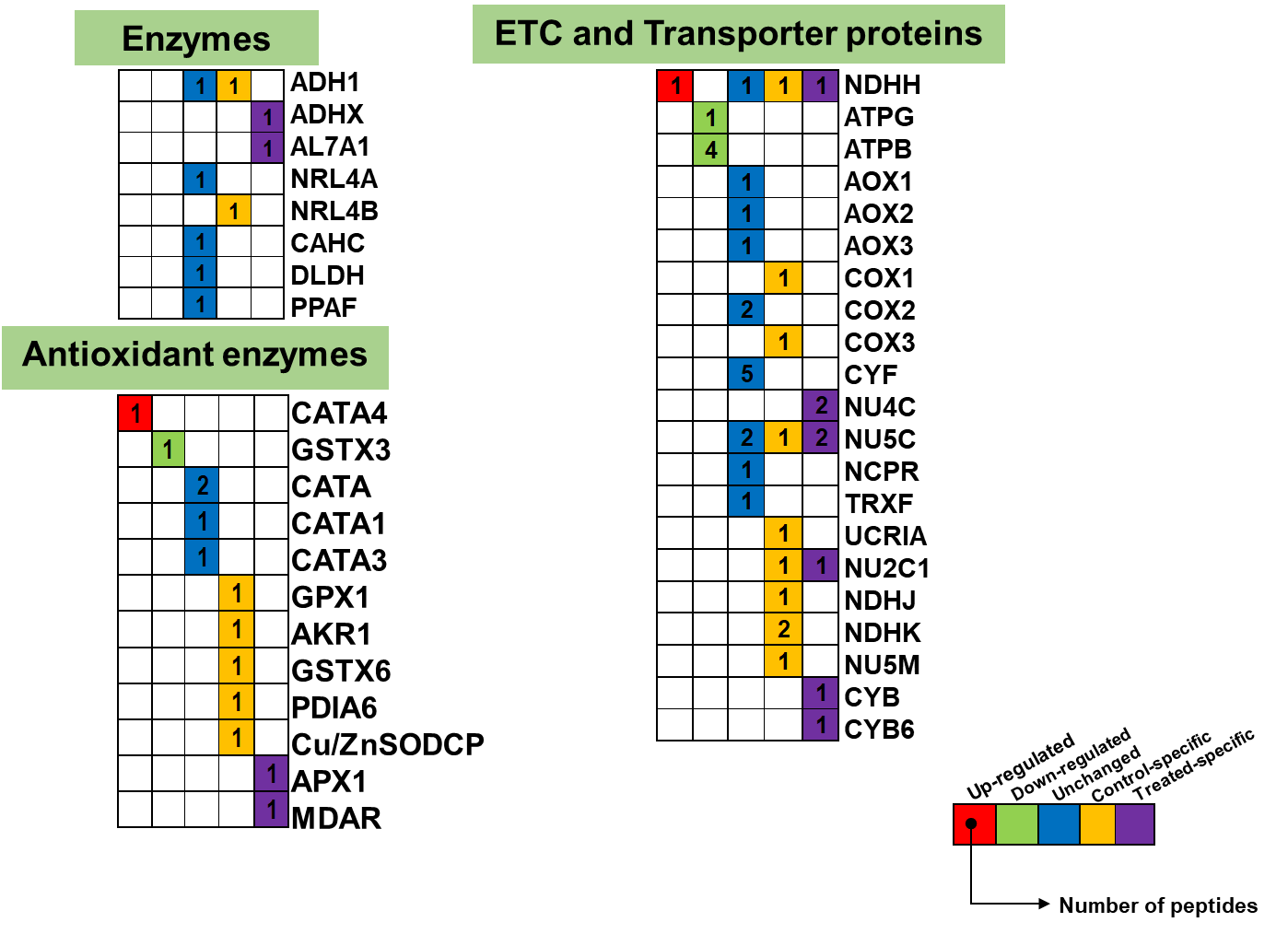


Figure S11. Relative fold expression of DEPs belongs to various metabolic pathways. Colour key represents fold expression and number indicates number of identified peptides; red colour: up-regulated proteins, green colour: down-regulated proteins, blue colour: unchanged proteins, yellow colour: control specific proteins, and violet colour: treated specific proteins. For protein abbreviations see in Table S3 and S4.


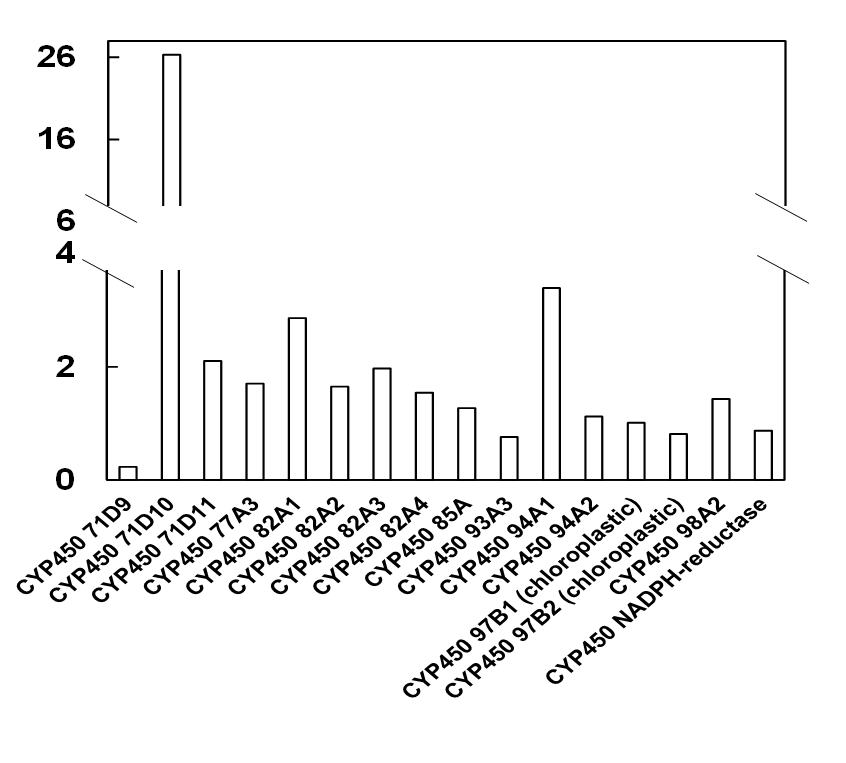


Figure S12. Relative fold expression of CYP450 family proteins.

Figure S13. Relative fold expression of DEPs. Colour key represents fold expression and number indicates number of identified peptides; red colour: up-regulated proteins, green colour: down-regulated proteins, blue colour: unchanged proteins, yellow colour: control specific proteins, and violet colour: treated specific proteins. For protein abbreviations see in Table S3 and S4.

Table S1. Photosynthetic and biochemical parameters of control and salt-treated leaves of *Pongamia* at 15 and 30 DAS.

|  |  |  |  |  |  |  |  |
| --- | --- | --- | --- | --- | --- | --- | --- |
| Parameters | Control |  | 300mM NaCl | |  | 500mM NaCl | |
|  |  |  | 15DAS | 30DAS |  | 15DAS | 30DAS |
|  |  |  | (mg/g) | (mg/g) |  | (mg/g) | (mgl/g) |
| Chl *a* | 2.20(±0.04) |  | 2.26(±0.02) | 2.00 (±0.01) |  | 2.27(±0.02) | 2.05(±0.02) |
| Chl *b* | 1.07(±0.06) |  | 1.06(±0.07) | 1.03(±0.01) |  | 1.05(±0.05) | 1.01(±0.02) |
| Total chl | 3.91(±0.04) |  | 3.92(±0.08) | 4.05(±0.04) |  | 3.96(±0.02) | 4.21(±0.02) |
| Chl *a*/*b* | 2.06(±0.60) |  | 2.13(±0.29) | 2.06(±0.35) |  | 2.11(±0.41) | 2.03(±0.18) |
| Total carotenoids | 0.73(±0.02) |  | 0.64(±0.01) | 0.74(±0.01) |  | 0.61(±0.05) | 0.72(±0.02) |
| Proline (FW) | 0.240(±0.01) |  | 0.24(±0.09) | 0.72(±0.040) |  | 0.30(±0.02) | 0.76(±0.01) |
| Total soluble | 16.064(±0.04) |  | 16.00(±0.10) | 11.59(±0.12) |  | 15.85(±0.06) | 9.15(±0.04) |
| sugars (DW) |  |  |  |  |  |  |  |
|  |  |  |  |  |  |  |  |

| Parameter | Control | 15DAS | |  | 30DAS | |
| --- | --- | --- | --- | --- | --- | --- |
|  |  | 300mM NaCl | 500mM NaCl |  | 300mM NaCl | 500mM NaCl |
| Tfm | 190 (±0.74) | 280 (±1.47) | 250 (±1.32) |  | 190 (±1.00) | 160 (±0.84) |
| Area | 22184 (±1.41) | 27073 (±1.22) | 27768 (±1.25) |  | 13462 (±0.61) | 9271 (±0.42) |
| Fo | 531 (±1.41) | 598 (±1.13) | 604 (±1.14) |  | 668 (±1.26) | 671 (±1.26) |
| Fm | 2656 (±2.1) | 2962 (±1.12) | 2987 (±1.12) |  | 2679 (±1.00) | 2618 (±0.99) |
| Fo/Fm | 0.200 (±0.1) | 0.201 (±0.02) | 0.202 (±0..01) |  | 0.249 (±0.25) | 0.256 (±0.28) |
| Sm/Tfm | 0.060 (±0.07) | 0.045 (±0.075) | 0.051 (±0.085) |  | 0.039 (±0.066) | 0.034 (±0.056) |
| PHIo/(1-PHIo) | 2.757 (±1.89) | 2.716 (±0.99) | 2.697 (±0.98) |  | 2.051 (±0.74) | 1.899 (±0.69) |
| PSIo/(1-PSIo) | 2.757 (± 2.1) | 0.524 (±0.19) | 0.526 (±0.19) |  | 0.287 (±0.48) | 0.202 (±0.34) |
| D.F. | 1.666 (±0.52) | 1.548 (±0.93) | 1.539 (±0.92) |  | 0.530 (±0.32) | 0.031 (±0.019) |

Table S2. JIP test parameters of control salt-treated leaves of *Pongamia* at 15 and 30 DAS.

Table S3. List of all proteins expressed in roots of 500 mM NaCl treated *Pongamia*.

| **S.No** | **Accession** | **Abbreviation** | **Description** | **Score** | **Fold change** | **P values** | **GO**  **description** |
| --- | --- | --- | --- | --- | --- | --- | --- |
| **Down-regulated proteins** | | | | | | | |
| 1 | Q41116 | AR5B | Arcelin-5B | 118.8 | 0.00 | 0.03 | Seed storage protein |
| 2 | Q42460 | AR5A | Arcelin-5A | 77.9 | 0.00 | 0.04 | Seed storage protein |
| 3 | Q8MCA4 | RK16 | 50S ribosomal protein L16, chloroplastic | 53.0 | 0.03 | 0.04 | Translation |
| 4 | P28002 | COMT1 | Caffeic acid 3-O-methyltransferase | 55.5 | 0.04 | 0.01 | Secondary metabolism |
| 5 | Q5D1B9 | MATK | Maturase K | 16.9 | 0.05 | 0.01 | mRNA processing |
| 6 | P48724 | IF5 | Eukaryotic translation initiation factor 5 | 57.6 | 0.05 | 0.02 | Translation |
| 7 | Q85XY8 | MATK | Maturase K | 29.1 | 0.05 | 0.05 | mRNA processing |
| 8 | P02237 | LGC3 | Leghemoglobin C3 | 8.3 | 0.09 | 0.02 | Nodulation |
| 9 | O24035 | PANC | Pantoate-β-alanine ligase | 28.4 | 0.16 | 0.04 | Cofactor biosynthesis |
| 10 | D4AEP7 | ALB2 | Albumin-2 | 115.3 | 0.16 | 0.04 | Seed storage protein |
| 11 | Q2PMP8 | RK16 | 50S ribosomal protein L16, chloroplastic | 53.0 | 0.16 | 0.03 | Translation |
| 12 | P46417 | GSTX3 | Glutathione S-transferase 3 | 26.8 | 0.18 | 0.01 | Redox homeostasis |
| 13 | P26585 | HMGL | HMG1/2-like protein | 73.7 | 0.18 | 0.02 | DNA binding |
| 14 | Q8LP17 | CCD1 | Carotenoid 9,10(9',10')-cleavage dioxygenase 1 | 110.2 | 0.19 | 0.05 | Hormone metabolism |
| 15 | O23884 | CHS5 | Chalcone synthase 5 | 34.8 | 0.21 | 0.02 | Secondary metabolism |
| 16 | Q10370 | HMGYB | HMG-Y-related protein B (Fragment) | 377.2 | 0.21 | 0.03 | DNA binding |
| 17 | O23882 | CHS4 | Chalcone synthase 4 | 34.8 | 0.21 | 0.01 | Secondary metabolism |
| 18 | P93164 | GGH | γ-glutamyl hydrolase | 71.9 | 0.21 | 0.02 | Amino acid metabolism |
| 19 | P02235 | LGC1 | Leghemoglobin C1 | 8.3 | 0.22 | 0.01 | Nodulation |
| 20 | P51084 | CHS2 | Chalcone synthase 2 | 34.8 | 0.22 | 0.05 | Secondary metabolism |
| 21 | P02871 | LEC | Favin | 213.3 | 0.22 | 0.03 | Secondary metabolism |
| 22 | P51077 | CHS4-1 | Chalcone synthase 4-1 | 34.8 | 0.23 | 0.04 | Secondary metabolism |
| 23 | P49440 | CHSY | Chalcone synthase 17 | 28.3 | 0.23 | 0.01 | Secondary metabolism |
| 24 | O81971 | C71D9 | Cytochrome P450 71D9 | 22.5 | 0.23 | 0.01 | Monooxygenase |
| 25 | Q9SML4 | CHS1 | Chalcone synthase 1 | 60.6 | 0.24 | 0.03 | Secondary metabolism |
| 26 | P18663 | RK2A | 50S ribosomal protein L2-A | 74.3 | 0.24 | 0.01 | Translation |
| 27 | P30073 | CHS1 | Chalcone synthase 1 | 34.8 | 0.24 | 0.05 | Secondary metabolism |
| 28 | Q00423 | HMGYA | HMG-Y-related protein A | 365.7 | 0.27 | 0.02 | DNA binding |
| 29 | P52417 | GLGS2 | Glucose-1-phosphate adenylyltransferase  small subunit 2 | 51.7 | 0.27 | 0.05 | Carbohydrate metabolism |
| 30 | Q9B1H9 | RK2 | 50S ribosomal protein L2 | 74.3 | 0.27 | 0.01 | Translation |
| 31 | A4GGF8 | RK2 | 50S ribosomal protein L2 | 135.7 | 0.27 | 0.02 | Translation |
| 32 | Q8LVH2 | RK2 | 50S ribosomal protein L2 | 143.8 | 0.28 | 0.03 | Translation |
| 33 | O48905 | MDHC | Malate dehydrogenase, cytoplasmic | 45.8 | 0.29 | 0.03 | Carbohydrate metabolism |
| 34 | Q01286 | CHS1 | Chalcone synthase 1 | 34.8 | 0.30 | 0.05 | Secondary metabolism |
| 35 | P01071 | ITRB | Trypsin inhibitor B | 97.0 | 0.30 | 0.05 | Protease inhibitor |
| 36 | Q96453 | 1433D | 14-3-3-like protein D | 62.2 | 0.31 | 0.01 | Defense response |
| 37 | P51089 | CHSY | Chalcone synthase | 28.3 | 0.31 | 0.01 | Secondary metabolism |
| 38 | O62964 | RBL | Ribulose bisphosphate carboxylase large chain | 6.8 | 0.32 | 0.04 | Carbohydrate metabolism |
| 39 | A7VLV2 | non-SGRW | Non-functional protein STAY-GREEN | 126.5 | 0.33 | 0.03 | Photosynthesis |
| 40 | Q2PMV0 | ATPB | ATP synthase subunit β, chloroplastic | 74.0 | 0.33 | 0.04 | Electron transport |
| 41 | O20304 | RBL | Ribulose bisphosphate carboxylase large chain (Fragment) | 41.2 | 0.33 | 0.01 | Carbohydrate metabolism |
| 42 | P42654 | 1433B | 14-3-3-like protein B | 59.6 | 0.34 | 0.03 | Photosynthesis |
| 43 | A7VLV1 | SGRW | Protein STAY-GREEN, chloroplastic | 126.5 | 0.35 | 0.05 | Saponin biosynthesis |
| 44 | D4Q9Z5 | SGT3 | Soyasaponin III rhamnosyltransferase | 13.3 | 0.35 | 0.04 | Defense response |
| 45 | Q96452 | 1433C | 14-3-3-like protein C | 88.0 | 0.36 | 0.03 | Monooxygenase |
| 46 | P93149 | C93B1 | Licodione synthase | 29.1 | 0.36 | 0.02 | Secondary metabolism |
| 47 | P52576 | IFR | Isoflavone reductase | 69.3 | 0.37 | 0.04 | Hormone metabolism |
| 48 | B5WWZ9 | FAO2 | Long-chain-alcohol oxidase FAO2 | 95.6 | 0.38 | 0.02 | Hormone metabolism |
| 49 | Q9SQ80 | G2OX1 | Gibberellin 2-β-dioxygenase 1 | 103.9 | 0.39 | 0.04 | Signal transduction |
| 50 | Q39836 | GBLP | Guanine nucleotide-binding protein  subunit β-like protein | 139.7 | 0.40 | 0.01 | Carbohydrate metabolism |
| 51 | A4GG89 | RBL | Ribulose bisphosphate carboxylase large chain | 32.5 | 0.40 | 0.05 | Fatty acid metabolism |
| 52 | Q42783 | BCCP | Biotin carboxyl carrier protein of acetyl-CoA  carboxylase, chloroplastic | 79.7 | 0.41 | 0.04 | Electron transport |
| 53 | P28552 | ATPG | ATP synthase γ chain, chloroplastic | 24.2 | 0.41 | 0.05 | Translation |
| 54 | Q2PMM3 | RK2B | 50S ribosomal protein L2-B, chloroplastic | 75.8 | 0.41 | 0.04 | Carbohydrate metabolism |
| 55 | O62943 | RBL | Ribulose bisphosphate carboxylase large chain | 56.8 | 0.42 | 0.04 | Signal tranduction |
| 56 | P62163 | CALM2 | Calmodulin-2 | 33.6 | 0.42 | 0.03 | Cell wall synthesis |
| 57 | Q43111 | PME3 | Pectinesterase 3 | 64.7 | 0.43 | 0.04 | Fatty acid metabolism |
| 58 | Q8W3P8 | AOG | Abscisate β-glucosyltransferase | 214.1 | 0.44 | 0.02 | Saponin biosynthesis |
| 59 | Q9LRH8 | BAMS | β-Amyrin synthase | 38.7 | 0.44 | 0.04 | Secondary metabolism |
| 60 | P19143 | PAL3 | Phenylalanine ammonia-lyase class 3 | 102.3 | 0.44 | 0.04 | Electron transport |
| 61 | A4GG90 | ATPB | ATP synthase subunit β, chloroplastic | 9.2 | 0.44 | 0.05 | Secondary metabolism |
| 62 | P26690 | 6DCS | NAD(P)H-dependent 6'-deoxychalcone synthase | 35.7 | 0.44 | 0.05 | Cell wall synthesis |
| 63 | P35334 | PGIP1 | Polygalacturonase inhibitor 1 | 74.5 | 0.45 | 0.04 | Electron transport |
| 64 | P05037 | ATPB | ATP synthase subunit β, chloroplastic | 15.2 | 0.45 | 0.02 | Secondary metabolism |
| 65 | I1L3T1 | 708D1 | UDP-glycosyltransferase 708D1 | 22.5 | 0.45 | 0.03 | Hormone metabolism |
| 66 | Q9XHM5 | G2OX2 | Gibberellin 2-β-dioxygenase 2 | 43.7 | 0.46 | 0.05 | Carbohydrate metabolism |
| 67 | O82515 | MTDH | Probable mannitol dehydrogenase | 102.9 | 0.46 | 0.03 | Signal transduction |
| 68 | O04369 | RAC1 | Rac-like GTP-binding protein RAC1 | 22.0 | 0.48 | 0.05 | Carbohydrate metabolism |
| 69 | Q01390 | SUS | Sucrose synthase | 57.4 | 0.48 | 0.01 | Secondary metabolism |
| 70 | Q42797 | TCMO | Trans-cinnamate 4-monooxygenase | 44.2 | 0.48 | 0.02 | Electron transport |
| 71 | Q9TKI7 | ATPB | ATP synthase subunit β, chloroplastic | 23.4 | 0.48 | 0.03 | Amino acid metabolism |
| 72 | P50246 | SAHH | Adenosylhomocysteinase | 53.1 | 0.49 | 0.01 | Nodulation |
| 73 | P10816 | LGB3 | Leghemoglobin 3 | 14.9 | 0.50 | 0.03 | Transport |
| **Unchanged protein** | | | | | | | |
| 74 | P04991 | RBL | Ribulose bisphosphate carboxylase large chain | 138.5 | 0.50 | 0.1 | Carbohydrate metabolism |
| 75 | P49093 | ASNS2 | Asparagine synthetase [glutamine-hydrolyzing] 2 | 165.7 | 0.51 | 0.3 | Amino acid metabolism |
| 76 | Q40357 | NO10 | Early nodulin-10 | 43.6 | 0.52 | 0.2 | Nodulation |
| 77 | P49092 | ASNS1 | Asparagine synthetase [glutamine-hydrolyzing] 1 | 11.8 | 0.52 | 0.5 | Amino acid metabolism |
| 78 | F5B8W1 | CONB3 | Conglutin β 3 | 95.6 | 0.52 | 0.5 | Seed storage protein |
| 79 | Q9BBU1 | RBL | Ribulose bisphosphate carboxylase large chain | 36.6 | 0.53 | 0.3 | Carbohydrate metabolism |
| 80 | A4GG84 | RR3 | 30S ribosomal protein S3, chloroplastic | 116.1 | 0.53 | 0.1 | Translation |
| 81 | Q5QGZ8 | NRL4A | Bifunctional nitrilase/nitrile hydratase NIT4A | 52.8 | 0.54 | 0.4 | Catalytic activity |
| 82 | P31926 | SUS | Sucrose synthase | 34.6 | 0.54 | 0.1 | Carbohydrate metabolism |
| 83 | P29530 | OLEO1 | P24 oleosin isoform A | 44.6 | 0.54 | 0.1 | Growth and development |
| 84 | P09886 | HS21C | Small heat shock protein, chloroplastic | 202.5 | 0.54 | 0.2 | Chaperone |
| 85 | P43237 | ALL11 | Allergen Ara h 1, clone P17 | 23.0 | 0.55 | 0.4 | Seed storage protein |
| 86 | Q42808 | TBP | TATA-box-binding protein | 109.7 | 0.55 | 0.1 | DNA binding |
| 87 | Q04672 | SBP | Sucrose-binding protein | 98.7 | 0.55 | 0.5 | Seed storage protein |
| 88 | O63094 | RBL | Ribulose bisphosphate carboxylase large chain | 36.6 | 0.55 | 0.5 | Carbohydrate metabolism |
| 89 | P02854 | VCLB | Provicilin (Fragment) | 63.3 | 0.55 | 0.2 | Seed storage protein |
| 90 | Q9BBP6 | NU5C | NAD(P)H-quinone oxidoreductase subunit 5, chloroplastic | 102.0 | 0.55 | 0.4 | Electron transport |
| 91 | A4GGB9 | CYF | Cytochrome f | 93.9 | 0.55 | 0.2 | Electron transport |
| 92 | Q41121 | NO30 | Nodulin-30 | 217.3 | 0.55 | 0.4 | Nodulation |
| 93 | Q5W915 | USP | UDP-sugar pyrophospharylase | 63.1 | 0.55 | 0.1 | Carbohydrate metabolism |
| 94 | A0A109QYD3 | RMS3 | Strigolactone esterase RMS3 | 110.8 | 0.55 | 0.4 | Defense response |
| 95 | P18823 | ACCD | Acetyl-coenzyme A carboxylase carboxyl transferase subunit β, chloroplastic | 55.3 | 0.57 | 0.2 | Fatty acid biosynthesis |
| 96 | P48490 | PP1 | Serine/threonine-protein phosphatase PP1 | 19.2 | 0.57 | 0.2 | Signal transduction |
| 97 | O48901 | DPOD1 | DNA polymerase δ catalytic subunit | 112.0 | 0.57 | 0.3 | Replication |
| 98 | P83594 | IFXA | Factor Xa inhibitor BuXI | 169.6 | 0.58 | 0.1 | Protease inhibitor |
| 99 | Q6WNR0 | C81E7 | Isoflavone 2'-hydroxylase | 69.1 | 0.59 | 0.4 | Secondary metabolism |
| 100 | P27456 | GSHRP | Glutathione reductase, chloroplastic/ mitochondrial | 43.4 | 0.59 | 0.1 | Redox homeostasis |
| 101 | Q40987 | LECR | Nodule lectin | 291.4 | 0.59 | 0.5 | Nodulation |
| 102 | P11728 | NO75 | Early nodulin-75 (Fragment) | 1382.1 | 0.59 | 0.1 | Nodulation |
| 103 | Q5YJU6 | MATK | Maturase K | 23.6 | 0.59 | 0.4 | mRNA processing |
| 104 | O49885 | RL13A | 60S ribosomal protein L13a | 144.4 | 0.60 | 0.4 | Translation |
| 105 | P25011 | CCNB1 | G2/mitotic-specific cyclin S13-6 | 116.6 | 0.60 | 0.4 | Cell cycle |
| 106 | Q6E2Z6 | REHY | 1-Cys peroxiredoxin | 51.0 | 0.61 | 0.1 | Redox homeostasis |
| 107 | Q5H873 | HLTT | 13-hydroxylupanine O-tigloyltransferase | 6.0 | 0.61 | 0.2 | Alkaloid biosynthesis |
| 108 | A6BM07 | I7GT1 | Isoflavone 7-O-glucosyltransferase 1 | 157.4 | 0.61 | 0.5 | Secondary metabolism |
| 109 | P37228 | MDHG | Malate dehydrogenase, glyoxysomal | 163.0 | 0.61 | 0.5 | Carbohydrate metabolism |
| 110 | Q9BBR8 | CYF | Cytochrome f | 108.4 | 0.61 | 0.5 | Electron transport |
| 111 | Q9AWB2 | SIR | Sulfite reductase [ferredoxin], chloroplastic (Fragment) | 66.2 | 0.61 | 0.2 | Replication |
| 112 | Q8MCJ4 | MATK | Maturase K | 30.8 | 0.62 | 0.2 | mRNA processing |
| 113 | Q9BBU0 | ATPB | ATP synthase subunit β, chloroplastic | 75.4 | 0.62 | 0.1 | Transport |
| 114 | Q43715 | TOC75 | Protein TOC75, chloroplastic | 60.7 | 0.63 | 0.4 | Transport |
| 115 | Q5YK47 | MATK | Maturase K | 34.5 | 0.63 | 0.5 | mRNA processing |
| 116 | P14594 | LEGB | Legumin B (Fragment) | 49.4 | 0.63 | 0.3 | Seed storage protein |
| 117 | O81980 | CFI1 | Chalcone-flavonone isomerase 1 (Fragment) | 7.1 | 0.63 | 0.1 | Secondary metabolism |
| 118 | P25698 | EF1A | Elongation factor 1-α | 95.4 | 0.63 | 0.4 | Translation |
| 119 | Q94IR2 | CCD1 | Carotenoid 9,10 (9',10')-cleavage dioxygenase 1 | 174.9 | 0.63 | 0.4 | Secondary metabolism |
| 120 | P49347 | CONB | Concanavalin B | 58.2 | 0.64 | 0.2 | Carbohydrate metabolism |
| 121 | P93471 | COP1 | E3 ubiquitin-protein ligase COP1 | 51.0 | 0.64 | 0.4 | Protein modification |
| 122 | Q96423 | TCMO | Trans-Cinnamate 4-monooxygenase | 89.8 | 0.64 | 0.3 | Secondary metabolism |
| 123 | Q9FZL4 | MGDG | Probable monogalactosyldiacylglycerol synthase,  chloroplastic | 99.8 | 0.66 | 0.3 | Lipid metabolism |
| 124 | P51139 | MSK3 | Glycogen synthase kinase-3 homolog MsK-3 | 186.1 | 0.66 | 0.5 | Signal transduction |
| 125 | Q6J540 | C79D4 | Isoleucine N-monooxygenase 2 | 66.2 | 0.66 | 0.3 | Secondary metabolism |
| 126 | Q07185 | AOX1 | Ubiquinol oxidase 1, mitochondrial | 63.4 | 0.67 | 0.4 | Electron transport |
| 127 | Q41266 | AOX2 | Ubiquinol oxidase 2, mitochondrial | 48.0 | 0.67 | 0.1 | Electron transport |
| 128 | O65015 | AMYB | β-Amylase | 122.3 | 0.67 | 0.5 | Carbohydrate metabolism |
| 129 | F5B8W5 | CONB7 | Conglutin β 7 | 65.7 | 0.67 | 0.1 | Seed storage protein |
| 130 | Q41008 | ACCA | Acetyl-coenzyme A carboxylase carboxyl transferase subunit α, chloroplastic | 47.4 | 0.67 | 0.4 | Fatty acid biosynthesis |
| 131 | P58822 | PGIP2 | Polygalacturonase inhibitor 2 | 74.5 | 0.68 | 0.1 | Cell wall synthesis |
| 132 | Q40316 | VESTR | Vestitone reductase | 110.0 | 0.68 | 0.5 | Defense response |
| 133 | P58823 | PGIP3 | Polygalacturonase inhibitor 3 | 26.7 | 0.68 | 0.2 | Cell wall synthesis |
| 134 | Q42919 | G6PD | Glucose-6-phosphate 1-dehydrogenase,  cytoplasmic isoform | 40.4 | 0.68 | 0.5 | Carbohydrate metabolism |
| 135 | P32646 | COX2 | Cytochrome c oxidase subunit 2,  mitochondrial (Fragment) | 89.6 | 0.68 | 0.2 | Electron transport |
| 136 | Q1W376 | PMM | Phosphomannomutase | 41.3 | 0.68 | 0.1 | Carbohydrate metabolism |
| 137 | P05311 | PSAB | Photosystem I P700 chlorophyll a apoprotein A2 | 17.3 | 0.68 | 0.1 | Photosynthesis |
| 138 | P27066 | RBL | Ribulose bisphosphate carboxylase large chain | 36.6 | 0.68 | 0.4 | Carbohydrate metabolism |
| 139 | P02873 | LEA1 | α-Amylase inhibitor 1 | 18.7 | 0.68 | 0.4 | Carbohydrate metabolism |
| 140 | Q2TE74 | PCS2 | Glutathione γ-glutamylcysteinyltransferase 2 | 35.4 | 0.69 | 0.5 | Hormone metabolism |
| 141 | P12628 | MAOX | NADP-dependent malic enzyme | 25.9 | 0.69 | 0.5 | Carbohydrate metabolism |
| 142 | Q43876 | SPSA | Probable sucrose-phosphate synthase | 73.5 | 0.69 | 0.2 | Carbohydrate metabolism |
| 143 | P92397 | RBL | Ribulose bisphosphate carboxylase large chain (Fragment) | 115.1 | 0.69 | 0.4 | Carbohydrate metabolism |
| 144 | Q8MCR7 | MATK | Maturase K | 26.3 | 0.70 | 0.3 | mRNA processing |
| 145 | O04235 | SSRP1 | FACT complex subunit SSRP1 | 87.1 | 0.70 | 0.3 | DNA Repair |
| 146 | P93324 | CHOMT | Isoliquiritigenin 2'-O-methyltransferase | 34.1 | 0.70 | 0.1 | Secondary metabolism |
| 147 | Q43092 | SSG1 | Granule-bound starch synthase 1, chloroplastic/ amyloplastic | 148.5 | 0.70 | 0.3 | Carbohydrate metabolism |
| 148 | D2XNR0 | FLOT3 | Flotillin-like protein 3 | 48.1 | 0.70 | 0.4 | Defense response |
| 149 | P19251 | ASNS1 | Asparagine synthetase, nodule [glutamine-hydrolyzing] | 113.4 | 0.72 | 0.2 | Amino acid metabolism |
| 150 | P69589 | RBL | Ribulose bisphosphate carboxylase large chain (Fragment) | 115.1 | 0.72 | 0.5 | Carbohydrate metabolism |
| 151 | Q42798 | C93A1 | 3,9-dihydroxypterocarpan 6α-monooxygenase | 110.9 | 0.72 | 0.1 | Defense response |
| 152 | O04862 | FOLM | Folate synthesis bifunctional protein, mitochondrial | 109.1 | 0.72 | 0.2 | Nucleotide metabolism |
| 153 | Q6RET7 | CCAMK | Calcium and calcium/ calmodulin-dependent  serine/threonine-protein kinase DMI-3 | 55.4 | 0.73 | 0.1 | Signal transduction |
| 154 | Q2TSC7 | PCS1 | Glutathione γ-glutamylcysteinyltransferase 1 | 6.2 | 0.73 | 0.4 | Hormone metabolism |
| 155 | P13915 | CVCA | Convicilin | 57.2 | 0.73 | 0.1 | Seed storage protein |
| 156 | Q8MCA5 | RR3 | 30S ribosomal protein S3, chloroplastic | 101.2 | 0.73 | 0.5 | Translation |
| 157 | Q9BBN7 | RR15 | 30S ribosomal protein S15, chloroplastic | 67.5 | 0.73 | 0.5 | Translation |
| 158 | Q0GXS4 | NFP | Serine/threonine receptor-like kinase NFP | 123.6 | 0.73 | 0.5 | Signal transduction |
| 159 | P06669 | CYF | Cytochrome f | 291.8 | 0.73 | 0.2 | Electron transport |
| 160 | P49161 | CYF | Cytochrome f | 24.0 | 0.74 | 0.4 | Electron transport |
| 161 | B5LMP9 | PSBB | Photosystem II CP47 reaction center protein | 58.7 | 0.74 | 0.3 | Photosynthesis |
| 162 | Q9TKP6 | MATK | Maturase K | 50.1 | 0.74 | 0.3 | mRNA processing |
| 163 | O24301 | SUS2 | Sucrose synthase 2 | 93.3 | 0.75 | 0.5 | Carbohydrate metabolism |
| 164 | Q2PMP7 | RR3 | 30S ribosomal protein S3, chloroplastic | 139.1 | 0.75 | 0.5 | Translation |
| 165 | P69572 | RBL | Ribulose bisphosphate carboxylase large chain (Fragment) | 115.1 | 0.75 | 0.5 | Carbohydrate metabolism |
| 166 | Q5YK03 | MATK | Maturase K | 23.8 | 0.75 | 0.1 | mRNA processing |
| 167 | P46277 | CCNB1 | G2/ mitotic-specific cyclin-1 | 18.2 | 0.76 | 0.3 | Cell cycle |
| 168 | P28553 | CRTI | Phytoene dehydrogenase, chloroplastic/ chromoplastic | 65.3 | 0.76 | 0.2 | Pigment Metabolism |
| 169 | O24509 | INVA | Acid β-fructofuranosidase | 46.4 | 0.76 | 0.4 | Carbohydrate metabolism |
| 170 | P69591 | RBL | Ribulose bisphosphate carboxylase large chain (Fragment) | 115.1 | 0.76 | 0.3 | Carbohydrate metabolism |
| 171 | P13708 | SUS | Sucrose synthase | 50.7 | 0.76 | 0.3 | Carbohydrate metabolism |
| 172 | Q42777 | MCCA | Methylcrotonoyl-CoA carboxylase subunit α, mitochondrial | 59.4 | 0.76 | 0.5 | Amino acid metabolism |
| 173 | O81973 | C93A3 | Cytochrome P450 93A3 | 38.7 | 0.76 | 0.5 | Monooxygenase |
| 174 | B3EWQ9 | LECA2 | Lectin α chain | 231.2 | 0.76 | 0.3 | Carbohydrate binding |
| 175 | Q8MCM4 | MATK | Maturase K | 68.6 | 0.76 | 0.2 | mRNA processing |
| 176 | Q93XE6 | CFI1A | Chalcone-flavonone isomerase 1A | 112.3 | 0.76 | 0.5 | Secondary metabolism |
| 177 | P10933 | FENR | Ferredoxin--NADP reductase, leaf isozyme, chloroplastic | 79.6 | 0.76 | 0.4 | Photosynthesis |
| 178 | P30124 | HEM2 | δ-aminolevulinic acid dehydratase, chloroplastic (Fragment) | 31.2 | 0.76 | 0.5 | Pigment Metabolism |
| 179 | P34811 | EFGC1 | Elongation factor G-1, chloroplastic | 80.2 | 0.76 | 0.3 | Translation |
| 180 | P69584 | RBL | Ribulose bisphosphate carboxylase large chain (Fragment) | 115.1 | 0.76 | 0.3 | Carbohydrate metabolism |
| 181 | Q42807 | STAD | Stearoyl-[acyl-carrier-protein] 9-desaturase,  chloroplastic | 297.6 | 0.76 | 0.3 | Lipid metabolism |
| 182 | P12859 | G3PB | Glyceraldehyde-3-phosphate dehydrogenase B,  chloroplastic | 72.8 | 0.77 | 0.5 | Carbohydrate metabolism |
| 183 | P41346 | FENR | Ferredoxin-NADP reductase, chloroplastic | 143.6 | 0.77 | 0.3 | Photosynthesis |
| 184 | A4GGD1 | PSBB | Photosystem II CP47 reaction center protein | 34.5 | 0.77 | 0.2 | Photosynthesis |
| 185 | Q5YK50 | MATK | Maturase K | 44.5 | 0.77 | 0.1 | mRNA processing |
| 186 | Q4VYC8 | NSP1 | Nodulation-signaling pathway 1 protein | 103.2 | 0.77 | 0.1 | Nodulation |
| 187 | P04347 | GLYG5 | Glycinin | 53.0 | 0.78 | 0.4 | Seed storage protein |
| 188 | P29001 | INVA | Acid β-fructofuranosidase | 62.9 | 0.78 | 0.3 | Carbohydrate metabolism |
| 189 | P45458 | MASY | Malate synthase, glyoxysomal (Fragment) | 42.1 | 0.78 | 0.1 | Carbohydrate metabolism |
| 190 | Q9BBS1 | ACCD | Acetyl-coenzyme A carboxylase carboxyl transferase subunit β, chloroplastic | 83.6 | 0.78 | 0.5 | Fatty acid biosynthesis |
| 191 | Q43067 | TCMO | Trans-cinnamate 4-monooxygenase | 46.5 | 0.78 | 0.1 | Secondary metabolism |
| 192 | I1K0K6 | EFGC2 | Elongation factor G-2, chloroplastic | 75.5 | 0.78 | 0.3 | Translation |
| 193 | F5B8W3 | CONB5 | Conglutin β 5 | 67.6 | 0.78 | 0.5 | Seed storage protein |
| 194 | Q5YK01 | MATK | Maturase K | 4.9 | 0.78 | 0.4 | mRNA processing |
| 195 | A3RF67 | BAGBG | Isoflavonoid 7-O-β-apiosyl-glucoside β-glycosidase | 77.5 | 0.78 | 0.5 | Secondary metabolism |
| 196 | Q2PMU3 | PSAB | Photosystem I P700 chlorophyll a apoprotein A1 | 79.6 | 0.79 | 0.3 | Photosynthesis |
| 197 | P93998 | RBL | Ribulose bisphosphate carboxylase large chain (Fragment) | 38.7 | 0.79 | 0.2 | Carbohydrate metabolism |
| 198 | P24099 | GLNA1 | Glutamine synthetase cytosolic isozyme 1 | 20.9 | 0.79 | 0.2 | Amino acid metabolism |
| 199 | P53536 | PHSL | α-1,4 Glucan phosphorylase L isozyme, chloroplastic/ amyloplastic | 120.6 | 0.79 | 0.1 | Carbohydrate metabolism |
| 200 | A4GG97 | PSAA | Photosystem I P700 chlorophyll a apoprotein A1 | 79.6 | 0.79 | 0.5 | Photosynthesis |
| 201 | Q8MCR8 | MATK | Maturase K | 73.0 | 0.79 | 0.3 | mRNA processing |
| 202 | P46280 | EFTU2 | Elongation factor Tu, chloroplastic | 28.2 | 0.79 | 0.3 | Translation |
| 203 | Q01861 | PAL1 | Phenylalanine ammonia-lyase 1 | 87.9 | 0.79 | 0.3 | Secondary metabolism |
| 204 | P05310 | PSAA | Photosystem I P700 chlorophyll a apoprotein A1 | 95.2 | 0.79 | 0.1 | Photosynthesis |
| 205 | P08281 | GLNA2 | Glutamine synthetase leaf isozyme, chloroplastic | 14.8 | 0.79 | 0.2 | Amino acid metabolism |
| 206 | P69579 | RBL | Ribulose bisphosphate carboxylase large chain (Fragment) | 115.1 | 0.79 | 0.3 | Carbohydrate metabolism |
| 207 | Q40983 | SPP | Stromal processing peptidase, chloroplastic | 63.4 | 0.79 | 0.3 | Proteolysis |
| 208 | Q9MUK5 | TOC64 | Translocon at the outer membrane of chloroplasts 64 | 121.3 | 0.79 | 0.2 | Transport |
| 209 | O04279 | GPA2 | Guanine nucleotide-binding protein α-2 subunit | 43.2 | 0.79 | 0.1 | Signal transduction |
| 210 | O65194 | RBS | Ribulose bisphosphate carboxylase small chain, chloroplastic | 23.1 | 0.80 | 0.5 | Carbohydrate metabolism |
| 211 | P13918 | VCLC | Vicilin | 94.2 | 0.80 | 0.4 | Seed storage protein |
| 212 | Q9SM60 | PGMC | Phosphoglucomutase, cytoplasmic | 131.3 | 0.80 | 0.5 | Carbohydrate metabolism |
| 213 | Q9BBP8 | RR3 | 30S ribosomal protein S3, chloroplastic | 78.8 | 0.80 | 0.1 | Translation |
| 214 | Q43056 | CFI | Chalcone-flavonone isomerase | 68.2 | 0.80 | 0.5 | Secondary metabolism |
| 215 | O49817 | LEA2 | Late embryogenesis abundant protein 2 | 74.4 | 0.80 | 0.4 | Defense response |
| 216 | P46258 | ACT3 | Actin-3 | 92.2 | 0.81 | 0.5 | Cytoskeleton |
| 217 | P27480 | LOXA | Linoleate 9S-lipoxygenase 1 | 31.9 | 0.81 | 0.5 | Fatty acid biosynthesis |
| 218 | Q5YJX4 | MATK | Maturase K | 12.8 | 0.81 | 0.2 | mRNA processing |
| 219 | P69580 | RBL | Ribulose bisphosphate carboxylase large chain (Fragment) | 115.1 | 0.81 | 0.1 | Carbohydrate metabolism |
| 220 | P49364 | GCST | Aminomethyltransferase, mitochondrial | 76.5 | 0.81 | 0.5 | Amino acid metabolism |
| 221 | P86349 | EFGC | Elongation factor G, chloroplastic (Fragments) | 56.0 | 0.81 | 0.5 | Translation |
| 222 | O48921 | C97B2 | Cytochrome P450 97B2, chloroplastic | 237.0 | 0.81 | 0.5 | Monooxygenase |
| 223 | Q9SWB4 | PARP3 | Poly [ADP-ribose] polymerase 3 | 42.7 | 0.82 | 0.1 | Transcription |
| 224 | Q8MCM9 | MATK | Maturase K | 24.9 | 0.82 | 0.3 | mRNA processing |
| 225 | P19252 | ASNS2 | Asparagine synthetase, root [glutamine-hydrolyzing] | 137.0 | 0.82 | 0.2 | Amino acid metabolism |
| 226 | Q6TND0 | MATK | Maturase K | 110.6 | 0.82 | 0.1 | mRNA processing |
| 227 | Q9BBS3 | ATPA | ATP synthase subunit α, chloroplastic | 38.3 | 0.82 | 0.4 | Transport |
| 228 | Q9M6E8 | NCED1 | 9-cis-epoxycarotenoid dioxygenase NCED1, chloroplastic | 50.8 | 0.82 | 0.4 | Hormone metabolism |
| 229 | P26205 | BGLT | Cyanogenic β-glucosidase (Fragment) | 38.9 | 0.82 | 0.5 | Carbohydrate metabolism |
| 230 | P31163 | RK2 | 50S ribosomal protein L2, chloroplastic | 82.3 | 0.82 | 0.2 | Translation |
| 231 | Q41062 | SECA | Protein translocase subunit SecA, chloroplastic | 78.4 | 0.82 | 0.2 | Transport |
| 232 | Q41060 | SBP65 | Seed biotin-containing protein SBP65 | 96.9 | 0.82 | 0.1 | Seed storage protein |
| 233 | Q8MCJ5 | MATK | Maturase K | 39.7 | 0.83 | 0.3 | mRNA processing |
| 234 | Q9SM59 | PGMP | Phosphoglucomutase, chloroplastic | 203.4 | 0.83 | 0.5 | Carbohydrate metabolism |
| 235 | A4GGA5 | RPOB | DNA-directed RNA polymerase subunit β | 82.3 | 0.83 | 0.4 | Transcription |
| 236 | Q9M6E9 | AGGL | Agglutinin-1 | 171.2 | 0.83 | 0.3 | Carbohydrate binding |
| 237 | Q5YJX3 | MATK | Maturase K | 22.9 | 0.83 | 0.2 | mRNA processing |
| 238 | P34922 | G3PC | Glyceraldehyde-3-phosphate dehydrogenase, cytosolic | 91.0 | 0.84 | 0.1 | Carbohydrate metabolism |
| 239 | O48560 | CATA3 | Catalase-3 | 25.2 | 0.84 | 0.5 | Redox homeostasis |
| 240 | Q9TK08 | MATK | Maturase K | 138.0 | 0.84 | 0.4 | mRNA processing |
| 241 | C6L7U1 | LIN1 | Putative E3 ubiquitin-protein ligase LIN-1 | 123.8 | 0.84 | 0.5 | Protein modification |
| 242 | P42348 | PI3K2 | Phosphatidylinositol 3-kinase, nodule isoform | 88.7 | 0.84 | 0.3 | Lipid metabolism |
| 243 | Q04903 | FNTB | Protein farnesyltransferase subunit β | 70.7 | 0.84 | 0.2 | Lipid metabolism |
| 244 | O48665 | LGB5 | Leghemoglobin Lb120-29 | 191.8 | 0.84 | 0.3 | Nodulation |
| 245 | P29756 | CATA1 | Catalase-1/2 | 23.5 | 0.84 | 0.2 | Redox homeostasis |
| 246 | Q8MCN3 | MATK | Maturase K | 53.7 | 0.84 | 0.4 | mRNA processing |
| 247 | Q2PMT9 | PSBC | Photosystem II CP43 reaction center protein | 66.1 | 0.84 | 0.1 | Photosynthesis |
| 248 | P45457 | ACEA2 | Isocitrate lyase 2 (Fragment) | 70.5 | 0.84 | 0.5 | Carbohydrate metabolism |
| 249 | Q9TKP0 | MATK | Maturase K | 157.5 | 0.84 | 0.3 | mRNA processing |
| 250 | Q75NZ0 | SIR | Sulfite reductase [ferredoxin], chloroplastic | 64.3 | 0.85 | 0.4 | Replication |
| 251 | P93673 | PHYA | Phytochrome type A | 31.2 | 0.85 | 0.1 | Signal transduction |
| 252 | Q5YJU0 | MATK | Maturase K | 43.0 | 0.85 | 0.4 | mRNA processing |
| 253 | P04776 | GLYG1 | Glycinin G1 | 72.1 | 0.85 | 0.2 | Seed storage protein |
| 254 | P13088 | AUX22 | Auxin-induced protein AUX22 | 87.8 | 0.85 | 0.1 | Growth and development |
| 255 | Q8MCR6 | MATK | Maturase K | 28.5 | 0.86 | 0.4 | mRNA processing |
| 256 | Q2PMQ9 | PSBB | Photosystem II CP47 reaction center protein | 84.6 | 0.86 | 0.4 | Photosynthesis |
| 257 | Q02909 | CAPP1 | Phosphoenolpyruvate carboxylase, housekeeping isozyme | 41.2 | 0.86 | 0.1 | Carbohydrate metabolism |
| 258 | Q9AU12 | CAPP | Phosphoenolpyruvate carboxylase | 45.6 | 0.86 | 0.5 | Carbohydrate metabolism |
| 259 | P00155 | CYF | Cytochrome f | 48.4 | 0.86 | 0.3 | Electron transport |
| 260 | A0A0G4DBR5 | FG3H | UDP-glycosyltransferase 79B30 | 12.2 | 0.87 | 0.5 | Carbohydrate metabolism |
| 261 | P24459 | ATPAM | ATP synthase subunit α, mitochondrial | 103.9 | 0.87 | 0.4 | Transport |
| 262 | Q672F7 | TPS2 | Tricyclene synthase EBOS, chloroplastic | 25.1 | 0.87 | 0.2 | Secondary metabolism |
| 263 | D1FP53 | LIN | Putative E3 ubiquitin-protein ligase LIN | 118.8 | 0.87 | 0.2 | Protein modification |
| 264 | P37116 | NCPR | NADPH-cytochrome P450 reductase | 80.5 | 0.87 | 0.4 | Electron transport |
| 265 | P15231 | PHAM | Leucoagglutinating phytohemagglutinin | 90.0 | 0.87 | 0.2 | Defense response |
| 266 | Q9TKI5 | MATK | Maturase K | 23.5 | 0.87 | 0.4 | mRNA processing |
| 267 | Q8MCR5 | MATK | Maturase K | 28.5 | 0.88 | 0.1 | mRNA processing |
| 268 | O24648 | G3OX | Gibberellin 3-β-dioxygenase 1 | 128.9 | 0.88 | 0.5 | Hormone metabolism |
| 269 | P43238 | ALL12 | Allergen Ara h 1, clone P41B | 9.7 | 0.88 | 0.4 | Seed storage protein |
| 270 | Q9SPB1 | LEGRE | Leghemoglobin reductase | 41.0 | 0.88 | 0.1 | Nodulation |
| 271 | K7LC65 | DAT1C | Diacylglycerol O-acyltransferase 1C | 30.9 | 0.88 | 0.1 | Lipid metabolism |
| 272 | Q9BBT1 | PSBC | Photosystem II CP43 reaction center protein | 66.1 | 0.88 | 0.2 | Photosynthesis |
| 273 | P55195 | PUR6 | Phosphoribosylaminoimidazole carboxylase, chloroplastic (Fragment) | 53.3 | 0.88 | 0.4 | Nucleotide metabolism |
| 274 | P13548 | VATA | V-type proton ATPase catalytic subunit A | 135.0 | 0.88 | 0.2 | Transport |
| 275 | P37114 | TCMO | Trans-cinnamate 4-monooxygenase | 67.4 | 0.88 | 0.1 | Secondary metabolism |
| 276 | P51061 | CAPP2 | Phosphoenolpyruvate carboxylase | 38.4 | 0.88 | 0.4 | Carbohydrate metabolism |
| 277 | Q5YJU7 | MATK | Maturase K | 47.1 | 0.88 | 0.3 | mRNA processing |
| 278 | Q5YK53 | MATK | Maturase K | 22.9 | 0.88 | 0.2 | mRNA processing |
| 279 | P34799 | URIC2 | Uricase-2 isozyme 2 | 15.5 | 0.88 | 0.5 | Nodulation |
| 280 | P31165 | RK15 | 50S ribosomal protein L15, chloroplastic (Fragment) | 44.4 | 0.88 | 0.5 | Translation |
| 281 | P06006 | PSBD | Photosystem II D2 protein | 512.7 | 0.88 | 0.2 | Photosynthesis |
| 282 | Q6E4Q8 | MATK | Maturase K | 92.0 | 0.88 | 0.3 | mRNA processing |
| 283 | Q9GI85 | MATK | Maturase K | 81.2 | 0.88 | 0.5 | mRNA processing |
| 284 | A0A072VMJ3 | CN15C | Protein CNGC15c | 44.1 | 0.88 | 0.3 | Transport |
| 285 | Q02028 | HSP7S | Stromal 70 kDa heat shock-related protein, chloroplastic | 58.3 | 0.89 | 0.2 | Chaperone |
| 286 | Q9SMJ4 | LEG | Legumin | 88.8 | 0.89 | 0.1 | Seed storage protein |
| 287 | O24303 | TI110 | Protein TIC110, chloroplastic | 110.7 | 0.89 | 0.3 | Transport |
| 288 | Q8MCL7 | MATK | Maturase K | 102.9 | 0.89 | 0.5 | mRNA processing |
| 289 | P54774 | CDC48 | Cell division cycle protein 48 homolog | 70.0 | 0.89 | 0.4 | Growth and development |
| 290 | Q6Q7X3 | MATK | Maturase K | 95.8 | 0.89 | 0.3 | mRNA processing |
| 291 | D1FP57 | LIN2 | Putative E3 ubiquitin-protein ligase LIN-2 | 74.5 | 0.89 | 0.4 | Protein modification |
| 292 | A4GGA1 | PSBC | Photosystem II CP43 reaction center protein | 66.1 | 0.89 | 0.4 | Photosynthesis |
| 293 | Q42899 | GLNA1 | Glutamine synthetase cytosolic isozyme | 1.2 | 0.89 | 0.1 | Amino acid metabolism |
| 294 | P23569 | CHSY | Chalcone synthase | 57.4 | 0.89 | 0.3 | Secondary metabolism |
| 295 | I1N2Z5 | SLE1 | Protein SLE1 | 81.1 | 0.89 | 0.1 | Signal transduction |
| 296 | P13089 | AUX28 | Auxin-induced protein AUX28 | 47.7 | 0.89 | 0.3 | Growth and development |
| 297 | Q8MCL8 | MATK | Maturase K | 78.8 | 0.90 | 0.4 | mRNA processing |
| 298 | A0A172J2D0 | UGT2 | UDP-glycosyltransferase 2 | 72.9 | 0.90 | 0.5 | Carbohydrate metabolism |
| 299 | O80405 | LGB3 | Leghemoglobin Lb120-1 | 483.0 | 0.90 | 0.5 | Nodulation |
| 300 | Q6TND4 | MATK | Maturase K | 110.6 | 0.90 | 0.1 | mRNA processing |
| 301 | Q8HVY5 | RPOB | DNA-directed RNA polymerase subunit β | 85.0 | 0.90 | 0.3 | Transcription |
| 302 | P42347 | PI3K1 | Phosphatidylinositol 3-kinase, root isoform | 110.0 | 0.90 | 0.1 | Lipid metabolism |
| 303 | B5LMM0 | PSBC | Photosystem II CP43 reaction center protein | 66.1 | 0.90 | 0.1 | Photosynthesis |
| 304 | G7IBJ4 | CN15A | Protein CNGC15a | 29.3 | 0.90 | 0.1 | Transport |
| 305 | Q8MCM1 | MATK | Maturase K | 82.5 | 0.90 | 0.5 | mRNA processing |
| 306 | Q948P5 | FRI4 | Ferritin-4, chloroplastic | 120.0 | 0.90 | 0.5 | Photosynthesis |
| 307 | Q9SXV6 | CAS1 | Cycloartenol synthase | 74.3 | 0.90 | 0.5 | Saponin biosynthesis |
| 308 | Q5YJY5 | MATK | Maturase K | 62.1 | 0.90 | 0.1 | mRNA processing |
| 309 | Q6EBC1 | CONB2 | Conglutin β 2 | 113.2 | 0.90 | 0.4 | Seed storage protein |
| 310 | P29828 | PDI | Protein disulfide-isomerase | 61.9 | 0.90 | 0.4 | Redox homeostasis |
| 311 | Q9SAZ0 | LB120-34 | Leghemoglobin Lb120-34 | 220.4 | 0.90 | 0.1 | Nodulation |
| 312 | P38414 | LOX1 | Linoleate 9S-lipoxygenase | 30.4 | 0.90 | 0.5 | Fatty acid biosynthesis |
| 313 | P02858 | GLYG4 | Glycinin G4 | 57.1 | 0.90 | 0.1 | Seed storage protein |
| 314 | Q9TKR9 | MATK | Maturase K | 37.1 | 0.90 | 0.1 | mRNA processing |
| 315 | Q8MCS1 | MATK | Maturase K | 22.1 | 0.91 | 0.5 | mRNA processing |
| 316 | O22437 | CHLD | Magnesium-chelatase subunit ChlD, chloroplastic | 22.2 | 0.91 | 0.3 | Photosynthesis |
| 317 | Q8LKZ1 | NORK | Nodulation receptor kinase | 31.1 | 0.91 | 0.4 | Nodulation |
| 318 | Q6RET6 | CCAMK | Calcium and calcium/calmodulin-dependent  serine/threonine-protein kinase (Fragment) | 123.2 | 0.91 | 0.5 | Signal transduction |
| 319 | O65026 | SUS | Sucrose synthase | 37.9 | 0.91 | 0.4 | Carbohydrate metabolism |
| 320 | Q5YJV9 | MATK | Maturase K | 27.8 | 0.91 | 0.4 | mRNA processing |
| 321 | B5LMM1 | PBSD | Photosystem II D2 protein | 512.7 | 0.91 | 0.1 | Photosynthesis |
| 322 | O20346 | RBL | Ribulose bisphosphate carboxylase large chain (Fragment) | 36.6 | 0.91 | 0.4 | Carbohydrate metabolism |
| 323 | Q8S3J3 | HIUH | Hydroxyisourate hydrolase | 225.7 | 0.91 | 0.3 | Nucleotide metabolism |
| 324 | Q8MCP4 | MATK | Maturase K | 31.8 | 0.92 | 0.1 | mRNA processing |
| 325 | Q8MCL9 | MATK | Maturase K | 115.0 | 0.92 | 0.1 | mRNA processing |
| 326 | Q39846 | SBP65 | Seed biotin-containing protein SBP65 | 147.9 | 0.92 | 0.5 | Seed storage protein |
| 327 | Q96450 | 1433A | 14-3-3-like protein A | 67.6 | 0.92 | 0.5 | Defense response |
| 328 | P32290 | CATA | Catalase | 32.6 | 0.92 | 0.4 | Redox homeostasis |
| 329 | P16149 | RS16 | 40S ribosomal protein S16 | 334.8 | 0.92 | 0.1 | Translation |
| 330 | A9UL14 | RBR | Retinoblastoma-related protein | 79.1 | 0.92 | 0.5 | Cell cycle |
| 331 | C0HK20 | LECC1 | Mannose-specific lectin CML-2 | 27.1 | 0.92 | 0.3 | Carbohydrate binding |
| 332 | Q9AVK4 | SCR | Protein SCARECROW | 33.6 | 0.92 | 0.5 | Cell cycle |
| 333 | P35055 | HEM6 | Oxygen-dependent coproporphyrinogen-III oxidase, chloroplastic | 79.7 | 0.93 | 0.2 | Pigment Metabolism |
| 334 | P42499 | PHYB | Phytochrome B | 51.2 | 0.93 | 0.1 | Signal transduction |
| 335 | Q02226 | COXT | Cytochrome C oxidase subunit 2, mitochondrial (Fragment) | 39.9 | 0.93 | 0.3 | Electron transport |
| 336 | I1N462 | SBT1 | Subtilisin-like protease | 272.8 | 0.93 | 0.4 | Defense response |
| 337 | P48640 | GSHRP | Glutathione reductase, chloroplastic | 32.0 | 0.93 | 0.5 | Redox homeostasis |
| 338 | P39870 | NIA2 | Inducible nitrate reductase [NADH] 2 | 90.9 | 0.93 | 0.3 | Nodulation |
| 339 | Q9BBU2 | MATK | Maturase K | 87.4 | 0.94 | 0.5 | mRNA processing |
| 340 | Q6PSE2 | MATK | Maturase K | 33.3 | 0.94 | 0.1 | mRNA processing |
| 341 | Q8MCP5 | MATK | Maturase K | 31.8 | 0.95 | 0.2 | mRNA processing |
| 342 | P48628 | FAD6C | ω-6 fatty acid desaturase, chloroplastic | 16.5 | 0.95 | 0.2 | Fatty acid biosynthesis |
| 343 | Q8HVY3 | RPOC2 | DNA-directed RNA polymerase subunit β | 89.0 | 0.95 | 0.1 | Transcription |
| 344 | Q41014 | FENR2 | Ferredoxin-NADP reductase, root isozyme, chloroplastic | 79.7 | 0.95 | 0.1 | Photosynthesis |
| 345 | P93163 | GPA2 | Guanine nucleotide-binding proteinα-2 subunit | 112.4 | 0.95 | 0.3 | Signal transduction |
| 346 | O64981 | RCA | Ribulose bisphosphate carboxylase/ oxygenase activase, chloroplastic | 141.6 | 0.96 | 0.2 | Carbohydrate metabolism |
| 347 | P09439 | LOX2 | Seed linoleate 9S-lipoxygenase-2 | 62.8 | 0.96 | 0.1 | Lipid metabolism |
| 348 | Q6PSC4 | MATK | Maturase K | 48.0 | 0.96 | 0.2 | mRNA processing |
| 349 | Q6UDF0 | CSLA1 | Mannan synthase 1 | 28.5 | 0.96 | 0.5 | Carbohydrate metabolism |
| 350 | Q9MB42 | BAMS | β-Amyrin synthase | 19.8 | 0.96 | 0.1 | Saponin biosynthesis |
| 351 | P25699 | FRI | Ferritin, chloroplastic | 151.2 | 0.96 | 0.3 | Photosynthesis |
| 352 | Q8MCP6 | MATK | Maturase K | 23.5 | 0.97 | 0.5 | mRNA processing |
| 353 | Q8MCN9 | MATK | Maturase K | 102.9 | 0.97 | 0.5 | mRNA processing |
| 354 | P12227 | RPOC2 | DNA-directed RNA polymerase subunit β (Fragment) | 29.3 | 0.97 | 0.2 | Transcription |
| 355 | Q9BBS9 | RPOB | DNA-directed RNA polymerase subunit β | 87.2 | 0.97 | 0.5 | Transcription |
| 356 | Q5H8A5 | POLLU | Ion channel POLLUX | 49.3 | 0.97 | 0.2 | Transport |
| 357 | Q39821 | SDLCA | Dynamin-related protein 12A | 138.7 | 0.97 | 0.5 | Growth and development |
| 358 | Q41114 | LEA2 | α-Amylase inhibitor 2 | 75.9 | 0.97 | 0.3 | Carbohydrate metabolism |
| 359 | Q5YJV6 | MATK | Maturase K | 97.5 | 0.97 | 0.5 | mRNA processing |
| 360 | P14298 | CFI | Chalcone-flavonone isomerase | 44.6 | 0.97 | 0.4 | Secondary metabolism |
| 361 | P06004 | PBSC | Photosystem II CP43 reaction center protein | 66.1 | 0.97 | 0.2 | Photosynthesis |
| 362 | P04770 | GLNA1 | Glutamine synthetase PR-1 | 64.1 | 0.97 | 0.2 | Amino acid metabolism |
| 363 | P39869 | NIA | Nitrate reductase [NADH] | 74.5 | 0.97 | 0.5 | Nodulation |
| 364 | Q8MCN8 | MATK | Maturase K | 109.3 | 0.98 | 0.4 | mRNA processing |
| 365 | Q8MCN0 | MATK | Maturase K | 109.3 | 0.98 | 0.2 | mRNA processing |
| 366 | Q8MCM3 | MATK | Maturase K | 103.1 | 0.98 | 0.5 | mRNA processing |
| 367 | Q8MCK8 | MATK | Maturase K | 63.1 | 0.98 | 0.3 | mRNA processing |
| 368 | Q9SLZ4 | RBR1 | Retinoblastoma-related protein 1 | 218.7 | 0.98 | 0.3 | Cell cycle |
| 369 | Q9FVL0 | HBL1 | Non-symbiotic hemoglobin 1 | 79.3 | 0.98 | 0.1 | Transport |
| 370 | Q43082 | HEM3 | Porphobilinogen deaminase, chloroplastic | 103.5 | 0.98 | 0.4 | Pigment Metabolism |
| 371 | A5JTQ3 | XYL2 | β-Xylosidase/α-L-arabinofuranosidase 2 | 48.8 | 0.98 | 0.5 | Cell wall synthesis |
| 372 | P57997 | IF2C | Translation initiation factor IF-2, chloroplastic | 42.9 | 0.98 | 0.1 | Translation |
| 373 | P69590 | RBL | Ribulose bisphosphate carboxylase large chain (Fragment) | 115.1 | 0.98 | 0.4 | Carbohydrate metabolism |
| 374 | Q42805 | PUR3 | Phosphoribosylglycinamide formyltransferase, chloroplastic | 80.3 | 0.98 | 0.3 | Nucleotide metabolism |
| 375 | Q41058 | GLGB1 | 1,4-α-glucan-branching enzyme 1, chloroplastic/ amyloplastic | 58.8 | 0.98 | 0.2 | Carbohydrate metabolism |
| 376 | Q6E4Q3 | MATK | Maturase K | 15.8 | 0.98 | 0.2 | mRNA processing |
| 377 | Q6DW76 | DGDG1 | Digalactosyldiacylglycerol synthase 1, chloroplastic | 99.8 | 0.98 | 0.3 | Cell wall synthesis |
| 378 | P21616 | AVP | Pyrophosphate-energized vacuolar membrane proton pump | 40.8 | 0.98 | 0.2 | Transport |
| 379 | Q70DJ5 | LECC1 | α-methyl-mannoside-specific lectin | 108.9 | 0.98 | 0.1 | Carbohydrate binding |
| 380 | I1KEV6 | FG3H | UDP-glycosyltransferase 79B30 | 13.4 | 0.98 | 0.3 | Carbohydrate metabolism |
| 381 | O04865 | PLDA1 | Phospholipase D α 1 | 47.7 | 0.98 | 0.3 | Lipid metabolism |
| 382 | P11140 | ABRA | Abrin-a | 62.4 | 0.99 | 0.2 | Defense response |
| 383 | Q8MCM8 | MATK | Maturase K | 61.7 | 0.99 | 0.1 | mRNA processing |
| 384 | Q8MCM7 | MATK | Maturase K | 93.8 | 0.99 | 0.4 | mRNA processing |
| 385 | Q8MCM2 | MATK | Maturase K | 109.7 | 0.99 | 0.1 | mRNA processing |
| 386 | P13916 | GLCA | β-conglycinin, α chain | 0.0 | 0.99 | 0.1 | Seed storage protein |
| 387 | A4GGF4 | TI214 | Protein TIC 214 | 87.8 | 0.99 | 0.4 | Transport |
| 388 | Q8L4H4 | NORK | Nodulation receptor kinase | 24.2 | 0.99 | 0.4 | Nodulation |
| 389 | P69575 | RBL | Ribulose bisphosphate carboxylase large chain (Fragment) | 115.1 | 0.99 | 0.3 | Carbohydrate metabolism |
| 390 | Q5YK00 | MATK | Maturase K | 4.9 | 0.99 | 0.2 | mRNA processing |
| 391 | Q53HY0 | CONB1 | Conglutin β 1 | 24.3 | 0.99 | 0.4 | Seed storage protein |
| 392 | P31023 | DLDH | Dihydrolipoyl dehydrogenase, mitochondrial | 107.1 | 0.99 | 0.5 | Catalytic activity |
| 393 | Q8MCN1 | MATK | Maturase K | 57.6 | 1.00 | 0.1 | mRNA processing |
| 394 | P17067 | CAHC | Carbonic anhydrase, chloroplastic | 60.2 | 1.00 | 0.4 | Catalytic activity |
| 395 | P51137 | MSK1 | Glycogen synthase kinase-3 homolog MsK-1 | 212.0 | 1.00 | 0.3 | Signal transduction |
| 396 | Q5D1C1 | MATK | Maturase K | 34.9 | 1.00 | 0.2 | mRNA processing |
| 397 | P37115 | TCMO | Trans-cinnamate 4-monooxygenase | 105.5 | 1.00 | 0.1 | Secondary metabolism |
| 398 | Q33438 | RBL | Ribulose bisphosphate carboxylase large chain (Fragment) | 65.6 | 1.00 | 0.3 | Carbohydrate metabolism |
| 399 | Q40224 | GPA1 | Guanine nucleotide-binding protein α-1 subunit | 84.3 | 1.00 | 0.5 | Signal transduction |
| 400 | Q9TKP8 | MATK | Maturase K | 31.7 | 1.00 | 0.3 | mRNA processing |
| 401 | O81928 | TCMO | Trans-cinnamate 4-monooxygenase | 52.7 | 1.00 | 0.5 | Secondary metabolism |
| 402 | Q8MCR9 | MATK | Maturase K | 113.7 | 1.01 | 0.5 | mRNA processing |
| 403 | A4ULF8 | METK | S-Adenosylmethionine synthase | 28.5 | 1.01 | 0.4 | Amino acid metabolism |
| 404 | Q8MCN2 | MATK | Maturase K | 102.9 | 1.01 | 0.2 | mRNA processing |
| 405 | Q43078 | C97B1 | Cytochrome P450 97B1, chloroplastic | 73.9 | 1.01 | 0.5 | Monooxygenase |
| 406 | P69573 | RBL | Ribulose bisphosphate carboxylase large chain (Fragment) | 115.1 | 1.01 | 0.4 | Carbohydrate metabolism |
| 407 | Q5YK05 | MATK | Maturase K | 17.6 | 1.01 | 0.1 | mRNA processing |
| 408 | Q39469 | THD1 | Threonine dehydratase biosynthetic, chloroplastic | 61.9 | 1.01 | 0.1 | Amino acid metabolism |
| 409 | P39866 | NIA2 | Nitrate reductase [NADH] 2 | 41.6 | 1.01 | 0.3 | Nodulation |
| 410 | Q6WNQ9 | C81E9 | Isoflavone 3'-hydroxylase (Fragment) | 133.2 | 1.02 | 0.2 | Secondary metabolism |
| 411 | P80366 | PPAF | Fe(3+)-Zn(2+) purple acid phosphatase | 49.2 | 1.02 | 0.4 | Catalytic activity |
| 412 | Q9XHM1 | EIF3C | Eukaryotic translation initiation factor 3 subunit C | 59.2 | 1.02 | 0.5 | Translation |
| 413 | Q9BBT0 | PBSD | Photosystem II D2 protein | 512.7 | 1.02 | 0.3 | Photosynthesis |
| 414 | P26969 | GCSP | Glycine dehydrogenase (decarboxylating), mitochondrial | 24.4 | 1.02 | 0.4 | Amino acid metabolism |
| 415 | P20077 | THS2 | Putative stilbene synthase 2 (Fragment) | 40.6 | 1.02 | 0.3 | Secondary metabolism |
| 416 | Q39891 | LEU1 | Probable 2-isopropylmalate synthase | 102.5 | 1.02 | 0.5 | Amino acid metabolism |
| 417 | P08283 | H1 | Histone H1 | 101.2 | 1.02 | 0.5 | DNA binding |
| 418 | P69582 | RBL | Ribulose bisphosphate carboxylase large chain (Fragment) | 115.1 | 1.02 | 0.1 | Carbohydrate metabolism |
| 419 | F5B8V9 | CONB1 | Conglutin β 1 | 64.2 | 1.02 | 0.5 | Seed storage protein |
| 420 | P58310 | PSAA | Photosystem I P700 chlorophyll a apoprotein A1 | 81.4 | 1.02 | 0.4 | Photosynthesis |
| 421 | Q1SGF1 | PARP3 | Putative poly [ADP-ribose] polymerase 3 | 60.7 | 1.02 | 0.2 | Transcription |
| 422 | P49613 | METK | S-Adenosylmethionine synthase 2 | 28.5 | 1.02 | 0.4 | Amino acid metabolism |
| 423 | Q6DW74 | DGDG1 | Digalactosyldiacylglycerol synthase 1,chloroplastic | 99.1 | 1.02 | 0.4 | Cell wall synthesis |
| 424 | Q9SWR5 | C93C1 | 2-hydroxyisoflavanone synthase | 23.5 | 1.02 | 0.5 | Secondary metabolism |
| 425 | Q9TKS6 | MATK | Maturase K | 111.1 | 1.02 | 0.2 | mRNA processing |
| 426 | Q8HUG7 | MATK | Maturase K | 48.3 | 1.03 | 0.2 | mRNA processing |
| 427 | O24534 | EF1A | Elongation factor 1-α | 77.5 | 1.03 | 0.4 | Translation |
| 428 | A4GGA7 | RPOC2 | DNA-directed RNA polymerase subunit β | 40.4 | 1.03 | 0.5 | Transcription |
| 429 | P39865 | NIA1 | Nitrate reductase [NADH] 1 | 50.7 | 1.03 | 0.4 | Nodulation |
| 430 | Q9TKS1 | MATK | Maturase K | 37.3 | 1.03 | 0.1 | mRNA processing |
| 431 | P50477 | CANA | Canavalin | 4.6 | 1.03 | 0.5 | Seed storage protein |
| 432 | Q2PMU2 | PSAB | Photosystem I P700 chlorophyll a apoprotein A2 | 40.8 | 1.04 | 0.4 | Photosynthesis |
| 433 | Q43468 | HSOP1 | Hsp70-Hsp90 organizing protein 1 | 95.0 | 1.04 | 0.5 | Chaperone |
| 434 | G7JND3 | CN15B | Protein CNGC15b | 63.3 | 1.04 | 0.2 | Transport |
| 435 | A4GGA2 | PSBD | Photosystem II D2 protein | 512.7 | 1.04 | 0.1 | Photosynthesis |
| 436 | P69581 | RBL | Ribulose bisphosphate carboxylase large chain (Fragment) | 115.1 | 1.04 | 0.2 | Carbohydrate metabolism |
| 437 | P32296 | P5CS | δ-1-pyrroline-5-carboxylate synthase | 72.2 | 1.04 | 0.3 | Amino acid metabolism |
| 438 | Q5YJU1 | MATK | Maturase K | 87.4 | 1.04 | 0.5 | mRNA processing |
| 439 | Q04593 | PAL2 | Phenylalanine ammonia-lyase 2 | 90.2 | 1.04 | 0.1 | Secondary metabolism |
| 440 | P42500 | PHYA | Phytochrome A | 51.8 | 1.04 | 0.3 | Signal transduction |
| 441 | Q8MCM0 | MATK | Maturase K | 91.2 | 1.05 | 0.3 | mRNA processing |
| 442 | P48406 | CHS5 | Chalcone synthase 5 | 18.9 | 1.05 | 0.4 | Secondary metabolism |
| 443 | Q84KK6 | I4OMT | Isoflavone 4'-O-methyltransferase | 160.1 | 1.05 | 0.5 | Secondary metabolism |
| 444 | P51109 | DFRA | Dihydroflavonol 4-reductase (Fragment) | 88.3 | 1.05 | 0.1 | Secondary metabolism |
| 445 | O98997 | RCA | Ribulose bisphosphate carboxylase/ oxygenase activase, chloroplastic | 36.1 | 1.05 | 0.5 | Carbohydrate metabolism |
| 446 | P10562 | CANA | Canavalin | 4.6 | 1.05 | 0.1 | Seed storage protein |
| 447 | P69588 | RBL | Ribulose bisphosphate carboxylase large chain (Fragment) | 115.1 | 1.05 | 0.2 | Carbohydrate metabolism |
| 448 | P08215 | ATPA | ATP synthase subunit α, chloroplastic | 40.3 | 1.05 | 0.2 | Transport |
| 449 | O62970 | RBL | Ribulose bisphosphate carboxylase large chain (Fragment) | 36.6 | 1.05 | 0.3 | Carbohydrate metabolism |
| 450 | B5WWZ8 | FAO1 | Long-chain-alcohol oxidase FAO1 | 105.3 | 1.05 | 0.1 | Lipid metabolism |
| 451 | Q9TKP9 | MATK | Maturase K | 47.7 | 1.05 | 0.5 | mRNA processing |
| 452 | Q03460 | GLSN | Glutamate synthase [NADH], amyloplastic | 40.1 | 1.06 | 0.2 | Amino acid metabolism |
| 453 | Q8MCN7 | MATK | Maturase K | 104.2 | 1.06 | 0.3 | mRNA processing |
| 454 | Q2PMS8 | ATPA | ATP synthase subunit α, chloroplastic | 126.4 | 1.06 | 0.4 | Transport |
| 455 | P08926 | RUBA | RuBisCO large subunit-binding protein subunit α, chloroplastic | 33.6 | 1.06 | 0.1 | Carbohydrate metabolism |
| 456 | P30706 | PLSB | Glycerol-3-phosphate acyltransferase, chloroplastic | 17.7 | 1.06 | 0.5 | Carbohydrate metabolism |
| 457 | P69578 | RBL | Ribulose bisphosphate carboxylase large chain (Fragment) | 115.1 | 1.06 | 0.4 | Carbohydrate metabolism |
| 458 | P69576 | RBL | Ribulose bisphosphate carboxylase large chain (Fragment) | 115.1 | 1.06 | 0.4 | Carbohydrate metabolism |
| 459 | F5B8W2 | CONB4 | Conglutin β 4 | 106.6 | 1.06 | 0.1 | Seed storage protein |
| 460 | P49612 | METK1 | S-Adenosylmethionine synthase 1 (Fragment) | 76.5 | 1.06 | 0.2 | Amino acid metabolism |
| 461 | Q02735 | CAPP | Phosphoenolpyruvate carboxylase | 52.3 | 1.06 | 0.3 | Carbohydrate metabolism |
| 462 | P31687 | 4CL2 | 4-Coumarate-CoA ligase 2 | 48.9 | 1.07 | 0.5 | Secondary metabolism |
| 463 | Q02920 | NO70 | Early nodulin-70 | 21.0 | 1.07 | 0.3 | Nodulation |
| 464 | Q9BBQ8 | PSBB | Photosystem II CP47 reaction center protein | 24.5 | 1.07 | 0.5 | Photosynthesis |
| 465 | P69587 | RBL | Ribulose bisphosphate carboxylase large chain (Fragment) | 115.1 | 1.07 | 0.5 | Carbohydrate metabolism |
| 466 | P69585 | RBL | Ribulose bisphosphate carboxylase large chain (Fragment) | 115.1 | 1.07 | 0.5 | Carbohydrate metabolism |
| 467 | Q01288 | CHS6 | Chalcone synthase 6 | 34.8 | 1.08 | 0.1 | Secondary metabolism |
| 468 | Q96558 | UGDH1 | UDP-glucose 6-dehydrogenase 1 | 82.3 | 1.08 | 0.4 | Carbohydrate metabolism |
| 469 | Q40106 | TBB2 | Tubulin β-2 chain | 171.1 | 1.08 | 0.2 | Cytoskeleton |
| 470 | Q8SKU2 | TIC62 | Protein TIC 62, chloroplastic | 99.1 | 1.08 | 0.2 | Transport |
| 471 | Q9BBN6 | TI214 | Protein TIC 214 | 83.5 | 1.08 | 0.4 | Transport |
| 472 | Q39817 | CALX | Calnexin homolog | 85.5 | 1.08 | 0.2 | Chaperone |
| 473 | C6TEX6 | FEN1 | Flap endonuclease 1 | 36.9 | 1.08 | 0.1 | DNA Repair |
| 474 | P69586 | RBL | Ribulose bisphosphate carboxylase large chain (Fragment) | 115.1 | 1.08 | 0.1 | Carbohydrate metabolism |
| 475 | P69577 | RBL | Ribulose bisphosphate carboxylase large chain (Fragment) | 115.1 | 1.08 | 0.1 | Carbohydrate metabolism |
| 476 | F5B8W4 | CONB6 | Conglutin β 6 | 102.7 | 1.08 | 0.3 | Seed storage protein |
| 477 | Q41706 | UPSA3 | Probable ureide permease A3 (Fragment) | 158.3 | 1.08 | 0.3 | Cell wall synthesis |
| 478 | P92407 | RBL | Ribulose bisphosphate carboxylase large chain (Fragment) | 115.1 | 1.08 | 0.5 | Carbohydrate metabolism |
| 479 | P92401 | RBL | Ribulose bisphosphate carboxylase large chain (Fragment) | 115.1 | 1.08 | 0.1 | Carbohydrate metabolism |
| 480 | Q9ZNX6 | GSH1 | Glutamate-cysteine ligase, chloroplastic | 43.3 | 1.08 | 0.2 | Amino acid metabolism |
| 481 | P24826 | CHS1 | Chalcone synthase 1 | 18.9 | 1.09 | 0.1 | Secondary metabolism |
| 482 | Q84KK4 | I4OMT | Isoflavone 4'-O-methyltransferase | 219.5 | 1.09 | 0.2 | Secondary metabolism |
| 483 | A0A172J2G3 | UGT43 | UDP-glycosyltransferase 43 | 68.4 | 1.09 | 0.3 | Carbohydrate metabolism |
| 484 | Q41107 | INO1 | Inositol-3-phosphate synthase | 46.0 | 1.09 | 0.2 | Lipid metabolism |
| 485 | A4GG98 | PSAB | Photosystem I P700 chlorophyll a apoprotein A2 | 11.6 | 1.09 | 0.4 | Photosynthesis |
| 486 | Q40313 | CAMT | Caffeoyl-CoA O-methyltransferase | 57.8 | 1.09 | 0.2 | Secondary metabolism |
| 487 | P92406 | RBL | Ribulose bisphosphate carboxylase large chain (Fragment) | 115.1 | 1.09 | 0.3 | Carbohydrate metabolism |
| 488 | O64407 | AMYB | β-Amylase | 84.2 | 1.09 | 0.5 | Carbohydrate metabolism |
| 489 | O04408 | KSA | Ent-copalyl diphosphate synthase, chloroplastic | 43.2 | 1.11 | 0.2 | Hormone metabolism |
| 490 | P07218 | PAL1 | Phenylalanine ammonia-lyase class 1 (Fragment) | 77.5 | 1.11 | 0.3 | Secondary metabolism |
| 491 | B8R4B1 | NU5C | NAD(P)H-quinone oxidoreductase subunit5C; chloroplastic | 15.9 | 1.11 | 0.5 | Electron transport |
| 492 | D2XNQ9 | FLOT2 | Flotillin-like protein 2 | 13.7 | 1.11 | 0.4 | Defense response |
| 493 | P49082 | GPA1 | Guanine nucleotide-binding protein α-1 subunit | 104.0 | 1.11 | 0.2 | Signal transduction |
| 494 | Q5YK33 | MATK | Maturase K | 54.4 | 1.11 | 0.4 | mRNA processing |
| 495 | Q42806 | KPYC | Pyruvate kinase, cytosolic isozyme | 94.4 | 1.11 | 0.2 | Carbohydrate metabolism |
| 496 | P08863 | NO26B | Nodulin-26B | 36.0 | 1.11 | 0.2 | Nodulation |
| 497 | P14856 | LOX2 | Seed linoleate 9S-lipoxygenase-2 | 75.1 | 1.11 | 0.5 | Lipid metabolism |
| 498 | O22585 | AMYB | β-Amylase | 12.4 | 1.11 | 0.2 | Carbohydrate metabolism |
| 499 | P08170 | LOX1 | Seed linoleate 13S-lipoxygenase-1 | 30.5 | 1.11 | 0.5 | Lipid metabolism |
| 500 | P35100 | CLPC | Chaperone protein ClpC, chloroplastic | 170.8 | 1.11 | 0.4 | Chaperone |
| 501 | P37900 | HSP7M | Heat shock 70 kDa protein, mitochondrial | 67.5 | 1.11 | 0.2 | Chaperone |
| 502 | Q43460 | NO20A | Nodulin-20a | 124.7 | 1.12 | 0.5 | Nodulation |
| 503 | Q9ZSK5 | ZOG | Zeatin O-glucosyltransferase | 35.0 | 1.12 | 0.2 | Hormone metabolism |
| 504 | P98188 | C94A2 | Cytochrome P450 94A2 | 60.2 | 1.12 | 0.3 | Monooxygenase |
| 505 | Q6PP79 | MATK | Maturase K | 4.0 | 1.12 | 0.3 | mRNA processing |
| 506 | P49044 | VPE | Vacuolar-processing enzyme | 88.4 | 1.12 | 0.1 | Seed storage protein |
| 507 | F5B8W0 | CONB2 | Conglutin β 2 | 117.7 | 1.12 | 0.3 | Seed storage protein |
| 508 | Q2PMP0 | TI214 | Protein TIC 214 | 101.6 | 1.13 | 0.2 | Transport |
| 509 | Q9BBS7 | RPOC2 | DNA-directed RNA polymerase subunit β | 25.4 | 1.13 | 0.3 | Transcription |
| 510 | Q43089 | INV1 | β-Fructofuranosidase, cell wall isozyme | 14.6 | 1.13 | 0.1 | Cell wall synthesis |
| 511 | D2XNQ8 | FLOT1 | Flotillin-like protein 1 | 184.9 | 1.13 | 0.4 | Defense response |
| 512 | Q5YJX5 | MATK | Maturase K | 36.0 | 1.13 | 0.1 | mRNA processing |
| 513 | Q09WE7 | USP1 | UDP-sugar pyrophosphorylase 1 | 68.6 | 1.13 | 0.3 | Carbohydrate metabolism |
| 514 | Q5NUF4 | HIDM | 2-hydroxyisoflavanone dehydratase | 15.0 | 1.13 | 0.3 | Secondary metabolism |
| 515 | Q9LRH7 | ABAMS | Mixed-amyrin synthase | 100.5 | 1.14 | 0.5 | Saponin biosynthesis |
| 516 | Q00016 | IFR | Isoflavone reductase | 61.9 | 1.14 | 0.5 | Secondary metabolism |
| 517 | P48621 | FAD3C | ω-3 Fatty acid desaturase, chloroplastic | 348.5 | 1.14 | 0.4 | Fatty acid biosynthesis |
| 518 | Q43621 | GSHRC | Glutathione reductase, cytosolic | 45.0 | 1.14 | 0.2 | Redox homeostasis |
| 519 | P69583 | RBL | Ribulose bisphosphate carboxylaselarge chain (Fragment) | 115.1 | 1.14 | 0.3 | Carbohydrate metabolism |
| 520 | P49084 | GPA1 | Guanine nucleotide-binding protein α-1 subunit | 50.9 | 1.14 | 0.4 | Signal transduction |
| 521 | P51062 | CAPP | Phosphoenolpyruvate carboxylase | 33.9 | 1.15 | 0.5 | Carbohydrate metabolism |
| 522 | P32293 | AX22A | Auxin-induced protein 22A | 77.2 | 1.15 | 0.4 | Growth and development |
| 523 | P69574 | RBL | Ribulose bisphosphate carboxylase large chain (Fragment) | 115.1 | 1.15 | 0.2 | Carbohydrate metabolism |
| 524 | Q06215 | PPO | Polyphenol oxidase A1, chloroplastic | 34.8 | 1.15 | 0.4 | Pigment Metabolism |
| 525 | Q9SAZ1 | LGB4 | Leghemoglobin Lb120-8 | 150.8 | 1.15 | 0.1 | Nodulation |
| 526 | Q40353 | MMK2 | Mitogen-activated protein kinase homolog MMK2 | 138.0 | 1.16 | 0.2 | Signal transduction |
| 527 | C6TAY1 | SOMT2 | Flavonoid 4'-O-methyltransferase | 30.8 | 1.16 | 0.2 | Secondary metabolism |
| 528 | Q01912 | 1A1C | 1-Aminocyclopropane-1-carboxylate synthase (Fragment) | 0.0 | 1.16 | 0.5 | Hormone metabolism |
| 529 | P17957 | CHS2 | Chalcone synthase 2 | 18.9 | 1.17 | 0.3 | Secondary metabolism |
| 530 | P09918 | LOX3 | Seed linoleate 9S-lipoxygenase-3 | 40.6 | 1.17 | 0.2 | Lipid metabolism |
| 531 | P07374 | UREA | Urease | 39.6 | 1.17 | 0.5 | Nodulation |
| 532 | P09186 | LOX3 | Seed linoleate 9S-lipoxygenase-3 | 67.5 | 1.19 | 0.5 | Lipid metabolism |
| 533 | P08960 | NO20A | Nodulin-20 | 5.4 | 1.19 | 0.4 | Nodulation |
| 534 | Q43066 | GLNA4 | Glutamine synthetase root isozyme β | 16.3 | 1.20 | 0.2 | Amino acid metabolism |
| 535 | Q6RHR6 | DMI1 | Ion channel DMI1 | 53.2 | 1.20 | 0.5 | Transport |
| 536 | O49816 | LEA1 | Late embryogenesis abundant protein 1 | 46.0 | 1.20 | 0.4 | Defense response |
| 537 | P54233 | NIA1 | Inducible nitrate reductase [NADH] 1 | 31.1 | 1.20 | 0.3 | Nodulation |
| 538 | P02855 | VCLA | Provicilin (Fragment) | 68.4 | 1.20 | 0.3 | Seed storage protein |
| 539 | O24310 | EFTU | Elongation factor Tu, chloroplastic | 8.8 | 1.21 | 0.2 | Translation |
| 540 | D2XNR1 | FLOT4 | Flotillin-like protein 4 | 26.4 | 1.21 | 0.4 | Defense response |
| 541 | Q39828 | SDL5A | Dynamin-related protein 5A | 121.1 | 1.21 | 0.2 | Growth and development |
| 542 | P19142 | PAL2 | Phenylalanine ammonia-lyase class 2 | 80.3 | 1.21 | 0.3 | Secondary metabolism |
| 543 | P52423 | PUR3 | Phosphoribosylglycinamide formyltransferase, chloroplastic | 4.8 | 1.21 | 0.1 | Nucleotide metabolism |
| 544 | P27990 | PALY | Phenylalanine ammonia-lyase | 219.8 | 1.21 | 0.3 | Secondary metabolism |
| 545 | Q9B133 | RR12 | 30S ribosomal protein S12, chloroplastic | 62.2 | 1.21 | 0.4 | Translation |
| 546 | P05493 | ATPAM | ATP synthase subunit α, mitochondrial | 113.4 | 1.21 | 0.1 | Transport |
| 547 | Q9SMK9 | PAL2 | Phenylalanine ammonia-lyase 2 | 19.8 | 1.22 | 0.3 | Secondary metabolism |
| 548 | P25890 | CATA | Catalase | 29.5 | 1.22 | 0.4 | Redox homeostasis |
| 549 | A4GGC9 | RR12 | 30S ribosomal protein S12, chloroplastic | 53.9 | 1.22 | 0.3 | Translation |
| 550 | A4GGA6 | RPOC1 | DNA-directed RNA polymerase subunit β | 48.7 | 1.22 | 0.1 | Transcription |
| 551 | O49856 | FTRC | Ferredoxin-thioredoxin reductase catalytic chain, chloroplastic | 2.0 | 1.22 | 0.4 | Photosynthesis |
| 552 | P38417 | LOX4 | Linoleate 9S-lipoxygenase-4 | 56.1 | 1.22 | 0.2 | Fatty acid biosynthesis |
| 553 | P52780 | SYQ | Glutamine-tRNA ligase | 31.7 | 1.23 | 0.3 | Translation |
| 554 | P27481 | LOXB | Linoleate 9S-lipoxygenase (Fragment) | 85.4 | 1.25 | 0.5 | Fatty acid biosynthesis |
| 555 | Q84N37 | PVIP | OBERON-like protein (Fragment) | 128.5 | 1.25 | 0.2 | Growth and development |
| 556 | Q8HVY4 | RPOC1 | DNA-directed RNA polymerasesubunit β | 44.6 | 1.25 | 0.4 | Transcription |
| 557 | P24095 | LOXX | Seed linoleate 9S-lipoxygenase | 45.9 | 1.26 | 0.3 | Lipid metabolism |
| 558 | P52416 | GLGS1 | Glucose-1-phosphate adenylyltransferase small subunit 1, chloroplastic | 103.9 | 1.26 | 0.3 | Carbohydrate metabolism |
| 559 | P07134 | RR12 | 30S ribosomal protein S12, chloroplastic | 59.9 | 1.26 | 0.5 | Translation |
| 560 | P11827 | GLCAP | β-Conglycinin, α chain | 36.2 | 1.26 | 0.1 | Seed storage protein |
| 561 | Q01899 | HSP7M | Heat shock 70 kDa protein, mitochondrial | 44.6 | 1.27 | 0.4 | Chaperone |
| 562 | O49931 | TIC55 | Protein TIC 55, chloroplastic | 153.3 | 1.27 | 0.1 | Transport |
| 563 | Q9BBS8 | RPOC1 | DNA-directed RNA polymerase subunit β | 63.5 | 1.27 | 0.4 | Transcription |
| 564 | Q43088 | RBCMT | Ribulose-1,5 bisphosphate carboxylase/oxygenase large subunit N-methyltransferase, chloroplastic | 81.6 | 1.27 | 0.1 | Carbohydrate metabolism |
| 565 | P22895 | P34 | P34 probable thiol protease | 67.3 | 1.27 | 0.5 | Proteolysis |
| 566 | P15001 | PHYA | Phytochrome A | 33.5 | 1.27 | 0.1 | Signal transduction |
| 567 | Q69F95 | C85A | Cytochrome P450 85A | 95.4 | 1.27 | 0.5 | Monooxygenase |
| 568 | O24304 | FNTA | Protein farnesyltransferase/ geranylgeranyltransferase type-1 subunit α | 40.7 | 1.28 | 0.1 | Lipid metabolism |
| 569 | D2XNR2 | FLOT6 | Flotillin-like protein 6 | 99.1 | 1.28 | 0.1 | Defense response |
| 570 | P29502 | TBB3 | Tubulin β-3 chain (Fragment) | 3.8 | 1.28 | 0.4 | Cytoskeleton |
| 571 | O49818 | LGUL | Lactoylglutathione lyase | 67.3 | 1.28 | 0.4 | Secondary metabolism |
| 572 | P30080 | CHS6 | Chalcone synthase 6 | 18.9 | 1.28 | 0.3 | Secondary metabolism |
| 573 | P37392 | TBB1 | Tubulin β-1 chain | 3.8 | 1.28 | 0.4 | Cytoskeleton |
| 574 | Q8W3Y4 | METK | S-Adenosylmethionine synthase | 131.0 | 1.28 | 0.2 | Amino acid metabolism |
| 575 | Q93XK2 | STSYN | Stachyose synthase | 58.5 | 1.30 | 0.2 | Carbohydrate metabolism |
| 576 | O24308 | TOP2 | DNA topoisomerase 2 | 115.3 | 1.30 | 0.1 | Replication |
| 577 | P28551 | TBB3 | Tubulin β chain (Fragment) | 3.8 | 1.30 | 0.3 | Cytoskeleton |
| 578 | P30164 | ACT1 | Actin-1 | 40.4 | 1.30 | 0.2 | Cytoskeleton |
| 579 | Q41651 | CYPB | Peptidyl-prolyl cis-trans isomerase, chloroplastic | 89.6 | 1.30 | 0.3 | Protein modification |
| 580 | P07694 | GLNA3 | Glutamine synthetase root isozyme A | 34.1 | 1.30 | 0.2 | Amino acid metabolism |
| 581 | P29450 | TRXF | Thioredoxin F-type, chloroplastic | 37.5 | 1.31 | 0.5 | Electron transport |
| 582 | P13919 | CVCB | Convicilin (Fragment) | 85.9 | 1.32 | 0.1 | Seed storage protein |
| 583 | P45732 | PALY | Phenylalanine ammonia-lyase | 78.1 | 1.32 | 0.2 | Secondary metabolism |
| 584 | P53537 | PHSH | α-Glucan phosphorylase, H isozyme | 44.9 | 1.32 | 0.1 | Carbohydrate metabolism |
| 585 | Q41011 | EF1A | Elongation factor 1-α | 83.0 | 1.32 | 0.2 | Translation |
| 586 | P53392 | SUT2 | High affinity sulfate transporter 2 | 150.1 | 1.34 | 0.2 | Transport |
| 587 | Q5H8A6 | CASTO | Ion channel CASTOR | 20.0 | 1.34 | 0.4 | Transport |
| 588 | H1A981 | C7263 | 11-Oxo-β-amyrin 30-oxidase | 62.8 | 1.34 | 0.1 | Saponin biosynthesis |
| 589 | Q8VWN6 | RFS | Galactinol-sucrose galactosyltransferase | 22.1 | 1.34 | 0.1 | Carbohydrate metabolism |
| 590 | Q6PSB9 | MATK | Maturase K | 41.3 | 1.34 | 0.1 | mRNA processing |
| 591 | Q6PP78 | MATK | Maturase K | 35.8 | 1.34 | 0.1 | mRNA processing |
| 592 | G1CWH1 | CYC2 | Cliotide T2 | 194.9 | 1.34 | 0.2 | Defense response |
| 593 | P27991 | PAL1 | Phenylalanine ammonia-lyase 1 | 144.0 | 1.35 | 0.4 | Secondary metabolism |
| 594 | P34798 | URIC1 | Uricase-2 isozyme 1 | 35.0 | 1.35 | 0.2 | Nodulation |
| 595 | P53391 | SUT1 | High affinity sulfate transporter 1 | 204.0 | 1.36 | 0.4 | Transport |
| 596 | Q43077 | AMO | Primary amine oxidase | 9.0 | 1.36 | 0.4 | Hormone metabolism |
| 597 | Q5NUF3 | HIDH | 2-hydroxyisoflavanone dehydratase | 50.4 | 1.36 | 0.1 | Secondary metabolism |
| 598 | P13911 | RPOA | DNA-directed RNA polymerase subunit α | 236.1 | 1.38 | 0.2 | Transcription |
| 599 | Q8GT66 | TIC40 | Protein TIC 40, chloroplastic | 204.6 | 1.39 | 0.5 | Transport |
| 600 | Q6J541 | C79D3 | Isoleucine N-monooxygenase 1 | 43.2 | 1.39 | 0.5 | Secondary metabolism |
| 601 | D4Q9Z4 | SGT2 | Soyasapogenol β glucuronide galactosyltransferase | 56.3 | 1.39 | 0.3 | Saponin biosynthesis |
| 602 | Q39857 | XTH1 | Xyloglucan endotransglucosylase/hydrolase 1 | 53.6 | 1.39 | 0.1 | Cell wall synthesis |
| 603 | P10538 | AMYB | β-Amylase | 59.0 | 1.39 | 0.1 | Carbohydrate metabolism |
| 604 | P32289 | GLNA | Glutamine synthetase nodule isozyme | 40.8 | 1.39 | 0.4 | Nodulation |
| 605 | Q9BAE0 | FTSH | ATP-dependent zinc metalloprotease FTSH, chloroplastic | 56.1 | 1.39 | 0.3 | Proteolysis |
| 606 | P08438 | VCL | Vicilin | 77.9 | 1.39 | 0.3 | Seed storage protein |
| 607 | P30165 | ACT2 | Actin-2 | 51.7 | 1.40 | 0.1 | Cytoskeleton |
| 608 | Q43467 | EFTU1 | Elongation factor Tu, chloroplastic | 62.9 | 1.42 | 0.3 | Translation |
| 609 | P29531 | OLEO2 | P24 oleosin isoform B | 107.3 | 1.42 | 0.3 | Growth and development |
| 610 | P45734 | PALY | Phenylalanine ammonia-lyase | 86.9 | 1.43 | 0.5 | Secondary metabolism |
| 611 | Q948P6 | FRI3 | Ferritin-3, chloroplastic | 108.8 | 1.43 | 0.5 | Photosynthesis |
| 612 | A5JTQ2 | XYL1 | β-Xylosidase/ α-L-Arabinofuranosidase 1 (Fragment) | 49.2 | 1.43 | 0.3 | Cell wall synthesis |
| 613 | O48922 | C98A2 | Cytochrome P450 98A2 | 77.6 | 1.43 | 0.3 | Monooxygenase |
| 614 | P08688 | ALB2 | Albumin-2 | 50.7 | 1.45 | 0.3 | Seed storage protein |
| 615 | O04278 | GPA1 | Guanine nucleotide-binding protein α-1 subunit | 70.1 | 1.45 | 0.3 | Signal transduction |
| 616 | P12886 | ADH1 | Alcohol dehydrogenase 1 | 17.5 | 1.46 | 0.5 | Catalytic activity |
| 617 | P31531 | 1A1C | 1-Aminocyclopropane-1-carboxylate synthase | 2.3 | 1.46 | 0.2 | Hormone metabolism |
| 618 | P12468 | RBS4 | Ribulose bisphosphate carboxylase small chain 4, chloroplastic | 102.0 | 1.46 | 0.4 | Carbohydrate metabolism |
| 619 | Q94G16 | TATB | Sec-independent protein translocase protein TATB, chloroplastic | 85.7 | 1.46 | 0.5 | Transport |
| 620 | P34899 | GLYM | Serine hydroxymethyltransferase, mitochondrial | 92.3 | 1.48 | 0.5 | Amino acid metabolism |
| 621 | P52418 | PUR1 | Amidophosphoribosyltransferase, chloroplastic | 43.1 | 1.48 | 0.2 | Nucleotide metabolism |
| 622 | C0HJB3 | MANA | α-Mannosidase | 38.2 | 1.48 | 0.1 | Carbohydrate metabolism |
| 623 | Q43138 | MTDH3 | Probable mannitol dehydrogenase 3 | 122.0 | 1.49 | 0.5 | Carbohydrate metabolism |
| 624 | Q5UB07 | TPS4 | Tricyclene synthase TPS4, chloroplastic | 112.5 | 1.49 | 0.1 | Secondary metabolism |
| 625 | P51082 | CHSB | Chalcone synthase 1B | 24.0 | 1.49 | 0.2 | Secondary metabolism |
| 626 | P42353 | RRL16 | 50S ribosomal protein L16; chloroplastic (Fragment) | 53.0 | 1.49 | 0.1 | Translation |
| 627 | P58385 | PSAB | Photosystem I P700 chlorophyll a apoprotein A2 | 31.8 | 1.51 | 0.3 | Photosynthesis |
| 628 | P26413 | HSP70 | Heat shock 70 kDa protein | 67.3 | 1.51 | 0.5 | Chaperone |
| 629 | P0DH60 | M3OM2 | (+)-6a-hydroxymaackiain 3-O-methyltransferase 2 | 61.7 | 1.52 | 0.1 | Secondary metabolism |
| 630 | P15102 | GLNA4 | Glutamine synthetase leaf isozyme, chloroplastic | 16.4 | 1.52 | 0.3 | Amino acid metabolism |
| 631 | B5LMN4 | ACCD | Acetyl-coenzyme A carboxylase carboxyl transferase subunit β, chloroplastic | 227.5 | 1.52 | 0.1 | Fatty acid biosynthesis |
| 632 | Q9BBT4 | YCF3 | Photosystem I assembly protein Ycf3 | 4.4 | 1.54 | 0.4 | Photosynthesis |
| 633 | O49859 | C82A4 | Cytochrome P450 82A4 | 101.4 | 1.54 | 0.4 | Monooxygenase |
| 634 | P48631 | FD6E2 | ω-6 fatty acid desaturase, endoplasmic reticulum isozyme 2 | 39.7 | 1.55 | 0.3 | Fatty acid biosynthesis |
| 635 | Q6EJ97 | ISPS | Isoprene synthase, chloroplastic | 58.4 | 1.57 | 0.4 | Lipid metabolism |
| 636 | Q2PMQ0 | RR8 | 30S ribosomal protein S8, chloroplastic | 210.7 | 1.57 | 0.5 | Translation |
| 637 | Q9BBQ4 | RPOA | DNA-directed RNA polymerase subunit α | 173.8 | 1.57 | 0.4 | Transcription |
| 638 | Q9BBP9 | RK16 | 50S ribosomal protein L16, chloroplastic | 119.0 | 1.57 | 0.4 | Translation |
| 639 | P20178 | THS1 | Stilbene synthase 1 | 0.0 | 1.58 | 0.5 | Defense response |
| 640 | Q2PMN8 | NDHH | NAD(P)H-quinone oxidoreductase subunit H, cloroplastic | 15.9 | 1.58 | 0.2 | Electron transport |
| 641 | Q04708 | P5CR | Pyrroline-5-carboxylate reductase | 251.3 | 1.58 | 0.4 | Amino acid metabolism |
| 642 | P93472 | DIM | δ(24)-sterol reductase | 75.0 | 1.58 | 0.1 | Steroid biosynthesis |
| 643 | Q2HVD6 | MTA70 | Putative N6-adenosine-methyltransferase MT-70-like | 90.1 | 1.60 | 0.2 | Nucleotide metabolism |
| 644 | P08241 | RR2 | 30S ribosomal protein S2, chloroplastic | 85.7 | 1.60 | 0.1 | Translation |
| 645 | Q764T8 | LUPS | Lupeol synthase | 31.2 | 1.62 | 0.5 | Saponin biosynthesis |
| 646 | Q6RVV4 | TIC32 | Short-chain dehydrogenase TIC 32, chloroplastic | 153.9 | 1.62 | 0.1 | Redox homeostasis |
| 647 | P40620 | HMGL | HMG1/2-like protein | 137.4 | 1.63 | 0.2 | DNA binding |
| 648 | Q42920 | PME | Pectinesterase/pectinesterase inhibitor | 45.6 | 1.65 | 0.3 | Cell wall synthesis |
| 649 | P31239 | ACCO | 1-Aminocyclopropane-1-carboxylate oxidase | 178.9 | 1.65 | 0.5 | Hormone metabolism |
| 650 | A4GG85 | RRL16 | 50S ribosomal protein L16, chloroplastic | 31.5 | 1.65 | 0.3 | Translation |
| 651 | Q42823 | RBS | Ribulose bisphosphate carboxylase small chain, chloroplastic | 99.4 | 1.65 | 0.2 | Carbohydrate metabolism |
| 652 | O81972 | C82A2 | Cytochrome P450 82A2 | 43.0 | 1.65 | 0.2 | Monooxygenase |
| 653 | Q01289 | POR | Protochlorophyllide reductase, chloroplastic | 106.7 | 1.67 | 0.5 | Pigment Metabolism |
| 654 | O24305 | M3OM1 | (+)-6a-hydroxymaackiain 3-O-methyltransferase 1 | 74.7 | 1.67 | 0.1 | Secondary metabolism |
| 655 | P51069 | THS3 | Stilbene synthase 3 | 0.0 | 1.67 | 0.2 | Defense response |
| 656 | Q41059 | GLGB2 | 1,4-α-glucan-branching enzyme 1, chloroplastic/amyloplastic (Fragment) | 150.8 | 1.67 | 0.1 | Carbohydrate metabolism |
| 657 | P02580 | ACT3 | Actin-3 | 64.7 | 1.70 | 0.3 | Cytoskeleton |
| 658 | P25012 | CCNB2 | G2/ mitotic-specific cyclin S13-7 (Fragment) | 19.8 | 1.70 | 0.4 | Cell cycle |
| 659 | O48928 | C77A3 | Cytochrome P450 77A3 | 49.5 | 1.70 | 0.5 | Monooxygenase |
| 660 | P51080 | CHS | Chalcone synthase (Fragment) | 43.8 | 1.72 | 0.3 | Secondary metabolism |
| 661 | Q43822 | PLSB | Glycerol-3-phosphate acyltransferase, chloroplastic | 78.7 | 1.72 | 0.1 | Carbohydrate metabolism |
| 662 | O48902 | MDHP | Malate dehydrogenase [NADP], chloroplastic | 144.0 | 1.72 | 0.1 | Carbohydrate metabolism |
| 663 | Q4VY51 | SYM8 | Probable ion channel SYM8 | 26.3 | 1.73 | 0.5 | Transport |
| 664 | Q84XA3 | IMDH | Inosine-5'-monophosphate dehydrogenase | 53.8 | 1.73 | 0.5 | Nucleotide metabolism |
| 665 | P12858 | G3PA | Glyceraldehyde-3-phosphate dehydrogenase A, chloroplastic | 190.9 | 1.73 | 0.3 | Carbohydrate metabolism |
| 666 | P15792 | KPK1 | Protein kinase PVPK-1 | 34.9 | 1.75 | 0.1 | Signal transduction |
| 667 | O03376 | AOX3 | Alternative oxidase 3, mitochondrial | 12.4 | 1.77 | 0.4 | Electron transport |
| 668 | O65729 | RL18 | 60S ribosomal protein L18 (Fragment) | 90.5 | 1.77 | 0.5 | Translation |
| 669 | P93328 | NO16 | Early nodulin-16 | 36.9 | 1.80 | 0.5 | Nodulation |
| 670 | P30077 | CHS9 | Chalcone synthase 9 | 34.8 | 1.80 | 0.2 | Secondary metabolism |
| 671 | Q9SC88 | GCP4 | γ-Tubulin complex component 4 homolog | 89.1 | 1.82 | 0.2 | Cytoskeleton |
| 672 | Q29U70 | I4OMT | Isoflavone 4'-O-methyltransferase | 80.4 | 1.84 | 0.3 | Secondary metabolism |
| 673 | P07219 | PHSA | Phaseolin, α-type | 30.2 | 1.86 | 0.2 | Seed storage protein |
| 674 | P51081 | CHSA | Chalcone synthase 1A | 6.1 | 1.88 | 0.1 | Secondary metabolism |
| 675 | Q43070 | GALE1 | UDP-glucose 4-epimerase | 27.9 | 1.90 | 0.4 | Carbohydrate metabolism |
| 676 | P51078 | CHS5 | Chalcone synthase 4-2 | 34.8 | 1.90 | 0.4 | Secondary metabolism |
| 677 | P51086 | CHS4 | Chalcone synthase 4 (Fragment) | 34.8 | 1.92 | 0.5 | Secondary metabolism |
| 678 | P30075 | CHS4 | Chalcone synthase 4 | 34.8 | 1.92 | 0.1 | Secondary metabolism |
| 679 | P30076 | CHS8 | Chalcone synthase 8 | 34.8 | 1.93 | 0.5 | Secondary metabolism |
| 680 | B0M3E8 | UGE1 | Bifunctional UDP-glucose 4-epimerase and  UDP-xylose 4-epimerase 1 | 27.9 | 1.93 | 0.4 | Cell wall synthesis |
| 681 | O48559 | UNI | Protein UNIFOLIATA | 43.1 | 1.97 | 0.4 | DNA binding |
| 682 | P51085 | CHS3 | Chalcone synthase 3 | 34.8 | 1.97 | 0.3 | Secondary metabolism |
| 683 | O49858 | C82A3 | Cytochrome P450 82A3 | 33.2 | 1.97 | 0.1 | Monooxygenase |
| 684 | P19168 | CHS3 | Chalcone synthase 3 | 18.9 | 1.99 | 0.3 | Secondary metabolism |
| **Up-regulated proteins** | | | | | | | |
| 685 | P36875 | 2AAA | Protein phosphatase PP2A regulatory subunit A (Fragment) | 56.1 | 2.01 | 0.04 | Signal transduction |
| 686 | Q9BBN8 | NDHH | NAD(P)H-quinone oxidoreductase subunit H, chloroplastic | 15.9 | 2.05 | 0.05 | electron transport |
| 687 | P30074 | CHS2 | Chalcone synthase 2 | 34.8 | 2.05 | 0.01 | Secondary metabolism |
| 688 | Q01915 | ATPAM | ATP synthase subunit α, mitochondrial | 101.9 | 2.05 | 0.05 | Transport |
| 689 | O23883 | CHS3 | Chalcone synthase 3 | 34.8 | 2.10 | 0.01 | Secondary metabolism |
| 690 | P52575 | IFR | Isoflavone reductase | 66.2 | 2.10 | 0.05 | Secondary metabolism |
| 691 | O22307 | C71DB | Cytochrome P450 71D11 (Fragment) | 34.2 | 2.10 | 0.03 | Monooxygenase |
| 692 | P02856 | VCL1 | Vicilin, 14 kDa component | 39.5 | 2.12 | 0.03 | Seed storage protein |
| 693 | P49046 | LEGU | Legumain | 86.0 | 2.14 | 0.02 | Seed storage protein |
| 694 | P53393 | SUT3 | Low affinity sulfate transporter 3 | 66.9 | 2.16 | 0.01 | Transport |
| 695 | O82709 | CALX | Calnexin homolog | 157.8 | 2.16 | 0.04 | Chaperone |
| 696 | P51087 | CHS5 | Chalcone synthase 5 | 34.8 | 2.20 | 0.05 | Secondary metabolism |
| 697 | P51083 | CHS1 | Chalcone synthase 1 | 34.8 | 2.20 | 0.01 | Secondary metabolism |
| 698 | P51079 | CHS6-4 | Chalcone synthase 6-4 | 24.9 | 2.20 | 0.04 | Secondary metabolism |
| 699 | P51088 | CHS6 | Chalcone synthase 6 | 34.8 | 2.23 | 0.01 | Secondary metabolism |
| 700 | Q01287 | CHS2 | Chalcone synthase 2 | 34.8 | 2.25 | 0.05 | Secondary metabolism |
| 701 | Q43785 | GLNA3 | Glutamine synthetase nodule isozyme | 5.8 | 2.25 | 0.01 | Nodulation |
| 702 | O24326 | VPE2 | Vacuolar-processing enzyme | 41.3 | 2.27 | 0.03 | Seed storage protein |
| 703 | P30081 | CHS7 | Chalcone synthase 7 | 24.9 | 2.29 | 0.03 | Secondary metabolism |
| 704 | A4GGB2 | ATPA | ATP synthase subunit α, chloroplastic | 33.6 | 2.34 | 0.03 | electron transport |
| 705 | O22586 | CHSY | Chalcone synthase | 34.8 | 2.36 | 0.05 | Secondary metabolism |
| 706 | P20780 | ARA1 | Arachin 21 kDa protein | 161.3 | 2.51 | 0.02 | Seed storage protein |
| 707 | P21528 | MDHP | Malate dehydrogenase [NADP], chloroplastic | 54.1 | 2.56 | 0.01 | Carbohydrate metabolism |
| 708 | P46259 | TBA1 | Tubulin α-1 chain | 142.3 | 2.61 | 0.03 | Cell cycle |
| 709 | P28583 | TBA1 | Calcium-dependent protein kinase SK5 | 87.1 | 2.69 | 0.01 | Signal transduction |
| 710 | O48561 | CATA4 | Catalase-4 | 48.6 | 2.75 | 0.05 | Redox homeostasis |
| 711 | Q43068 | C82A1 | Cytochrome P450 82A1 (Fragment) | 20.8 | 2.86 | 0.03 | Monooxygenase |
| 712 | Q43093 | SSG2 | Granule-bound starch synthase 2, chloroplastic/ amyloplastic | 26.2 | 3.00 | 0.04 | Carbohydrate metabolism |
| 713 | A4PU48 | METK | S-Adenosylmethionine synthase | 19.2 | 3.10 | 0.04 | Amino acid metabolism |
| 714 | P49680 | IAA6 | Auxin-induced protein IAA6 | 28.1 | 3.19 | 0.03 | Growth and development |
| 715 | Q9M5Q1 | FUT1 | Galactoside 2-α-L-fucosyltransferase | 22.8 | 3.25 | 0.01 | Cell wall synthesis |
| 716 | P93484 | VSR1 | Vacuolar-sorting receptor 1 | 40.4 | 3.25 | 0.05 | Transport |
| 717 | Q2PMU9 | ATPE | ATP synthase ε chain, chloroplastic | 78.5 | 3.29 | 0.04 | electron transport |
| 718 | O81117 | C94A1 | Cytochrome P450 94A1 | 51.5 | 3.39 | 0.05 | Monooxygenase |
| 719 | O04300 | RGP1 | Probable UDP-arabinopyranose mutase 1 | 32.5 | 3.46 | 0.05 | Cell wall synthesis |
| 720 | P00965 | GLNA3 | Glutamine synthetase N-1 | 7.4 | 3.67 | 0.05 | Amino acid metabolism |
| 721 | P28590 | ABRC | Abrin-c OS | 160.2 | 3.74 | 0.04 | Defense response |
| 722 | P25700 | IT2 | Trypsin inhibitor 2 | 237.8 | 4.18 | 0.02 | Protease inhibitor |
| 723 | P51851 | PDC2 | Pyruvate decarboxylase 2 (Fragment) | 189.4 | 4.57 | 0.01 | Carbohydrate metabolism |
| 724 | P13917 | 7SB1 | Basic 7S globulin | 117.1 | 5.10 | 0.02 | Seed storage protein |
| 725 | Q06009 | PP2A | Serine/ threonine-protein phosphatase PP2A catalytic subunit | 32.0 | 6.55 | 0.03 | Signal transduction |
| 726 | O82134 | PCNA | Proliferating cell nuclear antigen | 129.0 | 7.17 | 0.02 | Replication |
| 727 | P81007 | ENT | Cytolytic protein enterolobin | 199.0 | 7.32 | 0.03 | Pathogenesis |
| 728 | O65743 | RL24 | 60S ribosomal protein L24 | 28.8 | 7.92 | 0.01 | Translation |
| 729 | Q06930 | ABR18 | ABA-responsive protein ABR18 | 23.6 | 8.41 | 0.03 | Growth and development |
| 730 | B5BSX1 | BAMO | β-Amyrin 11-oxidase | 17.9 | 8.85 | 0.01 | Saponin biosynthesis |
| 731 | P45456 | ACEA1 | Isocitrate lyase 1 (Fragment) | 53.2 | 12.55 | 0.05 | Carbohydrate metabolism |
| 732 | P05190 | LEGB4 | Legumin type B | 14.7 | 13.20 | 0.01 | Seed storage protein |
| 733 | P01070 | ITRA | Trypsin inhibitor A | 347.1 | 17.99 | 0.02 | Protease inhibitor |
| 734 | P05693 | LEGK | Legumin K (Fragment) | 79.0 | 19.89 | 0.04 | Seed storage protein |
| 735 | Q6PSC6 | MATK | Maturase K | 15.1 | 23.81 | 0.04 | mRNA processing |
| 736 | O48923 | C71DA | Cytochrome P450 71D10 | 35.9 | 26.31 | 0.01 | Monooxygenase |
| 737 | Q9TKS4 | MATK | Maturase K | 17.1 | 27.66 | 0.02 | mRNA processing |
| 738 | Q8LSN3 | FYPP | Phytochrome-associated serine/threonine-protein phosphatase | 192.3 | 31.50 | 0.05 | Signal transduction |
| 739 | P16080 | LEGB7 | Legumin type B (Fragment) | 14.7 | 41.68 | 0.03 | Seed storage protein |
| 740 | P16078 | LEGB2 | Legumin type B (Fragment) | 14.7 | 42.95 | 0.01 | Seed storage protein |
| 741 | P05692 | LEGJ | Legumin J | 52.9 | 43.38 | 0.01 | Seed storage protein |
| 742 | P16079 | LEGB6 | Legumin type B (Fragment) | 32.1 | 46.53 | 0.05 | Seed storage protein |
| **Control-specific proteins** | | | | | | | |
| 743 | Q53B75 | CF1B1 | Chalcone-flavonone isomerase 1β-1 | 233.8 | Control |  | Secondary metabolism |
| 744 | P51820 | DRTS | Bifunctional dihydrofolate reductase-thymidylate synthase | 57.0 | Control |  | Nucleotide metabolism |
| 745 | P83311 | IBB | Bowman-Birk type proteinase inhibitor | 103.9 | Control |  | Protease inhibitor |
| 746 | Q03467 | E13B | Glucan endo-1,3-β-glucosidase | 96.4 | Control |  | Cell wall synthesis |
| 747 | P52904 | ODPB | Pyruvate dehydrogenase E1 component  subunit β, mitochondrial | 70.6 | Control |  | Carbohydrate metabolism |
| 748 | P52902 | ODPA | Pyruvate dehydrogenase E1 component  subunit α, mitochondrial | 62.6 | Control |  | Carbohydrate metabolism |
| 749 | P83304 | LEC | Mannose/glucose-specific lectin (Fragment) | 4.1 | Control |  | Carbohydrate binding |
| 750 | O49046 | ARGI | Arginase | 48.7 | Control |  | Amino acid metabolism |
| 751 | P46298 | RS13 | 40S ribosomal protein S13 | 66.7 | Control |  | Translation |
| 752 | Q6WNQ8 | C81E8 | Cytochrome P450 81E8 | 88.5 | Control |  | Monooxygenase |
| 753 | Q8GTE3 | RS3A | 40S ribosomal protein S3a | 58.2 | Control |  | Translation |
| 754 | P31656 | CADH | Probable cinnamyl alcohol dehydrogenase | 22.2 | Control |  | Secondary metabolism |
| 755 | P46275 | F16P1 | Fructose-1,6-bisphosphatase, chloroplastic | 112.0 | Control |  | Carbohydrate metabolism |
| 756 | P32733 | ID5A | Kunitz-type trypsin inhibitor α chain | 80.5 | Control |  | Protease inhibitor |
| 757 | P46256 | ALF1 | Fructose-bisphosphate aldolase, cytoplasmic isozyme 1 | 70.8 | Control |  | Carbohydrate metabolism |
| 758 | P14749 | AGAL | α-Galactosidase | 62.0 | Control |  | Carbohydrate metabolism |
| 759 | P04670 | URIC1 | Uricase-2 isozyme 1 | 35.7 | Control |  | Nodulation |
| 760 | P81406 | GAPN | NADP-dependent glyceraldehyde-3-phosphate dehydrogenase | 114.4 | Control |  | Carbohydrate metabolism |
| 761 | Q2PMU5 | RR4 | 30S ribosomal protein S4, chloroplastic | 111.8 | Control |  | Translation |
| 762 | P53385 | HUTU | Urocanate hydratase | 45.3 | Control |  | Nodulation |
| 763 | P22973 | LEC2 | Anti-H(O) lectin 2 | 82.7 | Control |  | Carbohydrate binding |
| 764 | Q564G7 | GMGT1 | Galactomannan galactosyltransferase 1 | 60.2 | Control |  | Cell wall synthesis |
| 765 | P38661 | PDIA6 | Probable protein disulfide-isomerase A6 | 102.1 | Control |  | Redox homeostasis |
| 766 | Q2PMR0 | CLPP | ATP-dependent Clp protease proteolytic subunit | 1.6 | Control |  | Proteolysis |
| 767 | P13603 | ADH1 | Alcohol dehydrogenase 1 | 4.5 | Control |  | Catalytic activity |
| 768 | P81726 | ICI2 | Subtilisin inhibitor CLSI-II | 46.0 | Control |  | Defense response |
| 769 | Q2PMQ3 | RPOA | DNA-directed RNA polymerase subunit α | 224.3 | Control |  | Transcription |
| 770 | Q01516 | ALFC1 | Fructose-bisphosphate aldolase 1, chloroplastic (Fragment) | 38.3 | Control |  | Carbohydrate metabolism |
| 771 | C6T0L2 | SLE3 | Protein SLE3 | 18.2 | Control |  | Signal transduction |
| 772 | P19594 | 2SS | 2S albumin | 15.5 | Control |  | Seed storage protein |
| 773 | Q9ZST8 | TIC20 | Protein TIC 20, chloroplastic | 118.7 | Control |  | Transport |
| 774 | P41089 | CFI | Chalcone-flavonone isomerase | 119.0 | Control |  | Secondary metabolism |
| 775 | P46519 | LEA14 | Desiccation protectant protein Lea14 homolog | 47.5 | Control |  | Defense response |
| 776 | P85172 | IBB1 | Bowman-Birk type proteinase inhibitor | 69.2 | Control |  | Protease inhibitor |
| 777 | P55844 | RL14 | Probable 60S ribosomal protein L14 | 31.3 | Control |  | Translation |
| 778 | O65751 | RSSA | 40S ribosomal protein SA | 53.6 | Control |  | Translation |
| 779 | Q9XHC6 | C93E1 | β-Amyrin 24-hydroxylase | 88.8 | Control |  | Saponin biosynthesis |
| 780 | Q41219 | LEGRE | Leghemoglobin reductase | 31.0 | Control |  | Nodulation |
| 781 | Q5NE24 | NSP2 | Nodulation-signaling pathway 2 protein | 32.5 | Control |  | Nodulation |
| 782 | C6SXZ3 | CSPL8 | CASP-like protein 1D1 | 23.4 | Control |  | Cell wall synthesis |
| 783 | O65731 | RS5 | 40S ribosomal protein S5 (Fragment) | 47.7 | Control |  | Translation |
| 784 | P35694 | XTH2 | Xyloglucan endotransglucosylase/ hydrolase 2 | 14.0 | Control |  | Cell wall synthesis |
| 785 | Q8LNZ5 | XTHB | Probable xyloglucan endotransglucosylase/ hydrolase protein B | 8.9 | Control |  | Cell wall synthesis |
| 786 | Q07463 | PUR7 | Phosphoribosylaminoimidazole-succinocarboxamide synthase, chloroplastic (Fragment) | 58.7 | Control |  | Nucleotide metabolism |
| 787 | Q01807 | LEC2 | Truncated lectin 2 | 69.9 | Control |  | DNA binding |
| 788 | P47905 | RS27A | Ubiquitin-40S ribosomal protein S27a | 163.7 | Control |  | Signal transduction |
| 789 | P50346 | RLA0 | 60S acidic ribosomal protein P0 | 134.6 | Control |  | Translation |
| 790 | P05088 | PHAE | Erythroagglutinating phytohemagglutinin | 138.5 | Control |  | Carbohydrate binding |
| 791 | P05087 | PHAL | Leucoagglutinating phytohemagglutinin | 378.3 | Control |  | Defense response |
| 792 | Q9SM57 | OEP21 | Outer envelope pore protein 21, chloroplastic | 48.3 | Control |  | Transport |
| 793 | P93332 | NOD3 | Bidirectional sugar transporter N3 | 61.8 | Control |  | Transport |
| 794 | A0AAR7 | CCAMK | Calcium and calcium/calmodulin-dependent  serine/threonine-protein kinase | 107.1 | Control |  | Signal transduction |
| 795 | P17673 | RBS | Ribulose bisphosphate carboxylase small chain, chloroplastic | 38.1 | Control |  | Carbohydrate metabolism |
| 796 | P49163 | RK22 | 50S ribosomal protein L22, chloroplastic | 16.7 | Control |  | Translation |
| 797 | Q8MC99 | RPOA | DNA-directed RNA polymerase subunit α | 110.3 | Control |  | Transcription |
| 798 | P26987 | SAM22 | Stress-induced protein SAM22 | 129.0 | Control |  | Defense response |
| 799 | Q9BBU3 | PSBA | Photosystem II protein D1 | 26.7 | Control |  | Photosynthesis |
| 800 | P40590 | RL34 | 60S ribosomal protein L34 | 58.5 | Control |  | Translation |
| 801 | O24296 | GPX1 | Phospholipid hydroperoxide glutathione peroxidase, chloroplastic | 97.4 | Control |  | Redox homeostasis |
| 802 | P05046 | LEC | Lectin | 47.1 | Control |  | Carbohydrate binding |
| 803 | P05045 | LEC1 | Seed lectin subunit I | 73.6 | Control |  | Carbohydrate binding |
| 804 | Q9BBR1 | RR18 | 30S ribosomal protein S18, chloroplastic | 71.8 | Control |  | Translation |
| 805 | Q9XH46 | TATA | Sec-independent protein translocase protein TATA, chloroplastic | 316.3 | Control |  | Transport |
| 806 | Q6PSU2 | CONG7 | Conglutin-7 | 54.0 | Control |  | Seed storage protein |
| 807 | Q9BBQ1 | RR8 | 30S ribosomal protein S8, chloroplastic | 44.7 | Control |  | Translation |
| 808 | Q9SP37 | SAHH | Adenosylhomocysteinase | 98.5 | Control |  | Amino acid metabolism |
| 809 | P81371 | LECS | Seed lectin | 107.0 | Control |  | Carbohydrate binding |
| 810 | P13240 | DR206 | Disease resistance response protein 206 | 25.9 | Control |  | Secondary metabolism |
| 811 | Q9S9E3 | IBB | Horsegram inhibitor 1 | 140.8 | Control |  | Protease inhibitor |
| 812 | Q39527 | LECR | Lectin-related protein (Fragment) | 273.1 | Control |  | Carbohydrate binding |
| 813 | Q43064 | PYRB3 | Aspartate carbamoyltransferase 3, chloroplastic | 90.0 | Control |  | Amino acid metabolism |
| 814 | P17928 | CALM | Calmodulin | 248.4 | Control |  | Signal transduction |
| 815 | Q3LSN4 | LIAS1 | Lipoyl synthase 1, mitochondrial | 37.7 | Control |  | Carbohydrate metabolism |
| 816 | P31893 | OAT | Ornithine aminotransferase | 40.2 | Control |  | Amino acid metabolism |
| 817 | P25804 | CYSP | Cysteine proteinase 15A | 55.3 | Control |  | Proteolysis |
| 818 | Q96451 | 1433B | 14-3-3-like protein B (Fragment) | 186.9 | Control |  | Defense response |
| 819 | A4GGF3 | RR15 | 30S ribosomal protein S15, chloroplastic | 365.8 | Control |  | Translation |
| 820 | P12459 | TBB1 | Tubulin β-1 chain | 95.6 | Control |  | Cytoskeleton |
| 821 | A4GGE3 | NU5C | NAD(P)H-quinone oxidoreductase subunit 5,  chloroplastic | 13.0 | Control |  | Electron transport |
| 822 | Q4LDF9 | OEP37 | Outer envelope pore protein 37, chloroplastic | 25.9 | Control |  | Transport |
| 823 | P22503 | GUN | Endoglucanase | 137.8 | Control |  | Cell wall synthesis |
| 824 | P12786 | COX1 | Cytochrome c oxidase subunit 1 | 115.2 | Control |  | Electron transport |
| 825 | P42088 | LEC | Lectin OS | 124.2 | Control |  | Carbohydrate binding |
| 826 | P36361 | CHI5 | Endochitinase CH5B | 87.9 | Control |  | Defense response |
| 827 | O22308 | 7OMT6 | Isoflavone-7-O-methyltransferase 6 | 92.3 | Control |  | Secondary metabolism |
| 828 | P26291 | UCRIA | Cytochrome b6-f complex iron-sulfur subunit,  chloroplastic | 31.1 | Control |  | Electron transport |
| 829 | P09059 | SVF30 | Unknown seed protein 30.1 | 103.8 | Control |  | Seed storage protein |
| 830 | P42054 | VDAC | Outer plastidial membrane protein porin | 96.6 | Control |  | Transport |
| 831 | C6TBN2 | AKR1 | Probable aldo-keto reductase 1 | 68.9 | Control |  | Redox homeostasis |
| 832 | Q41112 | SRP | Stress-related protein | 66.1 | Control |  | Defense response |
| 833 | Q9ZS21 | LGUL | Lactoylglutathione lyase | 57.9 | Control |  | Secondary metabolism |
| 834 | Q2HU68 | H2A1 | Probable histone H2A.1 | 282.1 | Control |  | DNA binding |
| 835 | P0CC62 | NU2C1 | NAD(P)H-quinone oxidoreductase subunit 2 A,  chloroplastic | 151.7 | Control |  | Electron transport |
| 836 | Q40359 | ALFIN | PHD finger protein Alfin1 | 68.7 | Control |  | Transcription |
| 837 | P11964 | SODCP | Superoxide dismutase [Cu-Zn], chloroplastic | 62.6 | Control |  | Redox homeostasis |
| 838 | A4GG96 | YCF3 | Photosystem I assembly protein Ycf3 | 52.4 | Control |  | Photosynthesis |
| 839 | A4GG95 | RR4 | 30S ribosomal protein S4, chloroplastic | 41.0 | Control |  | Translation |
| 840 | P32291 | FAD3E | ω-3 fatty acid desaturase, endoplasmic reticulum | 140.8 | Control |  | Fatty acid biosynthesis |
| 841 | P16148 | PLZ12 | Protein PPLZ12 | 77.3 | Control |  | Cell cycle |
| 842 | P52419 | PUR1 | Amidophosphoribosyltransferase, chloroplastic (Fragment) | 118.3 | Control |  | Nucleotide metabolism |
| 843 | P26204 | BGLS | Non-cyanogenic β-glucosidase | 111.7 | Control |  | Carbohydrate metabolism |
| 844 | P26563 | AATM | Aspartate aminotransferase P2, mitochondrial (Fragment) | 22.4 | Control |  | Amino acid metabolism |
| 845 | P52766 | NDHK | NAD(P)H-quinone oxidoreductase subunit K, chloroplastic | 19.4 | Control |  | Electron transport |
| 846 | A2Q1V6 | ATG4 | Cysteine protease ATG4 | 37.5 | Control |  | Proteolysis |
| 847 | P16121 | HSP70 | Heat shock 70 kDa protein (Fragment) | 98.2 | Control |  | Chaperone |
| 848 | Q8H0G2 | CFI1 | Chalcone-flavonone isomerase 1 | 14.5 | Control |  | Secondary metabolism |
| 849 | P31175 | NDHK | NAD(P)H-quinone oxidoreductase subunit K,  chloroplastic | 41.9 | Control |  | Electron transport |
| 850 | P31174 | NDHJ | NAD(P)H-quinone oxidoreductase subunit J,  chloroplastic | 113.5 | Control |  | Electron transport |
| 851 | P22196 | PER2 | Cationic peroxidase 2 | 164.4 | Control |  | Cell wall synthesis |
| 852 | P10821 | IT1A | Trypsin inhibitor 1A | 38.0 | Control |  | Protease inhibitor |
| 853 | O50044 | KDSA | 2-dehydro-3-deoxyphosphooctonate aldolase | 113.4 | Control |  | Carbohydrate metabolism |
| 854 | Q9XG83 | G2OX | Gibberellin 2-β-dioxygenase | 15.7 | Control |  | Hormone metabolism |
| 855 | Q3LRV4 | NRL4B | Bifunctional nitrilase/ nitrile hydratase NIT4B | 18.8 | Control |  | Catalytic activity |
| 856 | Q8LPW2 | RT13 | Small ribosomal subunit protein S13, mitochondrial | 47.0 | Control |  | Translation |
| 857 | P22177 | PCNA | Proliferating cell nuclear antigen (Fragment) | 196.1 | Control |  | Replication |
| 858 | Q9LKG7 | UGPA | UTP-glucose-1-phosphate uridylyltransferase | 91.9 | Control |  | Carbohydrate metabolism |
| 859 | Q42800 | DAPA | 4-hydroxy-tetrahydrodipicolinate synthase, chloroplastic | 151.2 | Control |  | Amino acid metabolism |
| 860 | P23233 | NO16 | Nodulin-16 | 93.4 | Control |  | Nodulation |
| 861 | Q41701 | NO12 | Early nodulin-12 | 235.2 | Control |  | Nodulation |
| 862 | C0HJX1 | LECC1 | Mannose/ glucose-specific lectin | 48.3 | Control |  | Carbohydrate binding |
| 863 | Q39445 | TBB | Tubulin β chain | 179.5 | Control |  | Cytoskeleton |
| 864 | C0HJW7 | MANA | α-Mannosidase (Fragments) | 441.9 | Control |  | Carbohydrate metabolism |
| 865 | P49351 | FPPS1 | Farnesyl pyrophosphate synthase 1 | 17.0 | Control |  | Lipid metabolism |
| 866 | C0HK81 | LECA | Lectin | 118.7 | Control |  | Carbohydrate binding |
| 867 | P14226 | PSBO | Oxygen-evolving enhancer protein 1, chloroplastic | 92.3 | Control |  | Photosynthesis |
| 868 | Q03227 | COX3 | Cytochrome c oxidase subunit 3 | 201.3 | Control |  | Electron transport |
| 869 | P23558 | LEC1 | Lectin 1 | 162.1 | Control |  | Carbohydrate binding |
| 870 | P04149 | ARA5 | Arachin 25 kDa protein | 79.8 | Control |  | Seed storage protein |
| 871 | P04144 | NO23 | Nodulin-23 | 140.3 | Control |  | Nodulation |
| 872 | P23535 | E13B | Glucan endo-1,3-β-glucosidase, basic isoform | 111.4 | Control |  | Cell wall synthesis |
| 873 | P04122 | LECB | Lectin β-1 and β-2 chains | 365.8 | Control |  | Carbohydrate binding |
| 874 | P80463 | PHS1 | Phaseolin | 86.6 | Control |  | Seed storage protein |
| 875 | P24924 | ITRY | Trypsin inhibitor | 189.7 | Control |  | Protease inhibitor |
| 876 | B5LMS0 | NDHH | NAD(P)H-quinone oxidoreductase subunit H,  chloroplastic | 48.5 | Control |  | Electron transport |
| 877 | P48513 | TF2B | Transcription initiation factor IIB | 112.2 | Control |  | Transcription |
| 878 | P02240 | LGB2 | Leghemoglobin-2 | 92.4 | Control |  | Nodulation |
| 879 | Q9SQL2 | CB24 | Chlorophyll a-b binding protein P4, chloroplastic | 100.2 | Control |  | Photosynthesis |
| 880 | P39882 | NIA | Nitrate reductase [NADH] (Fragment) | 19.3 | Control |  | Nodulation |
| 881 | P02238 | LGBA | Leghemoglobin A | 75.3 | Control |  | Nodulation |
| 882 | P02233 | LGB1 | Leghemoglobin-1 | 110.3 | Control |  | Nodulation |
| 883 | P02232 | LGB1 | Leghemoglobin-1 | 25.0 | Control |  | Nodulation |
| 884 | Q6DW73 | DGDG2 | Digalactosyldiacylglycerol synthase 2, chloroplastic | 117.4 | Control |  | Cell wall synthesis |
| 885 | Q41015 | PIP21 | Kunitz-type trypsin inhibitor-like 1 protein | 17.0 | Control |  | Protease inhibitor |
| 886 | P56707 | SMTA | Selenocysteine methyltransferase | 28.6 | Control |  | Amino acid metabolism |
| 887 | O22518 | RSSA | 40S ribosomal protein SA | 59.7 | Control |  | Translation |
| 888 | Q35639 | NU5M | NADH-ubiquinone oxidoreductase chain 5 (Fragment) | 81.3 | Control |  | Electron transport |
| 889 | Q42799 | C93A2 | Cytochrome P450 93A2 | 49.4 | Control |  | Monooxygenase |
| 890 | Q42796 | F16P1 | Fructose-1,6-bisphosphatase, chloroplastic | 104.4 | Control |  | Carbohydrate metabolism |
| 891 | Q647H2 | AHY3 | Arachin Ahy-3 | 79.0 | Control |  | Seed storage protein |
| 892 | P93162 | CHLI | Magnesium-chelatase subunit ChlI, chloroplastic | 43.6 | Control |  | Photosynthesis |
| 893 | Q84V83 | LAR | Leucoanthocyanidin reductase | 63.9 | Control |  | Secondary metabolism |
| 894 | P02870 | LEC | Lectin | 192.3 | Control |  | Carbohydrate binding |
| 895 | P35450 | EFGC | Elongation factor G, chloroplastic (Fragment) | 30.5 | Control |  | Translation |
| 896 | Q43857 | INVA | Acid β-fructofuranosidase | 104.3 | Control |  | Carbohydrate metabolism |
| 897 | P53763 | URIC | Uricase-2 | 37.0 | Control |  | Nodulation |
| 898 | P28640 | DHN2 | Dehydrin DHN2 | 181.4 | Control |  | Defense response |
| 899 | Q9ZTR1 | SPD1 | Spermidine synthase 1 | 46.5 | Control |  | Amino acid metabolism |
| 900 | Q9ZTR0 | SPD2 | Spermidine synthase 2 | 58.5 | Control |  | Amino acid metabolism |
| 901 | P02866 | CONA | Concanavalin-A | 43.8 | Control |  | Carbohydrate metabolism |
| 902 | P28639 | DHN1 | Dehydrin DHN1 | 226.7 | Control |  | Defense response |
| 903 | P10743 | VSPB | Stem 31 kDa glycoprotein | 52.3 | Control |  | Seed storage protein |
| 904 | O81970 | C71A9 | Cytochrome P450 71A9 | 58.7 | Control |  | Monooxygenase |
| 905 | Q94G17 | TATC | Sec-independent protein translocase protein TATC, chloroplastic | 66.6 | Control |  | Transport |
| 906 | P83052 | BBKI | Kunitz-type serine Protease inhibitor BbKI | 219.6 | Control |  | Protease inhibitor |
| 907 | P83051 | BBCI | Kunitz-type proteinase inhibitor BbCI | 179.4 | Control |  | Protease inhibitor |
| 908 | Q43820 | DCAM | S-ASdenosylmethionine decarboxylase proenzyme | 65.7 | Control |  | Amino acid metabolism |
| 909 | Q41649 | FKB15 | FK506-binding protein 2 | 115.6 | Control |  | Defense response |
| 910 | Q41640 | RAB7 | Ras-related protein Rab7 | 121.2 | Control |  | Signal transduction |
| 911 | Q41638 | XTHA | Xyloglucan endotransglucosylase/hydrolase protein A | 23.0 | Control |  | Cell wall synthesis |
| 912 | P83036 | ITRY | Trypsin inhibitor | 158.6 | Control |  | Protease inhibitor |
| 913 | O48920 | ARF | ADP-ribosylation factor | 44.8 | Control |  | Transport |
| 914 | P07371 | CB22 | Chlorophyll a-b binding protein AB80, chloroplastic | 40.7 | Control |  | Photosynthesis |
| 915 | O24076 | GBLP | Guanine nucleotide-binding protein subunit β-like protein | 139.7 | Control |  | Signal transduction |
| 916 | P86993 | LECT | Lectin | 81.4 | Control |  | Carbohydrate binding |
| 917 | P32110 | GSTX6 | Probable glutathione S-transferase | 168.0 | Control |  | Redox homeostasis |
| 918 | P86624 | LECA | Lectin α chain | 111.2 | Control |  | Carbohydrate binding |
| 919 | P51850 | PDC1 | Pyruvate decarboxylase 1 | 104.2 | Control |  | Carbohydrate metabolism |
| 920 | Q9M4T8 | PSA5 | Proteasome subunit α type-5 | 13.8 | Control |  | Protein Modification |
| 921 | A2SY66 | VICHY | Vicianin hydrolase (Fragment) | 63.9 | Control |  | Carbohydrate metabolism |
| 922 | A7ISP6 | CFI3 | Chalcone-flavonone isomerase 3 | 6.8 | Control |  | Secondary metabolism |
| 923 | P06585 | PSBA | Photosystem II protein D1 | 19.6 | Control |  | Photosynthesis |
| 924 | C6SZ04 | CSPL3 | CASP-like protein 2C1 | 40.9 | Control |  | Cell wall synthesis |
| **Treated-specific proteins** | | | | | | | |
| 925 | P20150 | CEMA | Chloroplast envelope membrane protein | 86.1 | Treated |  | Transport |
| 926 | O82043 | ILV5 | Ketol-acid reductoisomerase, chloroplastic | 95.3 | Treated |  | Amino acid metabolism |
| 927 | Q9ZTA9 | FRIL | Flt3 receptor-interacting lectin | 107.0 | Treated |  | Carbohydrate binding |
| 928 | O24325 | VPE1 | Vacuolar-processing enzyme | 85.7 | Treated |  | Seed storage protein |
| 929 | P56331 | IF1A | Eukaryotic translation initiation factor 1α | 188.1 | Treated |  | Translation |
| 930 | Q2N2K1 | PHYK1 | Probable phytol kinase 1, chloroplastic | 131.7 | Treated |  | Pigment Metabolism |
| 931 | Q2N2K0 | PHYK3 | Probable phytol kinase 3, chloroplastic | 23.3 | Treated |  | Pigment Metabolism |
| 932 | P46266 | 1433 | 14-3-3-like protein | 167.2 | Treated |  | Defense response |
| 933 | A0A067YBQ3 | UGT13 | UDP-glycosyltransferase 13 | 66.0 | Treated |  | Carbohydrate metabolism |
| 934 | P15838 | LEGA2 | Legumin A2 | 6.2 | Treated |  | Seed storage protein |
| 935 | P01066 | IBB1 | Bowman-Birk type proteinase inhibitor A-II | 258.1 | Treated |  | Protease inhibitor |
| 936 | P01057 | IBB3 | Bowman-Birk type proteinase inhibitor DE-3 | 188.0 | Treated |  | Protease inhibitor |
| 937 | Q94IC4 | FRI2 | Ferritin-2, chloroplastic | 25.4 | Treated |  | Photosynthesis |
| 938 | Q1S9I9 | H2B1 | Probable histone H2B.1 | 33.5 | Treated |  | DNA binding |
| 939 | K7LFJ0 | PP890 | Protein PROPEP890 | 587.9 | Treated |  | Cell cycle |
| 940 | Q42372 | LCB2 | Bark agglutinin I polypeptide β | 61.6 | Treated |  | Carbohydrate binding |
| 941 | Q2QKL5 | PCS3 | Glutathione γ-glutamylcysteinyltransferase 3 | 78.7 | Treated |  | Hormone metabolism |
| 942 | Q40193 | RB11C | Ras-related protein Rab11C | 225.8 | Treated |  | Signal transduction |
| 943 | Q40191 | RB11A | Ras-related protein Rab11A | 142.2 | Treated |  | Signal transduction |
| 944 | Q07176 | MMK1 | Mitogen-activated protein kinase homolog MMK1 | 46.5 | Treated |  | Signal transduction |
| 945 | P05718 | CYB | Cytochrome b | 85.0 | Treated |  | Electron transport |
| 946 | Q2PMQ5 | CYB6 | Cytochrome b6 | 21.7 | Treated |  | Electron transport |
| 947 | Q06076 | ABRD | Abrin-d | 38.4 | Treated |  | Defense response |
| 948 | O65781 | GALE2 | UDP-glucose 4-epimerase GEPI48 | 99.9 | Treated |  | Carbohydrate metabolism |
| 949 | A0A161AT60 | NLTP1 | Non-specific lipid-transfer protein 1 | 1034.5 | Treated |  | Cell wall synthesis |
| 950 | P10322 | NO25 | Nodulin-25 | 140.8 | Treated |  | Nodulation |
| 951 | Q2PMN2 | NU4C | NAD(P)H-quinone oxidoreductase chain 4, chloroplastic | 110.5 | Treated |  | Electron transport |
| 952 | P52581 | IFRH | Isoflavone reductase homolog | 60.1 | Treated |  | Secondary metabolism |
| 953 | Q2PMM9 | NU5C | NAD(P)H-quinone oxidoreductase subunit 5, chloroplastic | 101.3 | Treated |  | Electron transport |
| 954 | P25273 | KTI2 | Kunitz-type trypsin inhibitor KTI2 | 190.7 | Treated |  | Protease inhibitor |
| 955 | B1B5P4 | N8DT2 | Naringenin 8-dimethylallyltransferase 2, chloroplastic | 20.2 | Treated |  | Secondary metabolism |
| 956 | P47923 | NDK2 | Nucleoside diphosphate kinase 2, chloroplastic | 25.7 | Treated |  | Nucleotide metabolism |
| 957 | Q9FT25 | PDX1 | Pyridoxal 5'-phosphate synthase subunit PDX1 | 18.6 | Treated |  | Amino acid metabolism |
| 958 | O49929 | OEP24 | Outer envelope pore protein 24, chloroplastic | 156.9 | Treated |  | Transport |
| 959 | P24146 | LEC4 | Lectin-4 | 123.7 | Treated |  | Carbohydrate binding |
| 960 | P50345 | RLA0 | 60S acidic ribosomal protein P0 | 63.9 | Treated |  | Translation |
| 961 | P51428 | RT10 | Ribosomal protein S10, mitochondrial | 57.1 | Treated |  | Translation |
| 962 | P58155 | CEMA | Chloroplast envelope membrane protein | 74.3 | Treated |  | Transport |
| 963 | P86893 | LECS | Seed lectin | 16.9 | Treated |  | Carbohydrate binding |
| 964 | P62929 | ALB1D | Albumin-1 D | 48.4 | Treated |  | Seed storage protein |
| 965 | P49158 | ACCD | Acetyl-coenzyme A carboxylase carboxyl transferase subunit β, chloroplastic | 111.3 | Treated |  | Fatty acid biosynthesis |
| 966 | P00865 | RBS1 | Ribulose bisphosphate carboxylase small chain 1, chloroplastic | 188.6 | Treated |  | Carbohydrate metabolism |
| 967 | Q9BBR0 | RK20 | 50S ribosomal protein L20, chloroplastic | 176.0 | Treated |  | Translation |
| 968 | Q9BBQ9 | CLPP | ATP-dependent Clp protease proteolytic subunit | 15.8 | Treated |  | Proteolysis |
| 969 | Q43086 | PYRB1 | Aspartate carbamoyltransferase 1, chloroplastic | 151.8 | Treated |  | Amino acid metabolism |
| 970 | Q43075 | SPE1 | Arginine decarboxylase | 56.6 | Treated |  | Amino acid metabolism |
| 971 | Q43072 | HIS7 | Imidazoleglycerol-phosphate dehydratase | 70.2 | Treated |  | Nucleotide metabolism |
| 972 | Q39528 | LEC1 | Agglutinin-1 | 48.1 | Treated |  | Carbohydrate binding |
| 973 | H1A988 | C7254 | 11-oxo-β-amyrin 30-oxidase | 80.4 | Treated |  | Saponin biosynthesis |
| 974 | P08927 | RUBB | RuBisCO large subunit-binding protein subunit β, chloroplastic | 82.7 | Treated |  | Carbohydrate metabolism |
| 975 | O24542 | AX22D | Auxin-induced protein 22D | 158.2 | Treated |  | Growth and development |
| 976 | O24541 | AX22C | Auxin-induced protein 22C | 115.7 | Treated |  | Growth and development |
| 977 | P29257 | LEC2 | 2-Acetamido-2-deoxy-D-galactose-binding seed lectin 2 | 41.6 | Treated |  | Carbohydrate binding |
| 978 | A4GGF2 | NDHH | NAD(P)H-quinone oxidoreductase subunit H, chloroplastic | 86.4 | Treated |  | Electron transport |
| 979 | A4GGE6 | NU4C | NAD(P)H-quinone oxidoreductase chain 4, chloroplastic | 25.4 | Treated |  | Electron transport |
| 980 | P80572 | ADHX | Alcohol dehydrogenase class-3 | 41.5 | Treated |  | Catalytic activity |
| 981 | Q39818 | HS22M | Heat shock 22 kDa protein, mitochondrial | 127.7 | Treated |  | Chaperone |
| 982 | P39657 | RUAP | RuBisCO-associated protein | 62.0 | Treated |  | Carbohydrate metabolism |
| 983 | P48630 | FD6E1 | ω-6 fatty acid desaturase,endoplasmic reticulum isozyme 1 | 160.6 | Treated |  | Fatty acid biosynthesis |
| 984 | P27047 | DRR4 | Disease resistance response protein DRRG49-C | 45.1 | Treated |  | Secondary metabolism |
| 985 | A4GGB8 | CEMA | Chloroplast envelope membrane protein | 80.1 | Treated |  | Transport |
| 986 | Q41161 | LCS2 | Seed agglutinin 2 | 61.7 | Treated |  | Seed storage protein |
| 987 | Q41160 | LCB3 | Putative bark agglutinin LECRPA3 (Fragment) | 213.1 | Treated |  | Defense response |
| 988 | Q01417 | PM1 | 18 kDa seed maturation protein | 72.4 | Treated |  | Growth and development |
| 989 | Q03943 | IM30 | Membrane-associated 30 kDa protein, chloroplastic | 264.8 | Treated |  | Transport |
| 990 | Q41159 | LCB1 | Bark agglutinin I polypeptide A | 516.7 | Treated |  | Carbohydrate binding |
| 991 | Q9FRT8 | ALB1 | Albumin-1 (Fragment) | 60.5 | Treated |  | Seed storage protein |
| 992 | Q8H0P8 | RBP1 | RNA-binding protein 1 | 14.4 | Treated |  | mRNA processing |
| 993 | Q944T2 | TCTP | Translationally-controlled tumor protein homolog | 159.8 | Treated |  | Cell cycle |
| 994 | P0CC88 | NU2C1 | NAD(P)H-quinone oxidoreductase subunit 2 A, chloroplastic | 118.5 | Treated |  | Electron transport |
| 995 | Q1SU99 | H2B3 | Probable histone H2B.3 | 33.5 | Treated |  | DNA binding |
| 996 | Q8RW99 | F16P2 | Fructose-1,6-bisphosphatase, cytosolic | 10.5 | Treated |  | Carbohydrate metabolism |
| 997 | Q9SXS3 | C93C2 | 2-hydroxy-isoflavanone synthase | 45.6 | Treated |  | Secondary metabolism |
| 998 | P50288 | ASPG | Isoaspartyl peptidase/L-asparaginase | 136.5 | Treated |  | Amino acid metabolism |
| 999 | P29501 | TBB2 | Tubulin β-2 chain (Fragment) | 95.0 | Treated |  | Cytoskeleton |
| 1000 | A4GG99 | RR14 | 30S ribosomal protein S14, chloroplastic | 100.9 | Treated |  | Translation |
| 1001 | C6T2J5 | CSPL4 | CASP-like protein 2D1 | 44.2 | Treated |  | Cell wall synthesis |
| 1002 | P45621 | GSA | Glutamate-1-semialdehyde 2,1-aminomutase,  chloroplastic | 129.7 | Treated |  | Amino acid metabolism |
| 1003 | Q40345 | IDHP | Isocitrate dehydrogenase [NADP], chloroplastic (Fragment) | 176.1 | Treated |  | Carbohydrate metabolism |
| 1004 | P52424 | PUR5 | Phosphoribosylformylglycinamidine cyclo-ligase,  chloroplastic/ mitochondrial | 64.6 | Treated |  | Nucleotide metabolism |
| 1005 | E4NKF8 | PUB1 | U-box domain-containing protein 1 | 43.1 | Treated |  | Nodulation |
| 1006 | E3SXU4 | BHLHW | Basic helix-loop-helix protein A | 149.0 | Treated |  | Secondary metabolism |
| 1007 | P49045 | VPE | Vacuolar-processing enzyme | 85.8 | Treated |  | Seed storage protein |
| 1008 | Q42822 | RBS | Ribulose bisphosphate carboxylase small chain,  chloroplastic | 34.4 | Treated |  | Carbohydrate metabolism |
| 1009 | Q05462 | RL27 | 60S ribosomal protein L27 | 273.9 | Treated |  | Translation |
| 1010 | P25795 | AL7A1 | Aldehyde dehydrogenase family 7 member A1 | 21.2 | Treated |  | Catalytic activity |
| 1011 | P68430 | H32 | Histone H3.2 | 106.0 | Treated |  | DNA binding |
| 1012 | P42653 | 1433A | 14-3-3-like protein A | 165.0 | Treated |  | Defense response |
| 1013 | P68427 | H32 | Histone H3.2 | 106.0 | Treated |  | DNA binding |
| 1014 | A0AT30 | NLTP3 | Non-specific lipid-transfer protein 3 | 764.0 | Treated |  | Cell wall synthesis |
| 1015 | Q40977 | MDAR | Monodehydroascorbate reductase | 68.2 | Treated |  | Redox homeostasis |
| 1016 | Q9FEL7 | LAX2 | Auxin transporter-like protein 2 | 148.3 | Treated |  | Growth and development |
| 1017 | Q9FEL6 | LAX3 | Auxin transporter-like protein 3 | 108.4 | Treated |  | Growth and development |
| 1018 | U3THC0 | FG2KI | Inactive UDP-glycosyltransferase 79A6 | 49.1 | Treated |  | Carbohydrate metabolism |
| 1019 | Q9FY06 | PPF1 | Inner membrane protein PPF-1, chloroplastic | 67.4 | Treated |  | Transport |
| 1020 | C6SZP8 | CSPL5 | CASP-like protein 1E2 | 25.7 | Treated |  | Cell wall synthesis |
| 1021 | P30364 | ASPG | Isoaspartyl peptidase/L-asparaginase | 159.8 | Treated |  | Amino acid metabolism |
| 1022 | P30362 | ASPG | Isoaspartyl peptidase/L-asparaginase (Fragment) | 30.6 | Treated |  | Amino acid metabolism |
| 1023 | K7K424 | DAT2D | Diacylglycerol O-acyltransferase 2D | 47.7 | Treated |  | Lipid metabolism |
| 1024 | Q04655 | YMA6 | Uncharacterized mitochondrial protein ORF154 | 107.3 | Treated |  | Uncharacterized |
| 1025 | P15958 | NU5C | NAD(P)H-quinone oxidoreductase subunit 5,  chloroplastic | 68.5 | Treated |  | Electron transport |
| 1026 | P48534 | APX1 | L-Ascorbate peroxidase, cytosolic | 53.3 | Treated |  | Redox homeostasis |
| 1027 | P04793 | HSP13 | 17.5 kDa class I heat shock protein | 155.2 | Treated |  | Chaperone |
| 1028 | P14848 | LGB2 | Leghemoglobin 2 | 413.5 | Treated |  | Nodulation |
| 1029 | Q09134 | GRPA | Abscisic acid and environmental stress-inducible protein | 26.8 | Treated |  | Defense response |
| 1030 | Q09131 | PPAF | Purple acid phosphatase | 57.8 | Treated |  | Signal transduction |
| 1031 | Q41050 | OEP16 | Outer envelope pore protein 16, chloroplastic | 111.2 | Treated |  | Transport |
| 1032 | P28011 | AAT1 | Aspartate aminotransferase 1 | 52.3 | Treated |  | Amino acid metabolism |
| 1033 | P28010 | LGB4 | Leghemoglobin | 174.5 | Treated |  | Nodulation |
| 1034 | P02236 | LGB2 | Leghemoglobin C2 | 35.4 | Treated |  | Nodulation |
| 1035 | Q6DW75 | DGDG2 | Digalactosyldiacylglycerol synthase 2, chloroplastic | 18.3 | Treated |  | Cell wall synthesis |
| 1036 | P25096 | P21 | Protein P21 | 12.8 | Treated |  | Cell cycle |
| 1037 | Q43560 | PR1 | Class-10 pathogenesis-related protein 1 | 212.1 | Treated |  | Pathogenesis |
| 1038 | P52389 | CDC2 | Cell division control protein 2 homolog | 80.9 | Treated |  | Growth and development |
| 1039 | Q06197 | IDHC | Isocitrate dehydrogenase [NADP] | 71.9 | Treated |  | Carbohydrate metabolism |
| 1040 | P11894 | RK9 | 50S ribosomal protein L9, chloroplastic | 210.0 | Treated |  | Translation |
| 1041 | P11893 | RK24 | 50S ribosomal protein L24, chloroplastic | 52.5 | Treated |  | Translation |
| 1042 | Q6UD73 | LYK3 | LysM domain receptor-like kinase 3 | 69.3 | Treated |  | Defense response |
| 1043 | Q41009 | TOC34 | Translocase of chloroplast 34 | 169.3 | Treated |  | Transport |
| 1044 | Q41005 | CBPX | Serine carboxypeptidase-like (Fragment) | 29.2 | Treated |  | Secondary metabolism |
| 1045 | Q35638 | RHO1 | Rac-like GTP-binding protein RHO1 | 87.0 | Treated |  | Signal transduction |
| 1046 | O65874 | MTF1 | MADS-box transcription factor 1 | 91.8 | Treated |  | DNA binding |
| 1047 | P19329 | ARC1 | Arcelin-1 | 152.9 | Treated |  | Seed storage protein |
| 1048 | P16064 | ICI1 | Subtilisin inhibitor 1 | 860.5 | Treated |  | Defense response |
| 1049 | P16059 | PSBP | Oxygen-evolving enhancer protein 2, chloroplastic | 68.6 | Treated |  | Photosynthesis |
| 1050 | P02853 | PHSB | Phaseolin, β-type | 18.0 | Treated |  | Seed storage protein |
| 1051 | O81974 | C71D8 | Cytochrome P450 71D8 | 11.2 | Treated |  | Monooxygenase |
| 1052 | Q41669 | MT1A | Metallothionein-like protein 1A | 76.7 | Treated |  | Defense response |
| 1053 | A6XNC6 | UGFGT | Flavonoid 3-O-glucosyltransferaseαα | 129.6 | Treated |  | Secondary metabolism |
| 1054 | P27880 | HSP12 | 18.2 kDa class I heat shock protein | 206.4 | Treated |  | Chaperone |
| 1055 | Q43814 | OTC | Ornithine carbamoyltransferase, chloroplastic | 139.6 | Treated |  | Amino acid metabolism |
| 1056 | P16002 | PLAS | Plastocyanin, chloroplastic | 26.7 | Treated |  | Photosynthesis |
| 1057 | Q8W171 | CYP1 | Peptidyl-prolyl cis-trans isomerase 1 | 127.7 | Treated |  | Protein modification |
| 1058 | Q06445 | CYTI | Cysteine proteinase inhibitor | 103.3 | Treated |  | Protease inhibitor |
| 1059 | I1LCI8 | FG2KO | UDP-glycosyltransferase 79A6 | 49.1 | Treated |  | Carbohydrate metabolism |
| 1060 | Q9SWF9 | ZFNL | Zinc finger CCCH domain-containing protein ZFN-like | 29.8 | Treated |  | DNA binding |
| 1061 | P49252 | AMO | Primary amine oxidase (Fragment) | 66.0 | Treated |  | Hormone metabolism |
| 1062 | P25985 | PR1 | Pathogenesis-related protein 1 | 118.3 | Treated |  | Pathogenesis |

| **S.No** | **Accession** | **Abbreviation** | **Description** | **PLGS**  **Score** | **Fold change** | **No_ of_peptides  (Control)** | **No_ of_peptides  (Treated)** | **STDEV (Control)** | **STDEV (Treated)** | **P value** |
| --- | --- | --- | --- | --- | --- | --- | --- | --- | --- | --- |
| **Down-regulated proteins** | | | | | | | | | | |
| 1 | Q41116 | AR5B | **Arcelin-5B** | 118.8 | 0.003 | 5.0 | 4.0 | 73.4 | 248.5 | 0.03 |
| 2 | Q42460 | AR5A | **Arcelin-5A** | 77.9 | 0.004 | 4.0 | 6.0 | 208.2 | 281.4 | 0.04 |
| 3 | Q8MCA4 | RK16 | **50S ribosomal protein L16; chloroplastic** | 53.0 | 0.030 | 10.0 | 5.0 | 152.8 | 46.1 | 0.04 |
| 4 | P28002 | COMT1 | **Caffeic acid 3-O-methyltransferase** | 55.5 | 0.042 | 10.0 | 2.0 | 115.5 | 113.2 | 0.01 |
| 5 | Q5D1B9 | MATK | **Maturase K** | 16.9 | 0.045 | 11.0 | 14.0 | 152.8 | 152.8 | 0.01 |
| 6 | P48724 | IF5 | **Eukaryotic translation initiation factor 5** | 57.6 | 0.046 | 16.0 | 16.0 | 15.3 | 152.8 | 0.02 |
| 7 | Q85XY8 | MATK | **Maturase K** | 29.1 | 0.046 | 3.0 | 10.0 | 549.8 | 20.0 | 0.05 |
| 8 | P02237 | LGC3 | **Leghemoglobin C3** | 8.3 | 0.093 | 4.0 | 6.0 | 10.0 | 552.7 | 0.02 |
| 9 | O24035 | PANC | **Pantoate-beta-alanine ligase** | 28.4 | 0.156 | 6.0 | 8.0 | 329.7 | 25.7 | 0.04 |
| 10 | D4AEP7 | ALB2 | **Albumin-2** | 115.3 | 0.157 | 6.0 | 6.0 | 11.5 | 112.1 | 0.04 |
| 11 | Q2PMP8 | RK16 | **50S ribosomal protein L16, chloroplastic** | 53.0 | 0.159 | 5.0 | 5.0 | 463.1 | 416.3 | 0.03 |
| 12 | P46417 | GSTX3 | **Glutathione S-transferase 3** | 26.8 | 0.176 | 6.0 | 6.0 | 73.4 | 93.2 | 0.01 |
| 13 | P26585 | HMGL | **HMG1/2-like protein** | 73.7 | 0.177 | 4.0 | 13.0 | 40.4 | 642.9 | 0.02 |
| 14 | Q8LP17 | CCD1 | **Carotenoid 9,10(9',10')-cleavage dioxygenase 1** | 110.2 | 0.188 | 12.0 | 9.0 | 10.0 | 73.9 | 0.05 |
| 15 | O23884 | CHS5 | **Chalcone synthase 5** | 34.8 | 0.208 | 11.0 | 4.0 | 230.0 | 144.2 | 0.02 |
| 16 | Q10370 | HMGYB | **HMG-Y-related protein B (Fragment)** | 377.2 | 0.208 | 6.0 | 2.0 | 92.4 | 112.7 | 0.03 |
| 17 | O23882 | CHS4 | **Chalcone synthase 4** | 34.8 | 0.212 | 7.0 | 14.0 | 10.0 | 113.8 | 0.01 |
| 18 | P93164 | GGH | **Gamma-glutamyl hydrolase** | 71.9 | 0.214 | 3.0 | 10.0 | 10.0 | 78.5 | 0.02 |
| 19 | P02235 | LGC1 | **Leghemoglobin C1** | 8.3 | 0.221 | 2.0 | 7.0 | 246.3 | 767.1 | 0.01 |
| 20 | P51084 | CHS2 | **Chalcone synthase 2** | 34.8 | 0.223 | 7.0 | 9.0 | 59.1 | 111.3 | 0.05 |
| 21 | P02871 | LEC | **Favin** | 213.3 | 0.223 | 3.0 | 3.0 | 10.0 | 142.4 | 0.03 |
| 22 | P51077 | CHS4-1 | **Chalcone synthase 4-1** | 34.8 | 0.225 | 2.0 | 8.0 | 35.6 | 58.0 | 0.04 |
| 23 | P49440 | CHSY | **Chalcone synthase 17** | 28.3 | 0.230 | 12.0 | 10.0 | 132.8 | 67.5 | 0.01 |
| 24 | O81971 | C71D9 | **Cytochrome P450 71D9** | 22.5 | 0.232 | 5.0 | 8.0 | 10.0 | 100.0 | 0.01 |
| 25 | Q9SML4 | CHS1 | **Chalcone synthase 1** | 60.6 | 0.237 | 9.0 | 7.0 | 10.0 | 395.0 | 0.03 |
| 26 | P18663 | RK2A | **50S ribosomal protein L2-A** | 74.3 | 0.237 | 15.0 | 6.0 | 52.2 | 94.6 | 0.01 |
| 27 | P30073 | CHS1 | **Chalcone synthase 1** | 34.8 | 0.244 | 10.0 | 17.0 | 152.8 | 152.8 | 0.05 |
| 28 | Q00423 | HMGYA | **HMG-Y-related protein A** | 365.7 | 0.267 | 7.0 | 4.0 | 10.0 | 378.6 | 0.02 |
| 29 | P52417 | GLGS2 | **Glucose-1-phosphate adenylyltransferase small subunit 2** | 51.7 | 0.267 | 16.0 | 14.0 | 10.0 | 493.3 | 0.05 |
| 30 | Q9B1H9 | RK2 | **50S ribosomal protein L2** | 74.3 | 0.270 | 10.0 | 7.0 | 10.0 | 158.8 | 0.01 |
| 31 | A4GGF8 | RK2 | **50S ribosomal protein L2** | 135.7 | 0.273 | 12.0 | 6.0 | 751.0 | 233.8 | 0.02 |
| 32 | Q8LVH2 | RK2 | **50S ribosomal protein L2** | 143.8 | 0.278 | 11.0 | 10.0 | 34.6 | 173.3 | 0.03 |
| 33 | O48905 | MDHC | **Malate dehydrogenase, cytoplasmic** | 45.8 | 0.292 | 10.0 | 9.0 | 10.0 | 240.1 | 0.03 |
| 34 | Q01286 | CHS1 | **Chalcone synthase 1** | 34.8 | 0.298 | 13.0 | 12.0 | 17.3 | 22.5 | 0.05 |
| 35 | P01071 | ITRB | **Trypsin inhibitor B** | 97.0 | 0.304 | 5.0 | 3.0 | 254.0 | 176.3 | 0.05 |
| 36 | Q96453 | 1433D | **14-3-3-like protein D** | 62.2 | 0.307 | 9.0 | 7.0 | 100.0 | 51.3 | 0.01 |
| 37 | P51089 | CHSY | **Chalcone synthase** | 28.3 | 0.313 | 13.0 | 10.0 | 15.3 | 110.5 | 0.01 |
| 38 | O62964 | RBL | **Ribulose bisphosphate carboxylase large chain** | 6.8 | 0.317 | 6.0 | 3.0 | 10.0 | 10.0 | 0.04 |
| 39 | A7VLV2 | non-SGRW | **Non-functional protein STAY-GREEN** | 126.5 | 0.326 | 7.0 | 8.0 | 10.0 | 16.3 | 0.03 |
| 40 | Q2PMV0 | ATPB | **ATP synthase subunit beta, chloroplastic** | 74.0 | 0.330 | 5.0 | 5.0 | 144.2 | 20.8 | 0.04 |
| 41 | O20304 | RBL | **Ribulose bisphosphate carboxylase large chain (Fragment)** | 41.2 | 0.333 | 6.0 | 10.0 | 60.1 | 14.5 | 0.01 |
| 42 | P42654 | 1433B | **14-3-3-like protein B** | 59.6 | 0.340 | 8.0 | 12.0 | 43.6 | 76.4 | 0.03 |
| 43 | A7VLV1 | SGRW | **Protein STAY-GREEN, chloroplastic** | 126.5 | 0.353 | 7.0 | 5.0 | 10.0 | 153.4 | 0.05 |
| 44 | D4Q9Z5 | SGT3 | **Soyasaponin III rhamnosyltransferase** | 13.3 | 0.353 | 7.0 | 7.0 | 202.0 | 56.1 | 0.04 |
| 45 | Q96452 | 1433C | **14-3-3-like protein C** | 88.0 | 0.357 | 9.0 | 6.0 | 219.2 | 138.5 | 0.03 |
| 46 | P93149 | C93B1 | **Licodione synthase** | 29.1 | 0.361 | 11.0 | 12.0 | 452.3 | 141.9 | 0.02 |
| 47 | P52576 | IFR | **Isoflavone reductase** | 69.3 | 0.372 | 7.0 | 7.0 | 240.6 | 54.6 | 0.04 |
| 48 | B5WWZ9 | FAO2 | **Long-chain-alcohol oxidase FAO2** | 95.6 | 0.379 | 18.0 | 15.0 | 455.2 | 85.4 | 0.02 |
| 49 | Q9SQ80 | G2OX1 | **Gibberellin 2-beta-dioxygenase 1** | 103.9 | 0.387 | 7.0 | 6.0 | 257.8 | 426.6 | 0.04 |
| 50 | Q39836 | GBLP | **Guanine nucleotide-binding protein subunit beta-like protein** | 139.7 | 0.403 | 8.0 | 4.0 | 284.2 | 60.9 | 0.01 |
| 51 | A4GG89 | RBL | **Ribulose bisphosphate carboxylase large chain** | 32.5 | 0.403 | 6.0 | 6.0 | 60.8 | 25.5 | 0.05 |
| 52 | Q42783 | BCCP | **Biotin carboxyl carrier protein of acetyl-CoA carboxylase,  chloroplastic** | 79.7 | 0.407 | 2.0 | 5.0 | 10.0 | 49.2 | 0.04 |
| 53 | P28552 | ATPG | **ATP synthase gamma chain, chloroplastic** | 24.2 | 0.411 | 12.0 | 9.0 | 20.0 | 20.5 | 0.05 |
| 54 | Q2PMM3 | RK2B | **50S ribosomal protein L2-B, chloroplastic** | 75.8 | 0.411 | 11.0 | 7.0 | 42.1 | 184.7 | 0.04 |
| 55 | O62943 | RBL | **Ribulose bisphosphate carboxylase large chain** | 56.8 | 0.419 | 7.0 | 3.0 | 138.0 | 32.1 | 0.04 |
| 56 | P62163 | CALM2 | **Calmodulin-2** | 33.6 | 0.423 | 2.0 | 2.0 | 10.0 | 30.6 | 0.03 |
| 57 | Q43111 | PME3 | **Pectinesterase 3** | 64.7 | 0.427 | 13.0 | 15.0 | 15.3 | 378.6 | 0.04 |
| 58 | Q8W3P8 | AOG | **Abscisate beta-glucosyltransferase** | 214.1 | 0.436 | 6.0 | 9.0 | 15.3 | 100.6 | 0.02 |
| 59 | Q9LRH8 | BAMS | **Beta-amyrin synthase** | 38.7 | 0.436 | 15.0 | 12.0 | 10.0 | 67.7 | 0.04 |
| 60 | P19143 | PAL3 | **Phenylalanine ammonia-lyase class 3** | 102.3 | 0.440 | 11.0 | 18.0 | 343.9 | 69.3 | 0.04 |
| 61 | A4GG90 | ATPB | **ATP synthase subunit beta, chloroplastic** | 9.2 | 0.440 | 11.0 | 8.0 | 142.0 | 15.7 | 0.05 |
| 62 | P26690 | 6DCS | **NAD(P)H-dependent 6'-deoxychalcone synthase** | 35.7 | 0.445 | 10.0 | 7.0 | 63.2 | 95.6 | 0.05 |
| 63 | P35334 | PGIP1 | **Polygalacturonase inhibitor 1** | 74.5 | 0.454 | 6.0 | 3.0 | 34.9 | 20.8 | 0.04 |
| 64 | P05037 | ATPB | **ATP synthase subunit beta, chloroplastic** | 15.2 | 0.454 | 6.0 | 9.0 | 115.5 | 14.4 | 0.02 |
| 65 | I1L3T1 | 708D1 | **UDP-glycosyltransferase 708D1** | 22.5 | 0.454 | 4.0 | 10.0 | 115.7 | 6.8 | 0.03 |
| 66 | Q9XHM5 | G2OX2 | **Gibberellin 2-beta-dioxygenase 2** | 43.7 | 0.458 | 6.0 | 4.0 | 158.2 | 18.5 | 0.05 |
| 67 | O82515 | MTDH | **Probable mannitol dehydrogenase** | 102.9 | 0.458 | 10.0 | 4.0 | 102.6 | 77.3 | 0.03 |
| 68 | O04369 | RAC1 | **Rac-like GTP-binding protein RAC1** | 22.0 | 0.477 | 3.0 | 5.0 | 281.1 | 17.8 | 0.05 |
| 69 | Q01390 | SUS | **Sucrose synthase** | 57.4 | 0.477 | 18.0 | 15.0 | 116.2 | 245.1 | 0.01 |
| 70 | Q42797 | TCMO | **Trans-cinnamate 4-monooxygenase** | 44.2 | 0.482 | 11.0 | 8.0 | 102.5 | 69.8 | 0.02 |
| 71 | Q9TKI7 | ATPB | **ATP synthase subunit beta, chloroplastic** | 23.4 | 0.482 | 6.0 | 6.0 | 147.3 | 105.6 | 0.03 |
| 72 | P50246 | SAHH | **Adenosylhomocysteinase** | 53.1 | 0.492 | 9.0 | 12.0 | 207.5 | 85.1 | 0.01 |
| 73 | P10816 | LGB3 | **Leghemoglobin 3** | 14.9 | 0.497 | 2.0 | 2.0 | 15.3 | 54.6 | 0.03 |
| **Unchanged proteins** | | | | | | | | | | |
| 74 | P04991 | RBL | **Ribulose bisphosphate carboxylase large chain** | 138.5 | 0.502 | 6.0 | 6.0 | 429.1 | 795.5 | 0.10 |
| 75 | P49093 | ASNS2 | **Asparagine synthetase [glutamine-hydrolyzing] 2** | 165.7 | 0.512 | 15.0 | 15.0 | 65.7 | 545.2 | 0.30 |
| 76 | Q40357 | NO10 | **Early nodulin-10** | 43.6 | 0.517 | 3.0 | 4.0 | 10.0 | 105.0 | 0.20 |
| 77 | P49092 | ASNS1 | **Asparagine synthetase [glutamine-hydrolyzing] 1** | 11.8 | 0.522 | 16.0 | 15.0 | 61.5 | 393.0 | 0.50 |
| 78 | F5B8W1 | CONB3 | **Conglutin beta 3** | 95.6 | 0.522 | 7.0 | 12.0 | 282.6 | 429.3 | 0.50 |
| 79 | Q9BBU1 | RBL | **Ribulose bisphosphate carboxylase large chain** | 36.6 | 0.527 | 8.0 | 2.0 | 168.9 | 123.6 | 0.30 |
| 80 | A4GG84 | RR3 | **30S ribosomal protein S3, chloroplastic** | 116.1 | 0.533 | 5.0 | 11.0 | 144.8 | 153.0 | 0.10 |
| 81 | Q5QGZ8 | NRL4A | **Bifunctional nitrilase/nitrile hydratase NIT4A** | 52.8 | 0.543 | 5.0 | 4.0 | 10.0 | 249.8 | 0.40 |
| 82 | P31926 | SUS | **Sucrose synthase** | 34.6 | 0.543 | 21.0 | 17.0 | 731.2 | 762.6 | 0.10 |
| 83 | P29530 | OLEO1 | **P24 oleosin isoform A** | 44.6 | 0.543 | 6.0 | 4.0 | 222.0 | 410.4 | 0.10 |
| 84 | P09886 | HS21C | **Small heat shock protein, chloroplastic** | 202.5 | 0.543 | 6.0 | 2.0 | 317.5 | 467.0 | 0.20 |
| 85 | P43237 | ALL11 | **Allergen Ara h 1, clone P17** | 23.0 | 0.549 | 19.0 | 23.0 | 234.4 | 558.8 | 0.40 |
| 86 | Q42808 | TBP | **TATA-box-binding protein** | 109.7 | 0.549 | 9.0 | 4.0 | 198.0 | 197.1 | 0.10 |
| 87 | Q04672 | SBP | **Sucrose-binding protein** | 98.7 | 0.549 | 17.0 | 16.0 | 225.2 | 322.6 | 0.50 |
| 88 | O63094 | RBL | **Ribulose bisphosphate carboxylase large chain** | 36.6 | 0.549 | 9.0 | 7.0 | 308.1 | 248.7 | 0.50 |
| 89 | P02854 | VCLB | **Provicilin (Fragment)** | 63.3 | 0.549 | 6.0 | 6.0 | 165.1 | 81.6 | 0.20 |
| 90 | Q9BBP6 | NU5C | **NAD(P)H-quinone oxidoreductase subunit 5, chloroplastic** | 102.0 | 0.554 | 10.0 | 6.0 | 309.4 | 374.9 | 0.40 |
| 91 | A4GGB9 | CYF | **Cytochrome f** | 93.9 | 0.554 | 6.0 | 7.0 | 200.0 | 156.5 | 0.20 |
| 92 | Q41121 | NO30 | **Nodulin-30** | 217.3 | 0.554 | 8.0 | 4.0 | 445.7 | 432.3 | 0.40 |
| 93 | Q5W915 | USP | **UDP-sugar pyrophospharylase** | 63.1 | 0.554 | 12.0 | 18.0 | 784.9 | 412.2 | 0.10 |
| 94 | A0A109QYD3 | RMS3 | **Strigolactone esterase RMS3** | 110.8 | 0.554 | 7.0 | 4.0 | 72.8 | 139.9 | 0.40 |
| 95 | P18823 | ACCD | **Acetyl-coenzyme A carboxylase carboxyl transferase subunit beta, chloroplastic** | 55.3 | 0.566 | 11.0 | 11.0 | 407.6 | 485.6 | 0.20 |
| 96 | P48490 | PP1 | **Serine/threonine-protein phosphatase PP1** | 19.2 | 0.571 | 5.0 | 6.0 | 117.6 | 130.3 | 0.20 |
| 97 | O48901 | DPOD1 | **DNA polymerase delta catalytic subunit** | 112.0 | 0.571 | 31.0 | 34.0 | 118.8 | 347.0 | 0.30 |
| 98 | P83594 | IFXA | **Factor Xa inhibitor BuXI** | 169.6 | 0.583 | 3.0 | 3.0 | 321.5 | 248.0 | 0.10 |
| 99 | Q6WNR0 | C81E7 | **Isoflavone 2'-hydroxylase** | 69.1 | 0.589 | 15.0 | 12.0 | 183.7 | 303.1 | 0.40 |
| 100 | P27456 | GSHRP | **Glutathione reductase, chloroplastic/mitochondrial** | 43.4 | 0.589 | 13.0 | 7.0 | 308.4 | 233.9 | 0.10 |
| 101 | Q40987 | LECR | **Nodule lectin** | 291.4 | 0.589 | 2.0 | 2.0 | 15.7 | 118.3 | 0.50 |
| 102 | P11728 | NO75 | **Early nodulin-75 (Fragment)** | 1382.1 | 0.595 | 2.0 | 2.0 | 25.2 | 26.6 | 0.10 |
| 103 | Q5YJU6 | MATK | **Maturase K** | 23.6 | 0.595 | 7.0 | 7.0 | 70.9 | 258.3 | 0.40 |
| 104 | O49885 | RL13A | **60S ribosomal protein L13a** | 144.4 | 0.600 | 7.0 | 9.0 | 172.2 | 84.6 | 0.40 |
| 105 | P25011 | CCNB1 | **G2/mitotic-specific cyclin S13-6** | 116.6 | 0.600 | 16.0 | 7.0 | 607.7 | 362.7 | 0.40 |
| 106 | Q6E2Z6 | REHY | **1-Cys peroxiredoxin** | 51.0 | 0.607 | 5.0 | 4.0 | 447.0 | 396.3 | 0.10 |
| 107 | Q5H873 | HLTT | **13-hydroxylupanine O-tigloyltransferase** | 6.0 | 0.607 | 4.0 | 9.0 | 203.4 | 313.3 | 0.20 |
| 108 | A6BM07 | I7GT1 | **Isoflavone 7-O-glucosyltransferase 1** | 157.4 | 0.607 | 9.0 | 8.0 | 66.2 | 204.0 | 0.50 |
| 109 | P37228 | MDHG | **Malate dehydrogenase, glyoxysomal** | 163.0 | 0.613 | 5.0 | 8.0 | 197.8 | 200.8 | 0.50 |
| 110 | Q9BBR8 | CYF | **Cytochrome f** | 108.4 | 0.613 | 8.0 | 5.0 | 273.4 | 279.7 | 0.50 |
| 111 | Q9AWB2 | SIR | **Sulfite reductase [ferredoxin], chloroplastic (Fragment)** | 66.2 | 0.613 | 15.0 | 12.0 | 153.7 | 168.8 | 0.20 |
| 112 | Q8MCJ4 | MATK | **Maturase K** | 30.8 | 0.619 | 10.0 | 13.0 | 388.3 | 662.3 | 0.20 |
| 113 | Q9BBU0 | ATPB | **ATP synthase subunit beta, chloroplastic** | 75.4 | 0.619 | 6.0 | 10.0 | 443.0 | 108.2 | 0.10 |
| 114 | Q43715 | TOC75 | **Protein TOC75, chloroplastic** | 60.7 | 0.625 | 19.0 | 26.0 | 155.4 | 98.1 | 0.40 |
| 115 | Q5YK47 | MATK | **Maturase K** | 34.5 | 0.631 | 12.0 | 11.0 | 330.0 | 109.1 | 0.50 |
| 116 | P14594 | LEGB | **Legumin B (Fragment)** | 49.4 | 0.631 | 9.0 | 10.0 | 300.6 | 182.5 | 0.30 |
| 117 | O81980 | CFI1 | **Chalcone-flavonone isomerase 1 (Fragment)** | 7.1 | 0.631 | 9.0 | 6.0 | 369.1 | 235.0 | 0.10 |
| 118 | P25698 | EF1A | **Elongation factor 1-alpha** | 95.4 | 0.631 | 13.0 | 8.0 | 103.5 | 214.0 | 0.40 |
| 119 | Q94IR2 | CCD1 | **Carotenoid 9,10(9',10')-cleavage dioxygenase 1** | 174.9 | 0.631 | 13.0 | 14.0 | 254.4 | 255.7 | 0.40 |
| 120 | P49347 | CONB | **Concanavalin B** | 58.2 | 0.638 | 9.0 | 5.0 | 150.1 | 234.1 | 0.20 |
| 121 | P93471 | COP1 | **E3 ubiquitin-protein ligase COP1** | 51.0 | 0.638 | 16.0 | 16.0 | 122.0 | 177.8 | 0.40 |
| 122 | Q96423 | TCMO | **Trans-cinnamate 4-monooxygenase** | 89.8 | 0.644 | 15.0 | 16.0 | 406.6 | 277.5 | 0.30 |
| 123 | Q9FZL4 | MGDG | **Probable monogalactosyldiacylglycerol synthase, chloroplastic** | 99.8 | 0.657 | 9.0 | 15.0 | 545.5 | 486.0 | 0.30 |
| 124 | P51139 | MSK3 | **Glycogen synthase kinase-3 homolog MsK-3** | 186.1 | 0.664 | 12.0 | 7.0 | 228.2 | 175.7 | 0.50 |
| 125 | Q6J540 | C79D4 | **Isoleucine N-monooxygenase 2** | 66.2 | 0.664 | 15.0 | 7.0 | 230.6 | 283.4 | 0.30 |
| 126 | Q07185 | AOX1 | **Ubiquinol oxidase 1, mitochondrial** | 63.4 | 0.670 | 11.0 | 9.0 | 259.9 | 230.7 | 0.40 |
| 127 | Q41266 | AOX2 | **Ubiquinol oxidase 2, mitochondrial** | 48.0 | 0.670 | 6.0 | 9.0 | 166.0 | 215.1 | 0.10 |
| 128 | O65015 | AMYB | **Beta-amylase** | 122.3 | 0.670 | 9.0 | 6.0 | 214.7 | 169.7 | 0.50 |
| 129 | F5B8W5 | CONB7 | **Conglutin beta 7** | 65.7 | 0.670 | 16.0 | 12.0 | 794.0 | 952.1 | 0.10 |
| 130 | Q41008 | ACCA | **Acetyl-coenzyme A carboxylase carboxyl transferase subunit alpha, chloroplastic** | 47.4 | 0.670 | 17.0 | 24.0 | 725.5 | 992.0 | 0.40 |
| 131 | P58822 | PGIP2 | **Polygalacturonase inhibitor 2** | 74.5 | 0.677 | 7.0 | 7.0 | 682.1 | 810.7 | 0.10 |
| 132 | Q40316 | VESTR | **Vestitone reductase** | 110.0 | 0.677 | 9.0 | 6.0 | 259.8 | 451.4 | 0.50 |
| 133 | P58823 | PGIP3 | **Polygalacturonase inhibitor 3** | 26.7 | 0.684 | 3.0 | 7.0 | 160.1 | 265.5 | 0.20 |
| 134 | Q42919 | G6PD | **Glucose-6-phosphate 1-dehydrogenase, cytoplasmic isoform** | 40.4 | 0.684 | 13.0 | 9.0 | 197.2 | 208.4 | 0.50 |
| 135 | P32646 | COX2 | **Cytochrome c oxidase subunit 2, mitochondrial (Fragment)** | 89.6 | 0.684 | 10.0 | 11.0 | 335.3 | 358.3 | 0.20 |
| 136 | Q1W376 | PMM | **Phosphomannomutase** | 41.3 | 0.684 | 4.0 | 5.0 | 406.8 | 300.5 | 0.10 |
| 137 | P05311 | PSAB | **Photosystem I P700 chlorophyll a apoprotein A2** | 17.3 | 0.684 | 4.0 | 5.0 | 130.0 | 102.9 | 0.10 |
| 138 | P27066 | RBL | **Ribulose bisphosphate carboxylase large chain** | 36.6 | 0.684 | 4.0 | 6.0 | 72.1 | 225.3 | 0.40 |
| 139 | P02873 | LEA1 | **Alpha-amylase inhibitor 1** | 18.7 | 0.684 | 10.0 | 4.0 | 77.3 | 65.7 | 0.40 |
| 140 | Q2TE74 | PCS2 | **Glutathione gamma-glutamylcysteinyltransferase 2** | 35.4 | 0.691 | 7.0 | 6.0 | 101.9 | 13.1 | 0.50 |
| 141 | P12628 | MAOX | **NADP-dependent malic enzyme** | 25.9 | 0.691 | 10.0 | 13.0 | 945.7 | 349.0 | 0.50 |
| 142 | Q43876 | SPSA | **Probable sucrose-phosphate synthase** | 73.5 | 0.691 | 18.0 | 29.0 | 325.2 | 212.4 | 0.20 |
| 143 | P92397 | RBL | **Ribulose bisphosphate carboxylase large chain (Fragment)** | 115.1 | 0.691 | 5.0 | 5.0 | 221.1 | 110.5 | 0.40 |
| 144 | Q8MCR7 | MATK | **Maturase K** | 26.3 | 0.698 | 15.0 | 8.0 | 477.4 | 531.0 | 0.30 |
| 145 | O04235 | SSRP1 | **FACT complex subunit SSRP1** | 87.1 | 0.698 | 11.0 | 10.0 | 809.9 | 788.8 | 0.30 |
| 146 | P93324 | CHOMT | **Isoliquiritigenin 2'-O-methyltransferase** | 34.1 | 0.705 | 11.0 | 8.0 | 508.5 | 472.5 | 0.10 |
| 147 | Q43092 | SSG1 | **Granule-bound starch synthase 1, chloroplastic/amyloplastic** | 148.5 | 0.705 | 11.0 | 12.0 | 276.0 | 612.0 | 0.30 |
| 148 | D2XNR0 | FLOT3 | **Flotillin-like protein 3** | 48.1 | 0.705 | 10.0 | 15.0 | 991.8 | 819.3 | 0.40 |
| 149 | P19251 | ASNS1 | **Asparagine synthetase, nodule [glutamine-hydrolyzing]** | 113.4 | 0.719 | 13.0 | 19.0 | 107.8 | 152.8 | 0.20 |
| 150 | P69589 | RBL | **Ribulose bisphosphate carboxylase large chain (Fragment)** | 115.1 | 0.719 | 4.0 | 17.0 | 133.0 | 169.3 | 0.50 |
| 151 | Q42798 | C93A1 | **3,9-dihydroxypterocarpan 6A-monooxygenase** | 110.9 | 0.719 | 15.0 | 16.0 | 539.1 | 623.6 | 0.10 |
| 152 | O04862 | FOLM | **Folate synthesis bifunctional protein, mitochondrial** | 109.1 | 0.719 | 9.0 | 14.0 | 503.1 | 721.5 | 0.20 |
| 153 | Q6RET7 | CCAMK | **Calcium and calcium/calmodulin-dependent serine/ threonine-protein kinase DMI-3** | 55.4 | 0.726 | 15.0 | 13.0 | 459.3 | 861.9 | 0.10 |
| 154 | Q2TSC7 | PCS1 | **Glutathione gamma-glutamylcysteinyltransferase 1** | 6.2 | 0.726 | 3.0 | 6.0 | 65.2 | 99.0 | 0.40 |
| 155 | P13915 | CVCA | **Convicilin** | 57.2 | 0.733 | 15.0 | 14.0 | 678.4 | 510.8 | 0.10 |
| 156 | Q8MCA5 | RR3 | **30S ribosomal protein S3, chloroplastic** | 101.2 | 0.733 | 10.0 | 11.0 | 249.1 | 227.3 | 0.50 |
| 157 | Q9BBN7 | RR15 | **30S ribosomal protein S15, chloroplastic** | 67.5 | 0.733 | 3.0 | 8.0 | 205.6 | 54.5 | 0.50 |
| 158 | Q0GXS4 | NFP | **Serine/threonine receptor-like kinase NFP** | 123.6 | 0.733 | 11.0 | 10.0 | 291.4 | 246.3 | 0.50 |
| 159 | P06669 | CYF | **Cytochrome f** | 291.8 | 0.733 | 11.0 | 7.0 | 101.2 | 127.1 | 0.20 |
| 160 | P49161 | CYF | **Cytochrome f** | 24.0 | 0.741 | 5.0 | 3.0 | 267.0 | 331.1 | 0.40 |
| 161 | B5LMP9 | PSBB | **Photosystem II CP47 reaction center protein** | 58.7 | 0.741 | 6.0 | 12.0 | 260.7 | 164.6 | 0.30 |
| 162 | Q9TKP6 | MATK | **Maturase K** | 50.1 | 0.741 | 11.0 | 15.0 | 115.8 | 529.8 | 0.30 |
| 163 | O24301 | SUS2 | **Sucrose synthase 2** | 93.3 | 0.748 | 25.0 | 16.0 | 150.2 | 134.7 | 0.50 |
| 164 | Q2PMP7 | RR3 | **30S ribosomal protein S3, chloroplastic** | 139.1 | 0.748 | 8.0 | 10.0 | 427.2 | 563.0 | 0.50 |
| 165 | P69572 | RBL | **Ribulose bisphosphate carboxylase large chain (Fragment)** | 115.1 | 0.748 | 3.0 | 7.0 | 255.5 | 245.0 | 0.50 |
| 166 | Q5YK03 | MATK | **Maturase K** | 23.8 | 0.748 | 12.0 | 6.0 | 355.4 | 340.0 | 0.10 |
| 167 | P46277 | CCNB1 | **G2/mitotic-specific cyclin-1** | 18.2 | 0.756 | 10.0 | 8.0 | 366.1 | 91.2 | 0.30 |
| 168 | P28553 | CRTI | **Phytoene dehydrogenase, chloroplastic/chromoplastic** | 65.3 | 0.756 | 10.0 | 5.0 | 275.0 | 108.1 | 0.20 |
| 169 | O24509 | INVA | **Acid beta-fructofuranosidase** | 46.4 | 0.756 | 7.0 | 15.0 | 428.0 | 269.7 | 0.40 |
| 170 | P69591 | RBL | **Ribulose bisphosphate carboxylase large chain (Fragment)** | 115.1 | 0.756 | 6.0 | 6.0 | 221.1 | 221.2 | 0.30 |
| 171 | P13708 | SUS | **Sucrose synthase** | 50.7 | 0.756 | 21.0 | 17.0 | 518.8 | 289.0 | 0.30 |
| 172 | Q42777 | MCCA | **Methylcrotonoyl-CoA carboxylase subunit alpha, mitochondrial** | 59.4 | 0.756 | 17.0 | 15.0 | 25.0 | 451.2 | 0.50 |
| 173 | O81973 | C93A3 | **Cytochrome P450 93A3 (secondary metabolism)** | 38.7 | 0.756 | 7.0 | 5.0 | 206.6 | 192.3 | 0.50 |
| 174 | B3EWQ9 | LECA2 | **Lectin alpha chain** | 231.2 | 0.763 | 5.0 | 7.0 | 164.8 | 58.6 | 0.30 |
| 175 | Q8MCM4 | MATK | **Maturase K** | 68.6 | 0.763 | 13.0 | 7.0 | 99.3 | 71.7 | 0.20 |
| 176 | Q93XE6 | CFI1A | **Chalcone-flavonone isomerase 1A** | 112.3 | 0.763 | 7.0 | 4.0 | 313.7 | 123.8 | 0.50 |
| 177 | P10933 | FENR | **Ferredoxin--NADP reductase; leaf isozyme; chloroplastic** | 79.6 | 0.763 | 6.0 | 6.0 | 80.5 | 341.5 | 0.40 |
| 178 | P30124 | HEM2 | **Delta-aminolevulinic acid dehydratase, chloroplastic (Fragment)** | 31.2 | 0.763 | 5.0 | 10.0 | 64.0 | 130.0 | 0.50 |
| 179 | P34811 | EFGC1 | **Elongation factor G-1, chloroplastic** | 80.2 | 0.763 | 17.0 | 16.0 | 108.1 | 193.5 | 0.30 |
| 180 | P69584 | RBL | **Ribulose bisphosphate carboxylase large chain (Fragment)** | 115.1 | 0.763 | 5.0 | 5.0 | 314.5 | 351.6 | 0.30 |
| 181 | Q42807 | STAD | **Stearoyl-[acyl-carrier-protein] 9-desaturase, chloroplastic** | 297.6 | 0.763 | 7.0 | 10.0 | 226.4 | 234.0 | 0.30 |
| 182 | P12859 | G3PB | **Glyceraldehyde-3-phosphate dehydrogenase B, chloroplastic** | 72.8 | 0.771 | 16.0 | 13.0 | 404.9 | 324.0 | 0.50 |
| 183 | P41346 | FENR | **Ferredoxin-NADP reductase, chloroplastic** | 143.6 | 0.771 | 9.0 | 10.0 | 76.3 | 155.6 | 0.30 |
| 184 | A4GGD1 | PSBB | **Photosystem II CP47 reaction center protein** | 34.5 | 0.771 | 7.0 | 6.0 | 407.5 | 184.1 | 0.20 |
| 185 | Q5YK50 | MATK | **Maturase K** | 44.5 | 0.771 | 11.0 | 7.0 | 124.0 | 175.6 | 0.10 |
| 186 | Q4VYC8 | NSP1 | **Nodulation-signaling pathway 1 protein** | 103.2 | 0.771 | 7.0 | 15.0 | 93.5 | 161.1 | 0.10 |
| 187 | P04347 | GLYG5 | **Glycinin** | 53.0 | 0.779 | 7.0 | 11.0 | 82.2 | 148.9 | 0.40 |
| 188 | P29001 | INVA | **Acid beta-fructofuranosidase** | 62.9 | 0.779 | 19.0 | 17.0 | 455.5 | 671.5 | 0.30 |
| 189 | P45458 | MASY | **Malate synthase, glyoxysomal (Fragment)** | 42.1 | 0.779 | 15.0 | 15.0 | 122.0 | 168.3 | 0.10 |
| 190 | Q9BBS1 | ACCD | **Acetyl-coenzyme A carboxylase carboxyl transferase subunit beta, chloroplastic** | 83.6 | 0.779 | 12.0 | 5.0 | 175.2 | 124.3 | 0.50 |
| 191 | Q43067 | TCMO | **Trans-cinnamate 4-monooxygenase** | 46.5 | 0.779 | 16.0 | 11.0 | 77.6 | 518.1 | 0.10 |
| 192 | I1K0K6 | EFGC2 | **Elongation factor G-2, chloroplastic** | 75.5 | 0.779 | 13.0 | 21.0 | 171.6 | 49.0 | 0.30 |
| 193 | F5B8W3 | CONB5 | **Conglutin beta 5** | 67.6 | 0.779 | 21.0 | 26.0 | 46.4 | 154.7 | 0.50 |
| 194 | Q5YK01 | MATK | **Maturase K** | 4.9 | 0.779 | 8.0 | 16.0 | 80.4 | 232.9 | 0.40 |
| 195 | A3RF67 | BAGBG | **Isoflavonoid 7-O-beta-apiosyl-glucoside beta-glycosidase** | 77.5 | 0.779 | 18.0 | 8.0 | 138.1 | 54.5 | 0.50 |
| 196 | Q2PMU3 | PSAB | **Photosystem I P700 chlorophyll a apoprotein A1** | 79.6 | 0.787 | 7.0 | 7.0 | 235.6 | 238.0 | 0.30 |
| 197 | P93998 | RBL | **Ribulose bisphosphate carboxylase large chain (Fragment)** | 38.7 | 0.787 | 4.0 | 11.0 | 22.7 | 212.3 | 0.20 |
| 198 | P24099 | GLNA1 | **Glutamine synthetase cytosolic isozyme 1** | 20.9 | 0.787 | 3.0 | 6.0 | 123.9 | 107.1 | 0.20 |
| 199 | P53536 | PHSL | **Alpha-1,4 glucan phosphorylase L isozyme,  chloroplastic/amyloplastic** | 120.6 | 0.787 | 24.0 | 32.0 | 267.4 | 323.1 | 0.10 |
| 200 | A4GG97 | PSAA | **Photosystem I P700 chlorophyll a apoprotein A1** | 79.6 | 0.787 | 5.0 | 3.0 | 95.8 | 168.1 | 0.50 |
| 201 | Q8MCR8 | MATK | **Maturase K** | 73.0 | 0.795 | 12.0 | 15.0 | 121.6 | 115.9 | 0.30 |
| 202 | P46280 | EFTU2 | **Elongation factor Tu, chloroplastic** | 28.2 | 0.795 | 9.0 | 12.0 | 82.2 | 140.5 | 0.30 |
| 203 | Q01861 | PAL1 | **Phenylalanine ammonia-lyase 1** | 87.9 | 0.795 | 16.0 | 21.0 | 25.7 | 72.1 | 0.30 |
| 204 | P05310 | PSAA | **Photosystem I P700 chlorophyll a apoprotein A1** | 95.2 | 0.795 | 7.0 | 10.0 | 241.7 | 112.2 | 0.10 |
| 205 | P08281 | GLNA2 | **Glutamine synthetase leaf isozyme, chloroplastic** | 14.8 | 0.795 | 9.0 | 9.0 | 19.6 | 154.1 | 0.20 |
| 206 | P69579 | RBL | **Ribulose bisphosphate carboxylase large chain (Fragment)** | 115.1 | 0.795 | 7.0 | 7.0 | 133.0 | 74.8 | 0.30 |
| 207 | Q40983 | SPP | **Stromal processing peptidase, chloroplastic** | 63.4 | 0.795 | 28.0 | 11.0 | 216.4 | 77.1 | 0.30 |
| 208 | Q9MUK5 | TOC64 | **Translocon at the outer membrane of chloroplasts 64** | 121.3 | 0.795 | 8.0 | 11.0 | 123.0 | 123.3 | 0.20 |
| 209 | O04279 | GPA2 | **Guanine nucleotide-binding protein alpha-2 subunit** | 43.2 | 0.795 | 12.0 | 11.0 | 68.9 | 146.6 | 0.10 |
| 210 | O65194 | RBS | **Ribulose bisphosphate carboxylase small chain, chloroplastic** | 23.1 | 0.803 | 1.0 | 5.0 | 6.8 | 72.2 | 0.50 |
| 211 | P13918 | VCLC | **Vicilin** | 94.2 | 0.803 | 16.0 | 11.0 | 21.7 | 10.0 | 0.40 |
| 212 | Q9SM60 | PGMC | **Phosphoglucomutase, cytoplasmic** | 131.3 | 0.803 | 8.0 | 13.0 | 41.5 | 105.1 | 0.50 |
| 213 | Q9BBP8 | RR3 | **30S ribosomal protein S3, chloroplastic** | 78.8 | 0.803 | 7.0 | 11.0 | 78.5 | 77.6 | 0.10 |
| 214 | Q43056 | CFI | **Chalcone-flavonone isomerase** | 68.2 | 0.803 | 6.0 | 4.0 | 108.8 | 75.6 | 0.50 |
| 215 | O49817 | LEA2 | **Late embryogenesis abundant protein 2** | 74.4 | 0.803 | 7.0 | 9.0 | 34.4 | 32.0 | 0.40 |
| 216 | P46258 | ACT3 | **Actin-3** | 92.2 | 0.811 | 10.0 | 12.0 | 169.3 | 99.5 | 0.50 |
| 217 | P27480 | LOXA | **Linoleate 9S-lipoxygenase 1** | 31.9 | 0.811 | 17.0 | 21.0 | 56.7 | 30.2 | 0.50 |
| 218 | Q5YJX4 | MATK | **Maturase K** | 12.8 | 0.811 | 8.0 | 9.0 | 40.5 | 35.5 | 0.20 |
| 219 | P69580 | RBL | **Ribulose bisphosphate carboxylase large chain (Fragment** | 115.1 | 0.811 | 6.0 | 6.0 | 86.2 | 96.2 | 0.10 |
| 220 | P49364 | GCST | **Aminomethyltransferase, mitochondrial** | 76.5 | 0.811 | 10.0 | 9.0 | 189.3 | 44.8 | 0.50 |
| 221 | P86349 | EFGC | **Elongation factor G, chloroplastic (Fragments)** | 56.0 | 0.811 | 5.0 | 3.0 | 57.6 | 156.6 | 0.50 |
| 222 | O48921 | C97B2 | **Cytochrome P450 97B2, chloroplastic** | 237.0 | 0.811 | 9.0 | 6.0 | 164.7 | 26.0 | 0.50 |
| 223 | Q9SWB4 | PARP3 | **Poly [ADP-ribose] polymerase 3** | 42.7 | 0.819 | 25.0 | 25.0 | 213.6 | 432.0 | 0.10 |
| 224 | Q8MCM9 | MATK | **Maturase K** | 24.9 | 0.819 | 7.0 | 15.0 | 61.0 | 79.5 | 0.30 |
| 225 | P19252 | ASNS2 | **Asparagine synthetase, root [glutamine-hydrolyzing]** | 137.0 | 0.819 | 16.0 | 16.0 | 48.0 | 59.7 | 0.20 |
| 226 | Q6TND0 | MATK | **Maturase K** | 110.6 | 0.819 | 16.0 | 8.0 | 64.0 | 30.3 | 0.10 |
| 227 | Q9BBS3 | ATPA | **ATP synthase subunit alpha, chloroplastic** | 38.3 | 0.819 | 6.0 | 6.0 | 81.5 | 66.8 | 0.40 |
| 228 | Q9M6E8 | NCED1 | **9-cis-epoxycarotenoid dioxygenase NCED1, chloroplastic** | 50.8 | 0.819 | 14.0 | 14.0 | 98.4 | 14.7 | 0.40 |
| 229 | P26205 | BGLT | **Cyanogenic beta-glucosidase (Fragment)** | 38.9 | 0.819 | 11.0 | 6.0 | 99.7 | 68.4 | 0.50 |
| 230 | P31163 | RK2 | **50S ribosomal protein L2, chloroplastic** | 82.3 | 0.819 | 13.0 | 13.0 | 118.3 | 57.3 | 0.20 |
| 231 | Q41062 | SECA | **Protein translocase subunit SecA, chloroplastic** | 78.4 | 0.819 | 29.0 | 22.0 | 159.3 | 72.6 | 0.20 |
| 232 | Q41060 | SBP65 | **Seed biotin-containing protein SBP65** | 96.9 | 0.819 | 20.0 | 18.0 | 96.3 | 34.2 | 0.10 |
| 233 | Q8MCJ5 | MATK | **Maturase K** | 39.7 | 0.827 | 10.0 | 9.0 | 64.5 | 68.6 | 0.30 |
| 234 | Q9SM59 | PGMP | **Phosphoglucomutase, chloroplastic** | 203.4 | 0.827 | 10.0 | 13.0 | 46.5 | 100.9 | 0.50 |
| 235 | A4GGA5 | RPOB | **DNA-directed RNA polymerase subunit beta** | 82.3 | 0.827 | 23.0 | 27.0 | 156.4 | 43.0 | 0.40 |
| 236 | Q9M6E9 | AGGL | **Agglutinin-1** | 171.2 | 0.827 | 13.0 | 11.0 | 106.5 | 169.5 | 0.30 |
| 237 | Q5YJX3 | MATK | **Maturase K** | 22.9 | 0.827 | 8.0 | 11.0 | 112.3 | 34.7 | 0.20 |
| 238 | P34922 | G3PC | **Glyceraldehyde-3-phosphate dehydrogenase, cytosolic** | 91.0 | 0.835 | 8.0 | 10.0 | 122.6 | 21.9 | 0.10 |
| 239 | O48560 | CATA3 | **Catalase-3** | 25.2 | 0.835 | 10.0 | 20.0 | 44.8 | 197.8 | 0.50 |
| 240 | Q9TK08 | MATK | **Maturase K** | 138.0 | 0.835 | 7.0 | 11.0 | 70.1 | 78.8 | 0.40 |
| 241 | C6L7U1 | LIN1 | **Putative E3 ubiquitin-protein ligase LIN-1** | 123.8 | 0.835 | 35.0 | 34.0 | 374.0 | 65.6 | 0.50 |
| 242 | P42348 | PI3K2 | **Phosphatidylinositol 3-kinase, nodule isoform** | 88.7 | 0.835 | 13.0 | 10.0 | 30.6 | 20.5 | 0.30 |
| 243 | Q04903 | FNTB | **Protein farnesyltransferase subunit beta** | 70.7 | 0.835 | 6.0 | 5.0 | 80.4 | 45.0 | 0.20 |
| 244 | O48665 | LGB5 | **Leghemoglobin Lb120-29** | 191.8 | 0.835 | 4.0 | 3.0 | 44.5 | 14.7 | 0.30 |
| 245 | P29756 | CATA1 | **Catalase-1/2** | 23.5 | 0.835 | 10.0 | 7.0 | 70.8 | 90.6 | 0.20 |
| 246 | Q8MCN3 | MATK | **Maturase K** | 53.7 | 0.844 | 11.0 | 8.0 | 37.2 | 43.7 | 0.40 |
| 247 | Q2PMT9 | PSBC | **Photosystem II CP43 reaction center protein** | 66.1 | 0.844 | 6.0 | 6.0 | 156.8 | 326.4 | 0.10 |
| 248 | P45457 | ACEA2 | **Isocitrate lyase 2 (Fragment)** | 70.5 | 0.844 | 15.0 | 15.0 | 126.8 | 84.8 | 0.50 |
| 249 | Q9TKP0 | MATK | **Maturase K** | 157.5 | 0.844 | 10.0 | 17.0 | 116.4 | 58.6 | 0.30 |
| 250 | Q75NZ0 | SIR | **Sulfite reductase [ferredoxin], chloroplastic** | 64.3 | 0.852 | 16.0 | 14.0 | 45.3 | 52.6 | 0.40 |
| 251 | P93673 | PHYA | **Phytochrome type A** | 31.2 | 0.852 | 7.0 | 10.0 | 20.2 | 100.0 | 0.10 |
| 252 | Q5YJU0 | MATK | **Maturase K** | 43.0 | 0.852 | 13.0 | 7.0 | 61.0 | 78.8 | 0.40 |
| 253 | P04776 | GLYG1 | **Glycinin G1** | 72.1 | 0.852 | 7.0 | 9.0 | 55.6 | 48.5 | 0.20 |
| 254 | P13088 | AUX22 | **Auxin-induced protein AUX22** | 87.8 | 0.852 | 4.0 | 5.0 | 6.0 | 102.0 | 0.10 |
| 255 | Q8MCR6 | MATK | **Maturase K** | 28.5 | 0.861 | 5.0 | 5.0 | 67.9 | 77.8 | 0.40 |
| 256 | Q2PMQ9 | PSBB | **Photosystem II CP47 reaction center protein** | 84.6 | 0.861 | 8.0 | 7.0 | 12.3 | 5.5 | 0.40 |
| 257 | Q02909 | CAPP1 | **Phosphoenolpyruvate carboxylase, housekeeping isozyme** | 41.2 | 0.861 | 26.0 | 18.0 | 44.6 | 55.6 | 0.10 |
| 258 | Q9AU12 | CAPP | **Phosphoenolpyruvate carboxylase** | 45.6 | 0.861 | 25.0 | 23.0 | 10.4 | 79.2 | 0.50 |
| 259 | P00155 | CYF | **Cytochrome f** | 48.4 | 0.861 | 8.0 | 11.0 | 28.1 | 98.0 | 0.30 |
| 260 | A0A0G4DBR5 | FG3H | **UDP-glycosyltransferase 79B30** | 12.2 | 0.869 | 5.0 | 5.0 | 314.8 | 51.2 | 0.50 |
| 261 | P24459 | ATPAM | **ATP synthase subunit alpha, mitochondrial** | 103.9 | 0.869 | 22.0 | 14.0 | 72.8 | 65.8 | 0.40 |
| 262 | Q672F7 | TPS2 | **Tricyclene synthase EBOS, chloroplastic** | 25.1 | 0.869 | 7.0 | 10.0 | 70.5 | 56.6 | 0.20 |
| 263 | D1FP53 | LIN | **Putative E3 ubiquitin-protein ligase LIN** | 118.8 | 0.869 | 19.0 | 42.0 | 70.4 | 90.5 | 0.20 |
| 264 | P37116 | NCPR | **NADPH-cytochrome P450 reductase** | 80.5 | 0.869 | 8.0 | 17.0 | 21.1 | 113.2 | 0.40 |
| 265 | P15231 | PHAM | **Leucoagglutinating phytohemagglutinin** | 90.0 | 0.869 | 3.0 | 5.0 | 61.3 | 6.1 | 0.20 |
| 266 | Q9TKI5 | MATK | **Maturase K** | 23.5 | 0.869 | 7.0 | 6.0 | 18.7 | 67.2 | 0.40 |
| 267 | Q8MCR5 | MATK | **Maturase K** | 28.5 | 0.878 | 13.0 | 10.0 | 78.8 | 87.1 | 0.10 |
| 268 | O24648 | G3OX | **Gibberellin 3-beta-dioxygenase 1** | 128.9 | 0.878 | 3.0 | 4.0 | 29.4 | 104.6 | 0.50 |
| 269 | P43238 | ALL12 | **Allergen Ara h 1, clone P41B** | 9.7 | 0.878 | 15.0 | 22.0 | 157.9 | 91.9 | 0.40 |
| 270 | Q9SPB1 | LEGRE | **Leghemoglobin reductase** | 41.0 | 0.878 | 15.0 | 7.0 | 70.8 | 43.2 | 0.10 |
| 271 | K7LC65 | DAT1C | **Diacylglycerol O-acyltransferase 1C** | 30.9 | 0.878 | 9.0 | 9.0 | 83.2 | 51.1 | 0.10 |
| 272 | Q9BBT1 | PSBC | **Photosystem II CP43 reaction center protein** | 66.1 | 0.878 | 6.0 | 6.0 | 34.8 | 124.0 | 0.20 |
| 273 | P55195 | PUR6 | **Phosphoribosylaminoimidazole carboxylase,  chloroplastic (Fragment)** | 53.3 | 0.878 | 12.0 | 11.0 | 33.6 | 111.8 | 0.40 |
| 274 | P13548 | VATA | **V-type proton ATPase catalytic subunit A** | 135.0 | 0.878 | 15.0 | 16.0 | 168.6 | 37.7 | 0.20 |
| 275 | P37114 | TCMO | **Trans-cinnamate 4-monooxygenase** | 67.4 | 0.878 | 8.0 | 13.0 | 55.8 | 53.0 | 0.10 |
| 276 | P51061 | CAPP2 | **Phosphoenolpyruvate carboxylase** | 38.4 | 0.878 | 28.0 | 17.0 | 88.0 | 93.3 | 0.40 |
| 277 | Q5YJU7 | MATK | **Maturase K** | 47.1 | 0.878 | 8.0 | 12.0 | 89.8 | 94.2 | 0.30 |
| 278 | Q5YK53 | MATK | **Maturase K** | 22.9 | 0.878 | 11.0 | 12.0 | 89.4 | 135.2 | 0.20 |
| 279 | P34799 | URIC2 | **Uricase-2 isozyme 2** | 15.5 | 0.878 | 6.0 | 6.0 | 20.2 | 115.1 | 0.50 |
| 280 | P31165 | RK15 | **50S ribosomal protein L15, chloroplastic (Fragment)** | 44.4 | 0.878 | 4.0 | 8.0 | 103.7 | 127.2 | 0.50 |
| 281 | P06006 | PSBD | **Photosystem II D2 protein** | 512.7 | 0.878 | 4.0 | 15.0 | 32.9 | 35.4 | 0.20 |
| 282 | Q6E4Q8 | MATK | **Maturase K** | 92.0 | 0.878 | 12.0 | 14.0 | 63.6 | 41.4 | 0.30 |
| 283 | Q9GI85 | MATK | **Maturase K** | 81.2 | 0.878 | 10.0 | 13.0 | 29.1 | 80.5 | 0.50 |
| 284 | A0A072VMJ3 | CN15C | **Protein CNGC15c** | 44.1 | 0.878 | 22.0 | 17.0 | 94.9 | 50.3 | 0.30 |
| 285 | Q02028 | HSP7S | **Stromal 70 kDa heat shock-related protein, chloroplastic** | 58.3 | 0.887 | 20.0 | 25.0 | 37.2 | 31.4 | 0.20 |
| 286 | Q9SMJ4 | LEG | **Legumin** | 88.8 | 0.887 | 6.0 | 9.0 | 2.6 | 40.7 | 0.10 |
| 287 | O24303 | TI110 | **Protein TIC110, chloroplastic** | 110.7 | 0.887 | 35.0 | 27.0 | 73.4 | 98.2 | 0.30 |
| 288 | Q8MCL7 | MATK | **Maturase K** | 102.9 | 0.887 | 4.0 | 4.0 | 33.5 | 38.9 | 0.50 |
| 289 | P54774 | CDC48 | **Cell division cycle protein 48 homolog** | 70.0 | 0.887 | 21.0 | 23.0 | 87.6 | 108.1 | 0.40 |
| 290 | Q6Q7X3 | MATK | **Maturase K** | 95.8 | 0.887 | 2.0 | 10.0 | 98.7 | 207.6 | 0.30 |
| 291 | D1FP57 | LIN2 | **Putative E3 ubiquitin-protein ligase LIN-2** | 74.5 | 0.887 | 25.0 | 24.0 | 91.4 | 383.2 | 0.40 |
| 292 | A4GGA1 | PSBC | **Photosystem II CP43 reaction center protein** | 66.1 | 0.887 | 6.0 | 11.0 | 69.5 | 48.0 | 0.40 |
| 293 | Q42899 | GLNA1 | **Glutamine synthetase cytosolic isozyme** | 1.2 | 0.887 | 4.0 | 3.0 | 73.1 | 106.5 | 0.10 |
| 294 | P23569 | CHSY | **Chalcone synthase** | 57.4 | 0.887 | 7.0 | 6.0 | 106.5 | 180.0 | 0.30 |
| 295 | I1N2Z5 | SLE1 | **Protein SLE1** | 81.1 | 0.887 | 6.0 | 7.0 | 59.9 | 68.7 | 0.10 |
| 296 | P13089 | AUX28 | **Auxin-induced protein AUX28** | 47.7 | 0.887 | 4.0 | 4.0 | 108.4 | 100.0 | 0.30 |
| 297 | Q8MCL8 | MATK | **Maturase K** | 78.8 | 0.896 | 10.0 | 17.0 | 48.6 | 38.9 | 0.40 |
| 298 | A0A172J2D0 | UGT2 | **UDP-glycosyltransferase 2** | 72.9 | 0.896 | 14.0 | 7.0 | 51.5 | 64.4 | 0.50 |
| 299 | O80405 | LGB3 | **Leghemoglobin Lb120-1** | 483.0 | 0.896 | 5.0 | 8.0 | 22.8 | 75.3 | 0.50 |
| 300 | Q6TND4 | MATK | **Maturase K** | 110.6 | 0.896 | 7.0 | 7.0 | 34.0 | 18.8 | 0.10 |
| 301 | Q8HVY5 | RPOB | **DNA-directed RNA polymerase subunit beta** | 85.0 | 0.896 | 22.0 | 25.0 | 39.6 | 54.8 | 0.30 |
| 302 | P42347 | PI3K1 | **Phosphatidylinositol 3-kinase, root isoform** | 110.0 | 0.896 | 19.0 | 13.0 | 25.8 | 26.6 | 0.10 |
| 303 | B5LMM0 | PSBC | **Photosystem II CP43 reaction center protein** | 66.1 | 0.896 | 16.0 | 8.0 | 29.2 | 45.6 | 0.10 |
| 304 | G7IBJ4 | CN15A | **Protein CNGC15a** | 29.3 | 0.896 | 24.0 | 22.0 | 56.4 | 90.1 | 0.10 |
| 305 | Q8MCM1 | MATK | **Maturase K** | 82.5 | 0.905 | 10.0 | 13.0 | 45.1 | 23.5 | 0.50 |
| 306 | Q948P5 | FRI4 | **Ferritin-4, chloroplastic** | 120.0 | 0.905 | 7.0 | 11.0 | 41.6 | 8.7 | 0.50 |
| 307 | Q9SXV6 | CAS1 | **Cycloartenol synthase** | 74.3 | 0.905 | 9.0 | 13.0 | 54.7 | 102.4 | 0.50 |
| 308 | Q5YJY5 | MATK | **Maturase K** | 62.1 | 0.905 | 13.0 | 14.0 | 23.8 | 42.6 | 0.10 |
| 309 | Q6EBC1 | CONB2 | **Conglutin beta 2** | 113.2 | 0.905 | 15.0 | 9.0 | 32.5 | 78.2 | 0.40 |
| 310 | P29828 | PDI | **Protein disulfide-isomerase** | 61.9 | 0.905 | 6.0 | 10.0 | 33.3 | 80.3 | 0.40 |
| 311 | Q9SAZ0 | LB120-34 | **Leghemoglobin Lb120-34** | 220.4 | 0.905 | 5.0 | 5.0 | 22.5 | 112.1 | 0.10 |
| 312 | P38414 | LOX1 | **Linoleate 9S-lipoxygenase** | 30.4 | 0.905 | 23.0 | 16.0 | 40.1 | 74.5 | 0.50 |
| 313 | P02858 | GLYG4 | **Glycinin G4** | 57.1 | 0.905 | 3.0 | 6.0 | 88.1 | 106.5 | 0.10 |
| 314 | Q9TKR9 | MATK | **Maturase K** | 37.1 | 0.905 | 10.0 | 14.0 | 59.6 | 106.6 | 0.10 |
| 315 | Q8MCS1 | MATK | **Maturase K** | 22.1 | 0.914 | 11.0 | 9.0 | 69.5 | 48.4 | 0.50 |
| 316 | O22437 | CHLD | **Magnesium-chelatase subunit ChlD, chloroplastic** | 22.2 | 0.914 | 21.0 | 17.0 | 81.4 | 102.5 | 0.30 |
| 317 | Q8LKZ1 | NORK | **Nodulation receptor kinase** | 31.1 | 0.914 | 18.0 | 17.0 | 17.5 | 81.7 | 0.40 |
| 318 | Q6RET6 | CCAMK | **Calcium and calcium/calmodulin-dependent serine/ threonine-protein kinase (Fragment)** | 123.2 | 0.914 | 16.0 | 19.0 | 47.4 | 63.6 | 0.50 |
| 319 | O65026 | SUS | **Sucrose synthase** | 37.9 | 0.914 | 19.0 | 19.0 | 6.7 | 55.7 | 0.40 |
| 320 | Q5YJV9 | MATK | **Maturase K** | 27.8 | 0.914 | 11.0 | 15.0 | 18.2 | 70.3 | 0.40 |
| 321 | B5LMM1 | PBSD | **Photosystem II D2 protein** | 512.7 | 0.914 | 6.0 | 6.0 | 18.6 | 52.8 | 0.10 |
| 322 | O20346 | RBL | **Ribulose bisphosphate carboxylase large chain (Fragment)** | 36.6 | 0.914 | 14.0 | 4.0 | 32.1 | 21.5 | 0.40 |
| 323 | Q8S3J3 | HIUH | **Hydroxyisourate hydrolase** | 225.7 | 0.914 | 8.0 | 11.0 | 30.9 | 108.2 | 0.30 |
| 324 | Q8MCP4 | MATK | **Maturase K** | 31.8 | 0.923 | 8.0 | 13.0 | 16.5 | 32.7 | 0.10 |
| 325 | Q8MCL9 | MATK | **Maturase K** | 115.0 | 0.923 | 11.0 | 16.0 | 95.0 | 67.2 | 0.10 |
| 326 | Q39846 | SBP65 | **Seed biotin-containing protein SBP65** | 147.9 | 0.923 | 27.0 | 29.0 | 74.1 | 15.5 | 0.50 |
| 327 | Q96450 | 1433A | **14-3-3-like protein A** | 67.6 | 0.923 | 6.0 | 6.0 | 29.7 | 12.8 | 0.50 |
| 328 | P32290 | CATA | **Catalase** | 32.6 | 0.923 | 11.0 | 8.0 | 46.6 | 145.4 | 0.40 |
| 329 | P16149 | RS16 | **40S ribosomal protein S16** | 334.8 | 0.923 | 5.0 | 4.0 | 46.5 | 56.3 | 0.10 |
| 330 | A9UL14 | RBR | **Retinoblastoma-related protein** | 79.1 | 0.923 | 18.0 | 23.0 | 101.1 | 80.0 | 0.50 |
| 331 | C0HK20 | LECC1 | **Mannose-specific lectin CML-2** | 27.1 | 0.923 | 5.0 | 4.0 | 22.8 | 26.5 | 0.30 |
| 332 | Q9AVK4 | SCR | **Protein SCARECROW** | 33.6 | 0.923 | 13.0 | 9.0 | 10.5 | 21.9 | 0.50 |
| 333 | P35055 | HEM6 | **Oxygen-dependent coproporphyrinogen-III oxidase,  chloroplastic** | 79.7 | 0.932 | 7.0 | 8.0 | 22.3 | 101.2 | 0.20 |
| 334 | P42499 | PHYB | **Phytochrome B** | 51.2 | 0.932 | 23.0 | 38.0 | 63.2 | 66.4 | 0.10 |
| 335 | Q02226 | COXT | **Cytochrome c oxidase subunit 2, mitochondrial (Fragment)** | 39.9 | 0.932 | 10.0 | 6.0 | 4.0 | 2.1 | 0.30 |
| 336 | I1N462 | SBT1 | **Subtilisin-like protease** | 272.8 | 0.932 | 14.0 | 19.0 | 23.6 | 46.4 | 0.40 |
| 337 | P48640 | GSHRP | **Glutathione reductase, chloroplastic** | 32.0 | 0.932 | 10.0 | 13.0 | 30.0 | 60.1 | 0.50 |
| 338 | P39870 | NIA2 | **Inducible nitrate reductase [NADH] 2** | 90.9 | 0.932 | 15.0 | 16.0 | 46.9 | 75.8 | 0.30 |
| 339 | Q9BBU2 | MATK | **Maturase K** | 87.4 | 0.942 | 9.0 | 12.0 | 31.6 | 59.1 | 0.50 |
| 340 | Q6PSE2 | MATK | **Maturase K** | 33.3 | 0.942 | 9.0 | 10.0 | 32.0 | 65.7 | 0.10 |
| 341 | Q8MCP5 | MATK | **Maturase K** | 31.8 | 0.951 | 6.0 | 8.0 | 30.0 | 107.1 | 0.20 |
| 342 | P48628 | FAD6C | **Omega-6 fatty acid desaturase, chloroplastic** | 16.5 | 0.951 | 5.0 | 8.0 | 12.1 | 46.7 | 0.20 |
| 343 | Q8HVY3 | RPOC2 | **DNA-directed RNA polymerase subunit beta** | 89.0 | 0.951 | 20.0 | 33.0 | 3.5 | 25.7 | 0.10 |
| 344 | Q41014 | FENR2 | **Ferredoxin-NADP reductase, root isozyme, chloroplastic** | 79.7 | 0.951 | 11.0 | 10.0 | 20.1 | 57.9 | 0.10 |
| 345 | P93163 | GPA2 | **Guanine nucleotide-binding protein alpha-2 subunit** | 112.4 | 0.951 | 13.0 | 13.0 | 115.1 | 84.2 | 0.30 |
| 346 | O64981 | RCA | **Ribulose bisphosphate carboxylase/oxygenase activase,  chloroplastic** | 141.6 | 0.961 | 10.0 | 11.0 | 9.1 | 81.3 | 0.20 |
| 347 | P09439 | LOX2 | **Seed linoleate 9S-lipoxygenase-2** | 62.8 | 0.961 | 23.0 | 25.0 | 56.1 | 99.2 | 0.10 |
| 348 | Q6PSC4 | MATK | **Maturase K** | 48.0 | 0.961 | 8.0 | 10.0 | 35.8 | 34.5 | 0.20 |
| 349 | Q6UDF0 | CSLA1 | **Mannan synthase 1** | 28.5 | 0.961 | 10.0 | 14.0 | 1.5 | 39.9 | 0.50 |
| 350 | Q9MB42 | BAMS | **Beta-amyrin synthase** | 19.8 | 0.961 | 21.0 | 8.0 | 42.0 | 95.6 | 0.10 |
| 351 | P25699 | FRI | **Ferritin, chloroplastic** | 151.2 | 0.961 | 7.0 | 6.0 | 3.8 | 16.9 | 0.30 |
| 352 | Q8MCP6 | MATK | **Maturase K** | 23.5 | 0.970 | 9.0 | 15.0 | 24.4 | 88.8 | 0.50 |
| 353 | Q8MCN9 | MATK | **Maturase K** | 102.9 | 0.970 | 12.0 | 9.0 | 17.5 | 102.8 | 0.50 |
| 354 | P12227 | RPOC2 | **DNA-directed RNA polymerase subunit beta (Fragment)** | 29.3 | 0.970 | 16.0 | 20.0 | 117.9 | 98.2 | 0.20 |
| 355 | Q9BBS9 | RPOB | **DNA-directed RNA polymerase subunit beta** | 87.2 | 0.970 | 19.0 | 18.0 | 18.9 | 44.7 | 0.50 |
| 356 | Q5H8A5 | POLLU | **Ion channel POLLUX** | 49.3 | 0.970 | 18.0 | 18.0 | 46.3 | 65.3 | 0.20 |
| 357 | Q39821 | SDLCA | **Dynamin-related protein 12A** | 138.7 | 0.970 | 18.0 | 14.0 | 25.0 | 7.2 | 0.50 |
| 358 | Q41114 | LEA2 | **Alpha-amylase inhibitor 2** | 75.9 | 0.970 | 3.0 | 5.0 | 12.7 | 15.0 | 0.30 |
| 359 | Q5YJV6 | MATK | **Maturase K** | 97.5 | 0.970 | 9.0 | 9.0 | 21.3 | 63.9 | 0.50 |
| 360 | P14298 | CFI | **Chalcone-flavonone isomerase** | 44.6 | 0.970 | 4.0 | 4.0 | 16.2 | 20.3 | 0.40 |
| 361 | P06004 | PBSC | **Photosystem II CP43 reaction center protein** | 66.1 | 0.970 | 15.0 | 7.0 | 71.9 | 19.0 | 0.20 |
| 362 | P04770 | GLNA1 | **Glutamine synthetase PR-1** | 64.1 | 0.970 | 5.0 | 8.0 | 15.3 | 7.0 | 0.20 |
| 363 | P39869 | NIA | **Nitrate reductase [NADH]** | 74.5 | 0.970 | 19.0 | 16.0 | 39.8 | 19.3 | 0.50 |
| 364 | Q8MCN8 | MATK | **Maturase K** | 109.3 | 0.980 | 11.0 | 7.0 | 37.2 | 58.6 | 0.40 |
| 365 | Q8MCN0 | MATK | **Maturase K** | 109.3 | 0.980 | 9.0 | 9.0 | 28.7 | 30.4 | 0.20 |
| 366 | Q8MCM3 | MATK | **Maturase K** | 103.1 | 0.980 | 8.0 | 15.0 | 38.8 | 74.4 | 0.50 |
| 367 | Q8MCK8 | MATK | **Maturase K** | 63.1 | 0.980 | 9.0 | 11.0 | 17.4 | 11.5 | 0.30 |
| 368 | Q9SLZ4 | RBR1 | **Retinoblastoma-related protein 1** | 218.7 | 0.980 | 14.0 | 26.0 | 82.3 | 92.8 | 0.30 |
| 369 | Q9FVL0 | HBL1 | **Non-symbiotic hemoglobin 1** | 79.3 | 0.980 | 4.0 | 9.0 | 20.0 | 40.4 | 0.10 |
| 370 | Q43082 | HEM3 | **Porphobilinogen deaminase, chloroplastic** | 103.5 | 0.980 | 9.0 | 10.0 | 101.1 | 81.1 | 0.40 |
| 371 | A5JTQ3 | XYL2 | **Beta-xylosidase/alpha-L-arabinofuranosidase 2** | 48.8 | 0.980 | 17.0 | 18.0 | 16.4 | 57.2 | 0.50 |
| 372 | P57997 | IF2C | **Translation initiation factor IF-2, chloroplastic** | 42.9 | 0.980 | 12.0 | 33.0 | 61.0 | 32.0 | 0.10 |
| 373 | P69590 | RBL | **Ribulose bisphosphate carboxylase large chain (Fragment)** | 115.1 | 0.980 | 5.0 | 5.0 | 34.8 | 21.0 | 0.40 |
| 374 | Q42805 | PUR3 | **Phosphoribosylglycinamide formyltransferase, chloroplastic** | 80.3 | 0.980 | 6.0 | 8.0 | 41.8 | 56.6 | 0.30 |
| 375 | Q41058 | GLGB1 | **1,4-alpha-glucan-branching enzyme 1,  chloroplastic/amyloplastic** | 58.8 | 0.980 | 19.0 | 19.0 | 6.6 | 7.2 | 0.20 |
| 376 | Q6E4Q3 | MATK | **Maturase K** | 15.8 | 0.980 | 11.0 | 10.0 | 62.4 | 17.2 | 0.20 |
| 377 | Q6DW76 | DGDG1 | **Digalactosyldiacylglycerol synthase 1, chloroplastic** | 99.8 | 0.980 | 19.0 | 19.0 | 44.8 | 59.2 | 0.30 |
| 378 | P21616 | AVP | **Pyrophosphate-energized vacuolar membrane proton pump** | 40.8 | 0.980 | 7.0 | 12.0 | 11.9 | 119.0 | 0.20 |
| 379 | Q70DJ5 | LECC1 | **Alpha-methyl-mannoside-specific lectin** | 108.9 | 0.980 | 5.0 | 6.0 | 13.1 | 100.1 | 0.10 |
| 380 | I1KEV6 | FG3H | **UDP-glycosyltransferase 79B30** | 13.4 | 0.980 | 8.0 | 9.0 | 12.1 | 125.0 | 0.30 |
| 381 | O04865 | PLDA1 | **Phospholipase D alpha 1** | 47.7 | 0.980 | 18.0 | 19.0 | 19.1 | 53.7 | 0.30 |
| 382 | P11140 | ABRA | **Abrin-a** | 62.4 | 0.990 | 12.0 | 11.0 | 48.6 | 100.6 | 0.20 |
| 383 | Q8MCM8 | MATK | **Maturase K** | 61.7 | 0.990 | 11.0 | 15.0 | 27.2 | 61.2 | 0.10 |
| 384 | Q8MCM7 | MATK | **Maturase K** | 93.8 | 0.990 | 12.0 | 10.0 | 34.5 | 58.3 | 0.40 |
| 385 | Q8MCM2 | MATK | **Maturase K** | 109.7 | 0.990 | 9.0 | 14.0 | 31.6 | 46.5 | 0.10 |
| 386 | P13916 | GLCA | **Beta-conglycinin, alpha chain** | 0.0 | 0.990 | 14.0 | 12.0 | 7.8 | 111.4 | 0.10 |
| 387 | A4GGF4 | TI214 | **Protein TIC 214** | 87.8 | 0.990 | 58.0 | 59.0 | 54.2 | 184.2 | 0.40 |
| 388 | Q8L4H4 | NORK | **Nodulation receptor kinase** | 24.2 | 0.990 | 15.0 | 14.0 | 23.2 | 70.9 | 0.40 |
| 389 | P69575 | RBL | **Ribulose bisphosphate carboxylase large chain (Fragment)** | 115.1 | 0.990 | 4.0 | 4.0 | 21.6 | 100.2 | 0.30 |
| 390 | Q5YK00 | MATK | **Maturase K** | 4.9 | 0.990 | 5.0 | 6.0 | 15.8 | 103.2 | 0.20 |
| 391 | Q53HY0 | CONB1 | **Conglutin beta 1** | 24.3 | 0.990 | 13.0 | 11.0 | 90.8 | 30.9 | 0.40 |
| 392 | P31023 | DLDH | **Dihydrolipoyl dehydrogenase, mitochondrial** | 107.1 | 0.990 | 16.0 | 11.0 | 149.5 | 58.8 | 0.50 |
| 393 | Q8MCN1 | MATK | **Maturase K** | 57.6 | 1.000 | 10.0 | 11.0 | 28.3 | 46.5 | 0.10 |
| 394 | P17067 | CAHC | **Carbonic anhydrase, chloroplastic** | 60.2 | 1.000 | 3.0 | 4.0 | 20.0 | 24.8 | 0.40 |
| 395 | P51137 | MSK1 | **Glycogen synthase kinase-3 homolog MsK-1** | 212.0 | 1.000 | 17.0 | 13.0 | 18.4 | 54.9 | 0.30 |
| 396 | Q5D1C1 | MATK | **Maturase K** | 34.9 | 1.000 | 14.0 | 13.0 | 24.6 | 84.7 | 0.20 |
| 397 | P37115 | TCMO | **Trans-cinnamate 4-monooxygenase** | 105.5 | 1.000 | 6.0 | 13.0 | 42.6 | 47.2 | 0.10 |
| 398 | Q33438 | RBL | **Ribulose bisphosphate carboxylase large chain (Fragment)** | 65.6 | 1.000 | 5.0 | 5.0 | 2.0 | 54.3 | 0.30 |
| 399 | Q40224 | GPA1 | **Guanine nucleotide-binding protein alpha-1 subunit** | 84.3 | 1.000 | 19.0 | 15.0 | 10.7 | 89.5 | 0.50 |
| 400 | Q9TKP8 | MATK | **Maturase K** | 31.7 | 1.000 | 13.0 | 12.0 | 35.3 | 69.1 | 0.30 |
| 401 | O81928 | TCMO | **Trans-cinnamate 4-monooxygenase** | 52.7 | 1.000 | 15.0 | 12.0 | 62.1 | 11.0 | 0.50 |
| 402 | Q8MCR9 | MATK | **Maturase K** | 113.7 | 1.010 | 14.0 | 16.0 | 57.1 | 83.4 | 0.50 |
| 403 | A4ULF8 | METK | **S-adenosylmethionine synthase** | 28.5 | 1.010 | 7.0 | 6.0 | 23.6 | 40.9 | 0.40 |
| 404 | Q8MCN2 | MATK | **Maturase K** | 102.9 | 1.010 | 11.0 | 5.0 | 30.4 | 147.3 | 0.20 |
| 405 | Q43078 | C97B1 | **Cytochrome P450 97B1, chloroplastic** | 73.9 | 1.010 | 12.0 | 13.0 | 76.5 | 31.2 | 0.50 |
| 406 | P69573 | RBL | **Ribulose bisphosphate carboxylase large chain (Fragment)** | 115.1 | 1.010 | 16.0 | 10.0 | 6.7 | 96.8 | 0.40 |
| 407 | Q5YK05 | MATK | **Maturase K** | 17.6 | 1.010 | 13.0 | 11.0 | 34.6 | 134.5 | 0.10 |
| 408 | Q39469 | THD1 | **Threonine dehydratase biosynthetic, chloroplastic** | 61.9 | 1.010 | 12.0 | 18.0 | 92.2 | 52.9 | 0.10 |
| 409 | P39866 | NIA2 | **Nitrate reductase [NADH] 2** | 41.6 | 1.010 | 21.0 | 22.0 | 103.3 | 167.1 | 0.30 |
| 410 | Q6WNQ9 | C81E9 | **Isoflavone 3'-hydroxylase (Fragment)** | 133.2 | 1.020 | 12.0 | 15.0 | 30.9 | 58.8 | 0.20 |
| 411 | P80366 | PPAF | **Fe(3+)-Zn(2+) purple acid phosphatase** | 49.2 | 1.020 | 7.0 | 6.0 | 48.2 | 139.3 | 0.40 |
| 412 | Q9XHM1 | EIF3C | **Eukaryotic translation initiation factor 3 subunit C** | 59.2 | 1.020 | 31.0 | 24.0 | 242.3 | 88.3 | 0.50 |
| 413 | Q9BBT0 | PBSD | **Photosystem II D2 protein** | 512.7 | 1.020 | 4.0 | 4.0 | 15.1 | 106.5 | 0.30 |
| 414 | P26969 | GCSP | **Glycine dehydrogenase (decarboxylating), mitochondrial** | 24.4 | 1.020 | 23.0 | 12.0 | 21.0 | 56.6 | 0.40 |
| 415 | P20077 | THS2 | **Putative stilbene synthase 2 (Fragment)** | 40.6 | 1.020 | 7.0 | 6.0 | 40.1 | 84.2 | 0.30 |
| 416 | Q39891 | LEU1 | **Probable 2-isopropylmalate synthase** | 102.5 | 1.020 | 13.0 | 8.0 | 16.5 | 79.9 | 0.50 |
| 417 | P08283 | H1 | **Histone H1** | 101.2 | 1.020 | 12.0 | 18.0 | 55.3 | 57.0 | 0.50 |
| 418 | P69582 | RBL | **Ribulose bisphosphate carboxylase large chain (Fragment)** | 115.1 | 1.020 | 5.0 | 5.0 | 29.4 | 39.0 | 0.10 |
| 419 | F5B8V9 | CONB1 | **Conglutin beta 1** | 64.2 | 1.020 | 9.0 | 19.0 | 33.7 | 26.1 | 0.50 |
| 420 | P58310 | PSAA | **Photosystem I P700 chlorophyll a apoprotein A1** | 81.4 | 1.020 | 10.0 | 8.0 | 40.6 | 67.7 | 0.40 |
| 421 | Q1SGF1 | PARP3 | **Putative poly [ADP-ribose] polymerase 3** | 60.7 | 1.020 | 20.0 | 19.0 | 69.3 | 50.8 | 0.20 |
| 422 | P49613 | METK | **S-adenosylmethionine synthase 2** | 28.5 | 1.020 | 6.0 | 6.0 | 44.2 | 106.1 | 0.40 |
| 423 | Q6DW74 | DGDG1 | **Digalactosyldiacylglycerol synthase 1, chloroplastic** | 99.1 | 1.020 | 14.0 | 14.0 | 275.8 | 97.3 | 0.40 |
| 424 | Q9SWR5 | C93C1 | **2-hydroxyisoflavanone synthase** | 23.5 | 1.020 | 11.0 | 10.0 | 24.0 | 118.7 | 0.50 |
| 425 | Q9TKS6 | MATK | **Maturase K** | 111.1 | 1.020 | 11.0 | 8.0 | 49.3 | 75.9 | 0.20 |
| 426 | Q8HUG7 | MATK | **Maturase K** | 48.3 | 1.030 | 13.0 | 7.0 | 45.4 | 24.5 | 0.20 |
| 427 | O24534 | EF1A | **Elongation factor 1-alpha** | 77.5 | 1.030 | 10.0 | 14.0 | 151.3 | 77.2 | 0.40 |
| 428 | A4GGA7 | RPOC2 | **DNA-directed RNA polymerase subunit beta** | 40.4 | 1.030 | 30.0 | 25.0 | 74.0 | 91.4 | 0.50 |
| 429 | P39865 | NIA1 | **Nitrate reductase [NADH] 1** | 50.7 | 1.030 | 17.0 | 15.0 | 21.5 | 102.6 | 0.40 |
| 430 | Q9TKS1 | MATK | **Maturase K** | 37.3 | 1.030 | 13.0 | 14.0 | 41.8 | 131.2 | 0.10 |
| 431 | P50477 | CANA | **Canavalin** | 4.6 | 1.030 | 8.0 | 8.0 | 71.6 | 123.6 | 0.50 |
| 432 | Q2PMU2 | PSAB | **Photosystem I P700 chlorophyll a apoprotein A2** | 40.8 | 1.041 | 3.0 | 7.0 | 49.1 | 96.2 | 0.40 |
| 433 | Q43468 | HSOP1 | **Hsp70-Hsp90 organizing protein 1** | 95.0 | 1.041 | 23.0 | 23.0 | 69.7 | 184.9 | 0.50 |
| 434 | G7JND3 | CN15B | **Protein CNGC15b** | 63.3 | 1.041 | 6.0 | 9.0 | 38.1 | 178.3 | 0.20 |
| 435 | A4GGA2 | PSBD | **Photosystem II D2 protein** | 512.7 | 1.041 | 6.0 | 5.0 | 35.8 | 131.9 | 0.10 |
| 436 | P69581 | RBL | **Ribulose bisphosphate carboxylase large chain (Fragment)** | 115.1 | 1.041 | 11.0 | 12.0 | 38.8 | 127.4 | 0.20 |
| 437 | P32296 | P5CS | **Delta-1-pyrroline-5-carboxylate synthase** | 72.2 | 1.041 | 21.0 | 19.0 | 116.3 | 63.1 | 0.30 |
| 438 | Q5YJU1 | MATK | **Maturase K** | 87.4 | 1.041 | 13.0 | 14.0 | 12.1 | 59.1 | 0.50 |
| 439 | Q04593 | PAL2 | **Phenylalanine ammonia-lyase 2** | 90.2 | 1.041 | 14.0 | 16.0 | 38.2 | 98.0 | 0.10 |
| 440 | P42500 | PHYA | **Phytochrome A** | 51.8 | 1.041 | 28.0 | 19.0 | 30.6 | 135.7 | 0.30 |
| 441 | Q8MCM0 | MATK | **Maturase K** | 91.2 | 1.051 | 11.0 | 18.0 | 53.4 | 106.5 | 0.30 |
| 442 | P48406 | CHS5 | **Chalcone synthase 5** | 18.9 | 1.051 | 4.0 | 6.0 | 108.8 | 71.3 | 0.40 |
| 443 | Q84KK6 | I4OMT | **Isoflavone 4'-O-methyltransferase** | 160.1 | 1.051 | 11.0 | 7.0 | 77.5 | 66.7 | 0.50 |
| 444 | P51109 | DFRA | **Dihydroflavonol 4-reductase (Fragment)** | 88.3 | 1.051 | 5.0 | 11.0 | 12.5 | 84.5 | 0.10 |
| 445 | O98997 | RCA | **Ribulose bisphosphate carboxylase/oxygenase activase,  chloroplastic** | 36.1 | 1.051 | 10.0 | 9.0 | 38.0 | 89.7 | 0.50 |
| 446 | P10562 | CANA | **Canavalin** | 4.6 | 1.051 | 7.0 | 10.0 | 21.0 | 99.7 | 0.10 |
| 447 | P69588 | RBL | **Ribulose bisphosphate carboxylase large chain (Fragment)** | 115.1 | 1.051 | 11.0 | 11.0 | 28.6 | 101.0 | 0.20 |
| 448 | P08215 | ATPA | **ATP synthase subunit alpha, chloroplastic** | 40.3 | 1.051 | 8.0 | 12.0 | 114.4 | 175.8 | 0.20 |
| 449 | O62970 | RBL | **Ribulose bisphosphate carboxylase large chain (Fragment)** | 36.6 | 1.051 | 6.0 | 4.0 | 96.1 | 91.5 | 0.30 |
| 450 | B5WWZ8 | FAO1 | **Long-chain-alcohol oxidase FAO1** | 105.3 | 1.051 | 17.0 | 16.0 | 49.7 | 50.0 | 0.10 |
| 451 | Q9TKP9 | MATK | **Maturase K** | 47.7 | 1.051 | 11.0 | 9.0 | 34.6 | 57.9 | 0.50 |
| 452 | Q03460 | GLSN | **Glutamate synthase [NADH], amyloplastic** | 40.1 | 1.062 | 52.0 | 47.0 | 102.0 | 119.7 | 0.20 |
| 453 | Q8MCN7 | MATK | **Maturase K** | 104.2 | 1.062 | 12.0 | 10.0 | 25.2 | 45.8 | 0.30 |
| 454 | Q2PMS8 | ATPA | **ATP synthase subunit alpha, chloroplastic** | 126.4 | 1.062 | 11.0 | 8.0 | 47.3 | 54.5 | 0.40 |
| 455 | P08926 | RUBA | **RuBisCO large subunit-binding protein subunit alpha,  chloroplastic** | 33.6 | 1.062 | 14.0 | 18.0 | 83.3 | 44.5 | 0.10 |
| 456 | P30706 | PLSB | **Glycerol-3-phosphate acyltransferase, chloroplastic** | 17.7 | 1.062 | 9.0 | 9.0 | 16.9 | 50.2 | 0.50 |
| 457 | P69578 | RBL | **Ribulose bisphosphate carboxylase large chain (Fragment)** | 115.1 | 1.062 | 4.0 | 4.0 | 29.7 | 108.5 | 0.40 |
| 458 | P69576 | RBL | **Ribulose bisphosphate carboxylase large chain (Fragment)** | 115.1 | 1.062 | 7.0 | 6.0 | 57.3 | 144.8 | 0.40 |
| 459 | F5B8W2 | CONB4 | **Conglutin beta 4** | 106.6 | 1.062 | 24.0 | 14.0 | 13.1 | 121.5 | 0.10 |
| 460 | P49612 | METK1 | **S-adenosylmethionine synthase 1 (Fragment)** | 76.5 | 1.062 | 5.0 | 5.0 | 129.0 | 85.0 | 0.20 |
| 461 | Q02735 | CAPP | **Phosphoenolpyruvate carboxylase** | 52.3 | 1.062 | 37.0 | 23.0 | 12.3 | 111.9 | 0.30 |
| 462 | P31687 | 4CL2 | **4-coumarate-CoA ligase 2** | 48.9 | 1.073 | 13.0 | 14.0 | 96.1 | 62.0 | 0.50 |
| 463 | Q02920 | NO70 | **Early nodulin-70** | 21.0 | 1.073 | 14.0 | 12.0 | 52.5 | 101.5 | 0.30 |
| 464 | Q9BBQ8 | PSBB | **Photosystem II CP47 reaction center protein** | 24.5 | 1.073 | 17.0 | 10.0 | 85.6 | 71.0 | 0.50 |
| 465 | P69587 | RBL | **Ribulose bisphosphate carboxylase large chain (Fragment)** | 115.1 | 1.073 | 2.0 | 10.0 | 44.0 | 100.5 | 0.50 |
| 466 | P69585 | RBL | **Ribulose bisphosphate carboxylase large chain (Fragment)** | 115.1 | 1.073 | 5.0 | 17.0 | 42.0 | 61.7 | 0.50 |
| 467 | Q01288 | CHS6 | **Chalcone synthase 6** | 34.8 | 1.083 | 10.0 | 12.0 | 38.3 | 101.4 | 0.10 |
| 468 | Q96558 | UGDH1 | **UDP-glucose 6-dehydrogenase 1** | 82.3 | 1.083 | 16.0 | 12.0 | 68.1 | 53.6 | 0.40 |
| 469 | Q40106 | TBB2 | **Tubulin beta-2 chain** | 171.1 | 1.083 | 8.0 | 7.0 | 40.3 | 45.6 | 0.20 |
| 470 | Q8SKU2 | TIC62 | **Protein TIC 62, chloroplastic** | 99.1 | 1.083 | 4.0 | 7.0 | 12.0 | 35.6 | 0.20 |
| 471 | Q9BBN6 | TI214 | **Protein TIC 214** | 83.5 | 1.083 | 72.0 | 73.0 | 23.8 | 96.6 | 0.40 |
| 472 | Q39817 | CALX | **Calnexin homolog** | 85.5 | 1.083 | 14.0 | 17.0 | 138.5 | 63.4 | 0.20 |
| 473 | C6TEX6 | FEN1 | **Flap endonuclease 1** | 36.9 | 1.083 | 11.0 | 5.0 | 70.9 | 105.5 | 0.10 |
| 474 | P69586 | RBL | **Ribulose bisphosphate carboxylase large chain (Fragment)** | 115.1 | 1.083 | 4.0 | 4.0 | 23.4 | 63.3 | 0.10 |
| 475 | P69577 | RBL | **Ribulose bisphosphate carboxylase large chain (Fragment)** | 115.1 | 1.083 | 14.0 | 5.0 | 63.7 | 102.3 | 0.10 |
| 476 | F5B8W4 | CONB6 | **Conglutin beta 6** | 102.7 | 1.083 | 24.0 | 17.0 | 19.6 | 31.8 | 0.30 |
| 477 | Q41706 | UPSA3 | **Probable ureide permease A3 (Fragment)** | 158.3 | 1.083 | 5.0 | 4.0 | 15.1 | 35.2 | 0.30 |
| 478 | P92407 | RBL | **Ribulose bisphosphate carboxylase large chain (Fragment)** | 115.1 | 1.083 | 2.0 | 12.0 | 28.5 | 66.0 | 0.50 |
| 479 | P92401 | RBL | **Ribulose bisphosphate carboxylase large chain (Fragment)** | 115.1 | 1.083 | 6.0 | 6.0 | 66.9 | 72.0 | 0.10 |
| 480 | Q9ZNX6 | GSH1 | **Glutamate-cysteine ligase, chloroplastic** | 43.3 | 1.083 | 13.0 | 12.0 | 77.1 | 52.5 | 0.20 |
| 481 | P24826 | CHS1 | **Chalcone synthase 1** | 18.9 | 1.094 | 4.0 | 4.0 | 35.7 | 61.5 | 0.10 |
| 482 | Q84KK4 | I4OMT | **Isoflavone 4'-O-methyltransferase** | 219.5 | 1.094 | 5.0 | 9.0 | 23.1 | 56.3 | 0.20 |
| 483 | A0A172J2G3 | UGT43 | **UDP-glycosyltransferase 43** | 68.4 | 1.094 | 8.0 | 8.0 | 25.6 | 188.8 | 0.30 |
| 484 | Q41107 | INO1 | **Inositol-3-phosphate synthase** | 46.0 | 1.094 | 10.0 | 6.0 | 34.5 | 163.7 | 0.20 |
| 485 | A4GG98 | PSAB | **Photosystem I P700 chlorophyll a apoprotein A2** | 11.6 | 1.094 | 1.0 | 9.0 | 19.0 | 30.8 | 0.40 |
| 486 | Q40313 | CAMT | **Caffeoyl-CoA O-methyltransferase** | 57.8 | 1.094 | 9.0 | 6.0 | 30.2 | 94.3 | 0.20 |
| 487 | P92406 | RBL | **Ribulose bisphosphate carboxylase large chain (Fragment)** | 115.1 | 1.094 | 5.0 | 5.0 | 10.1 | 57.8 | 0.30 |
| 488 | O64407 | AMYB | **Beta-amylase** | 84.2 | 1.094 | 5.0 | 8.0 | 85.2 | 56.5 | 0.50 |
| 489 | O04408 | KSA | **Ent-copalyl diphosphate synthase, chloroplastic** | 43.2 | 1.105 | 14.0 | 22.0 | 87.9 | 65.3 | 0.20 |
| 490 | P07218 | PAL1 | **Phenylalanine ammonia-lyase class 1 (Fragment)** | 77.5 | 1.105 | 19.0 | 18.0 | 66.5 | 60.7 | 0.30 |
| 491 | B8R4B1 | NU5C | **NAD(P)H-quinone oxidoreductase subunit5C; chloroplastic** | 15.9 | 1.105 | 6.0 | 6.0 | 15.1 | 43.6 | 0.50 |
| 492 | D2XNQ9 | FLOT2 | **Flotillin-like protein 2** | 13.7 | 1.105 | 15.0 | 13.0 | 31.3 | 114.8 | 0.40 |
| 493 | P49082 | GPA1 | **Guanine nucleotide-binding protein alpha-1 subunit** | 104.0 | 1.105 | 11.0 | 13.0 | 72.0 | 101.6 | 0.20 |
| 494 | Q5YK33 | MATK | **Maturase K** | 54.4 | 1.105 | 17.0 | 12.0 | 61.7 | 127.4 | 0.40 |
| 495 | Q42806 | KPYC | **Pyruvate kinase, cytosolic isozyme** | 94.4 | 1.105 | 13.0 | 17.0 | 36.8 | 97.0 | 0.20 |
| 496 | P08863 | NO26B | **Nodulin-26B** | 36.0 | 1.105 | 5.0 | 2.0 | 38.4 | 50.9 | 0.20 |
| 497 | P14856 | LOX2 | **Seed linoleate 9S-lipoxygenase-2** | 75.1 | 1.105 | 25.0 | 20.0 | 54.6 | 46.0 | 0.50 |
| 498 | O22585 | AMYB | **Beta-amylase** | 12.4 | 1.105 | 10.0 | 9.0 | 24.6 | 86.2 | 0.20 |
| 499 | P08170 | LOX1 | **Seed linoleate 13S-lipoxygenase-1** | 30.5 | 1.105 | 20.0 | 12.0 | 14.3 | 147.7 | 0.50 |
| 500 | P35100 | CLPC | **Chaperone protein ClpC, chloroplastic** | 170.8 | 1.105 | 18.0 | 27.0 | 52.7 | 86.4 | 0.40 |
| 501 | P37900 | HSP7M | **Heat shock 70 kDa protein, mitochondrial** | 67.5 | 1.105 | 24.0 | 13.0 | 49.2 | 709.0 | 0.20 |
| 502 | Q43460 | NO20A | **Nodulin-20a** | 124.7 | 1.116 | 3.0 | 4.0 | 78.8 | 142.7 | 0.50 |
| 503 | Q9ZSK5 | ZOG | **Zeatin O-glucosyltransferase** | 35.0 | 1.116 | 9.0 | 10.0 | 56.7 | 60.7 | 0.20 |
| 504 | P98188 | C94A2 | **Cytochrome P450 94A2 (Fatty acid biosynthesis, Plamitate,  laurate C10-C18)** | 60.2 | 1.116 | 6.0 | 8.0 | 30.1 | 37.9 | 0.30 |
| 505 | Q6PP79 | MATK | **Maturase K** | 4.0 | 1.116 | 8.0 | 12.0 | 15.1 | 63.3 | 0.30 |
| 506 | P49044 | VPE | **Vacuolar-processing enzyme** | 88.4 | 1.116 | 5.0 | 7.0 | 20.3 | 85.4 | 0.10 |
| 507 | F5B8W0 | CONB2 | **Conglutin beta 2** | 117.7 | 1.116 | 15.0 | 16.0 | 50.3 | 107.7 | 0.30 |
| 508 | Q2PMP0 | TI214 | **Protein TIC 214** | 101.6 | 1.127 | 68.0 | 68.0 | 20.3 | 150.7 | 0.20 |
| 509 | Q9BBS7 | RPOC2 | **DNA-directed RNA polymerase subunit beta** | 25.4 | 1.127 | 34.0 | 31.0 | 93.0 | 175.5 | 0.30 |
| 510 | Q43089 | INV1 | **Beta-fructofuranosidase, cell wall isozyme** | 14.6 | 1.127 | 10.0 | 9.0 | 9.3 | 122.1 | 0.10 |
| 511 | D2XNQ8 | FLOT1 | **Flotillin-like protein 1** | 184.9 | 1.127 | 16.0 | 11.0 | 34.5 | 50.3 | 0.40 |
| 512 | Q5YJX5 | MATK | **Maturase K** | 36.0 | 1.127 | 11.0 | 15.0 | 31.2 | 136.5 | 0.10 |
| 513 | Q09WE7 | USP1 | **UDP-sugar pyrophosphorylase 1** | 68.6 | 1.127 | 12.0 | 6.0 | 50.7 | 54.1 | 0.30 |
| 514 | Q5NUF4 | HIDM | **2-hydroxyisoflavanone dehydratase** | 15.0 | 1.127 | 5.0 | 6.0 | 67.2 | 81.2 | 0.30 |
| 515 | Q9LRH7 | ABAMS | **Mixed-amyrin synthase** | 100.5 | 1.139 | 20.0 | 23.0 | 67.8 | 94.7 | 0.50 |
| 516 | Q00016 | IFR | **Isoflavone reductase** | 61.9 | 1.139 | 7.0 | 10.0 | 55.5 | 123.0 | 0.50 |
| 517 | P48621 | FAD3C | **Omega-3 fatty acid desaturase, chloroplastic** | 348.5 | 1.139 | 9.0 | 8.0 | 83.2 | 49.5 | 0.40 |
| 518 | Q43621 | GSHRC | **Glutathione reductase, cytosolic** | 45.0 | 1.139 | 12.0 | 19.0 | 71.9 | 40.5 | 0.20 |
| 519 | P69583 | RBL | **Ribulose bisphosphate carboxylase large chain (Fragment)** | 115.1 | 1.139 | 14.0 | 5.0 | 99.4 | 42.8 | 0.30 |
| 520 | P49084 | GPA1 | **Guanine nucleotide-binding protein alpha-1 subunit** | 50.9 | 1.139 | 15.0 | 13.0 | 37.3 | 60.5 | 0.40 |
| 521 | P51062 | CAPP | **Phosphoenolpyruvate carboxylase** | 33.9 | 1.150 | 34.0 | 21.0 | 115.0 | 35.7 | 0.50 |
| 522 | P32293 | AX22A | **Auxin-induced protein 22A** | 77.2 | 1.150 | 6.0 | 5.0 | 24.4 | 20.0 | 0.40 |
| 523 | P69574 | RBL | **Ribulose bisphosphate carboxylase large chain (Fragment)** | 115.1 | 1.150 | 12.0 | 11.0 | 42.7 | 58.5 | 0.20 |
| 524 | Q06215 | PPO | **Polyphenol oxidase A1, chloroplastic** | 34.8 | 1.150 | 16.0 | 12.0 | 28.5 | 100.0 | 0.40 |
| 525 | Q9SAZ1 | LGB4 | **Leghemoglobin Lb120-8** | 150.8 | 1.150 | 5.0 | 7.0 | 19.5 | 106.5 | 0.10 |
| 526 | Q40353 | MMK2 | **Mitogen-activated protein kinase homolog MMK2** | 138.0 | 1.162 | 11.0 | 10.0 | 32.2 | 122.7 | 0.20 |
| 527 | C6TAY1 | SOMT2 | **Flavonoid 4'-O-methyltransferase** | 30.8 | 1.162 | 5.0 | 7.0 | 21.5 | 72.8 | 0.20 |
| 528 | Q01912 | 1A1C | **1-aminocyclopropane-1-carboxylate synthase (Fragment)** | 0.0 | 1.162 | 12.0 | 10.0 | 18.5 | 45.1 | 0.50 |
| 529 | P17957 | CHS2 | **Chalcone synthase 2** | 18.9 | 1.174 | 14.0 | 11.0 | 73.7 | 114.8 | 0.30 |
| 530 | P09918 | LOX3 | **Seed linoleate 9S-lipoxygenase-3** | 40.6 | 1.174 | 20.0 | 15.0 | 70.7 | 72.5 | 0.20 |
| 531 | P07374 | UREA | **Urease** | 39.6 | 1.174 | 20.0 | 14.0 | 45.5 | 97.1 | 0.50 |
| 532 | P09186 | LOX3 | **Seed linoleate 9S-lipoxygenase-3** | 67.5 | 1.185 | 20.0 | 18.0 | 7.0 | 43.6 | 0.50 |
| 533 | P08960 | NO20A | **Nodulin-20** | 5.4 | 1.185 | 4.0 | 4.0 | 41.8 | 75.5 | 0.40 |
| 534 | Q43066 | GLNA4 | **Glutamine synthetase root isozyme B** | 16.3 | 1.197 | 1.0 | 7.0 | 28.0 | 57.2 | 0.20 |
| 535 | Q6RHR6 | DMI1 | **Ion channel DMI1** | 53.2 | 1.197 | 22.0 | 14.0 | 92.9 | 87.0 | 0.50 |
| 536 | O49816 | LEA1 | **Late embryogenesis abundant protein 1** | 46.0 | 1.197 | 5.0 | 9.0 | 34.0 | 35.7 | 0.40 |
| 537 | P54233 | NIA1 | **Inducible nitrate reductase [NADH] 1** | 31.1 | 1.197 | 17.0 | 16.0 | 93.3 | 93.5 | 0.30 |
| 538 | P02855 | VCLA | **Provicilin (Fragment)** | 68.4 | 1.197 | 10.0 | 12.0 | 98.0 | 40.7 | 0.30 |
| 539 | O24310 | EFTU | **Elongation factor Tu, chloroplastic** | 8.8 | 1.209 | 10.0 | 6.0 | 54.4 | 57.7 | 0.20 |
| 540 | D2XNR1 | FLOT4 | **Flotillin-like protein 4** | 26.4 | 1.209 | 17.0 | 13.0 | 87.8 | 44.8 | 0.40 |
| 541 | Q39828 | SDL5A | **Dynamin-related protein 5A** | 121.1 | 1.209 | 16.0 | 11.0 | 40.7 | 518.0 | 0.20 |
| 542 | P19142 | PAL2 | **Phenylalanine ammonia-lyase class 2** | 80.3 | 1.209 | 12.0 | 16.0 | 47.9 | 58.7 | 0.30 |
| 543 | P52423 | PUR3 | **Phosphoribosylglycinamide formyltransferase, chloroplastic** | 4.8 | 1.209 | 5.0 | 5.0 | 42.5 | 13.3 | 0.10 |
| 544 | P27990 | PALY | **Phenylalanine ammonia-lyase** | 219.8 | 1.209 | 14.0 | 16.0 | 36.6 | 92.4 | 0.30 |
| 545 | Q9B133 | RR12 | **30S ribosomal protein S12, chloroplastic** | 62.2 | 1.209 | 11.0 | 7.0 | 19.0 | 34.0 | 0.40 |
| 546 | P05493 | ATPAM | **ATP synthase subunit alpha, mitochondrial** | 113.4 | 1.209 | 18.0 | 12.0 | 7.2 | 27.8 | 0.10 |
| 547 | Q9SMK9 | PAL2 | **Phenylalanine ammonia-lyase 2** | 19.8 | 1.221 | 12.0 | 23.0 | 24.1 | 65.1 | 0.30 |
| 548 | P25890 | CATA | **Catalase** | 29.5 | 1.221 | 15.0 | 10.0 | 80.9 | 109.0 | 0.40 |
| 549 | A4GGC9 | RR12 | **30S ribosomal protein S12, chloroplastic** | 53.9 | 1.221 | 4.0 | 3.0 | 52.0 | 128.0 | 0.30 |
| 550 | A4GGA6 | RPOC1 | **DNA-directed RNA polymerase subunit beta** | 48.7 | 1.221 | 16.0 | 19.0 | 36.7 | 21.5 | 0.10 |
| 551 | O49856 | FTRC | **Ferredoxin-thioredoxin reductase catalytic chain, chloroplastic** | 2.0 | 1.221 | 2.0 | 3.0 | 82.3 | 83.6 | 0.40 |
| 552 | P38417 | LOX4 | **Linoleate 9S-lipoxygenase-4** | 56.1 | 1.221 | 22.0 | 21.0 | 174.7 | 267.2 | 0.20 |
| 553 | P52780 | SYQ | **Glutamine-tRNA ligase** | 31.7 | 1.234 | 18.0 | 27.0 | 33.0 | 71.0 | 0.30 |
| 554 | P27481 | LOXB | **Linoleate 9S-lipoxygenase (Fragment)** | 85.4 | 1.246 | 18.0 | 18.0 | 85.2 | 136.3 | 0.50 |
| 555 | Q84N37 | PVIP | **OBERON-like protein (Fragment)** | 128.5 | 1.246 | 19.0 | 12.0 | 10.5 | 62.6 | 0.20 |
| 556 | Q8HVY4 | RPOC1 | **DNA-directed RNA polymerase subunit beta** | 44.6 | 1.246 | 13.0 | 13.0 | 13.9 | 67.0 | 0.40 |
| 557 | P24095 | LOXX | **Seed linoleate 9S-lipoxygenase** | 45.9 | 1.259 | 15.0 | 23.0 | 48.7 | 105.6 | 0.30 |
| 558 | P52416 | GLGS1 | **Glucose-1-phosphate adenylyltransferase small subunit 1,  chloroplastic** | 103.9 | 1.259 | 10.0 | 9.0 | 29.3 | 88.3 | 0.30 |
| 559 | P07134 | RR12 | **30S ribosomal protein S12, chloroplastic** | 59.9 | 1.259 | 5.0 | 15.0 | 178.1 | 63.2 | 0.50 |
| 560 | P11827 | GLCAP | **Beta-conglycinin, alpha' chain** | 36.2 | 1.259 | 20.0 | 10.0 | 55.9 | 71.4 | 0.10 |
| 561 | Q01899 | HSP7M | **Heat shock 70 kDa protein, mitochondrial** | 44.6 | 1.271 | 19.0 | 22.0 | 26.4 | 101.3 | 0.40 |
| 562 | O49931 | TIC55 | **Protein TIC 55, chloroplastic** | 153.3 | 1.271 | 18.0 | 16.0 | 130.6 | 123.1 | 0.10 |
| 563 | Q9BBS8 | RPOC1 | **DNA-directed RNA polymerase subunit beta** | 63.5 | 1.271 | 21.0 | 12.0 | 48.5 | 37.6 | 0.40 |
| 564 | Q43088 | RBCMT | **Ribulose-1,5 bisphosphate carboxylase/oxygenase  large subunit N-methyltransferase, chloroplastic** | 81.6 | 1.271 | 7.0 | 11.0 | 34.5 | 24.3 | 0.10 |
| 565 | P22895 | P34 | **P34 probable thiol protease** | 67.3 | 1.271 | 9.0 | 8.0 | 27.2 | 160.3 | 0.50 |
| 566 | P15001 | PHYA | **Phytochrome A** | 33.5 | 1.271 | 23.0 | 25.0 | 67.2 | 551.4 | 0.10 |
| 567 | Q69F95 | C85A | **Cytochrome P450 85A (BA synthesis)** | 95.4 | 1.271 | 19.0 | 11.0 | 49.4 | 134.0 | 0.50 |
| 568 | O24304 | FNTA | **Protein farnesyltransferase/geranylgeranyltransferase type-1  subunit alpha** | 40.7 | 1.284 | 7.0 | 12.0 | 38.7 | 144.8 | 0.10 |
| 569 | D2XNR2 | FLOT6 | **Flotillin-like protein 6** | 99.1 | 1.284 | 6.0 | 12.0 | 18.0 | 78.2 | 0.10 |
| 570 | P29502 | TBB3 | **Tubulin beta-3 chain (Fragment)** | 3.8 | 1.284 | 6.0 | 12.0 | 14.0 | 39.3 | 0.40 |
| 571 | O49818 | LGUL | **Lactoylglutathione lyase** | 67.3 | 1.284 | 7.0 | 5.0 | 97.5 | 52.3 | 0.40 |
| 572 | P30080 | CHS6 | **Chalcone synthase 6** | 18.9 | 1.284 | 10.0 | 11.0 | 317.7 | 350.4 | 0.30 |
| 573 | P37392 | TBB1 | **Tubulin beta-1 chain** | 3.8 | 1.284 | 11.0 | 12.0 | 18.2 | 55.0 | 0.40 |
| 574 | Q8W3Y4 | METK | **S-adenosylmethionine synthase** | 131.0 | 1.284 | 2.0 | 7.0 | 36.5 | 33.0 | 0.20 |
| 575 | Q93XK2 | STSYN | **Stachyose synthase** | 58.5 | 1.297 | 19.0 | 8.0 | 22.0 | 109.2 | 0.20 |
| 576 | O24308 | TOP2 | **DNA topoisomerase 2** | 115.3 | 1.297 | 50.0 | 49.0 | 81.1 | 99.3 | 0.10 |
| 577 | P28551 | TBB3 | **Tubulin beta chain (Fragment)** | 3.8 | 1.297 | 7.0 | 7.0 | 15.0 | 23.6 | 0.30 |
| 578 | P30164 | ACT1 | **Actin-1** | 40.4 | 1.297 | 15.0 | 2.0 | 15.7 | 38.0 | 0.20 |
| 579 | Q41651 | CYPB | **Peptidyl-prolyl cis-trans isomerase, chloroplastic** | 89.6 | 1.297 | 6.0 | 7.0 | 29.1 | 196.2 | 0.30 |
| 580 | P07694 | GLNA3 | **Glutamine synthetase root isozyme A** | 34.1 | 1.297 | 5.0 | 6.0 | 44.7 | 37.9 | 0.20 |
| 581 | P29450 | TRXF | **Thioredoxin F-type, chloroplastic** | 37.5 | 1.310 | 5.0 | 9.0 | 49.7 | 54.2 | 0.50 |
| 582 | P13919 | CVCB | **Convicilin (Fragment)** | 85.9 | 1.323 | 13.0 | 10.0 | 38.4 | 33.3 | 0.10 |
| 583 | P45732 | PALY | **Phenylalanine ammonia-lyase** | 78.1 | 1.323 | 10.0 | 14.0 | 32.9 | 70.4 | 0.20 |
| 584 | P53537 | PHSH | **Alpha-glucan phosphorylase, H isozyme** | 44.9 | 1.323 | 10.0 | 9.0 | 7.5 | 73.9 | 0.10 |
| 585 | Q41011 | EF1A | **Elongation factor 1-alpha** | 83.0 | 1.323 | 11.0 | 17.0 | 65.1 | 208.2 | 0.20 |
| 586 | P53392 | SUT2 | **High affinity sulfate transporter 2** | 150.1 | 1.336 | 18.0 | 10.0 | 4.4 | 22.6 | 0.20 |
| 587 | Q5H8A6 | CASTO | **Ion channel CASTOR** | 20.0 | 1.336 | 12.0 | 15.0 | 35.1 | 21.5 | 0.40 |
| 588 | H1A981 | C7263 | **11-oxo-beta-amyrin 30-oxidase** | 62.8 | 1.336 | 13.0 | 10.0 | 13.5 | 73.1 | 0.10 |
| 589 | Q8VWN6 | RFS | **Galactinol-sucrose galactosyltransferase** | 22.1 | 1.336 | 15.0 | 14.0 | 11.0 | 70.9 | 0.10 |
| 590 | Q6PSB9 | MATK | **Maturase K** | 41.3 | 1.336 | 7.0 | 7.0 | 5.6 | 95.0 | 0.10 |
| 591 | Q6PP78 | MATK | **Maturase K** | 35.8 | 1.336 | 11.0 | 11.0 | 9.5 | 41.6 | 0.10 |
| 592 | G1CWH1 | CYC2 | **Cliotide T2** | 194.9 | 1.336 | 3.0 | 6.0 | 16.8 | 14.5 | 0.20 |
| 593 | P27991 | PAL1 | **Phenylalanine ammonia-lyase 1** | 144.0 | 1.350 | 14.0 | 22.0 | 11.5 | 56.6 | 0.40 |
| 594 | P34798 | URIC1 | **Uricase-2 isozyme 1** | 35.0 | 1.350 | 10.0 | 10.0 | 25.6 | 134.5 | 0.20 |
| 595 | P53391 | SUT1 | **High affinity sulfate transporter 1** | 204.0 | 1.363 | 4.0 | 13.0 | 23.9 | 13.3 | 0.40 |
| 596 | Q43077 | AMO | **Primary amine oxidase** | 9.0 | 1.363 | 6.0 | 17.0 | 43.5 | 91.0 | 0.40 |
| 597 | Q5NUF3 | HIDH | **2-hydroxyisoflavanone dehydratase** | 50.4 | 1.363 | 5.0 | 3.0 | 21.8 | 77.2 | 0.10 |
| 598 | P13911 | RPOA | **DNA-directed RNA polymerase subunit alpha** | 236.1 | 1.377 | 17.0 | 7.0 | 27.2 | 6.7 | 0.20 |
| 599 | Q8GT66 | TIC40 | **Protein TIC 40, chloroplastic** | 204.6 | 1.391 | 5.0 | 11.0 | 11.0 | 66.5 | 0.50 |
| 600 | Q6J541 | C79D3 | **Isoleucine N-monooxygenase 1** | 43.2 | 1.391 | 18.0 | 14.0 | 7.2 | 65.6 | 0.50 |
| 601 | D4Q9Z4 | SGT2 | **Soyasapogenol B glucuronide galactosyltransferase** | 56.3 | 1.391 | 17.0 | 15.0 | 11.9 | 124.0 | 0.30 |
| 602 | Q39857 | XTH1 | **Xyloglucan endotransglucosylase/hydrolase 1** | 53.6 | 1.391 | 8.0 | 6.0 | 4.0 | 76.0 | 0.10 |
| 603 | P10538 | AMYB | **Beta-amylase** | 59.0 | 1.391 | 6.0 | 7.0 | 24.8 | 45.6 | 0.10 |
| 604 | P32289 | GLNA | **Glutamine synthetase nodule isozyme** | 40.8 | 1.391 | 5.0 | 5.0 | 22.5 | 60.0 | 0.40 |
| 605 | Q9BAE0 | FTSH | **ATP-dependent zinc metalloprotease FTSH, chloroplastic** | 56.1 | 1.391 | 17.0 | 11.0 | 40.3 | 27.5 | 0.30 |
| 606 | P08438 | VCL | **Vicilin** | 77.9 | 1.391 | 14.0 | 10.0 | 31.5 | 72.9 | 0.30 |
| 607 | P30165 | ACT2 | **Actin-2** | 51.7 | 1.405 | 7.0 | 9.0 | 26.4 | 86.4 | 0.10 |
| 608 | Q43467 | EFTU1 | **Elongation factor Tu, chloroplastic** | 62.9 | 1.419 | 7.0 | 12.0 | 33.3 | 11.4 | 0.30 |
| 609 | P29531 | OLEO2 | **P24 oleosin isoform B** | 107.3 | 1.419 | 7.0 | 7.0 | 27.6 | 31.0 | 0.30 |
| 610 | P45734 | PALY | **Phenylalanine ammonia-lyase** | 86.9 | 1.433 | 19.0 | 24.0 | 14.7 | 63.4 | 0.50 |
| 611 | Q948P6 | FRI3 | **Ferritin-3, chloroplastic** | 108.8 | 1.433 | 6.0 | 8.0 | 22.6 | 78.7 | 0.50 |
| 612 | A5JTQ2 | XYL1 | **Beta-xylosidase/alpha-L-arabinofuranosidase 1 (Fragment)** | 49.2 | 1.433 | 18.0 | 9.0 | 15.1 | 7.4 | 0.30 |
| 613 | O48922 | C98A2 | **Cytochrome P450 98A2** | 77.6 | 1.433 | 18.0 | 13.0 | 80.3 | 138.3 | 0.30 |
| 614 | P08688 | ALB2 | **Albumin-2** | 50.7 | 1.448 | 6.0 | 5.0 | 4.9 | 62.0 | 0.30 |
| 615 | O04278 | GPA1 | **Guanine nucleotide-binding protein alpha-1 subunit** | 70.1 | 1.448 | 14.0 | 16.0 | 13.0 | 53.7 | 0.30 |
| 616 | P12886 | ADH1 | **Alcohol dehydrogenase 1** | 17.5 | 1.462 | 3.0 | 6.0 | 16.8 | 53.7 | 0.50 |
| 617 | P31531 | 1A1C | **1-aminocyclopropane-1-carboxylate synthase** | 2.3 | 1.462 | 9.0 | 6.0 | 16.7 | 36.7 | 0.20 |
| 618 | P12468 | RBS4 | **Ribulose bisphosphate carboxylase small chain 4, chloroplastic** | 102.0 | 1.462 | 3.0 | 6.0 | 18.6 | 33.2 | 0.40 |
| 619 | Q94G16 | TATB | **Sec-independent protein translocase protein TATB,  chloroplastic** | 85.7 | 1.462 | 7.0 | 6.0 | 11.0 | 101.3 | 0.50 |
| 620 | P34899 | GLYM | **Serine hydroxymethyltransferase, mitochondrial** | 92.3 | 1.477 | 12.0 | 13.0 | 19.1 | 178.8 | 0.50 |
| 621 | P52418 | PUR1 | **Amidophosphoribosyltransferase, chloroplastic** | 43.1 | 1.477 | 7.0 | 10.0 | 39.5 | 31.2 | 0.20 |
| 622 | C0HJB3 | MANA | **Alpha-mannosidase** | 38.2 | 1.477 | 9.0 | 21.0 | 24.0 | 62.8 | 0.10 |
| 623 | Q43138 | MTDH3 | **Probable mannitol dehydrogenase 3** | 122.0 | 1.492 | 5.0 | 7.0 | 29.0 | 7.2 | 0.50 |
| 624 | Q5UB07 | TPS4 | **Tricyclene synthase TPS4, chloroplastic** | 112.5 | 1.492 | 10.0 | 15.0 | 43.3 | 5.7 | 0.10 |
| 625 | P51082 | CHSB | **Chalcone synthase 1B** | 24.0 | 1.492 | 8.0 | 11.0 | 19.1 | 12.7 | 0.20 |
| 626 | P42353 | RRL16 | **50S ribosomal protein L16; chloroplastic (Fragment)** | 53.0 | 1.492 | 5.0 | 5.0 | 10.4 | 43.8 | 0.10 |
| 627 | P58385 | PSAB | **Photosystem I P700 chlorophyll a apoprotein A2** | 31.8 | 1.507 | 11.0 | 5.0 | 10.0 | 10.7 | 0.30 |
| 628 | P26413 | HSP70 | **Heat shock 70 kDa protein** | 67.3 | 1.507 | 16.0 | 13.0 | 19.0 | 11.5 | 0.50 |
| 629 | P0DH60 | M3OM2 | **(+)-6a-hydroxymaackiain 3-O-methyltransferase 2** | 61.7 | 1.522 | 12.0 | 5.0 | 8.4 | 23.2 | 0.10 |
| 630 | P15102 | GLNA4 | **Glutamine synthetase leaf isozyme, chloroplastic** | 16.4 | 1.522 | 7.0 | 4.0 | 16.1 | 15.3 | 0.30 |
| 631 | B5LMN4 | ACCD | **Acetyl-coenzyme A carboxylase carboxyl transferase subunit beta,chloroplastic** | 227.5 | 1.522 | 7.0 | 7.0 | 7.0 | 21.0 | 0.10 |
| 632 | Q9BBT4 | YCF3 | **Photosystem I assembly protein Ycf3** | 4.4 | 1.537 | 4.0 | 4.0 | 17.5 | 17.2 | 0.40 |
| 633 | O49859 | C82A4 | **Cytochrome P450 82A4** | 101.4 | 1.537 | 4.0 | 8.0 | 5.8 | 12.2 | 0.40 |
| 634 | P48631 | FD6E2 | **Omega-6 fatty acid desaturase,  endoplasmic reticulum isozyme 2** | 39.7 | 1.553 | 8.0 | 5.0 | 45.0 | 143.0 | 0.30 |
| 635 | Q6EJ97 | ISPS | **Isoprene synthase, chloroplastic** | 58.4 | 1.568 | 13.0 | 13.0 | 37.6 | 41.0 | 0.40 |
| 636 | Q2PMQ0 | RR8 | **30S ribosomal protein S8, chloroplastic** | 210.7 | 1.568 | 9.0 | 8.0 | 64.7 | 30.8 | 0.50 |
| 637 | Q9BBQ4 | RPOA | **DNA-directed RNA polymerase subunit alpha** | 173.8 | 1.568 | 12.0 | 10.0 | 17.1 | 12.7 | 0.40 |
| 638 | Q9BBP9 | RK16 | **50S ribosomal protein L16, chloroplastic** | 119.0 | 1.568 | 5.0 | 6.0 | 26.1 | 37.3 | 0.40 |
| 639 | P20178 | THS1 | **Stilbene synthase 1** | 0.0 | 1.584 | 7.0 | 10.0 | 7.6 | 20.4 | 0.50 |
| 640 | Q2PMN8 | NDHH | **NAD(P)H-quinone oxidoreductase subunit H, chloroplastic** | 15.9 | 1.584 | 14.0 | 12.0 | 43.4 | 29.0 | 0.20 |
| 641 | Q04708 | P5CR | **Pyrroline-5-carboxylate reductase** | 251.3 | 1.584 | 5.0 | 7.0 | 16.4 | 11.5 | 0.40 |
| 642 | P93472 | DIM | **Delta(24)-sterol reductase** | 75.0 | 1.584 | 9.0 | 8.0 | 21.7 | 21.0 | 0.10 |
| 643 | Q2HVD6 | MTA70 | **Putative N6-adenosine-methyltransferase MT-A70-like** | 90.1 | 1.600 | 14.0 | 14.0 | 9.5 | 25.2 | 0.20 |
| 644 | P08241 | RR2 | **30S ribosomal protein S2, chloroplastic** | 85.7 | 1.600 | 11.0 | 13.0 | 35.1 | 7.8 | 0.10 |
| 645 | Q764T8 | LUPS | **Lupeol synthase** | 31.2 | 1.616 | 6.0 | 8.0 | 21.6 | 28.6 | 0.50 |
| 646 | Q6RVV4 | TIC32 | **Short-chain dehydrogenase TIC 32, chloroplastic** | 153.9 | 1.616 | 12.0 | 6.0 | 31.8 | 16.0 | 0.10 |
| 647 | P40620 | HMGL | **HMG1/2-like protein** | 137.4 | 1.632 | 9.0 | 4.0 | 9.7 | 24.6 | 0.20 |
| 648 | Q42920 | PME | **Pectinesterase/pectinesterase inhibitor** | 45.6 | 1.649 | 18.0 | 12.0 | 9.8 | 16.0 | 0.30 |
| 649 | P31239 | ACCO | **1-aminocyclopropane-1-carboxylate oxidase** | 178.9 | 1.649 | 6.0 | 7.0 | 17.9 | 13.1 | 0.50 |
| 650 | A4GG85 | RRL16 | **50S ribosomal protein L16; chloroplastic** | 31.5 | 1.649 | 5.0 | 6.0 | 33.7 | 21.2 | 0.30 |
| 651 | Q42823 | RBS | **Ribulose bisphosphate carboxylase small chain, chloroplastic** | 99.4 | 1.649 | 5.0 | 7.0 | 14.5 | 45.5 | 0.20 |
| 652 | O81972 | C82A2 | **Cytochrome P450 82A2** | 43.0 | 1.649 | 10.0 | 11.0 | 4.7 | 16.3 | 0.20 |
| 653 | Q01289 | POR | **Protochlorophyllide reductase, chloroplastic** | 106.7 | 1.665 | 12.0 | 14.0 | 23.1 | 25.5 | 0.50 |
| 654 | O24305 | M3OM1 | **(+)-6a-hydroxymaackiain 3-O-methyltransferase 1** | 74.7 | 1.665 | 5.0 | 5.0 | 25.9 | 53.9 | 0.10 |
| 655 | P51069 | THS3 | **Stilbene synthase 3** | 0.0 | 1.665 | 6.0 | 8.0 | 29.3 | 104.6 | 0.20 |
| 656 | Q41059 | GLGB2 | **1,4-alpha-glucan-branching enzyme 1, chloroplastic/ amyloplastic (Fragment)** | 150.8 | 1.665 | 14.0 | 16.0 | 16.3 | 3.1 | 0.10 |
| 657 | P02580 | ACT3 | **Actin-3** | 64.7 | 1.699 | 7.0 | 5.0 | 8.3 | 42.8 | 0.30 |
| 658 | P25012 | CCNB2 | **G2/mitotic-specific cyclin S13-7 (Fragment)** | 19.8 | 1.699 | 3.0 | 7.0 | 30.9 | 8.5 | 0.40 |
| 659 | O48928 | C77A3 | **Cytochrome P450 77A3** | 49.5 | 1.699 | 13.0 | 13.0 | 14.8 | 11.1 | 0.50 |
| 660 | P51080 | CHS | **Chalcone synthase (Fragment)** | 43.8 | 1.716 | 3.0 | 3.0 | 40.5 | 21.0 | 0.30 |
| 661 | Q43822 | PLSB | **Glycerol-3-phosphate acyltransferase, chloroplastic** | 78.7 | 1.716 | 6.0 | 11.0 | 26.8 | 47.2 | 0.10 |
| 662 | O48902 | MDHP | **Malate dehydrogenase [NADP], chloroplastic** | 144.0 | 1.716 | 14.0 | 10.0 | 27.2 | 61.2 | 0.10 |
| 663 | Q4VY51 | SYM8 | **Probable ion channel SYM8** | 26.3 | 1.733 | 19.0 | 16.0 | 22.2 | 4.7 | 0.50 |
| 664 | Q84XA3 | IMDH | **Inosine-5'-monophosphate dehydrogenase** | 53.8 | 1.733 | 9.0 | 5.0 | 79.3 | 53.8 | 0.50 |
| 665 | P12858 | G3PA | **Glyceraldehyde-3-phosphate dehydrogenase A, chloroplastic** | 190.9 | 1.733 | 14.0 | 16.0 | 8.2 | 46.5 | 0.30 |
| 666 | P15792 | KPK1 | **Protein kinase PVPK-1** | 34.9 | 1.751 | 6.0 | 14.0 | 31.1 | 22.9 | 0.10 |
| 667 | O03376 | AOX3 | **Alternative oxidase 3, mitochondrial** | 12.4 | 1.768 | 11.0 | 8.0 | 35.5 | 24.6 | 0.40 |
| 668 | O65729 | RL18 | **60S ribosomal protein L18 (Fragment)** | 90.5 | 1.768 | 10.0 | 5.0 | 26.1 | 20.7 | 0.50 |
| 669 | P93328 | NO16 | **Early nodulin-16** | 36.9 | 1.804 | 3.0 | 5.0 | 36.1 | 15.1 | 0.50 |
| 670 | P30077 | CHS9 | **Chalcone synthase 9** | 34.8 | 1.804 | 4.0 | 11.0 | 35.6 | 1.5 | 0.20 |
| 671 | Q9SC88 | GCP4 | **Gamma-tubulin complex component 4 homolog** | 89.1 | 1.822 | 13.0 | 23.0 | 25.5 | 35.8 | 0.20 |
| 672 | Q29U70 | I4OMT | **Isoflavone 4'-O-methyltransferase** | 80.4 | 1.840 | 9.0 | 14.0 | 28.0 | 34.2 | 0.30 |
| 673 | P07219 | PHSA | **Phaseolin, alpha-type** | 30.2 | 1.859 | 6.0 | 9.0 | 3.6 | 27.5 | 0.20 |
| 674 | P51081 | CHSA | **Chalcone synthase 1A** | 6.1 | 1.878 | 11.0 | 5.0 | 30.6 | 11.7 | 0.10 |
| 675 | Q43070 | GALE1 | **UDP-glucose 4-epimerase** | 27.9 | 1.896 | 8.0 | 8.0 | 7.6 | 10.2 | 0.40 |
| 676 | P51078 | CHS5 | **Chalcone synthase 4-2** | 34.8 | 1.896 | 8.0 | 7.0 | 30.1 | 19.7 | 0.40 |
| 677 | P51086 | CHS4 | **Chalcone synthase 4 (Fragment)** | 34.8 | 1.916 | 14.0 | 10.0 | 9.6 | 64.5 | 0.50 |
| 678 | P30075 | CHS4 | **Chalcone synthase 4** | 34.8 | 1.916 | 7.0 | 7.0 | 16.9 | 25.5 | 0.10 |
| 679 | P30076 | CHS8 | **Chalcone synthase 8** | 34.8 | 1.935 | 10.0 | 12.0 | 21.9 | 46.0 | 0.50 |
| 680 | B0M3E8 | UGE1 | **Bifunctional UDP-glucose 4-epimerase and  UDP-xylose 4-epimerase 1** | 27.9 | 1.935 | 10.0 | 15.0 | 34.3 | 5.1 | 0.40 |
| 681 | O48559 | UNI | **Protein UNIFOLIATA** | 43.1 | 1.974 | 8.0 | 3.0 | 37.4 | 17.6 | 0.40 |
| 682 | P51085 | CHS3 | **Chalcone synthase 3** | 34.8 | 1.974 | 10.0 | 14.0 | 23.5 | 21.5 | 0.30 |
| 683 | O49858 | C82A3 | **Cytochrome P450 82A3** | 33.2 | 1.974 | 12.0 | 9.0 | 12.4 | 20.8 | 0.10 |
| 684 | P19168 | CHS3 | **Chalcone synthase 3** | 18.9 | 1.994 | 5.0 | 5.0 | 22.1 | 42.9 | 0.30 |
| **Up-regulated proteins** | | | | | | | | | | |
| 685 | P36875 | 2AAA | **Protein phosphatase PP2A regulatory subunit A (Fragment)** | 56.1 | 2.014 | 8.0 | 5.0 | 152.0 | 433.6 | 0.04 |
| 686 | Q9BBN8 | NDHH | **NAD(P)H-quinone oxidoreductase subunit H, chloroplastic** | 15.9 | 2.054 | 12.0 | 10.0 | 25.7 | 332.9 | 0.05 |
| 687 | P30074 | CHS2 | **Chalcone synthase 2** | 34.8 | 2.054 | 13.0 | 11.0 | 60.4 | 222.6 | 0.01 |
| 688 | Q01915 | ATPAM | **ATP synthase subunit alpha, mitochondrial** | 101.9 | 2.054 | 19.0 | 13.0 | 22.1 | 575.2 | 0.05 |
| 689 | O23883 | CHS3 | **Chalcone synthase 3** | 34.8 | 2.096 | 5.0 | 5.0 | 345.9 | 192.9 | 0.01 |
| 690 | P52575 | IFR | **Isoflavone reductase** | 66.2 | 2.096 | 8.0 | 10.0 | 37.0 | 151.1 | 0.05 |
| 691 | O22307 | C71DB | **Cytochrome P450 71D11 (Fragment)** | 34.2 | 2.096 | 8.0 | 14.0 | 65.3 | 39.7 | 0.03 |
| 692 | P02856 | VCL1 | **Vicilin, 14 kDa component** | 39.5 | 2.117 | 5.0 | 4.0 | 12.7 | 25.0 | 0.03 |
| 693 | P49046 | LEGU | **Legumain** | 86.0 | 2.138 | 11.0 | 7.0 | 41.2 | 74.5 | 0.02 |
| 694 | P53393 | SUT3 | **Low affinity sulfate transporter 3** | 66.9 | 2.160 | 8.0 | 6.0 | 8.1 | 11.9 | 0.01 |
| 695 | O82709 | CALX | **Calnexin homolog** | 157.8 | 2.160 | 18.0 | 22.0 | 40.9 | 29.8 | 0.04 |
| 696 | P51087 | CHS5 | **Chalcone synthase 5** | 34.8 | 2.203 | 14.0 | 7.0 | 39.9 | 35.9 | 0.05 |
| 697 | P51083 | CHS1 | **Chalcone synthase 1** | 34.8 | 2.203 | 7.0 | 4.0 | 27.0 | 7.0 | 0.01 |
| 698 | P51079 | CHS6-4 | **Chalcone synthase 6-4** | 24.9 | 2.203 | 6.0 | 6.0 | 19.1 | 6.8 | 0.04 |
| 699 | P51088 | CHS6 | **Chalcone synthase 6** | 34.8 | 2.226 | 4.0 | 11.0 | 42.4 | 34.9 | 0.01 |
| 700 | Q01287 | CHS2 | **Chalcone synthase 2** | 34.8 | 2.248 | 7.0 | 7.0 | 30.7 | 104.3 | 0.05 |
| 701 | Q43785 | GLNA3 | **Glutamine synthetase nodule isozyme** | 5.8 | 2.248 | 9.0 | 7.0 | 28.7 | 73.8 | 0.01 |
| 702 | O24326 | VPE2 | **Vacuolar-processing enzyme** | 41.3 | 2.270 | 10.0 | 10.0 | 37.0 | 9.1 | 0.03 |
| 703 | P30081 | CHS7 | **Chalcone synthase 7** | 24.9 | 2.293 | 8.0 | 8.0 | 21.7 | 9.8 | 0.03 |
| 704 | A4GGB2 | ATPA | **ATP synthase subunit alpha, chloroplastic** | 33.6 | 2.340 | 10.0 | 6.0 | 32.5 | 32.8 | 0.03 |
| 705 | O22586 | CHSY | **Chalcone synthase** | 34.8 | 2.363 | 12.0 | 12.0 | 29.5 | 60.5 | 0.05 |
| 706 | P20780 | ARA1 | **Arachin 21 kDa protein** | 161.3 | 2.509 | 3.0 | 4.0 | 36.1 | 36.6 | 0.02 |
| 707 | P21528 | MDHP | **Malate dehydrogenase [NADP], chloroplastic** | 54.1 | 2.560 | 11.0 | 9.0 | 56.3 | 6.0 | 0.01 |
| 708 | P46259 | TBA1 | **Tubulin alpha-1 chain** | 142.3 | 2.612 | 4.0 | 8.0 | 27.2 | 21.5 | 0.03 |
| 709 | P28583 | TBA1 | **Calcium-dependent protein kinase SK5** | 87.1 | 2.691 | 9.0 | 8.0 | 36.3 | 37.4 | 0.01 |
| 710 | O48561 | CATA4 | **Catalase-4** | 48.6 | 2.746 | 19.0 | 9.0 | 15.0 | 135.5 | 0.05 |
| 711 | Q43068 | C82A1 | **Cytochrome P450 82A1 (Fragment)** | 20.8 | 2.858 | 13.0 | 11.0 | 70.2 | 66.3 | 0.03 |
| 712 | Q43093 | SSG2 | **Granule-bound starch synthase 2, chloroplastic/amyloplastic** | 26.2 | 3.004 | 15.0 | 23.0 | 48.1 | 60.3 | 0.04 |
| 713 | A4PU48 | METK | **S-adenosylmethionine synthase** | 19.2 | 3.096 | 4.0 | 6.0 | 9.2 | 15.0 | 0.04 |
| 714 | P49680 | IAA6 | **Auxin-induced protein IAA6** | 28.1 | 3.190 | 5.0 | 5.0 | 20.2 | 16.3 | 0.03 |
| 715 | Q9M5Q1 | FUT1 | **Galactoside 2-alpha-L-fucosyltransferase** | 22.8 | 3.254 | 11.0 | 10.0 | 71.6 | 15.3 | 0.01 |
| 716 | P93484 | VSR1 | **Vacuolar-sorting receptor 1** | 40.4 | 3.254 | 12.0 | 8.0 | 51.9 | 56.7 | 0.05 |
| 717 | Q2PMU9 | ATPE | **ATP synthase epsilon chain, chloroplastic** | 78.5 | 3.287 | 5.0 | 3.0 | 8.3 | 9.5 | 0.04 |
| 718 | O81117 | C94A1 | **Cytochrome P450 94A1(Fatty acid biosynthesis,  Plamitate, laurate C10-C18)** | 51.5 | 3.387 | 13.0 | 6.0 | 19.3 | 60.6 | 0.05 |
| 719 | O04300 | RGP1 | **Probable UDP-arabinopyranose mutase 1** | 32.5 | 3.456 | 3.0 | 5.0 | 61.6 | 14.0 | 0.05 |
| 720 | P00965 | GLNA3 | **Glutamine synthetase N-1** | 7.4 | 3.669 | 7.0 | 7.0 | 12.7 | 114.7 | 0.05 |
| 721 | P28590 | ABRC | **Abrin-c OS** | 160.2 | 3.743 | 6.0 | 12.0 | 18.0 | 35.2 | 0.04 |
| 722 | P25700 | IT2 | **Trypsin inhibitor 2** | 237.8 | 4.179 | 5.0 | 4.0 | 12.1 | 52.3 | 0.02 |
| 723 | P51851 | PDC2 | **Pyruvate decarboxylase 2 (Fragment)** | 189.4 | 4.572 | 13.0 | 10.0 | 10.2 | 48.6 | 0.01 |
| 724 | P13917 | 7SB1 | **Basic 7S globulin** | 117.1 | 5.104 | 9.0 | 7.0 | 25.4 | 31.0 | 0.02 |
| 725 | Q06009 | PP2A | **Serine/threonine-protein phosphatase PP2A catalytic subunit** | 32.0 | 6.554 | 3.0 | 5.0 | 5.9 | 166.6 | 0.03 |
| 726 | O82134 | PCNA | **Proliferating cell nuclear antigen** | 129.0 | 7.171 | 9.0 | 8.0 | 17.6 | 59.6 | 0.02 |
| 727 | P81007 | ENT | **Cytolytic protein enterolobin** | 199.0 | 7.316 | 6.0 | 6.0 | 11.3 | 64.8 | 0.03 |
| 728 | O65743 | RL24 | **60S ribosomal protein L24** | 28.8 | 7.925 | 6.0 | 6.0 | 9.6 | 28.5 | 0.01 |
| 729 | Q06930 | ABR18 | **ABA-responsive protein ABR18** | 23.6 | 8.415 | 4.0 | 4.0 | 5.5 | 62.2 | 0.03 |
| 730 | B5BSX1 | BAMO | **Beta-amyrin 11-oxidase** | 17.9 | 8.846 | 10.0 | 5.0 | 9.0 | 73.6 | 0.01 |
| 731 | P45456 | ACEA1 | **Isocitrate lyase 1 (Fragment)** | 53.2 | 12.554 | 9.0 | 16.0 | 35.8 | 20.3 | 0.05 |
| 732 | P05190 | LEGB4 | **Legumin type B** | 14.7 | 13.197 | 5.0 | 6.0 | 17.6 | 25.0 | 0.01 |
| 733 | P01070 | ITRA | **Trypsin inhibitor A** | 347.1 | 17.993 | 7.0 | 6.0 | 17.6 | 24.9 | 0.02 |
| 734 | P05693 | LEGK | **Legumin K (Fragment)** | 79.0 | 19.886 | 9.0 | 13.0 | 13.3 | 13.2 | 0.04 |
| 735 | Q6PSC6 | MATK | **Maturase K** | 15.1 | 23.807 | 12.0 | 10.0 | 18.8 | 9.3 | 0.04 |
| 736 | O48923 | C71DA | **Cytochrome P450 71D10** | 35.9 | 26.311 | 8.0 | 6.0 | 37.5 | 9.7 | 0.01 |
| 737 | Q9TKS4 | MATK | **Maturase K** | 17.1 | 27.660 | 13.0 | 12.0 | 26.6 | 22.9 | 0.02 |
| 738 | Q8LSN3 | FYPP | **Phytochrome-associated serine/threonine-protein phosphatase** | 192.3 | 31.500 | 8.0 | 8.0 | 95.0 | 23.7 | 0.05 |
| 739 | P16080 | LEGB7 | **Legumin type B (Fragment)** | 14.7 | 41.679 | 9.0 | 4.0 | 22.7 | 33.5 | 0.03 |
| 740 | P16078 | LEGB2 | **Legumin type B (Fragment)** | 14.7 | 42.948 | 6.0 | 17.0 | 263.9 | 285.5 | 0.01 |
| 741 | P05692 | LEGJ | **Legumin J** | 52.9 | 43.380 | 12.0 | 13.0 | 24.4 | 21.5 | 0.01 |
| 742 | P16079 | LEGB6 | **Legumin type B (Fragment)** | 32.1 | 46.525 | 3.0 | 6.0 | 13.4 | 48.8 | 0.05 |
| **Control specific proteins** | | | | | | | | | | |
| 743 | Q53B75 | CF1B1 | **Chalcone-flavonone isomerase 1B-1** | 233.8 | Control_C5 | 6.0 | 0.0 | 733.3 | NA | NA |
| 744 | P51820 | DRTS | **Bifunctional dihydrofolate reductase-thymidylate synthase** | 57.0 | Control_C5 | 9.0 | 0.0 | 426.9 | NA | NA |
| 745 | P83311 | IBB | **Bowman-Birk type proteinase inhibitor** | 103.9 | Control_C5 | 2.0 | 0.0 | 599.3 | NA | NA |
| 746 | Q03467 | E13B | **Glucan endo-1,3-beta-glucosidase** | 96.4 | Control_C5 | 9.0 | 0.0 | 207.7 | NA | NA |
| 747 | P52904 | ODPB | **Pyruvate dehydrogenase E1 component subunit beta,  mitochondrial** | 70.6 | Control_C5 | 7.0 | 0.0 | 330.5 | NA | NA |
| 748 | P52902 | ODPA | **Pyruvate dehydrogenase E1 component subunit alpha,  mitochondrial** | 62.6 | Control_C5 | 15.0 | 0.0 | 225.1 | NA | NA |
| 749 | P83304 | LEC | **Mannose/glucose-specific lectin (Fragment)** | 4.1 | Control_C5 | 9.0 | 0.0 | 239.6 | NA | NA |
| 750 | O49046 | ARGI | **Arginase** | 48.7 | Control_C5 | 12.0 | 0.0 | 414.5 | NA | NA |
| 751 | P46298 | RS13 | **40S ribosomal protein S13** | 66.7 | Control_C5 | 3.0 | 0.0 | 93.9 | NA | NA |
| 752 | Q6WNQ8 | C81E8 | **Cytochrome P450 81E8** | 88.5 | Control_C5 | 11.0 | 0.0 | 184.6 | NA | NA |
| 753 | Q8GTE3 | RS3A | **40S ribosomal protein S3a** | 58.2 | Control_C5 | 9.0 | 0.0 | 737.5 | NA | NA |
| 754 | P31656 | CADH | **Probable cinnamyl alcohol dehydrogenase** | 22.2 | Control_C5 | 6.0 | 0.0 | 560.0 | NA | NA |
| 755 | P46275 | F16P1 | **Fructose-1,6-bisphosphatase, chloroplastic** | 112.0 | Control_C5 | 5.0 | 0.0 | 492.4 | NA | NA |
| 756 | P32733 | ID5A | **Kunitz-type trypsin inhibitor alpha chain** | 80.5 | Control_C5 | 2.0 | 0.0 | 447.6 | NA | NA |
| 757 | P46256 | ALF1 | **Fructose-bisphosphate aldolase, cytoplasmic isozyme 1** | 70.8 | Control_C5 | 3.0 | 0.0 | 919.4 | NA | NA |
| 758 | P14749 | AGAL | **Alpha-galactosidase** | 62.0 | Control_C5 | 3.0 | 0.0 | 973.2 | NA | NA |
| 759 | P04670 | URIC1 | **Uricase-2 isozyme 1** | 35.7 | Control_C5 | 5.0 | 0.0 | 302.6 | NA | NA |
| 760 | P81406 | GAPN | **NADP-dependent glyceraldehyde-3-phosphate dehydrogenase** | 114.4 | Control_C5 | 13.0 | 0.0 | 230.7 | NA | NA |
| 761 | Q2PMU5 | RR4 | **30S ribosomal protein S4, chloroplastic** | 111.8 | Control_C5 | 3.0 | 0.0 | 94.2 | NA | NA |
| 762 | P53385 | HUTU | **Urocanate hydratase** | 45.3 | Control_C5 | 7.0 | 0.0 | 868.4 | NA | NA |
| 763 | P22973 | LEC2 | **Anti-H(O) lectin 2** | 82.7 | Control_C5 | 2.0 | 0.0 | 808.9 | NA | NA |
| 764 | Q564G7 | GMGT1 | **Galactomannan galactosyltransferase 1** | 60.2 | Control_C5 | 10.0 | 0.0 | 314.5 | NA | NA |
| 765 | P38661 | PDIA6 | **Probable protein disulfide-isomerase A6** | 102.1 | Control_C5 | 9.0 | 0.0 | 421.8 | NA | NA |
| 766 | Q2PMR0 | CLPP | **ATP-dependent Clp protease proteolytic subunit** | 1.6 | Control_C5 | 7.0 | 0.0 | 564.6 | NA | NA |
| 767 | P13603 | ADH1 | **Alcohol dehydrogenase 1** | 4.5 | Control_C5 | 5.0 | 0.0 | 271.9 | NA | NA |
| 768 | P81726 | ICI2 | **Subtilisin inhibitor CLSI-II** | 46.0 | Control_C5 | 2.0 | 0.0 | 545.2 | NA | NA |
| 769 | Q2PMQ3 | RPOA | **DNA-directed RNA polymerase subunit alpha** | 224.3 | Control_C5 | 12.0 | 0.0 | 401.1 | NA | NA |
| 770 | Q01516 | ALFC1 | **Fructose-bisphosphate aldolase 1, chloroplastic (Fragment)** | 38.3 | Control_C5 | 7.0 | 0.0 | 264.9 | NA | NA |
| 771 | C6T0L2 | SLE3 | **Protein SLE3** | 18.2 | Control_C5 | 2.0 | 0.0 | 196.5 | NA | NA |
| 772 | P19594 | 2SS | **2S albumin** | 15.5 | Control_C5 | 8.0 | 0.0 | 409.0 | NA | NA |
| 773 | Q9ZST8 | TIC20 | **Protein TIC 20, chloroplastic** | 118.7 | Control_C5 | 13.0 | 0.0 | 242.7 | NA | NA |
| 774 | P41089 | CFI | **Chalcone-flavonone isomerase** | 119.0 | Control_C5 | 6.0 | 0.0 | 511.1 | NA | NA |
| 775 | P46519 | LEA14 | **Desiccation protectant protein Lea14 homolog** | 47.5 | Control_C5 | 5.0 | 0.0 | 988.1 | NA | NA |
| 776 | P85172 | IBB1 | **Bowman-Birk type proteinase inhibitor** | 69.2 | Control_C5 | 2.0 | 0.0 | 62.4 | NA | NA |
| 777 | P55844 | RL14 | **Probable 60S ribosomal protein L14** | 31.3 | Control_C5 | 3.0 | 0.0 | 870.6 | NA | NA |
| 778 | O65751 | RSSA | **40S ribosomal protein SA** | 53.6 | Control_C5 | 9.0 | 0.0 | 617.3 | NA | NA |
| 779 | Q9XHC6 | C93E1 | **Beta-amyrin 24-hydroxylase** | 88.8 | Control_C5 | 8.0 | 0.0 | 456.7 | NA | NA |
| 780 | Q41219 | LEGRE | **Leghemoglobin reductase** | 31.0 | Control_C5 | 14.0 | 0.0 | 253.9 | NA | NA |
| 781 | Q5NE24 | NSP2 | **Nodulation-signaling pathway 2 protein** | 32.5 | Control_C5 | 4.0 | 0.0 | 919.3 | NA | NA |
| 782 | C6SXZ3 | CSPL8 | **CASP-like protein 1D1** | 23.4 | Control_C5 | 3.0 | 0.0 | 438.0 | NA | NA |
| 783 | O65731 | RS5 | **40S ribosomal protein S5 (Fragment)** | 47.7 | Control_C5 | 5.0 | 0.0 | 359.4 | NA | NA |
| 784 | P35694 | XTH2 | **Xyloglucan endotransglucosylase/hydrolase 2** | 14.0 | Control_C5 | 4.0 | 0.0 | 676.2 | NA | NA |
| 785 | Q8LNZ5 | XTHB | **Probable xyloglucan endotransglucosylase/hydrolase protein B** | 8.9 | Control_C5 | 5.0 | 0.0 | 842.4 | NA | NA |
| 786 | Q07463 | PUR7 | **Phosphoribosylaminoimidazole-succinocarboxamide synthase,  chloroplastic (Fragment)** | 58.7 | Control_C5 | 1.0 | 0.0 | 559.2 | NA | NA |
| 787 | Q01807 | LEC2 | **Truncated lectin 2** | 69.9 | Control_C5 | 4.0 | 0.0 | 192.0 | NA | NA |
| 788 | P47905 | RS27A | **Ubiquitin-40S ribosomal protein S27a** | 163.7 | Control_C5 | 8.0 | 0.0 | 241.7 | NA | NA |
| 789 | P50346 | RLA0 | **60S acidic ribosomal protein P0** | 134.6 | Control_C5 | 5.0 | 0.0 | 825.0 | NA | NA |
| 790 | P05088 | PHAE | **Erythroagglutinating phytohemagglutinin** | 138.5 | Control_C5 | 3.0 | 0.0 | 709.6 | NA | NA |
| 791 | P05087 | PHAL | **Leucoagglutinating phytohemagglutinin** | 378.3 | Control_C5 | 4.0 | 0.0 | 183.5 | NA | NA |
| 792 | Q9SM57 | OEP21 | **Outer envelope pore protein 21, chloroplastic** | 48.3 | Control_C5 | 6.0 | 0.0 | 692.3 | NA | NA |
| 793 | P93332 | NOD3 | **Bidirectional sugar transporter N3** | 61.8 | Control_C5 | 4.0 | 0.0 | 310.1 | NA | NA |
| 794 | A0AAR7 | CCAMK | **Calcium and calcium/calmodulin-dependent  serine/threonine-protein kinase** | 107.1 | Control_C5 | 16.0 | 0.0 | 498.2 | NA | NA |
| 795 | P17673 | RBS | **Ribulose bisphosphate carboxylase small chain, chloroplastic** | 38.1 | Control_C5 | 3.0 | 0.0 | 252.0 | NA | NA |
| 796 | P49163 | RK22 | **50S ribosomal protein L22, chloroplastic** | 16.7 | Control_C5 | 2.0 | 0.0 | 649.4 | NA | NA |
| 797 | Q8MC99 | RPOA | **DNA-directed RNA polymerase subunit alpha** | 110.3 | Control_C5 | 10.0 | 0.0 | 350.7 | NA | NA |
| 798 | P26987 | SAM22 | **Stress-induced protein SAM22** | 129.0 | Control_C5 | 4.0 | 0.0 | 282.4 | NA | NA |
| 799 | Q9BBU3 | PSBA | **Photosystem II protein D1** | 26.7 | Control_C5 | 3.0 | 0.0 | 59.0 | NA | NA |
| 800 | P40590 | RL34 | **60S ribosomal protein L34** | 58.5 | Control_C5 | 6.0 | 0.0 | 235.1 | NA | NA |
| 801 | O24296 | GPX1 | **Phospholipid hydroperoxide glutathione peroxidase, chloroplastic** | 97.4 | Control_C5 | 6.0 | 0.0 | 333.9 | NA | NA |
| 802 | P05046 | LEC | **Lectin** | 47.1 | Control_C5 | 3.0 | 0.0 | 524.6 | NA | NA |
| 803 | P05045 | LEC1 | **Seed lectin subunit I** | 73.6 | Control_C5 | 2.0 | 0.0 | 711.8 | NA | NA |
| 804 | Q9BBR1 | RR18 | **30S ribosomal protein S18, chloroplastic** | 71.8 | Control_C5 | 4.0 | 0.0 | 308.2 | NA | NA |
| 805 | Q9XH46 | TATA | **Sec-independent protein translocase protein TATA, chloroplastic** | 316.3 | Control_C5 | 4.0 | 0.0 | 821.7 | NA | NA |
| 806 | Q6PSU2 | CONG7 | **Conglutin-7** | 54.0 | Control_C5 | 3.0 | 0.0 | 83.3 | NA | NA |
| 807 | Q9BBQ1 | RR8 | **30S ribosomal protein S8, chloroplastic** | 44.7 | Control_C5 | 4.0 | 0.0 | 878.6 | NA | NA |
| 808 | Q9SP37 | SAHH | **Adenosylhomocysteinase** | 98.5 | Control_C5 | 15.0 | 0.0 | 259.1 | NA | NA |
| 809 | P81371 | LECS | **Seed lectin** | 107.0 | Control_C5 | 5.0 | 0.0 | 286.9 | NA | NA |
| 810 | P13240 | DR206 | **Disease resistance response protein 206** | 25.9 | Control_C5 | 2.0 | 0.0 | 280.2 | NA | NA |
| 811 | Q9S9E3 | IBB | **Horsegram inhibitor 1** | 140.8 | Control_C5 | 3.0 | 0.0 | 860.8 | NA | NA |
| 812 | Q39527 | LECR | **Lectin-related protein (Fragment)** | 273.1 | Control_C5 | 3.0 | 0.0 | 195.0 | NA | NA |
| 813 | Q43064 | PYRB3 | **Aspartate carbamoyltransferase 3, chloroplastic** | 90.0 | Control_C5 | 11.0 | 0.0 | 360.9 | NA | NA |
| 814 | P17928 | CALM | **Calmodulin** | 248.4 | Control_C5 | 5.0 | 0.0 | 742.4 | NA | NA |
| 815 | Q3LSN4 | LIAS1 | **Lipoyl synthase 1, mitochondrial** | 37.7 | Control_C5 | 8.0 | 0.0 | 312.3 | NA | NA |
| 816 | P31893 | OAT | **Ornithine aminotransferase** | 40.2 | Control_C5 | 14.0 | 0.0 | 861.1 | NA | NA |
| 817 | P25804 | CYSP | **Cysteine proteinase 15A** | 55.3 | Control_C5 | 8.0 | 0.0 | 418.0 | NA | NA |
| 818 | Q96451 | 1433B | **14-3-3-like protein B (Fragment)** | 186.9 | Control_C5 | 5.0 | 0.0 | 127.7 | NA | NA |
| 819 | A4GGF3 | RR15 | **30S ribosomal protein S15, chloroplastic** | 365.8 | Control_C5 | 5.0 | 0.0 | 709.7 | NA | NA |
| 820 | P12459 | TBB1 | **Tubulin beta-1 chain** | 95.6 | Control_C5 | 6.0 | 0.0 | 948.8 | NA | NA |
| 821 | A4GGE3 | NU5C | **NAD(P)H-quinone oxidoreductase subunit 5, chloroplastic** | 13.0 | Control_C5 | 5.0 | 0.0 | 609.6 | NA | NA |
| 822 | Q4LDF9 | OEP37 | **Outer envelope pore protein 37, chloroplastic** | 25.9 | Control_C5 | 4.0 | 0.0 | 66.5 | NA | NA |
| 823 | P22503 | GUN | **Endoglucanase** | 137.8 | Control_C5 | 5.0 | 0.0 | 175.7 | NA | NA |
| 824 | P12786 | COX1 | **Cytochrome c oxidase subunit 1** | 115.2 | Control_C5 | 4.0 | 0.0 | 726.2 | NA | NA |
| 825 | P42088 | LEC | **Lectin OS** | 124.2 | Control_C5 | 6.0 | 0.0 | 364.5 | NA | NA |
| 826 | P36361 | CHI5 | **Endochitinase CH5B** | 87.9 | Control_C5 | 8.0 | 0.0 | 497.7 | NA | NA |
| 827 | O22308 | 7OMT6 | **Isoflavone-7-O-methyltransferase 6** | 92.3 | Control_C5 | 4.0 | 0.0 | 658.6 | NA | NA |
| 828 | P26291 | UCRIA | **Cytochrome b6-f complex iron-sulfur subunit, chloroplastic** | 31.1 | Control_C5 | 4.0 | 0.0 | 312.5 | NA | NA |
| 829 | P09059 | SVF30 | **Unknown seed protein 30.1** | 103.8 | Control_C5 | 5.0 | 0.0 | 708.9 | NA | NA |
| 830 | P42054 | VDAC | **Outer plastidial membrane protein porin** | 96.6 | Control_C5 | 6.0 | 0.0 | 759.0 | NA | NA |
| 831 | C6TBN2 | AKR1 | **Probable aldo-keto reductase 1** | 68.9 | Control_C5 | 6.0 | 0.0 | 237.0 | NA | NA |
| 832 | Q41112 | SRP | **Stress-related protein** | 66.1 | Control_C5 | 5.0 | 0.0 | 51.2 | NA | NA |
| 833 | Q9ZS21 | LGUL | **Lactoylglutathione lyase** | 57.9 | Control_C5 | 4.0 | 0.0 | 625.3 | NA | NA |
| 834 | Q2HU68 | H2A1 | **Probable histone H2A.1** | 282.1 | Control_C5 | 7.0 | 0.0 | 423.8 | NA | NA |
| 835 | P0CC62 | NU2C1 | **NAD(P)H-quinone oxidoreductase subunit 2 A, chloroplastic** | 151.7 | Control_C5 | 7.0 | 0.0 | 743.1 | NA | NA |
| 836 | Q40359 | ALFIN | **PHD finger protein Alfin1** | 68.7 | Control_C5 | 7.0 | 0.0 | 518.1 | NA | NA |
| 837 | P11964 | SODCP | **Superoxide dismutase [Cu-Zn], chloroplastic** | 62.6 | Control_C5 | 2.0 | 0.0 | 313.6 | NA | NA |
| 838 | A4GG96 | YCF3 | **Photosystem I assembly protein Ycf3** | 52.4 | Control_C5 | 3.0 | 0.0 | 428.2 | NA | NA |
| 839 | A4GG95 | RR4 | **30S ribosomal protein S4, chloroplastic** | 41.0 | Control_C5 | 3.0 | 0.0 | 424.6 | NA | NA |
| 840 | P32291 | FAD3E | **Omega-3 fatty acid desaturase, endoplasmic reticulum** | 140.8 | Control_C5 | 9.0 | 0.0 | 752.7 | NA | NA |
| 841 | P16148 | PLZ12 | **Protein PPLZ12** | 77.3 | Control_C5 | 10.0 | 0.0 | 681.8 | NA | NA |
| 842 | P52419 | PUR1 | **Amidophosphoribosyltransferase, chloroplastic (Fragment)** | 118.3 | Control_C5 | 10.0 | 0.0 | 689.6 | NA | NA |
| 843 | P26204 | BGLS | **Non-cyanogenic beta-glucosidase** | 111.7 | Control_C5 | 11.0 | 0.0 | 487.7 | NA | NA |
| 844 | P26563 | AATM | **Aspartate aminotransferase P2, mitochondrial (Fragment)** | 22.4 | Control_C5 | 6.0 | 0.0 | 664.3 | NA | NA |
| 845 | P52766 | NDHK | **NAD(P)H-quinone oxidoreductase subunit K, chloroplastic** | 19.4 | Control_C5 | 6.0 | 0.0 | 356.1 | NA | NA |
| 846 | A2Q1V6 | ATG4 | **Cysteine protease ATG4** | 37.5 | Control_C5 | 16.0 | 0.0 | 469.1 | NA | NA |
| 847 | P16121 | HSP70 | **Heat shock 70 kDa protein (Fragment)** | 98.2 | Control_C5 | 6.0 | 0.0 | 670.8 | NA | NA |
| 848 | Q8H0G2 | CFI1 | **Chalcone-flavonone isomerase 1** | 14.5 | Control_C5 | 5.0 | 0.0 | 142.8 | NA | NA |
| 849 | P31175 | NDHK | **NAD(P)H-quinone oxidoreductase subunit K, chloroplastic** | 41.9 | Control_C5 | 5.0 | 0.0 | 221.9 | NA | NA |
| 850 | P31174 | NDHJ | **NAD(P)H-quinone oxidoreductase subunit J, chloroplastic** | 113.5 | Control_C5 | 4.0 | 0.0 | 399.1 | NA | NA |
| 851 | P22196 | PER2 | **Cationic peroxidase 2** | 164.4 | Control_C5 | 6.0 | 0.0 | 172.0 | NA | NA |
| 852 | P10821 | IT1A | **Trypsin inhibitor 1A** | 38.0 | Control_C5 | 4.0 | 0.0 | 363.1 | NA | NA |
| 853 | O50044 | KDSA | **2-dehydro-3-deoxyphosphooctonate aldolase** | 113.4 | Control_C5 | 6.0 | 0.0 | 359.5 | NA | NA |
| 854 | Q9XG83 | G2OX | **Gibberellin 2-beta-dioxygenase** | 15.7 | Control_C5 | 3.0 | 0.0 | 191.0 | NA | NA |
| 855 | Q3LRV4 | NRL4B | **Bifunctional nitrilase/nitrile hydratase NIT4B** | 18.8 | Control_C5 | 6.0 | 0.0 | 591.6 | NA | NA |
| 856 | Q8LPW2 | RT13 | **Small ribosomal subunit protein S13, mitochondrial** | 47.0 | Control_C5 | 8.0 | 0.0 | 432.9 | NA | NA |
| 857 | P22177 | PCNA | **Proliferating cell nuclear antigen (Fragment)** | 196.1 | Control_C5 | 4.0 | 0.0 | 537.6 | NA | NA |
| 858 | Q9LKG7 | UGPA | **UTP-glucose-1-phosphate uridylyltransferase** | 91.9 | Control_C5 | 10.0 | 0.0 | 530.7 | NA | NA |
| 859 | Q42800 | DAPA | **4-hydroxy-tetrahydrodipicolinate synthase, chloroplastic** | 151.2 | Control_C5 | 9.0 | 0.0 | 403.2 | NA | NA |
| 860 | P23233 | NO16 | **Nodulin-16** | 93.4 | Control_C5 | 2.0 | 0.0 | 970.6 | NA | NA |
| 861 | Q41701 | NO12 | **Early nodulin-12** | 235.2 | Control_C5 | 6.0 | 0.0 | 624.1 | NA | NA |
| 862 | C0HJX1 | LECC1 | **Mannose/glucose-specific lectin** | 48.3 | Control_C5 | 6.0 | 0.0 | 418.5 | NA | NA |
| 863 | Q39445 | TBB | **Tubulin beta chain** | 179.5 | Control_C5 | 10.0 | 0.0 | 696.1 | NA | NA |
| 864 | C0HJW7 | MANA | **Alpha-mannosidase (Fragments)** | 441.9 | Control_C5 | 3.0 | 0.0 | 472.5 | NA | NA |
| 865 | P49351 | FPPS1 | **Farnesyl pyrophosphate synthase 1** | 17.0 | Control_C5 | 4.0 | 0.0 | 396.1 | NA | NA |
| 866 | C0HK81 | LECA | **Lectin** | 118.7 | Control_C5 | 5.0 | 0.0 | 474.9 | NA | NA |
| 867 | P14226 | PSBO | **Oxygen-evolving enhancer protein 1, chloroplastic** | 92.3 | Control_C5 | 12.0 | 0.0 | 97.8 | NA | NA |
| 868 | Q03227 | COX3 | **Cytochrome c oxidase subunit 3** | 201.3 | Control_C5 | 3.0 | 0.0 | 284.1 | NA | NA |
| 869 | P23558 | LEC1 | **Lectin 1** | 162.1 | Control_C5 | 5.0 | 0.0 | 484.5 | NA | NA |
| 870 | P04149 | ARA5 | **Arachin 25 kDa protein** | 79.8 | Control_C5 | 6.0 | 0.0 | 951.0 | NA | NA |
| 871 | P04144 | NO23 | **Nodulin-23** | 140.3 | Control_C5 | 4.0 | 0.0 | 706.8 | NA | NA |
| 872 | P23535 | E13B | **Glucan endo-1,3-beta-glucosidase, basic isoform** | 111.4 | Control_C5 | 5.0 | 0.0 | 391.3 | NA | NA |
| 873 | P04122 | LECB | **Lectin beta-1 and beta-2 chains** | 365.8 | Control_C5 | 6.0 | 0.0 | 197.7 | NA | NA |
| 874 | P80463 | PHS1 | **Phaseolin** | 86.6 | Control_C5 | 9.0 | 0.0 | 482.7 | NA | NA |
| 875 | P24924 | ITRY | **Trypsin inhibitor** | 189.7 | Control_C5 | 6.0 | 0.0 | 403.3 | NA | NA |
| 876 | B5LMS0 | NDHH | **NAD(P)H-quinone oxidoreductase subunit H, chloroplastic** | 48.5 | Control_C5 | 5.0 | 0.0 | 279.2 | NA | NA |
| 877 | P48513 | TF2B | **Transcription initiation factor IIB** | 112.2 | Control_C5 | 9.0 | 0.0 | 585.8 | NA | NA |
| 878 | P02240 | LGB2 | **Leghemoglobin-2** | 92.4 | Control_C5 | 4.0 | 0.0 | 963.5 | NA | NA |
| 879 | Q9SQL2 | CB24 | **Chlorophyll a-b binding protein P4, chloroplastic** | 100.2 | Control_C5 | 5.0 | 0.0 | 219.2 | NA | NA |
| 880 | P39882 | NIA | **Nitrate reductase [NADH] (Fragment)** | 19.3 | Control_C5 | 6.0 | 0.0 | 403.6 | NA | NA |
| 881 | P02238 | LGBA | **Leghemoglobin A** | 75.3 | Control_C5 | 9.0 | 0.0 | 473.0 | NA | NA |
| 882 | P02233 | LGB1 | **Leghemoglobin-1** | 110.3 | Control_C5 | 5.0 | 0.0 | 236.1 | NA | NA |
| 883 | P02232 | LGB1 | **Leghemoglobin-1** | 25.0 | Control_C5 | 4.0 | 0.0 | 753.8 | NA | NA |
| 884 | Q6DW73 | DGDG2 | **Digalactosyldiacylglycerol synthase 2, chloroplastic** | 117.4 | Control_C5 | 12.0 | 0.0 | 119.3 | NA | NA |
| 885 | Q41015 | PIP21 | **Kunitz-type trypsin inhibitor-like 1 protein** | 17.0 | Control_C5 | 4.0 | 0.0 | 299.0 | NA | NA |
| 886 | P56707 | SMTA | **Selenocysteine methyltransferase** | 28.6 | Control_C5 | 2.0 | 0.0 | 109.4 | NA | NA |
| 887 | O22518 | RSSA | **40S ribosomal protein SA** | 59.7 | Control_C5 | 5.0 | 0.0 | 236.3 | NA | NA |
| 888 | Q35639 | NU5M | **NADH-ubiquinone oxidoreductase chain 5 (Fragment)** | 81.3 | Control_C5 | 5.0 | 0.0 | 389.7 | NA | NA |
| 889 | Q42799 | C93A2 | **Cytochrome P450 93A2** | 49.4 | Control_C5 | 9.0 | 0.0 | 522.3 | NA | NA |
| 890 | Q42796 | F16P1 | **Fructose-1,6-bisphosphatase, chloroplastic** | 104.4 | Control_C5 | 8.0 | 0.0 | 666.9 | NA | NA |
| 891 | Q647H2 | AHY3 | **Arachin Ahy-3** | 79.0 | Control_C5 | 10.0 | 0.0 | 513.1 | NA | NA |
| 892 | P93162 | CHLI | **Magnesium-chelatase subunit ChlI, chloroplastic** | 43.6 | Control_C5 | 4.0 | 0.0 | 374.8 | NA | NA |
| 893 | Q84V83 | LAR | **Leucoanthocyanidin reductase** | 63.9 | Control_C5 | 9.0 | 0.0 | 518.7 | NA | NA |
| 894 | P02870 | LEC | **Lectin** | 192.3 | Control_C5 | 6.0 | 0.0 | 562.4 | NA | NA |
| 895 | P35450 | EFGC | **Elongation factor G, chloroplastic (Fragment)** | 30.5 | Control_C5 | 3.0 | 0.0 | 146.0 | NA | NA |
| 896 | Q43857 | INVA | **Acid beta-fructofuranosidase** | 104.3 | Control_C5 | 11.0 | 0.0 | 358.7 | NA | NA |
| 897 | P53763 | URIC | **Uricase-2** | 37.0 | Control_C5 | 6.0 | 0.0 | 156.4 | NA | NA |
| 898 | P28640 | DHN2 | **Dehydrin DHN2** | 181.4 | Control_C5 | 4.0 | 0.0 | 901.8 | NA | NA |
| 899 | Q9ZTR1 | SPD1 | **Spermidine synthase 1** | 46.5 | Control_C5 | 7.0 | 0.0 | 381.7 | NA | NA |
| 900 | Q9ZTR0 | SPD2 | **Spermidine synthase 2** | 58.5 | Control_C5 | 6.0 | 0.0 | 241.7 | NA | NA |
| 901 | P02866 | CONA | **Concanavalin-A** | 43.8 | Control_C5 | 3.0 | 0.0 | 648.5 | NA | NA |
| 902 | P28639 | DHN1 | **Dehydrin DHN1** | 226.7 | Control_C5 | 6.0 | 0.0 | 313.2 | NA | NA |
| 903 | P10743 | VSPB | **Stem 31 kDa glycoprotein** | 52.3 | Control_C5 | 7.0 | 0.0 | 601.1 | NA | NA |
| 904 | O81970 | C71A9 | **Cytochrome P450 71A9** | 58.7 | Control_C5 | 10.0 | 0.0 | 784.0 | NA | NA |
| 905 | Q94G17 | TATC | **Sec-independent protein translocase protein TATC, chloroplastic** | 66.6 | Control_C5 | 4.0 | 0.0 | 457.4 | NA | NA |
| 906 | P83052 | BBKI | **Kunitz-type serine protease inhibitor BbKI** | 219.6 | Control_C5 | 2.0 | 0.0 | 910.3 | NA | NA |
| 907 | P83051 | BBCI | **Kunitz-type proteinase inhibitor BbCI** | 179.4 | Control_C5 | 6.0 | 0.0 | 677.3 | NA | NA |
| 908 | Q43820 | DCAM | **S-adenosylmethionine decarboxylase proenzyme** | 65.7 | Control_C5 | 5.0 | 0.0 | 447.0 | NA | NA |
| 909 | Q41649 | FKB15 | **FK506-binding protein 2** | 115.6 | Control_C5 | 2.0 | 0.0 | 314.8 | NA | NA |
| 910 | Q41640 | RAB7 | **Ras-related protein Rab7** | 121.2 | Control_C5 | 5.0 | 0.0 | 202.3 | NA | NA |
| 911 | Q41638 | XTHA | **Xyloglucan endotransglucosylase/hydrolase protein A** | 23.0 | Control_C5 | 4.0 | 0.0 | 544.4 | NA | NA |
| 912 | P83036 | ITRY | **Trypsin inhibitor** | 158.6 | Control_C5 | 10.0 | 0.0 | 290.6 | NA | NA |
| 913 | O48920 | ARF | **ADP-ribosylation factor** | 44.8 | Control_C5 | 6.0 | 0.0 | 417.5 | NA | NA |
| 914 | P07371 | CB22 | **Chlorophyll a-b binding protein AB80, chloroplastic** | 40.7 | Control_C5 | 6.0 | 0.0 | 225.6 | NA | NA |
| 915 | O24076 | GBLP | **Guanine nucleotide-binding protein subunit beta-like protein** | 139.7 | Control_C5 | 6.0 | 0.0 | 900.2 | NA | NA |
| 916 | P86993 | LECT | **Lectin** | 81.4 | Control_C5 | 3.0 | 0.0 | 866.0 | NA | NA |
| 917 | P32110 | GSTX6 | **Probable glutathione S-transferase** | 168.0 | Control_C5 | 4.0 | 0.0 | 170.0 | NA | NA |
| 918 | P86624 | LECA | **Lectin alpha chain** | 111.2 | Control_C5 | 3.0 | 0.0 | 362.8 | NA | NA |
| 919 | P51850 | PDC1 | **Pyruvate decarboxylase 1** | 104.2 | Control_C5 | 12.0 | 0.0 | 659.5 | NA | NA |
| 920 | Q9M4T8 | PSA5 | **Proteasome subunit alpha type-5** | 13.8 | Control_C5 | 3.0 | 0.0 | 609.6 | NA | NA |
| 921 | A2SY66 | VICHY | **Vicianin hydrolase (Fragment)** | 63.9 | Control_C5 | 12.0 | 0.0 | 406.8 | NA | NA |
| 922 | A7ISP6 | CFI3 | **Chalcone-flavonone isomerase 3** | 6.8 | Control_C5 | 3.0 | 0.0 | 405.9 | NA | NA |
| 923 | P06585 | PSBA | **Photosystem II protein D1** | 19.6 | Control_C5 | 5.0 | 0.0 | 968.2 | NA | NA |
| 924 | C6SZ04 | CSPL3 | **CASP-like protein 2C1** | 40.9 | Control_C5 | 6.0 | 0.0 | 684.7 | NA | NA |
| **Treated-specific proteins** | | | | | | | | | | |
| 925 | P20150 | CEMA | **Chloroplast envelope membrane protein** | 86.1 | Treated_T3 | 0.0 | 5.0 | NA | 824.8 | NA |
| 926 | O82043 | ILV5 | **Ketol-acid reductoisomerase, chloroplastic** | 95.3 | Treated_T3 | 0.0 | 7.0 | NA | 857.0 | NA |
| 927 | Q9ZTA9 | FRIL | **Flt3 receptor-interacting lectin** | 107.0 | Treated_T3 | 0.0 | 4.0 | NA | 487.6 | NA |
| 928 | O24325 | VPE1 | **Vacuolar-processing enzyme** | 85.7 | Treated_T3 | 0.0 | 11.0 | NA | 155.1 | NA |
| 929 | P56331 | IF1A | **Eukaryotic translation initiation factor 1A** | 188.1 | Treated_T3 | 0.0 | 8.0 | NA | 233.8 | NA |
| 930 | Q2N2K1 | PHYK1 | **Probable phytol kinase 1, chloroplastic** | 131.7 | Treated_T3 | 0.0 | 5.0 | NA | 939.2 | NA |
| 931 | Q2N2K0 | PHYK3 | **Probable phytol kinase 3, chloroplastic** | 23.3 | Treated_T3 | 0.0 | 7.0 | NA | 564.0 | NA |
| 932 | P46266 | 1433 | **14-3-3-like protein** | 167.2 | Treated_T3 | 0.0 | 6.0 | NA | 217.8 | NA |
| 933 | A0A067YBQ3 | UGT13 | **UDP-glycosyltransferase 13** | 66.0 | Treated_T3 | 0.0 | 11.0 | NA | 165.4 | NA |
| 934 | P15838 | LEGA2 | **Legumin A2** | 6.2 | Treated_T3 | 0.0 | 13.0 | NA | 647.4 | NA |
| 935 | P01066 | IBB1 | **Bowman-Birk type proteinase inhibitor A-II** | 258.1 | Treated_T3 | 0.0 | 4.0 | NA | 93.2 | NA |
| 936 | P01057 | IBB3 | **Bowman-Birk type proteinase inhibitor DE-3** | 188.0 | Treated_T3 | 0.0 | 3.0 | NA | 144.7 | NA |
| 937 | Q94IC4 | FRI2 | **Ferritin-2, chloroplastic** | 25.4 | Treated_T3 | 0.0 | 3.0 | NA | 170.2 | NA |
| 938 | Q1S9I9 | H2B1 | **Probable histone H2B.1** | 33.5 | Treated_T3 | 0.0 | 8.0 | NA | 575.0 | NA |
| 939 | K7LFJ0 | PP890 | **Protein PROPEP890** | 587.9 | Treated_T3 | 0.0 | 2.0 | NA | 242.6 | NA |
| 940 | Q42372 | LCB2 | **Bark agglutinin I polypeptide B** | 61.6 | Treated_T3 | 0.0 | 6.0 | NA | 103.9 | NA |
| 941 | Q2QKL5 | PCS3 | **Glutathione gamma-glutamylcysteinyltransferase 3** | 78.7 | Treated_T3 | 0.0 | 6.0 | NA | 359.4 | NA |
| 942 | Q40193 | RB11C | **Ras-related protein Rab11C** | 225.8 | Treated_T3 | 0.0 | 4.0 | NA | 551.1 | NA |
| 943 | Q40191 | RB11A | **Ras-related protein Rab11A** | 142.2 | Treated_T3 | 0.0 | 11.0 | NA | 959.6 | NA |
| 944 | Q07176 | MMK1 | **Mitogen-activated protein kinase homolog MMK1** | 46.5 | Treated_T3 | 0.0 | 7.0 | NA | 578.0 | NA |
| 945 | P05718 | CYB | **Cytochrome b** | 85.0 | Treated_T3 | 0.0 | 4.0 | NA | 216.2 | NA |
| 946 | Q2PMQ5 | CYB6 | **Cytochrome b6** | 21.7 | Treated_T3 | 0.0 | 4.0 | NA | 889.9 | NA |
| 947 | Q06076 | ABRD | **Abrin-d** | 38.4 | Treated_T3 | 0.0 | 7.0 | NA | 595.3 | NA |
| 948 | O65781 | GALE2 | **UDP-glucose 4-epimerase GEPI48** | 99.9 | Treated_T3 | 0.0 | 6.0 | NA | 160.2 | NA |
| 949 | A0A161AT60 | NLTP1 | **Non-specific lipid-transfer protein 1** | 1034.5 | Treated_T3 | 0.0 | 6.0 | NA | 57.8 | NA |
| 950 | P10322 | NO25 | **Nodulin-25** | 140.8 | Treated_T3 | 0.0 | 8.0 | NA | 153.2 | NA |
| 951 | Q2PMN2 | NU4C | **NAD(P)H-quinone oxidoreductase chain 4, chloroplastic** | 110.5 | Treated_T3 | 0.0 | 8.0 | NA | 699.7 | NA |
| 952 | P52581 | IFRH | **Isoflavone reductase homolog** | 60.1 | Treated_T3 | 0.0 | 4.0 | NA | 863.4 | NA |
| 953 | Q2PMM9 | NU5C | **NAD(P)H-quinone oxidoreductase subunit 5, chloroplastic** | 101.3 | Treated_T3 | 0.0 | 11.0 | NA | 266.8 | NA |
| 954 | P25273 | KTI2 | **Kunitz-type trypsin inhibitor KTI2** | 190.7 | Treated_T3 | 0.0 | 6.0 | NA | 429.7 | NA |
| 955 | B1B5P4 | N8DT2 | **Naringenin 8-dimethylallyltransferase 2, chloroplastic** | 20.2 | Treated_T3 | 0.0 | 7.0 | NA | 450.1 | NA |
| 956 | P47923 | NDK2 | **Nucleoside diphosphate kinase 2, chloroplastic** | 25.7 | Treated_T3 | 0.0 | 7.0 | NA | 194.3 | NA |
| 957 | Q9FT25 | PDX1 | **Pyridoxal 5'-phosphate synthase subunit PDX1** | 18.6 | Treated_T3 | 0.0 | 8.0 | NA | 781.7 | NA |
| 958 | O49929 | OEP24 | **Outer envelope pore protein 24, chloroplastic** | 156.9 | Treated_T3 | 0.0 | 6.0 | NA | 463.5 | NA |
| 959 | P24146 | LEC4 | **Lectin-4** | 123.7 | Treated_T3 | 0.0 | 2.0 | NA | 568.5 | NA |
| 960 | P50345 | RLA0 | **60S acidic ribosomal protein P0** | 63.9 | Treated_T3 | 0.0 | 4.0 | NA | 386.4 | NA |
| 961 | P51428 | RT10 | **Ribosomal protein S10, mitochondrial** | 57.1 | Treated_T3 | 0.0 | 9.0 | NA | 382.6 | NA |
| 962 | P58155 | CEMA | **Chloroplast envelope membrane protein** | 74.3 | Treated_T3 | 0.0 | 4.0 | NA | 113.8 | NA |
| 963 | P86893 | LECS | **Seed lectin** | 16.9 | Treated_T3 | 0.0 | 1.0 | NA | 304.9 | NA |
| 964 | P62929 | ALB1D | **Albumin-1 D** | 48.4 | Treated_T3 | 0.0 | 3.0 | NA | 752.1 | NA |
| 965 | P49158 | ACCD | **Acetyl-coenzyme A carboxylase carboxyl transferase subunit beta,chloroplastic** | 111.3 | Treated_T3 | 0.0 | 5.0 | NA | 498.0 | NA |
| 966 | P00865 | RBS1 | **Ribulose bisphosphate carboxylase small chain 1, chloroplastic** | 188.6 | Treated_T3 | 0.0 | 5.0 | NA | 426.3 | NA |
| 967 | Q9BBR0 | RK20 | **50S ribosomal protein L20, chloroplastic** | 176.0 | Treated_T3 | 0.0 | 6.0 | NA | 490.4 | NA |
| 968 | Q9BBQ9 | CLPP | **ATP-dependent Clp protease proteolytic subunit** | 15.8 | Treated_T3 | 0.0 | 5.0 | NA | 446.5 | NA |
| 969 | Q43086 | PYRB1 | **Aspartate carbamoyltransferase 1, chloroplastic** | 151.8 | Treated_T3 | 0.0 | 13.0 | NA | 458.4 | NA |
| 970 | Q43075 | SPE1 | **Arginine decarboxylase** | 56.6 | Treated_T3 | 0.0 | 3.0 | NA | 928.9 | NA |
| 971 | Q43072 | HIS7 | **Imidazoleglycerol-phosphate dehydratase** | 70.2 | Treated_T3 | 0.0 | 4.0 | NA | 464.6 | NA |
| 972 | Q39528 | LEC1 | **Agglutinin-1** | 48.1 | Treated_T3 | 0.0 | 4.0 | NA | 636.9 | NA |
| 973 | H1A988 | C7254 | **11-oxo-beta-amyrin 30-oxidase** | 80.4 | Treated_T3 | 0.0 | 9.0 | NA | 451.3 | NA |
| 974 | P08927 | RUBB | **RuBisCO large subunit-binding protein subunit beta, chloroplastic** | 82.7 | Treated_T3 | 0.0 | 12.0 | NA | 215.9 | NA |
| 975 | O24542 | AX22D | **Auxin-induced protein 22D** | 158.2 | Treated_T3 | 0.0 | 5.0 | NA | 862.0 | NA |
| 976 | O24541 | AX22C | **Auxin-induced protein 22C** | 115.7 | Treated_T3 | 0.0 | 6.0 | NA | 797.2 | NA |
| 977 | P29257 | LEC2 | **2-acetamido-2-deoxy-D-galactose-binding seed lectin 2** | 41.6 | Treated_T3 | 0.0 | 2.0 | NA | 186.2 | NA |
| 978 | A4GGF2 | NDHH | **NAD(P)H-quinone oxidoreductase subunit H, chloroplastic** | 86.4 | Treated_T3 | 0.0 | 7.0 | NA | 674.4 | NA |
| 979 | A4GGE6 | NU4C | **NAD(P)H-quinone oxidoreductase chain 4, chloroplastic** | 25.4 | Treated_T3 | 0.0 | 2.0 | NA | 270.7 | NA |
| 980 | P80572 | ADHX | **Alcohol dehydrogenase class-3** | 41.5 | Treated_T3 | 0.0 | 3.0 | NA | 342.5 | NA |
| 981 | Q39818 | HS22M | **Heat shock 22 kDa protein, mitochondrial** | 127.7 | Treated_T3 | 0.0 | 8.0 | NA | 453.6 | NA |
| 982 | P39657 | RUAP | **RuBisCO-associated protein** | 62.0 | Treated_T3 | 0.0 | 4.0 | NA | 900.1 | NA |
| 983 | P48630 | FD6E1 | **Omega-6 fatty acid desaturase, endoplasmic reticulum isozyme 1** | 160.6 | Treated_T3 | 0.0 | 12.0 | NA | 207.5 | NA |
| 984 | P27047 | DRR4 | **Disease resistance response protein DRRG49-C** | 45.1 | Treated_T3 | 0.0 | 5.0 | NA | 265.7 | NA |
| 985 | A4GGB8 | CEMA | **Chloroplast envelope membrane protein** | 80.1 | Treated_T3 | 0.0 | 4.0 | NA | 149.4 | NA |
| 986 | Q41161 | LCS2 | **Seed agglutinin 2** | 61.7 | Treated_T3 | 0.0 | 5.0 | NA | 273.1 | NA |
| 987 | Q41160 | LCB3 | **Putative bark agglutinin LECRPA3 (Fragment)** | 213.1 | Treated_T3 | 0.0 | 4.0 | NA | 389.2 | NA |
| 988 | Q01417 | PM1 | **18 kDa seed maturation protein** | 72.4 | Treated_T3 | 0.0 | 10.0 | NA | 118.6 | NA |
| 989 | Q03943 | IM30 | **Membrane-associated 30 kDa protein, chloroplastic** | 264.8 | Treated_T3 | 0.0 | 9.0 | NA | 110.7 | NA |
| 990 | Q41159 | LCB1 | **Bark agglutinin I polypeptide A** | 516.7 | Treated_T3 | 0.0 | 10.0 | NA | 583.1 | NA |
| 991 | Q9FRT8 | ALB1 | **Albumin-1 (Fragment)** | 60.5 | Treated_T3 | 0.0 | 1.0 | NA | 494.9 | NA |
| 992 | Q8H0P8 | RBP1 | **RNA-binding protein 1** | 14.4 | Treated_T3 | 0.0 | 13.0 | NA | 485.6 | NA |
| 993 | Q944T2 | TCTP | **Translationally-controlled tumor protein homolog** | 159.8 | Treated_T3 | 0.0 | 7.0 | NA | 864.0 | NA |
| 994 | P0CC88 | NU2C1 | **NAD(P)H-quinone oxidoreductase subunit 2 A, chloroplastic** | 118.5 | Treated_T3 | 0.0 | 13.0 | NA | 484.9 | NA |
| 995 | Q1SU99 | H2B3 | **Probable histone H2B.3** | 33.5 | Treated_T3 | 0.0 | 6.0 | NA | 904.2 | NA |
| 996 | Q8RW99 | F16P2 | **Fructose-1,6-bisphosphatase, cytosolic** | 10.5 | Treated_T3 | 0.0 | 4.0 | NA | 306.2 | NA |
| 997 | Q9SXS3 | C93C2 | **2-hydroxyisoflavanone synthase** | 45.6 | Treated_T3 | 0.0 | 6.0 | NA | 722.8 | NA |
| 998 | P50288 | ASPG | **Isoaspartyl peptidase/L-asparaginase** | 136.5 | Treated_T3 | 0.0 | 8.0 | NA | 736.3 | NA |
| 999 | P29501 | TBB2 | **Tubulin beta-2 chain (Fragment)** | 95.0 | Treated_T3 | 0.0 | 6.0 | NA | 607.7 | NA |
| 1000 | A4GG99 | RR14 | **30S ribosomal protein S14, chloroplastic** | 100.9 | Treated_T3 | 0.0 | 5.0 | NA | 472.4 | NA |
| 1001 | C6T2J5 | CSPL4 | **CASP-like protein 2D1** | 44.2 | Treated_T3 | 0.0 | 2.0 | NA | 433.4 | NA |
| 1002 | P45621 | GSA | **Glutamate-1-semialdehyde 2,1-aminomutase, chloroplastic** | 129.7 | Treated_T3 | 0.0 | 17.0 | NA | 163.1 | NA |
| 1003 | Q40345 | IDHP | **Isocitrate dehydrogenase [NADP], chloroplastic (Fragment)** | 176.1 | Treated_T3 | 0.0 | 8.0 | NA | 769.6 | NA |
| 1004 | P52424 | PUR5 | **Phosphoribosylformylglycinamidine cyclo-ligase,  chloroplastic/mitochondrial** | 64.6 | Treated_T3 | 0.0 | 6.0 | NA | 91.5 | NA |
| 1005 | E4NKF8 | PUB1 | **U-box domain-containing protein 1** | 43.1 | Treated_T3 | 0.0 | 9.0 | NA | 813.5 | NA |
| 1006 | E3SXU4 | BHLHW | **Basic helix-loop-helix protein A** | 149.0 | Treated_T3 | 0.0 | 10.0 | NA | 130.2 | NA |
| 1007 | P49045 | VPE | **Vacuolar-processing enzyme** | 85.8 | Treated_T3 | 0.0 | 10.0 | NA | 301.9 | NA |
| 1008 | Q42822 | RBS | **Ribulose bisphosphate carboxylase small chain, chloroplastic** | 34.4 | Treated_T3 | 0.0 | 5.0 | NA | 254.3 | NA |
| 1009 | Q05462 | RL27 | **60S ribosomal protein L27** | 273.9 | Treated_T3 | 0.0 | 6.0 | NA | 687.7 | NA |
| 1010 | P25795 | AL7A1 | **Aldehyde dehydrogenase family 7 member A1** | 21.2 | Treated_T3 | 0.0 | 5.0 | NA | 582.4 | NA |
| 1011 | P68430 | H32 | **Histone H3.2** | 106.0 | Treated_T3 | 0.0 | 6.0 | NA | 371.2 | NA |
| 1012 | P42653 | 1433A | **14-3-3-like protein A** | 165.0 | Treated_T3 | 0.0 | 11.0 | NA | 318.1 | NA |
| 1013 | P68427 | H32 | **Histone H3.2** | 106.0 | Treated_T3 | 0.0 | 3.0 | NA | 229.0 | NA |
| 1014 | A0AT30 | NLTP3 | **Non-specific lipid-transfer protein 3** | 764.0 | Treated_T3 | 0.0 | 6.0 | NA | 505.8 | NA |
| 1015 | Q40977 | MDAR | **Monodehydroascorbate reductase** | 68.2 | Treated_T3 | 0.0 | 7.0 | NA | 844.5 | NA |
| 1016 | Q9FEL7 | LAX2 | **Auxin transporter-like protein 2** | 148.3 | Treated_T3 | 0.0 | 3.0 | NA | 507.4 | NA |
| 1017 | Q9FEL6 | LAX3 | **Auxin transporter-like protein 3** | 108.4 | Treated_T3 | 0.0 | 7.0 | NA | 217.7 | NA |
| 1018 | U3THC0 | FG2KI | **Inactive UDP-glycosyltransferase 79A6** | 49.1 | Treated_T3 | 0.0 | 3.0 | NA | 909.8 | NA |
| 1019 | Q9FY06 | PPF1 | **Inner membrane protein PPF-1, chloroplastic** | 67.4 | Treated_T3 | 0.0 | 4.0 | NA | 700.0 | NA |
| 1020 | C6SZP8 | CSPL5 | **CASP-like protein 1E2** | 25.7 | Treated_T3 | 0.0 | 1.0 | NA | 925.5 | NA |
| 1021 | P30364 | ASPG | **Isoaspartyl peptidase/L-asparaginase** | 159.8 | Treated_T3 | 0.0 | 6.0 | NA | 135.0 | NA |
| 1022 | P30362 | ASPG | **Isoaspartyl peptidase/L-asparaginase (Fragment)** | 30.6 | Treated_T3 | 0.0 | 3.0 | NA | 438.0 | NA |
| 1023 | K7K424 | DAT2D | **Diacylglycerol O-acyltransferase 2D** | 47.7 | Treated_T3 | 0.0 | 7.0 | NA | 631.4 | NA |
| 1024 | Q04655 | YMA6 | **Uncharacterized mitochondrial protein ORF154** | 107.3 | Treated_T3 | 0.0 | 11.0 | NA | 643.7 | NA |
| 1025 | P15958 | NU5C | **NAD(P)H-quinone oxidoreductase subunit 5, chloroplastic** | 68.5 | Treated_T3 | 0.0 | 7.0 | NA | 445.6 | NA |
| 1026 | P48534 | APX1 | **L-ascorbate peroxidase, cytosolic** | 53.3 | Treated_T3 | 0.0 | 4.0 | NA | 714.5 | NA |
| 1027 | P04793 | HSP13 | **17.5 kDa class I heat shock protein** | 155.2 | Treated_T3 | 0.0 | 10.0 | NA | 462.4 | NA |
| 1028 | P14848 | LGB2 | **Leghemoglobin 2** | 413.5 | Treated_T3 | 0.0 | 7.0 | NA | 667.2 | NA |
| 1029 | Q09134 | GRPA | **Abscisic acid and environmental stress-inducible protein** | 26.8 | Treated_T3 | 0.0 | 3.0 | NA | 632.0 | NA |
| 1030 | Q09131 | PPAF | **Purple acid phosphatase** | 57.8 | Treated_T3 | 0.0 | 5.0 | NA | 834.4 | NA |
| 1031 | Q41050 | OEP16 | **Outer envelope pore protein 16, chloroplastic** | 111.2 | Treated_T3 | 0.0 | 3.0 | NA | 893.4 | NA |
| 1032 | P28011 | AAT1 | **Aspartate aminotransferase 1** | 52.3 | Treated_T3 | 0.0 | 4.0 | NA | 304.1 | NA |
| 1033 | P28010 | LGB4 | **Leghemoglobin** | 174.5 | Treated_T3 | 0.0 | 5.0 | NA | 437.9 | NA |
| 1034 | P02236 | LGB2 | **Leghemoglobin C2** | 35.4 | Treated_T3 | 0.0 | 3.0 | NA | 862.9 | NA |
| 1035 | Q6DW75 | DGDG2 | **Digalactosyldiacylglycerol synthase 2, chloroplastic** | 18.3 | Treated_T3 | 0.0 | 6.0 | NA | 260.9 | NA |
| 1036 | P25096 | P21 | **Protein P21** | 12.8 | Treated_T3 | 0.0 | 1.0 | NA | 316.7 | NA |
| 1037 | Q43560 | PR1 | **Class-10 pathogenesis-related protein 1** | 212.1 | Treated_T3 | 0.0 | 5.0 | NA | 920.2 | NA |
| 1038 | P52389 | CDC2 | **Cell division control protein 2 homolog** | 80.9 | Treated_T3 | 0.0 | 7.0 | NA | 731.3 | NA |
| 1039 | Q06197 | IDHC | **Isocitrate dehydrogenase [NADP]** | 71.9 | Treated_T3 | 0.0 | 6.0 | NA | 329.4 | NA |
| 1040 | P11894 | RK9 | **50S ribosomal protein L9, chloroplastic** | 210.0 | Treated_T3 | 0.0 | 9.0 | NA | 378.1 | NA |
| 1041 | P11893 | RK24 | **50S ribosomal protein L24, chloroplastic** | 52.5 | Treated_T3 | 0.0 | 6.0 | NA | 755.7 | NA |
| 1042 | Q6UD73 | LYK3 | **LysM domain receptor-like kinase 3** | 69.3 | Treated_T3 | 0.0 | 10.0 | NA | 661.9 | NA |
| 1043 | Q41009 | TOC34 | **Translocase of chloroplast 34** | 169.3 | Treated_T3 | 0.0 | 10.0 | NA | 869.8 | NA |
| 1044 | Q41005 | CBPX | **Serine carboxypeptidase-like (Fragment)** | 29.2 | Treated_T3 | 0.0 | 2.0 | NA | 218.3 | NA |
| 1045 | Q35638 | RHO1 | **Rac-like GTP-binding protein RHO1** | 87.0 | Treated_T3 | 0.0 | 6.0 | NA | 482.7 | NA |
| 1046 | O65874 | MTF1 | **MADS-box transcription factor 1** | 91.8 | Treated_T3 | 0.0 | 4.0 | NA | 521.2 | NA |
| 1047 | P19329 | ARC1 | **Arcelin-1** | 152.9 | Treated_T3 | 0.0 | 3.0 | NA | 105.7 | NA |
| 1048 | P16064 | ICI1 | **Subtilisin inhibitor 1** | 860.5 | Treated_T3 | 0.0 | 3.0 | NA | 120.4 | NA |
| 1049 | P16059 | PSBP | **Oxygen-evolving enhancer protein 2, chloroplastic** | 68.6 | Treated_T3 | 0.0 | 7.0 | NA | 264.2 | NA |
| 1050 | P02853 | PHSB | **Phaseolin, beta-type** | 18.0 | Treated_T3 | 0.0 | 4.0 | NA | 657.0 | NA |
| 1051 | O81974 | C71D8 | **Cytochrome P450 71D8** | 11.2 | Treated_T3 | 0.0 | 5.0 | NA | 329.3 | NA |
| 1052 | Q41669 | MT1A | **Metallothionein-like protein 1A** | 76.7 | Treated_T3 | 0.0 | 2.0 | NA | 111.7 | NA |
| 1053 | A6XNC6 | UGFGT | **Flavonoid 3-O-glucosyltransferase** | 129.6 | Treated_T3 | 0.0 | 7.0 | NA | 602.4 | NA |
| 1054 | P27880 | HSP12 | **18.2 kDa class I heat shock protein** | 206.4 | Treated_T3 | 0.0 | 7.0 | NA | 768.5 | NA |
| 1055 | Q43814 | OTC | **Ornithine carbamoyltransferase, chloroplastic** | 139.6 | Treated_T3 | 0.0 | 9.0 | NA | 979.8 | NA |
| 1056 | P16002 | PLAS | **Plastocyanin, chloroplastic** | 26.7 | Treated_T3 | 0.0 | 3.0 | NA | 251.2 | NA |
| 1057 | Q8W171 | CYP1 | **Peptidyl-prolyl cis-trans isomerase 1** | 127.7 | Treated_T3 | 0.0 | 4.0 | NA | 435.5 | NA |
| 1058 | Q06445 | CYTI | **Cysteine proteinase inhibitor** | 103.3 | Treated_T3 | 0.0 | 4.0 | NA | 754.4 | NA |
| 1059 | I1LCI8 | FG2KO | **UDP-glycosyltransferase 79A6** | 49.1 | Treated_T3 | 0.0 | 11.0 | NA | 294.6 | NA |
| 1060 | Q9SWF9 | ZFNL | **Zinc finger CCCH domain-containing protein ZFN-like** | 29.8 | Treated_T3 | 0.0 | 6.0 | NA | 55.7 | NA |
| 1061 | P49252 | AMO | **Primary amine oxidase (Fragment)** | 66.0 | Treated_T3 | 0.0 | 14.0 | NA | 458.6 | NA |
| 1062 | P25985 | PR1 | **Pathogenesis-related protein 1** | 118.3 | Treated_T3 | 0.0 | 3.0 | NA | 266.8 | NA |
|  |  |  |  |  |  |  |  |  |  |  |

Table S4. List of all proteins expressed in roots of 500 mM NaCl treated *Pongamia*. Two-way ANOVA test was performed to measure *P*-values ns (not significant), *(*P* < 0.05), **(*P* < 0.01) and ***(*P* < 0.001) respectively.
